# Regioselective Synthesis of 5-Trifluoromethyl 1,2,4-Triazoles via [3 + 2]-Cycloaddition of Nitrile Imines with CF_3_CN

**DOI:** 10.3390/molecules27196568

**Published:** 2022-10-04

**Authors:** Bo Lin, Zipeng Zhang, Yunfei Yao, Yi You, Zhiqiang Weng

**Affiliations:** 1 Key Laboratory of Molecule Synthesis and Function Discovery, Fujian Provincial Key Laboratory of Electrochemical Energy Storage Materials, College of Chemistry, Fuzhou University, Fuzhou 350108, China; 2 Fujian Engineering Research Center of New Chinese Lacquer Material, College of Materials and Chemical Engineering, Minjiang University, Fuzhou 350108, China

**Keywords:** trifluoromethyl, triazole, cycloaddition, nitrile imines, trifluoroacetonitrile

## Abstract

We herein describe a general approach to 5-trifluoromethyl 1,2,4-triazoles via the [3 + 2]-cycloaddition of nitrile imines generated in situ from hydrazonyl chloride with CF_3_CN, utilizing 2,2,2-trifluoroacetaldehyde *O*-(aryl)oxime as the precursor of trifluoroacetonitrile. Various functional groups, including alkyl-substituted hydrazonyl chloride, were tolerated during cycloaddition. Furthermore, the gram-scale synthesis and common downstream transformations proved the potential synthetic relevance of this developed methodology.

## 1. Introduction

**Figure 1:**
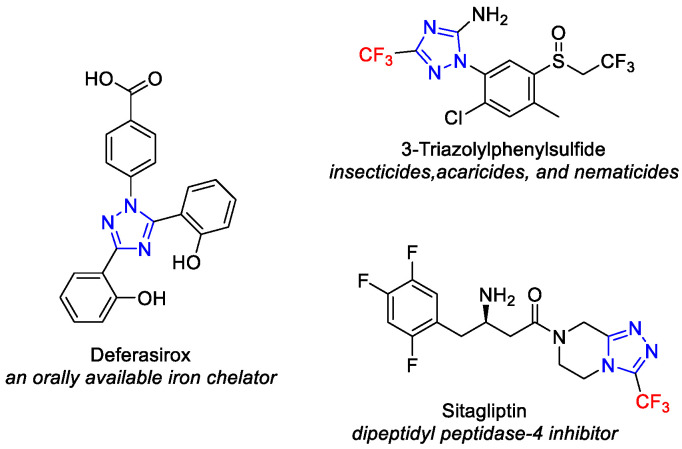
Representative examples of potent molecules bearing 1,2, 4-triazole scaffold.

1,2,4-Triazoles, five-membered heterocycles with three nitrogen atoms, prevalent in both natural and synthetic molecules, find use as drugs, insecticides, and synthetic materials. Their diverse biological activities in natural products and therapeutic agents include antifungal, antibacterial, antitubercular, antiviral, anti-inflammatory, anticancer, and analgesic activities [1,2,3,4]. Some potent molecules, such as deferasirox [5], 3-triazolylphenylsulfide [6], and sitagliptin [7], contain 1,2,4-triazole as the core structural framework (Figure 1).

Among these 1,2,4-triazoles, trifluoromethylated 1,2,4-triazoles have attracted significant attention [8]. It has been well established that the introduction of trifluoromethyl into therapeutic compounds can impart beneficial effects to lipophilicity, metabolic stability, conformational preference, and bioavailability [9,10,11]. As a consequence, a broad range of protocols for the construction of trifluoromethylated 1,2,4-triazoles have been disclosed [12,13,14,15,16,17,18,19,20,21,22,23,24,25,26,27,28].

**Scheme 1:**
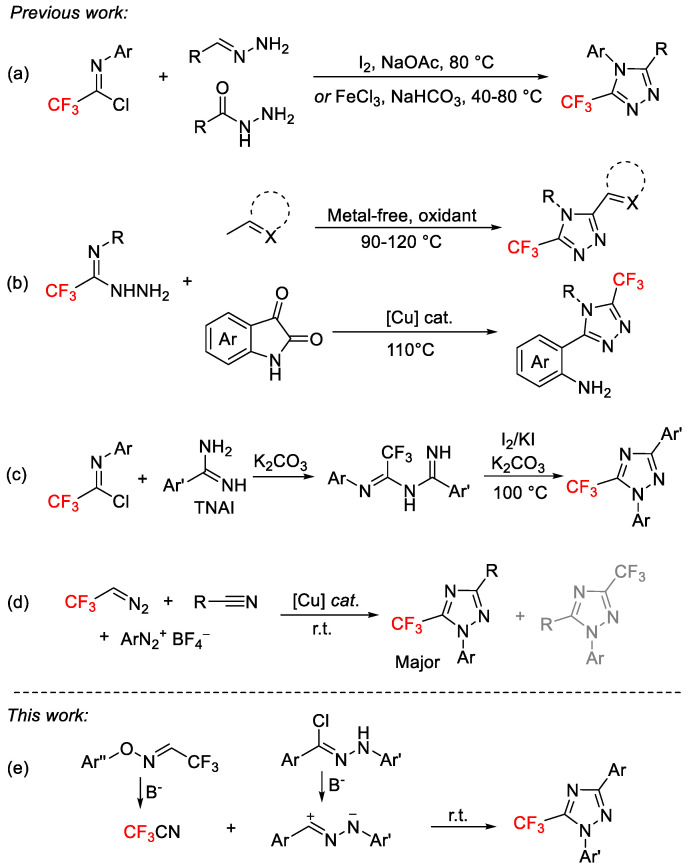
Methods for the preparation of 5-trifluoromethyl 1,2,4-triazoles.(**a**) FeCl3-mediated cascade annulation of trifluoroacetimidoyl chlorides (**b**) Copper-catalyzed intramolecular decarbonylative cyclization reaction of trifluoroacetimidohydrazides (**c**) Iodine-mediated intramolecular oxidative cyclization of N-(2,2,2-trifluoro-1-(arylimino)ethyl)benzimidamide (**d**) Copper-catalyzed three-component reaction of aryldiazonium salts with 2,2,2-trifluorodiazoethane and nitriles (**e**) [3 + 2]-Cycloaddition of nitrile imines with CF3CN.

However, current synthetic approaches for 5-trifluoromethyl 1,2,4-triazoles are scarce. Wu and co-workers reported a iodine-mediated annulation and a FeCl_3_-mediated cascade annulation of trifluoroacetimidoyl chlorides and hydrazones or hydrazides for the synthesis of 5-trifluoromethyl-1,2,4-triazoles (Scheme 1a) [29,30]. Wu and co-workers subsequently developed a metal-free oxidative cyclization and a copper-catalyzed intramolecular decarbonylative cyclization reaction of trifluoroacetimidohydrazides with methylhetarenes or isatins for the synthesis of 5-trifluoromethyl-1,2,4-triazoles [31,32,33] and 2-(5-trifluoromethyl-1,2,4-triazol-3-yl)aniline derivatives [34], respectively. Darehkordi and co-workers described the synthesis of 1,3-diaryl-5-(trifluoromethyl)-1*H*-1,2,4-triazoles via the iodine-mediated intramolecular oxidative cyclization of *N*-(2,2,2-trifluoro-1-(arylimino)ethyl)benzimidamide intermediates, synthesized from the reaction of *N*-aryl-2,2,2-trifluoroacetimidoyl chlorides and benzamide hydrochloride derivatives (Scheme 1c) [35]. More recently, the Ma research group developed a copper-catalyzed three-component reaction of aryldiazonium salts with fluorinated diazo reagents and nitriles, leading to two regiomers of trifluoromethylated N^1^-aryl-1,2,4-triazoles (Scheme 1d) [36]. Although these previous reports were found to be effective, they suffered from some limitations, such as utilizing transition-metal catalysis, the use of the explosiveness of diazo and diazonium reagents, the complexity of the reaction mixture, and poor regioselectivities. Therefore, there is still a need for additional methodologies to access this important chemical scaffold from readily available starting materials under mild conditions.

As part of our ongoing interest centered on the synthesis of trifluoromethylated heterocycles [37], we recently disclosed a novel protocol for the construction of trifluoromethylated oxadiazoles from the reaction of nitrile oxide derivatives using 2,2,2-trifluoroacetaldehyde *O*-(aryl)oxime as the precursor of trifluoroacetonitrile [38]. Based on these results and considering that nitrile imines, generated in situ via the treatment of the hydrazonyl halides with stoichiometric amounts of base [39], could serve as the attractive C1N2 synthons in [3 + 2] cycloaddition reactions [40,41], we now investigated the feasibility of this approach for the regioselective synthesis of 5-trifluoromethyl 1,2,4-triazoles (Scheme 1e).

## 2. Results

Our study commenced by choosing *N*-phenyl-benzohydrazonoyl chloride, **2a**, and trifluoroacetaldehyde *O*-(2,4-dinitrophenyl) oxime, **1**, as the model substrates (Table 1), using our previously reported optimized conditions for the synthesis of trifluoromethylated oxadiazoles [38]. To our delight, the reaction of **2a** with **1** in a 1:2 ratio in the presence of 2.0 equiv of NEt_3_ afforded the desired product, 5-trifluoromethyl 1,2,4-triazole, **3a**, in 3% and 8% yields in THF and DMSO, respectively (Entries 1 and 2). Among other solvents (Entries 3–6), the reaction worked best in CH_2_Cl_2_ (Entry 5), whereas in toluene, the reaction could not proceed (Entry 6). Notably, upon increasing the amount of **2a** and keeping the other parameters constant, the yield of **3a** was further improved (37% and 49% with **2a** and **1** in 1:1 and 2:1 ratios, respectively; Entries 7 and 8). Gratifyingly, the yield of **3a** was further improved to 75% by increasing the amount of NEt_3_ (3.0 equiv) (Entry 9). The best yield (83%) was obtained with the reaction of **2a** with **1** in a 1.5:1 ratio in the presence of 3.0 equiv of NEt_3_ in CH_2_Cl_2_ (Entry 10).

**Figure 2:**
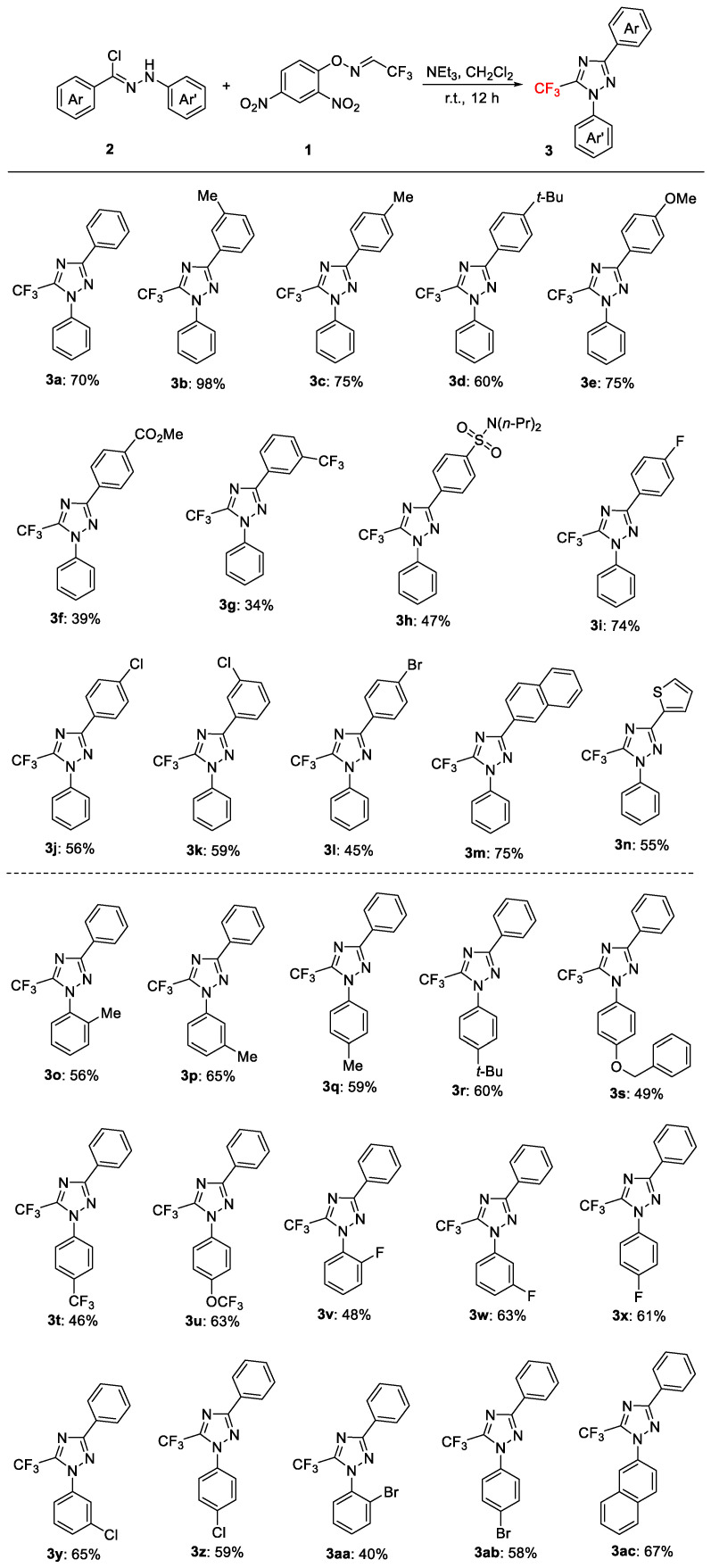
Scope of the hydrazonyl chlorides. Reaction conditions: **2** (0.30 mmol), **1** (0.20 mmol), NEt_3_ (0.60 mmol, 3.0 equiv), CH_2_Cl_2_ (1.0 mL), r.t., 12 h, in benchtop air atmosphere. Isolated yields (calculated on basis of starting material **1**).

Subsequently, the efficacy of this developed protocol for the regioselective synthesis of 5-trifluoromethyl 1,2,4-triazoles with various hydrazonyl chlorides **2** was investigated (Figure 2). The reaction of 3-, or 4-Me-, and 4-*t*-Bu-substituted on the Ar moieties of hydrazonyl chlorides **2b**–**2d** with **1** provided annulated products **3b**–**3d** in 98%, 75%, and 60% yields, respectively. The variation in the electronic properties of the aryl substituents in the hydrazonyl chlorides had a significant effect on the reaction efficiency. The reaction of the hydrazonyl chloride possessing a strong electron-donating group such as -OMe (**2e**) successfully provided the corresponding triazole, **3e**, with good yield (75%). On the other hand, the substrates (**2f** and **2g**) containing strongly electron-withdrawing substituents (-CO_2_Me, -CF_3_, and –SO_2_N(*n*-Pr)_2_) furnished the desired triazoles (**3f**–**3h**) in moderate yields. These results are consistent with the previous observations in the synthesis of 1,2,4-triazol-3-ones by Wu and co-workers [42]. Of note, the halogen substituents, such as fluoro, and chloro, were also tested for the cycloaddition, and to our delight, good yields (56–74%) of the corresponding triazoles products, **3i**–**3l**, were obtained. A 2-naphthyl-substituted hydrazonyl chloride also furnished the desired product, **3m**, in 75% yield. Likewise, 2-thienyl substituted hydrazonyl chloride also smoothly underwent a reaction with **1**, giving the desired product, **3n**, in a 55% yield.

Meanwhile, cycloaddition was tested with different substituents at the Ar′ moieties of the hydrazonyl chlorides. Under the optimized conditions, a wide range of hydrazonyl chlorides contained different functional groups, such as methyl, *tert*-butyl, benzyloxy, trifluoromethyl, trifluoromethoxy, fluoro, chloro, and bromo groups, which were well tolerated and afforded triazole products **3o**–**3ab** in good yields (40–65%). 2-naphthyl-substituted hydrazonyl chloride was also efficient as a substrate to produce corresponding product **3ac** in a 67% yield. 

**Figure 3:**
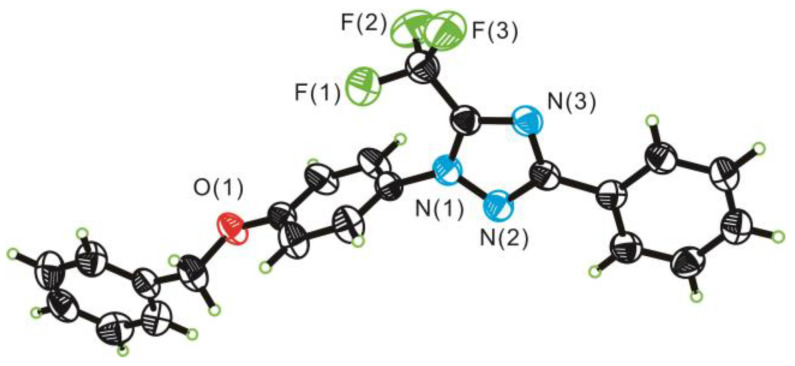
ORTEP diagram of **3s** with thermal ellipsoids at the 40% probability level.

The structures of products **3a**–**3ac** were confirmed by the IR spectroscopy, and ^1^H, ^13^C{^1^H}, ^19^F NMR spectroscopy (Supplementary Materials). Additionally, the structure of **3s** was unambiguously confirmed via single-crystal X-ray diffraction analyses (Figure 3).

**Figure 4:**
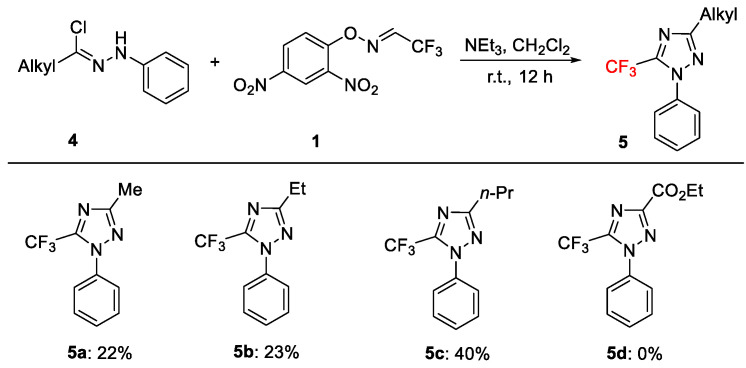
Scope of alkyl-substituted hydrazonyl chlorides. Reaction conditions: **4** (0.30 mmol), **1** (0.20 mmol), NEt_3_ (0.60 mmol, 3.0 equiv), CH_2_Cl_2_ (1.0 mL), r.t., 12 h, in benchtop air atmosphere. Isolated yields (calculated on basis of starting material **1**).

Next, we attempted the cycloaddition of alkyl-substituted hydrazonyl chlorides **4** with **1** (Figure 4). Under the optimized conditions, both methyl, ethyl, and *n*-propyl-substituted hydrazonyl chlorides furnished the desired products, **5a**–**5c**, in only moderate yields (22–40%), which could be a result of the inherent instability of the hydrazonyl chloride substrates with an alkyl substituent. However, ester-substituted hydrazonyl chloride **4d** did not result in the desired cyclized product, **5d**, under our standard or modified reaction conditions, probably due to the resulting decreased nucleophilicity of the nitrogen atom through the conjugated system from the nitrile imine intermediates.

**Scheme 2:**
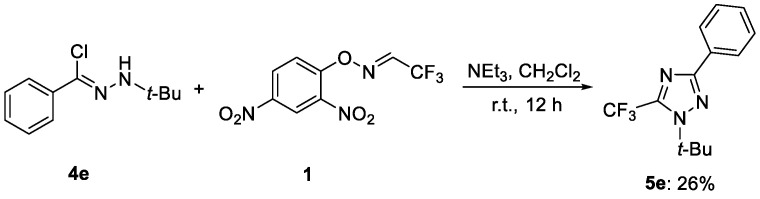
Synthesis of **5e**.

Nevertheless, a hydrazonyl chloride with a *t*-butyl substituent on the nitrogen atom reacted with **1** to produce the corresponding product, **5e**, in a 26% yield (Scheme 2).

**Scheme 3:**
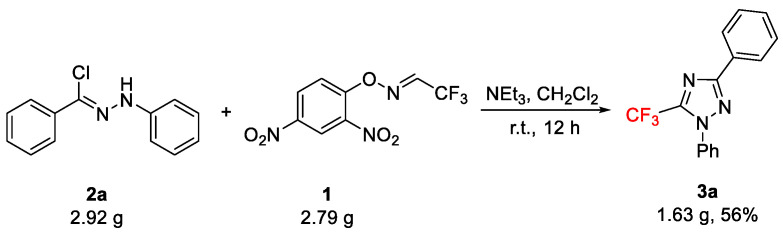
Gram-scale synthesis of **3a**.

To assess the scale-up of the procedure, the reaction of **2a** with **1** as the representative example was investigated on a 10.0 mmol scale (Scheme 3). Under the optimized reaction conditions, the cycloaddition occurred to give **3a** in a 56% (1.63 g) yield.

**Scheme 4:**
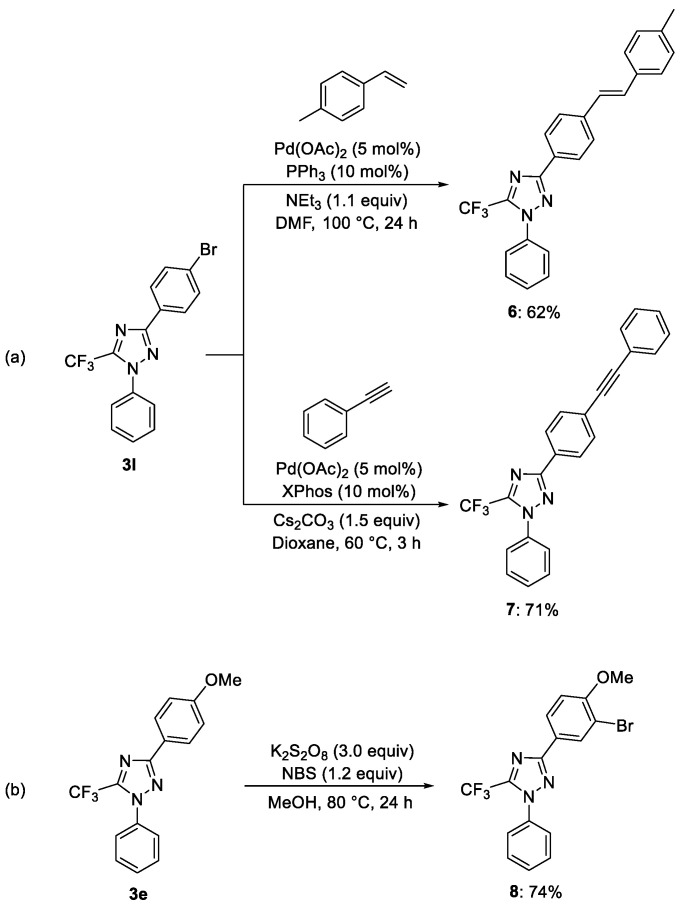
Synthetic utility. (**a**) Heck reaction and Sonogashira coupling with 3l (**b**) Bromination of 3e.

To further demonstrate the synthetic utility of the products, Heck reaction and Sonogashira coupling of bromide **3l** were carried out with *p*-methylstyrene and phenylacetylene to afford the corresponding products, **6** and **7**, in 62% and 71% yields, respectively (Scheme 4a). Finally, the bromination of the C–H bond of **3e** was performed under oxidative conditions to furnish product **8** in a 74% yield (Scheme 4b).

**Scheme 5:**
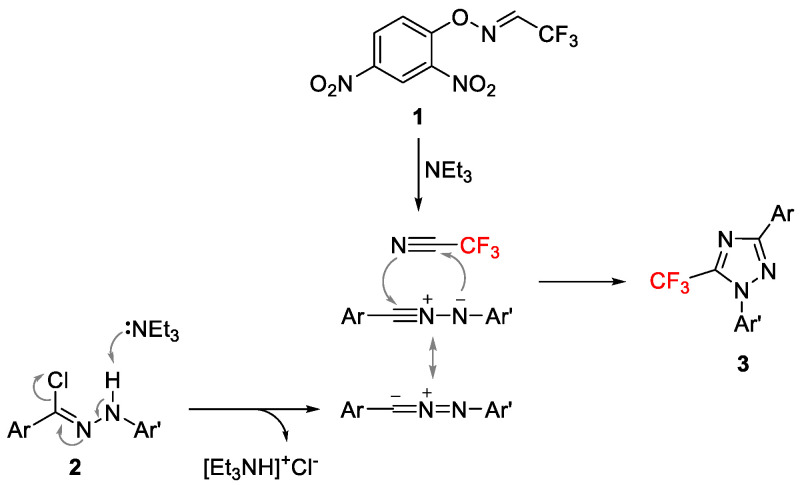
Proposed mechanism for the formation of **3**.

Based on the results obtained from these experiments and literature reports [38,43], a plausible mechanism for the formation of 5-trifluoromethyl 1,2,4-triazole (**3**) was proposed (Scheme 5). The nitrile imine generated in situ from hydrazonyl chloride **2** in the presence of a base underwent regioselective [3 + 2] cycloaddition with the in situ-generated CF_3_CN from **1** to generate the desired product, **3**.

## 3. Materials and Methods

^1^H NMR, ^19^F NMR, and ^13^C NMR spectra were recorded using a Bruker AVIII 400 spectrometer. ^1^H NMR and ^13^C NMR chemical shifts were reported in parts per million (ppm) downfield from tetramethylsilane, and ^19^F NMR chemical shifts were determined relative to CFCl_3_ as the external standard; low field was positive. Coupling constants (*J*) are reported in Hertz (Hz). The residual solvent peak was used as an internal reference: ^1^H NMR (CDCl_3_ δ 7.26), ^13^C NMR (CDCl_3_ δ 77.0). The following abbreviations were used to explain the multiplicities: s = singlet, d = doublet, t = triplet, q = quartet, m = multiplet, br = broad. The infrared (IR) spectra were recorded using Nicolet iS50 at room temperature. HRMS were obtained from State Key Discipline Testing Center for Physical Chemistry of Fuzhou University. Trifluoroacetaldehyde *O*-(2,4-dinitrophenyl) oxime **1** [38], and *N′*-phenylacylhydrazides and hydrazonoyl chlorides [44,45] were prepared according to the published procedures. Starting materials and solvents that were received from commercial sources were used without further purification. Column chromatography purifications were performed via flash chromatography using Merck silica gel 60.

**Caution**: It is known that trifluoroacetonitrile is a highly toxic gas (boiling point, −64 °C) and must be handled with care. The rapid evolution of CF_3_CN gas occurs when this precursor reacts with base. All operations were performed in a fume hood under good conditions.

### General Procedure for the Synthesis of 5-Trifluoromethyl 1,2,4-Triazoles 3

A mixture of hydrazonoyl chloride, **2** (0.30 mmol, 1.5 equiv), and trifluoroacetaldehyde *O*-(2,4-dinitrophenyl) oxime, **1** (54.1 mg, 0.20 mmol, 1.0 equiv), in CH_2_Cl_2_ (1.0 mL) was added to a Schlenk tube equipped with a stir bar. Then, NEt_3_ (60.6 mg, 83.2 μL, 0.60 mmol, 3.0 equiv) was added. The tube was immediately sealed with a Teflon cap and stirred at room temperature for 12 h. After the reaction was terminated, the solvent was removed in vacuo under reduced pressure. Product **3** was purified via flash column chromatography on silica gel with petroleum ether and CH_2_Cl_2_ as eluent.

### Crystal Structure Analyses

The suitable crystals of **3s** were mounted on quartz fibers and X-ray data collected on a Bruker AXS APEX diffractometer, equipped with a CCD detector at −50 ºC, using MoKα radiation (λ 0.71073 Å). The data was corrected for Lorentz and polarisation effect with the SMART suite of programs and for absorption effects with SADABS [46]. Structure solution and refinement were carried out with the SHELXTL suite of programs. The structure was solved by direct methods to locate the heavy atoms, followed by difference maps for the light non-hydrogen atoms. CCDC 2183507 contain the supplementary crystallographic data. These data can also be obtained free of charge at ccdc.cam.ac.uk/structures/ from the Cambridge Crystallographic Data Centre.

## 4. Conclusions

In summary, we accomplished a [3 + 2]-cycloaddition of nitrile imines with CF_3_CN for the synthesis of 5-trifluoromethyl 1,2,4-triazoles utilizing 2,2,2-trifluoroacetaldehyde *O*-(aryl)oxime as the precursor of trifluoroacetonitrile. The exclusive regioselectivity, functional group tolerance, mild reaction conditions, and efficient scalability are the important practical advantages.

## Figures and Tables

**Table 1:**
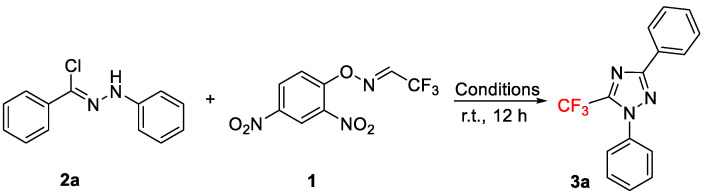
Optimization of the reaction conditions *^a^*.

Entry	2a:1	Base (equiv)	Solvent	Yield of 3a (%) *^b^*
1	1:2	NEt_3_ (2)	THF	3
2	1:2	NEt_3_ (2)	DMSO	8
3	1:2	NEt_3_ (2)	DMF	22
4	1:2	NEt_3_ (2)	MeCN	19
5	1:2	NEt_3_ (2)	CH_2_Cl_2_	25
6	1:2	NEt_3_ (2)	Toluene	0
7	1:1	NEt_3_ (2)	CH_2_Cl_2_	37
8	2:1	NEt_3_ (2)	CH_2_Cl_2_	49
9	2:1	NEt_3_ (3)	CH_2_Cl_2_	75
10	1.5:1	NEt_3_ (3)	CH_2_Cl_2_	83

*^a^* Reaction conditions: **2a** (0.20–0.30 mmol), **1** (0.20–0.40 mmol), base (0.40 mmol, 3.0 equiv), solvent (1.0 mL), r.t., 12 h, in benchtop air atmosphere. *^b^* The yield was determined using ^19^F NMR spectroscopy with PhOCF_3_ as the internal standard.

## Data Availability

The data presented in this study are available in this article.

## Supplementary Materials

The following supporting information can be downloaded at: https://www.mdpi.com/article/10.3390/molecules27196568/s1, Experimental procedures and copies of spectroscopic characterization of all new compounds.

## Sample Availability

Samples of the compounds **3**, **5**, and **6**–**8** are available from the authors.

## References

[1] Shalini K., Kumar N., Drabu S., Sharma P.K. (2011). Advances in Synthetic Approach to and Antifungal Activity of Triazoles. Beilstein J. Org. Chem..

[2] Zhou C.H., Wang Y. (2012). Recent Researches in Triazole Compounds as Medicinal Drugs. Curr. Med. Chem..

[3] Aggarwal R., Sumran G. (2020). An Insight on Medicinal Attributes of 1,2,4-Triazoles. Eur. J. Med. Chem..

[4] Strzelecka M., Świątek P. (2021). 1,2,4-Triazoles as Important Antibacterial Agents. Pharmaceuticals.

[5] Valentovic M., Enna S.J., Bylund D.B. (2008). Deferasirox. xPharm: The Comprehensive Pharmacology Reference.

[6] Toriyabe K., Yamaguchi M., Itou Y., Kinpara S., Yano H., Takahashi S., Yonekura N., Hamaguchi R. (2006). Preparation of 3-Triazolylphenylsulfide Compounds as Insecticides/Acaricides/Nematicides for Agricultural or Horticultural Plants. Patent.

[7] Plosker G.L. (2014). Sitagliptin: A review of its use in patients with type 2 diabetes mellitus. Drugs.

[8] Ostrovskii V.A., Trifonov R.E., Nenajdenko V. (2014). Fluorinated Triazoles and Tetrazoles. Fluorine in Heterocyclic Chemistry.

[9] Hagmann W.K. (2008). The Many Roles for Fluorine in Medicinal Chemistry. J. Med. Chem..

[10] Wang J., Sánchez-Roselló M., Aceña J.L., del Pozo C., Sorochinsky A.E., Fustero S., Soloshonok V.A., Liu H. (2014). Fluorine in Pharmaceutical Industry: Fluorine-Containing Drugs Introduced to the Market in the Last Decade (2001–2011). Chem. Rev..

[11] Gillis E.P., Eastman K.J., Hill M.D., Donnelly D.J., Meanwell N.A. (2015). Applications of Fluorine in Medicinal Chemistry. J. Med. Chem..

[12] Brown H.C., Cheng M.T. (1962). Nucleophilic Attack on the 2,5-Bis(perfluoroalkyl)-1,3,4-oxadiazoles. I. Synthesis of 3,5-Bis(perfluoroalkyl)-1,2,4-triazoles and 4-Methyl-1,2,4,4H-triazoles1. J. Org. Chem..

[13] Czollner L., Szilgyi G., Lang J., Janky J. (1988). 1,2,4-Triazoles, II Synthesis of 1,5-diphenyl-3-trifluoromethyl-1H-1,2,4-triazoles. Monatsh. Chem..

[14] Reitz D.B., Finkes M.J. (1989). Reaction of 2,5-bis(trifluoromethyl)-1,3,4-oxadiazole with hydrazine. The synthesis of 4-amino-3,5-bis(trifluoromethyl)-4H-1,2,4-triazole. J. Org. Chem..

[15] Reitz D.B., Finkes M.J. (1989). Reaction of 2,5-bis(trifluoromethyl)-1,3,4-oxadiazole with primary amines. Synthesis of 4-substituted-3,5-bis(trifluoromethyl)-4H-1,2,4-triazoles. J. Heterocycl. Chem..

[16] Abdul-Ghani M., Tipping A.E. (1990). The Synthesis from 2.5-Dichloro-1.1.1.6.6,6-hexafluoro-3.4-diazahexa-2.4- diene of 4H-3.5-Bis(trifluoromethyl)-1.2,4-triazole and some 1- and 4-Substituted Derivatives. J. Fluor. Chem..

[17] Uneyama K., Sugimoto K. (1992). N-Substituted 2,2,2-trifluoroethanimidic acid 1-methylethylidene hydrazides as synthetic blocks for trifluoromethylated nitrogen heterocycles. J. Org. Chem..

[18] Funabiki K., Noma N., Kuzuya G., Matsui M., Shibata K. (1999). A Direct and General Synthesis of 5-Substituted 3-Trifluoromethyl-1,2,4-triazoles via the Three Component Condensation Reaction of Ethyl Trifluoroacetate, Hydrazine and Amidines. J. Chem. Res..

[19] Buscemi S., Pace A., Pibiri I., Vivona N., Spinelli D. (2003). Fluorinated heterocyclic compounds. An expedient route to 5-perfluoroalkyl-1,2,4-triazoles via an unusual hydrazinolysis of 5-perfluoroalkyl-1,2,4-oxadiazoles. J. Org. Chem..

[20] Xue H., Twamley B., Shreeve J.n.M. (2004). The first 1-alkyl-3-perfluoroalkyl-4,5- dimethyl-1,2,4-triazolium salts. J. Org. Chem..

[21] Sibgatulin D., Bezdudny A., Mykhailiuk P., Voievoda N., Kondratov I., Volochnyuk D., Tolmachev A. (2010). Novel Synthetic Approaches to (Trifluoromethyl)triazoles. Synthesis.

[22] Grünebaum M., Gerlitz A.I., Buchheit A., Jeschke S., Daniliuc C.G., Wiemhöfer H.-D. (2016). Improved synthesis of perfluoroalkyl substituted 1, 3, 4-oxadiazoles as precursors for corresponding 1, 2, 4-triazoles. J. Fluor. Chem..

[23] Zhang J.-W., Wang Y.-R., Pan J.-H., He Y.-H., Yu W., Han B. (2020). Deconstructive Oxygenation of Unstrained Cycloalkanamines. Angew. Chem. Int. Ed..

[24] Yan Z., Lu T., Liu Y., Liu W., Zhao B., Wang Y., Ge Z. (2021). High Thermal Stability and Insensitive Fused Triazole-Triazine Trifluoromethyl-Containing Explosives (TFX). ACS Omega.

[25] Zhang F., Peng X., Ma J.A. (2019). Recent Advances in the Synthesis of CF_3_-Substituted Triazoles and Tetrazoles. Chin. J. Org. Chem..

[26] Yang H., Lu S.-N., Song Y., Chen Z., Wu X.-F. (2021). Copper-mediated [3 + 2] cycloaddition of trifluoroacetimidoyl chlorides and N -isocyanoiminotriphenylphosphorane for the synthesis of 3-trifluoromethyl-1,2,4-triazoles. Org. Chem. Front..

[27] Lu S.N., Yang H., Zhang J., Chen Z., Wu X.F. (2021). Oxidative Cyclization of Trifluoroacetimidohydrazides with D-Glucose for the Metal-Free Synthesis of 3-Trifluoromethyl-1,2,4-Triazoles. Adv. Synth. Catal..

[28] Liu X., Liu H., Bian C., Wang K.-H., Wang J., Huang D., Su Y., Lv X., Hu Y. (2022). Synthesis of 3-Trifluoromethyl-1,2,4-triazolines and 1,2,4-Triazoles via Tandem Addition/Cyclization of Trifluoromethyl N-Acylhydrazones with Cyanamide. J. Org. Chem..

[29] Hu S., Yang Z., Chen Z., Wu X.F. (2019). Metal-Free Synthesis of 5-Trifluoromethyl-1,2,4-Triazoles from Iodine-Mediated Annulation of Trifluoroacetimidoyl Chlorides and Hydrazones. Adv. Synth. Catal..

[30] Du S., Wang L.C., Yang Z., Chen Z., Wu X.F. (2020). A Convenient FeCl_3_-Mediated Synthesis of 5-Trifluoromethyl-1,2,4-triazoles from Trifluoroacetimidoyl Chlorides and Hydrazides. Adv. Synth. Catal..

[31] Zhang J., Xu T.-H., Chen Z., Wu X.-F. (2021). Metal-free oxidative cyclization of trifluoroacetimidohydrazides with methylhetarenes: A facile access to 3-hetaryl-5-trifluoromethyl-1,2,4-triazoles. Org. Chem. Front..

[32] Zhang J., Tang J., Chen Z., Wu X.F. (2021). Elemental Sulfur and Dimethyl Sulfoxide-Promoted Oxidative Cyclization of Trifluoroacetimidohydrazides with Methylhetarenes for the Synthesis of 3-Hetaryl-5-trifluoromethyl-1,2,4-triazoles. Chin. J. Chem..

[33] Zhang J., Tang J., Chen Z., Wu X.F. (2021). Synthesis of 5-Trifluoromethyl-1,2,4-Triazoles via Metal-Free Annulation of Trifluoroacetimidohydrazides and Methyl Ketones. Adv. Synth. Catal..

[34] Zhang Y., Yang Z., Chen Z., Liu L., Wu X.F. (2022). Copper-Catalyzed Decarbonylative Cyclization of Isatins and Trifluoroacetimidohydrazides for the Synthesis of 2-(5-Trifluoromethyl-1,2,4-triazol-3-yl)anilines. Adv. Synth. Catal..

[35] Kazemi E., Darehkordi A. (2020). Intramolecular oxidative cyclization of N-(2,2,2-trifluoro-1-(phenylimino)ethyl)benzimidamide. Mol. Divers..

[36] Peng X., Zhang F.G., Ma J.A. (2020). Cu-Catalysed Three-Component Reaction of Aryldiazonium Salts with Fluorinated Diazo Reagents and Nitriles. Adv. Synth. Catal..

[37] Wu W., You Y., Weng Z. (2022). Recent advances in the synthesis of fluoroalkylated compounds using fluoroalkyl anhydrides. Chin. Chem. Lett..

[38] Lin B., Yao Y., Huang Y., Weng Z. (2022). 2,2,2-Trifluoroacetaldehyde O-(Aryl)oxime: A Precursor of Trifluoroacetonitrile. Org. Lett..

[39] Huisgen R., Seidel M., Wallbillich G., Knupfer H. (1962). Diphenyl-nitrilimin und seine 1.3-dipolaren additionen an alkene und alkine. Tetrahedron.

[40] Deepthi A., Acharjee N., Sruthi S.L., Meenakshy C.B. (2022). An overview of nitrile imine based [3 + 2] cycloadditions over half a decade. Tetrahedron.

[41] Livingstone K., Little G., Jamieson C. (2021). Recent Advances in the Generation of Nitrilium Betaine 1,3-Dipoles. Synthesis.

[42] Du S., Wang W.-F., Song Y., Chen Z., Wu X.-F. (2021). Palladium-Catalyzed Cascade Carbonylative Synthesis of 1,2,4-Triazol-3-ones from Hydrazonoyl Chlorides and NaN_3_. Org. Lett..

[43] Zeng H., Fang X., Yang Z., Zhu C., Jiang H. (2021). Regioselective Synthesis of 5-Trifluoromethylpyrazoles by [3 + 2] Cycloaddition of Nitrile Imines and 2-Bromo-3,3,3-trifluoropropene. J. Org. Chem..

[44] Tirapegui C., Acevedo-Fuentes W., Dahech P., Torrent C., Barrias P., Rojas-Poblete M., Mascayano C. (2017). Easy and rapid preparation of benzoylhydrazides and their diazene derivatives as inhibitors of 15-lipoxygenase. Bioorg. Med. Chem. Lett..

[45] Yavari I., Khaledian O. (2020). A formal [3+2] cycloaddition reaction of N-methylimidazole as a masked hydrogen cyanide: Access to 1,3-disubstitued-1H-1,2,4-triazoles. Chem. Commun..

[46] (1997). SHELXTL.

