# Supplementary material for: Regioselective Synthesis of 5-Trifluoromethyl 1,2,4-Triazoles via [3 + 2]-Cycloaddition of Nitrile Imines with CF3CN
Source: Molecules. 2022 Oct 4;27(19):6568. doi: 10.3390/molecules27196568 (PMC9572902; doi:10.3390/molecules27196568)

Supporting information *for*

Regioselective Synthesis of 5-Trifluoromethyl 1,2,4-Triazoles via [3 + 2]-Cycloaddition of Nitrile Imines with CF_3CN

Bo Lin [1, ‡], Zipeng Zhang [1,‡], Yunfei Yao [1], Yi You[1,*], and Zhiqiang Weng [1,2,*]

1. Key Laboratory of Molecule Synthesis and Function Discovery, and Fujian Provincial Key Laboratory of Electrochemical Energy Storage Materials, College of Chemistry, Fuzhou University, Fuzhou, 350108, China.
2. Fujian Engineering Research Center of New Chinese lacquer Material, College of Materials and Chemical Engineering, Minjiang University, Fuzhou, 350108, China.

E-mail: zweng@mju.edu.cn

Table of contents

General information.. S2

Synthesis of hydrazonoyl chloride substrates…….. S3

General procedure for the synthesis of 5-trifluoromethyl 1,2,4-triazoles………. S7

Procedure for the gram-scale synthesis of **3a**……….. S8

Product derivatization……………………………………………………….... S9

Data for compounds……………………………………………………………... S12

Crystal structure analyses……………………………………………………... S32

References………………………………………………………….................... S35

Copies of ^{1}H NMR, ^{19}F NMR and ^{13}C NMR spectra…………………………… S36

General information

^{1}H NMR, ^{19}F NMR and ^{13}C NMR spectra were recorded using Bruker AVIII 400 spectrometer. ^{1}H NMR and ^{13}C NMR chemical shifts were reported in parts per million (ppm) downfield from tetramethylsilane and ^{19}F NMR chemical shifts were determined relative to $CFCl_3$ as the external standard and low field is positive. Coupling constants (*J*) are reported in Hertz (Hz). The residual solvent peak was used as an internal reference: ^{1}H NMR ($CDCl_3$ δ 7.26), ^{13}C NMR ($CDCl_3$ δ 77.0), The following abbreviations were used to explain the multiplicities: s = singlet, d = doublet, t = triplet, q = quartet, m = multiplet, br = broad. The infrared (IR) spectra were recorded using a Nicolet iS50 at room temperature. HRMS were obtained on State Key Discipline Testing Center for Physical Chemistry of Fuzhou University. Trifluoroacetaldehyde *O*-(2,4-dinitrophenyl) oxime **1** was prepared according to the published procedures.[1] Starting materials and solvents that were received from commercial sources were used without further purification. Column chromatography purifications were performed by flash chromatography using Merck silica gel 60.

Caution: It is known that trifluoroacetonitrile is a highly toxic gas (boiling point -64 °C) and must be handled with care. Rapid evolution of CF_3CN gas will occur when this precursor reacts with base. All operations were performed in fume hood in good condition.

Synthesis of hydrazonoyl chloride substrates

N'-phenylacylhydrazides and hydrazonoyl chlorides were prepared according to the published procedures.[2]

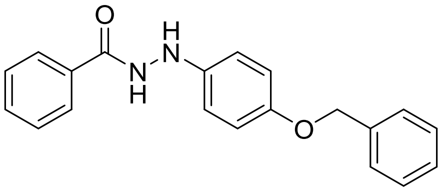

***N'*-(4-(benzyloxy)phenyl)benzohydrazide (S-2s)**

Obtained as a white solid in 60% yield (572.5 mg). R_f (petroleum ether/ethyl acetate = 10:1) = 0.36. Mp: 154.8 – 155.5 ℃. ^{1}H NMR (400 MHz, DMSO-d_6) δ 10.37 (s, 1H), 7.92 (d, *J* = 6.9 Hz, 2H), 7.63 (s, 1H), 7.61 – 7.54 (m, 1H), 7.53 – 7.46 (m, 2H), 7.46 – 7.35 (m, 4H), 7.34 – 7.28 (m, 1H), 6.87 (d, *J* = 7.4 Hz, 2H), 6.77 (d, *J* = 7.6 Hz, 2H), 5.00 (s, 2H). ^{13}C NMR (101 MHz, DMSO-d_6) δ 166.7 (s), 152.2 (s), 144.1 (s), 138.0 (s), 133.6 (s), 132.0 (s), 128.9 (s), 128.8 (s), 128.1 (s), 128.0 (s), 127.7 (s), 115.8 (s), 114.2 (s), 70.1 (s). IR (ATR): ν 3239, 3031, 2907, 2862, 1639, 1506, 1467, 1379, 1227, 1016, 913, 825 cm^{-1}. HRMS (ESI) m/z: calcd. for $C_{20}H_{19}N_2O_2$ [M + H]$^+$: 319.1441; found: 319.1435.

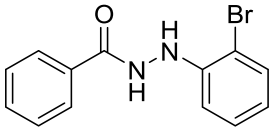

***N'*-(2-bromophenyl)benzohydrazide (S-2aa)**

Obtained as a white solid in 57% yield (1.60 g). R_f (petroleum ether/ethyl acetate = 10:1) = 0.76. Mp: 152.5 – 153.7 ℃. ^{1}H NMR (400 MHz, DMSO-d_6) δ 10.60 (s, 1H), 8.01 – 7.86 (m, 2H), 7.69 – 7.57 (m, 1H), 7.57 – 7.46 (m, 2H), 7.42 (s, 1H), 7.22 (s, 1H), 6.86 (d, *J* = 7.4 Hz, 1H), 6.73 (s, 1H). ^{13}C NMR (101 MHz, DMSO-d_6) δ 166.7 (s), 146.3 (s), 133.1 (s), 132.8 (s), 132.3 (s), 129.0 (s), 128.9 (s), 127.9 (s), 120.9 (s), 113.8 (s), 107.7 (s). IR (ATR): ν 3253, 1642, 1587, 1483, 1325, 1289, 1241, 1025, 902, 742 cm^{-1}. HRMS (ESI) m/z: calcd. for $C_{13}H_{12}BrN_2O$ [M + H]$^+$: 291.0128; found:

291.0126.

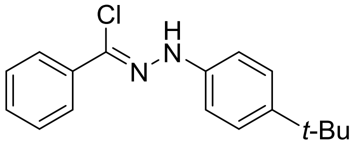

N-(4-($tert$-butyl)phenyl)benzohydrazonoyl chloride (2r)

Obtained as an orange solid in 51% yield (437.7 mg). R_f (petroleum ether) = 0.40. Mp: 65.8 – 66.9 ℃. ^{1}H NMR (400 MHz, $CDCl_3$) δ 8.16 (br s, 1H), 8.09 (d, J = 7.5 Hz, 2H), 7.63 – 7.42 (m, 5H), 7.28 (d, J = 8.2 Hz, 2H), 1.50 (s, 9H). ^{13}C NMR (101 MHz, $CDCl_3$) δ 144.0 (s), 141.0 (s), 134.6 (s), 129.1 (s), 128.4 (s), 126.4 (s), 126.2 (s), 124.1 (s), 113.2 (s), 34.2 (s), 31.6 (s). IR (ATR): ν 3313, 2962, 2865, 1609, 1515, 1445, 1317, 1234, 942, 825, 756, 688 cm^{-1}. HRMS (ESI) m/z: calcd. for $C_{17}H_{20}ClN_2$ [M + H]$^+$: 287.1310; found: 287.1306.

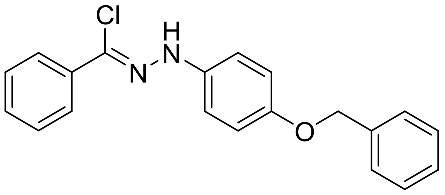

N-(4-(benzyloxy)phenyl)benzohydrazonoyl chloride (2s)

Obtained as a white solid in 15% yield (0.20 g). R_f (petroleum ether/ethyl acetate = 10:1) = 0.53. Mp: 99.5 – 100.5 ℃. ^{1}H NMR (400 MHz, $CDCl_3$) δ 8.02 – 7.86 (m, 3H), 7.57 – 7.33 (m, 8H), 7.16 (d, J = 7.8 Hz, 2H), 7.00 (d, J = 7.9 Hz, 2H), 5.09 (s, 2H). ^{13}C NMR (101 MHz, $CDCl_3$) δ 153.7 (s), 137.7 (s), 137.3 (s), 134.6 (s), 129.0 (s), 128.6 (s), 128.4 (s), 128.0 (s), 127.6 (s), 126.3 (s), 123.9 (s), 116.0 (s), 114.6 (s), 70.7 (s). IR (ATR): ν 3312, 1567, 1447, 1227, 1135, 1105, 822, 735, 681 cm^{-1}. HRMS (ESI) m/z: calcd. for $C_{20}H_{18}ClN_2O$ [M + H]$^+$: 337.1102; found: 337.1083

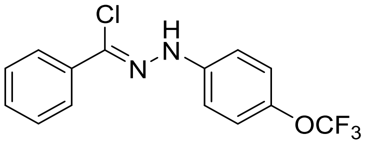

N-(4-(trifluoromethoxy)phenyl)benzohydrazonoyl chloride (2u)

Obtained as a white solid in 76% yield (0.99 g). R_f (petroleum ether/ethyl acetate = 10:1) = 0.82. Mp: 57.8 – 58.3 ℃. ^{1}H NMR (400 MHz, $CDCl_3$) δ 8.09 (s, 1H), 7.96 (d, J = 5.0 Hz, 2H), 7.54 – 7.35 (m, 3H), 7.27 – 7.12 (m, 4H). ^{19}F NMR (376 MHz, $CDCl_3$) δ -58.3 (s, 3F). ^{13}C NMR (101 MHz, $CDCl_3$) δ 143.2 (s), 142.1 (s), 134.2 (s), 129.5 (s), 128.5 (s), 126.5 (s), 125.7 (s), 122.5 (s), 120.7 (q, J = 256.0 Hz), 114.1 (s). IR (ATR): ν 3349, 1606, 1510, 1445, 1215, 1194, 1121, 836, 759, 684 cm^{-1}. HRMS (ESI) m/z: calcd. for $C_{14}H_{11}ClF_3N_2O$ [M + H]$^+$: 315.0507; found: 315.0504.

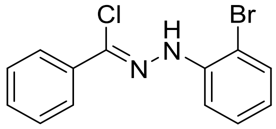

N-(2-bromophenyl)benzohydrazonoyl chloride (2aa)

Obtained as a white solid in 67% yield (0.80 g). R_f (petroleum ether/ethyl acetate = 10:1) = 0.75. Mp: 81.7 – 82.8 ℃. ^{1}H NMR (400 MHz, $CDCl_3$) δ 8.63 (s, 1H), 7.98 (s, 2H), 7.61 (d, J = 7.8 Hz, 1H), 7.52 (d, J = 7.7 Hz, 1H), 7.44 (s, 3H), 7.34 (s, 1H), 6.84 (s, 1H). ^{13}C NMR (101 MHz, $CDCl_3$) δ 140.4 (s), 134.2 (s), 132.5 (s), 129.6 (s), 128.7 (s), 128.5 (s), 127.2 (s), 126.7 (s), 121.7 (s), 114.9 (s), 107.8 (s). IR (ATR): ν 3310, 1581, 1504, 1487, 1444, 1312, 1230, 1141, 1018, 944, 736, 685 cm^{-1}. HRMS (ESI) m/z: calcd. for $C_{13}H_{11}ClBrN_2$ [M + H]$^+$: 308.9789; found: 308.9787.

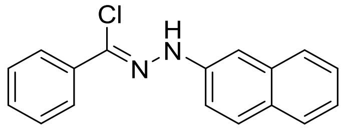

N-(naphthalen-2-yl)benzohydrazonoyl chloride (2ac)

Obtained as a white solid in 53% yield (1.41 g). R_f (petroleum ether/ethyl acetate = 10:1) = 0.58. Mp: 162.5 – 163.3 ℃. ^{1}H NMR (400 MHz, $CDCl_3$) δ 8.25 (s, 1H), 8.05 – 7.97 (m, 2H), 7.87 – 7.76 (m, 3H), 7.57 (d, J = 2.2 Hz, 1H), 7.52 – 7.40 (m, 5H), 7.38 – 7.31 (m, 1H). ^{13}C NMR (101 MHz, $CDCl_3$) δ 141.0 (s), 134.6 (s), 134.4 (s), 129.4 (s, 2C), 129.3 (s), 128.5 (s), 127.8 (s), 126.8 (s), 126.7 (s), 126.5 (s), 125.2 (s), 123.5 (s), 115.6 (s), 108.1 (s). IR (ATR): ν 3299, 1619, 1552, 1471, 1440, 1294, 1213,

1131, 924, 837, 744, 680 cm^{-1}. HRMS (ESI) m/z: calcd. for $C_{17}H_{14}ClN_2$ $[M + H]^+$: 281.0840; found: 281.0838.

General procedure for the synthesis of 5-trifluoromethyl 1,2,4-triazoles

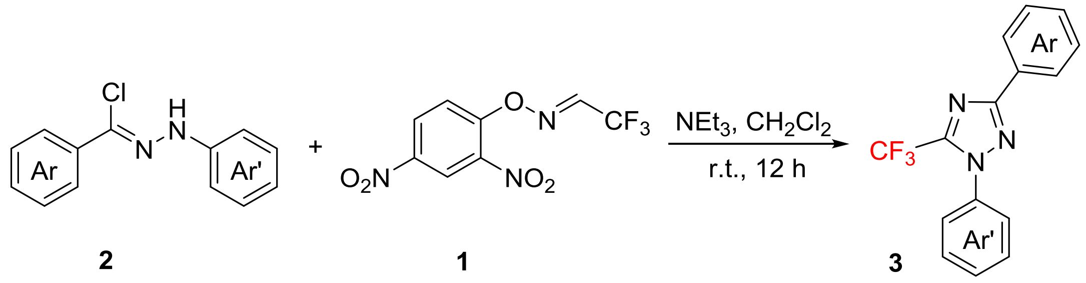

A mixture of hydrazonoyl chloride **2** (0.30 mmol, 1.5 equiv) and trifluoroacetaldehyde *O*-(2,4-dinitrophenyl) oxime **1** (54.1 mg, 0.20 mmol, 1.0 equiv) in CH_2Cl_2 (1.0 mL) was added to a Schlenk tube equipped with a stir bar. Then NEt_3 (60.6 mg, 83.2 μL, 0.60 mmol, 3.0 equiv) was added. The tube was immediately sealed with a Teflon cap and stirred at room temperature for 12 hours. After the reaction was terminated, the solvent was removed in vacuo under reduced pressure. The product was purified by flash column chromatography on silica gel with petroleum ether and CH_2Cl_2 as eluent.

Procedure for the gram-scale synthesis of 3a

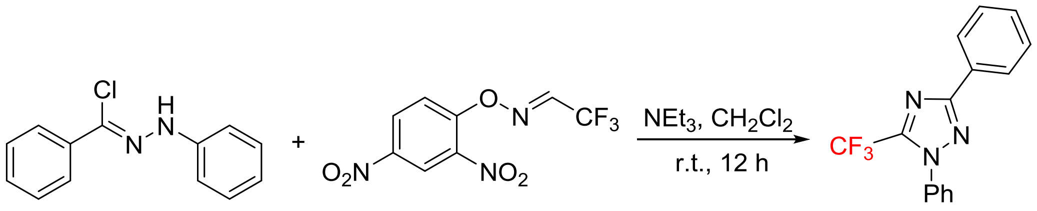

2a 2.92 g | **1** 2.79 g | **3a** 1.63 g, 56%

A 100 mL Schlenk tube was charged with *N*-phenylbenzohydrazonoyl chloride **2a** (2.92 g, 12.9 mmol, 1.3 equiv), *O*-(2,4-dinitrophenyl) oxime **1** (2.79 g, 10.0 mmol, 1.0 equiv) and CH_2Cl_2 (10 mL). The reaction system was cooled with liquid nitrogen. NEt_3 (3.0 g, 4.1 mL, 30 mmol, 3.0 equiv) was added to the reaction system and the reaction tube was sealed with a Teflon cap. The reaction tube was gradually warmed to room temperature and stirred vigorously for 12 hours. After the reaction was terminated, the solvent was removed under vacuum, and the residue was purified by column chromatography (silica gel) with petroleum ether and CH_2Cl_2 as eluent to obtain **3a** in yield of 56% (1.63 g).

Product derivatization

(a)

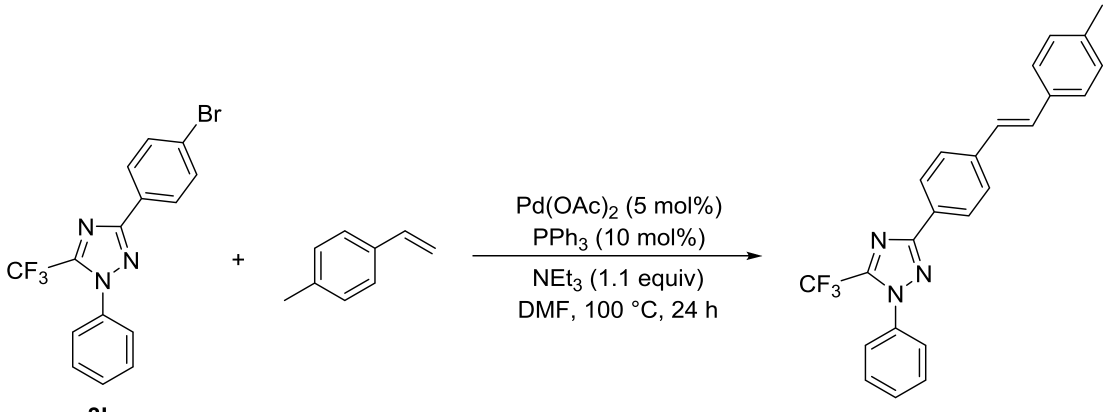

A 10 mL Schlenk tube was charged with 3-(4-bromophenyl)-1-phenyl-5-(trifluoromethyl)-1*H*-1,2,4-triazole **3l** (72.2 mg, 0.20 mmol, 1.0 equiv), 1-methyl-4-vinylbenzene (47.3 mg, 47.9 μL, 0.40 mmol, 2.0 equiv), $Pd(OAc)_2$ (2.2 mg, 0.010 mmol, 0.05 equiv), PPh_3 (5.2 mg, 0.020 mmol, 0.10 equiv), NEt_3 (22.2 mg, 30.5 μL, 0.22 mmol, 1.1 equiv) and DMF (1 mL). The resulting mixture was stirred overnight at 100 ℃ under N_2. After the tube was cooled to room temperature, the mixture was poured into separatory funnel. The aqueous was extracted with ethyl acetate (10 mL × 3). The combined organic layer was washed with brine, dried over anhydrous sodium sulfate, and filtered. The solvent was removed under reduced pressure, and the residue was purified by flash column chromatography on silica gel with petroleum ether and ethyl acetate to give coupling product **6** in 62% yield.

(b)

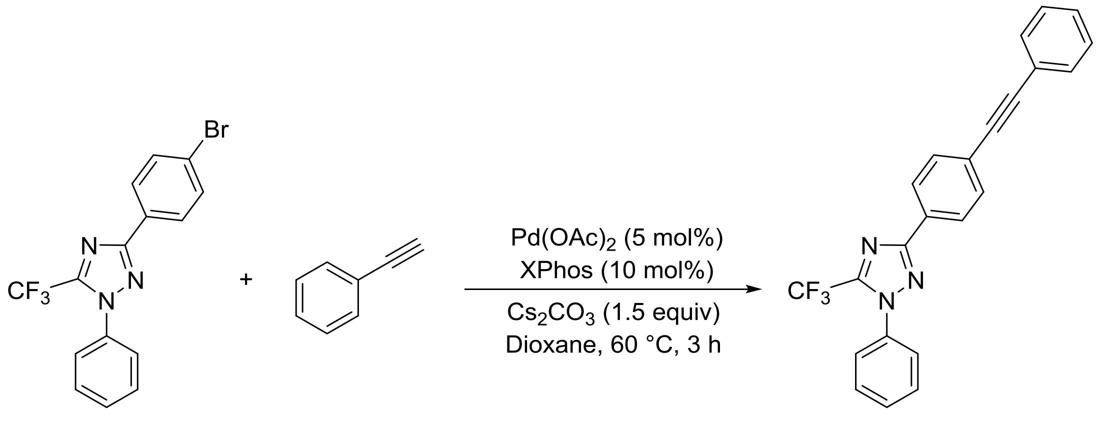

A 10 mL Schlenk tube was charged with 3-(4-bromophenyl)-1-phenyl-5-(trifluoromethyl)-1*H*-1,2,4-triazole **3l** (72.2 mg, 0.20 mmol, 1.0 equiv), phenylacetylene (40.8 mg, 41.8 μL, 0.40 mmol, 2.0 equiv), $Pd(OAc)_2$ (2.2 mg, 0.010 mmol, 0.05 equiv), XPhos (9.5 mg, 0.020 mmol, 0.1 equiv), Cs_2CO_3 (97.7 mg, 0.30 mmol, 1.5 equiv), and 1,4-dioxane (1 mL). The resulting mixture was stirred at 60 ℃ for 3 h under N_2. After the tube was cooled to room temperature, the mixture was poured into separatory funnel. The aqueous was extracted with ethyl acetate (10 mL × 3). The combined organic layer was washed with brine, dried over anhydrous sodium sulfate, and filtered. The solvent was removed under reduced pressure, and the residue was purified by flash column chromatography on silica gel with petroleum ether and ethyl acetate to give coupling product **7** as a yellow solid in 71% yield.

(c)

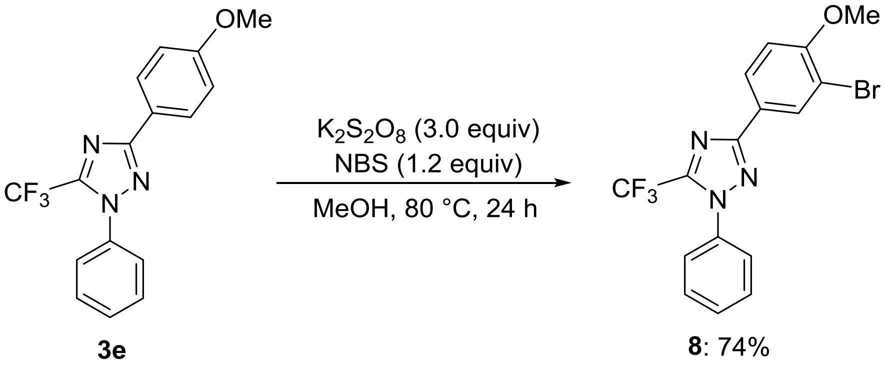

To a 10 mL Schlenk tube was added 3-(4-methoxyphenyl)-1-phenyl-5-(trifluoromethyl)-1*H*-1,2,4-triazole **3e** (63.8 mg, 0.20 mmol, 1.0 equiv), potassium persulfate (162.2 mg, 0.60 mmol, 3.0 equiv), *N*-bromosuccinimide (42.7 mg, 0.24 mmol, 1.2 equiv), and MeOH (1 mL). The reaction tube was sealed with a Teflon cap and the mixture was stirred at 80 °C for 24 h. After the reaction was terminated, sodium sulfite was added to mixture, and the solution was poured into the separatory funnel. Water (10 mL) was added to the mixture. The aqueous was extracted with ethyl acetate (3 × 10 mL). The organic layer was washed with brine, and dried over anhydrous sodium sulfate. The solution was filtered and the filtrate was vacuumed to remove the solvent. The crude product was purified by column chromatography (silica gel) with petroleum ether and ethyl acetate as eluent to obtain **8** in 74% yield.

Data for compounds

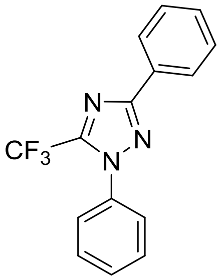

1,3-diphenyl-5-(trifluoromethyl)-1*H*-1,2,4-triazole (3a)

Obtained as a white solid in 70% yield (40.5 mg). R_f (petroleum ether/CH_2Cl_2 = 5:1) = 0.50. Mp: 37.4 – 38.1 ℃. ^{1}H NMR (400 MHz, $CDCl_3$) δ 8.23 – 8.14 (m, 2H), 7.56 (s, 5H), 7.49 – 7.42 (m, 3H). ^{19}F NMR (376 MHz, $CDCl_3$) δ -60.5 (s, 3F). ^{13}C NMR (101 MHz, $CDCl_3$) δ 162.2 (s), 145.1 (q, *J* = 40.5 Hz), 136.7 (s), 130.4 (s), 130.3 (s), 129.6 (s), 129.4 (s), 128.9 (s), 126.8 (s), 125.6 (s), 117.9 (q, *J* = 291.8 Hz). IR (ATR): ν 3304, 3051, 1595, 1527, 1500, 1446, 1309, 1217, 1187, 1122, 1003, 766, 739, 685 cm^{-1}. HRMS (ESI) m/z: calcd. for $C_{15}H_{11}F_3N_3$ $[M + H]^+$: 290.0900; found: 290.0896.

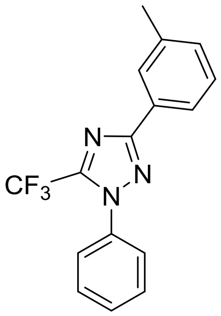

1-phenyl-3-(*m*-tolyl)-5-(trifluoromethyl)-1*H*-1,2,4-triazole (3b)

Obtained as a white solid in 98% yield (59.4 mg). R_f (petroleum ether/CH_2Cl_2 = 5:1) = 0.67. Mp: 70.1 – 71.9 ℃. ^{1}H NMR (400 MHz, $CDCl_3$) δ 8.01 (s, 1H), 7.97 (d, *J* = 7.6 Hz, 1H), 7.54 (s, 5H), 7.35 (t, *J* = 7.6 Hz, 1H), 7.26 (d, *J* = 7.5 Hz, 1H), 2.41 (s, 3H). ^{19}F NMR (376 MHz, $CDCl_3$) δ -60.5 (s, 3F). ^{13}C NMR (101 MHz, $CDCl_3$) δ 162.3 (s), 145.0 (q, *J* = 40.4 Hz), 138.6 (s), 136.7 (s), 131.1 (s), 130.4 (s), 129.6 (s), 129.3 (s), 128.8 (s), 127.4 (s), 125.6 (s), 124.0 (s), 118.2 (q, *J* = 271.8 Hz), 21.5 (s). IR (ATR): ν 3308, 2921, 1594, 1499, 1462, 1309, 1185, 1123, 1084, 1004, 688 cm^{-1}.

HRMS (ESI) m/z: calcd. for $C_{16}H_{13}F_3N_3$ $[M + H]^+$: 304.1056; found: 304.1054.

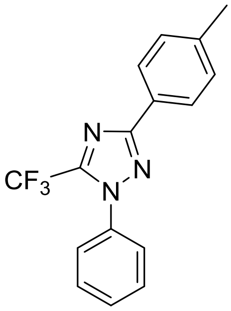

1-phenyl-3-(*p*-tolyl)-5-(trifluoromethyl)-1*H*-1,2,4-triazole (3c)

Obtained as a white solid in 75% yield (45.5 mg). R_f (petroleum ether/CH_2Cl_2 = 5:1) = 0.36. Mp: 118.2 – 119.0 ℃. ^1H NMR (400 MHz, $CDCl_3$) δ 8.07 (d, J = 7.8 Hz, 2H), 7.54 (s, 5H), 7.27 (d, J = 7.8 Hz, 2H), 2.40 (s, 3H). ^{19}F NMR (376 MHz, $CDCl_3$) δ -60.5 (s, 3F). ^{13}C NMR (101 MHz, $CDCl_3$) δ 162.3 (s), 144.9 (q, J = 40.4 Hz), 140.4 (s), 136.8 (s), 130.4 (s), 129.6 (s, 2C), 126.7 (s, 2C), 125.6 (s), 118.2 (q, J = 271.7 Hz), 21.6 (s). IR (ATR): ν 2912, 1588, 1490, 1457, 1308, 1179, 1123, 1005, 744, 680 cm^{-1}. HRMS (ESI) m/z: calcd. for $C_{16}H_{13}F_3N_3$ $[M + H]^+$: 304.1056; found: 304.1055.

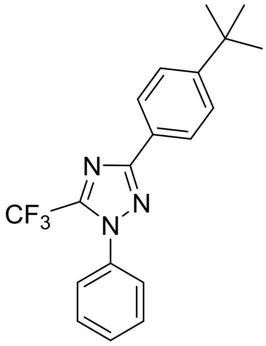

3-(4-(*tert*-butyl)phenyl)-1-phenyl-5-(trifluoromethyl)-1*H*-1,2,4-triazole (3d)

Obtained as a white solid in 60% yield (41.4 mg). R_f (petroleum ether/CH_2Cl_2 = 5:1) = 0.33. Mp: 49.0 – 50.3 ℃. ^1H NMR (400 MHz, $CDCl_3$) δ 8.11 (d, J = 8.0 Hz, 2H), 7.54 (s, 5H), 7.49 (d, J = 8.0 Hz, 2H), 1.36 (s, 9H). ^{19}F NMR (376 MHz, $CDCl_3$) δ -60.4 (s, 3F). ^{13}C NMR (101 MHz, $CDCl_3$) δ 162.2 (s), 153.5 (s), 144.9 (q, J = 40.4 Hz), 136.7 (s), 130.3 (s), 129.5 (s), 126.6 (s), 126.5 (s), 125.8 (s), 125.6 (s), 118.2 (q, J = 271.8 Hz), 34.9 (s), 31.3 (s). IR (ATR): ν 3313, 2948, 1499, 1187, 1025, 997,

688 cm^{-1}. HRMS (ESI) m/z: calcd. for $C_{19}H_{19}F_3N_3$ $[M + H]^+$: 346.1526; found: 346.1524.

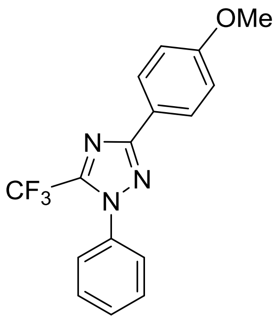

3-(4-methoxyphenyl)-1-phenyl-5-(trifluoromethyl)-1*H*-1,2,4-triazole (3e)

Obtained as yellow solid in 75% yield (36.5 mg). R_f (petroleum ether/CH_2Cl_2 = 5:1) = 0.75. Mp: 105.8 – 106.9 ℃. ^{1}H NMR (400 MHz, $CDCl_3$) δ 8.14 (d, *J* = 8.5 Hz, 2H), 7.57 (s, 5H), 7.01 (d, *J* = 8.5 Hz, 2H), 3.89 (s, 3H). ^{19}F NMR (376 MHz, $CDCl_3$) δ -60.5 (s, 3F). ^{13}C NMR (101 MHz, $CDCl_3$) δ 162.0 (s), 161.2 (s), 144.8 (q, *J* = 40.4 Hz), 136.7 (s), 130.2 (s), 129.4 (s), 128.2 (s), 125.5 (s), 122.0 (s), 118.1 (q, *J* = 270.0 Hz), 114.1 (s), 55.4 (s). IR (ATR): ν 1611, 1493, 1420, 1294, 1246, 1142, 997, 826, 758 cm^{-1}. HRMS (ESI) m/z: calcd. for $C_{16}H_{13}F_3N_3O$ $[M + H]^+$: 320.1005; found: 320.1003.

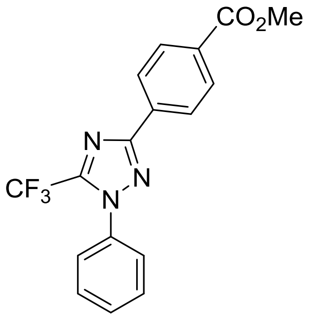

methyl 4-(1-phenyl-5-(trifluoromethyl)-1*H*-1,2,4-triazol-3-yl)benzoate (3f)

Obtained as a white solid in 39% yield (27.1 mg). R_f (petroleum ether/CH_2Cl_2 = 1:1) = 0.37. Mp: 116.3 – 117.8 ℃. ^{1}H NMR (400 MHz, $CDCl_3$) δ 8.26 (d, *J* = 8.0 Hz, 2H), 8.14 (d, *J* = 8.0 Hz, 2H), 7.65 – 7.47 (m, 5H), 3.95 (s, 3H). ^{19}F NMR (376 MHz, $CDCl_3$) δ -60.5 (s, 3F). ^{13}C NMR (101 MHz, $CDCl_3$) δ 166.8 (s), 161.2 (s), 145.4 (q, *J* = 40.7 Hz), 136.6 (s), 133.6 (s), 131.6 (s), 130.6 (s), 130.2 (s), 129.6 (s), 126.7

(s), 125.5 (s), 118.1 (q, J = 271.9 Hz), 52.4 (s). IR (ATR): ν 2942, 1723, 1532, 1499, 1280, 1187, 1123, 991, 767, 739, 685 cm^{-1}. HRMS (ESI) m/z: calcd. for $C_{17}H_{13}F_3N_3O_2$ $[M + H]^+$: 348.0954; found: 348.0952.

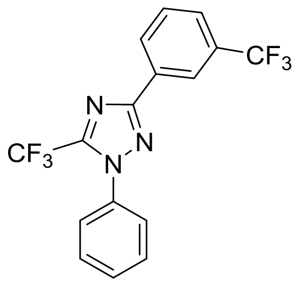

1-phenyl-5-(trifluoromethyl)-3-(3-(trifluoromethyl)phenyl)-1*H*-1,2,4-triazole (3g)

Obtained as a white solid in 34% yield (24.3 mg). R_f (petroleum ether/CH_2Cl_2 = 5:1) = 0.28. Mp: 99.2 – 100.9 ℃. ^1H NMR (400 MHz, $CDCl_3$) δ 8.46 (s, 1H), 8.37 (d, J = 7.8 Hz, 1H), 7.72 (d, J = 7.8 Hz, 1H), 7.64 – 7.50 (m, 6H). ^{19}F NMR (376 MHz, $CDCl_3$) δ -60.6 (s, 3F), -62.7 (s, 3F). ^{13}C NMR (101 MHz, $CDCl_3$) δ 160.9 (s), 145.4 (q, J = 40.8 Hz), 136.5 (s), 131.4 (q, J = 32.7 Hz), 130.7 (s), 130.3 (s), 130.0 (s), 129.7 (s), 129.4 (s), 126.8 (q, J = 3.7 Hz), 125.5 (s), 124.0 (q, J = 272.5 Hz), 123.7 (q, J = 3.8 Hz), 118.1 (q, J = 271.9 Hz). IR (ATR): ν 1521, 1496, 1314, 1131, 1106, 1061, 1005, 893, 812, 685 cm^{-1}. HRMS (ESI) m/z: calcd. for $C_{16}H_{10}F_6N_3$ $[M + H]^+$: 358.0773; found: 358.0772.

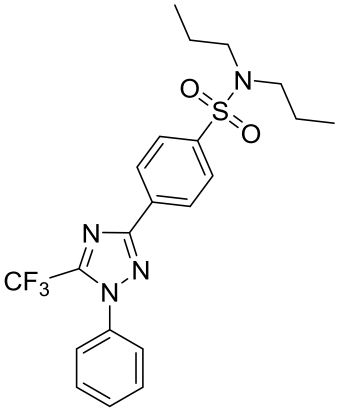

4-(1-phenyl-5-(trifluoromethyl)-1*H*-1,2,4-triazol-3-yl)-*N*,

***N*-dipropylbenzenesulfonamide (3h)**

Obtained as a brown solid in 47% yield (211.2 mg). R_f (petroleum ether/CH_2Cl_2 =10:1) = 0.27. Mp: 85.7 – 86.9 ℃. ^{1}H NMR (400 MHz, $CDCl_3$) δ 8.32 (d, *J* = 8.4 Hz, 2H), 7.92 (d, *J* = 8.4 Hz, 2H), 7.66 – 7.49 (m, 5H), 3.27 – 2.91 (m, 4H), 1.66 – 1.46 (m, 4H), 0.88 (t, *J* = 7.4 Hz, 6H). ^{13}C NMR (101 MHz, $CDCl_3$) δ 160.6 (s), 145.3 (q, *J* = 40.8 Hz), 141.6 (s), 136.4 (s), 133.0 (s), 130.6 (s), 129.6 (s), 127.5 (s), 127.2 (s), 125.4 (s), 117.9 (q, *J* = 271.9 Hz), 49.8 (s), 21.9 (s), 11.2 (s). ^{19}F NMR (376 MHz, $CDCl_3$) δ -60.6 (s, 3F). IR (ATR): ν 2962, 2933, 2873, 1529, 1499, 1465, 1343, 1189, 1137, 1005, 772, 606 cm^{-1}. HRMS (EI) m/z: calcd. for $C_{21}H_{23}F_3N_4SO_2$ $[M]^+$: 452.1488; found: 452.1479.

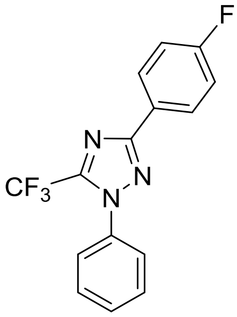

3-(4-fluorophenyl)-1-phenyl-5-(trifluoromethyl)-1*H*-1,2,4-triazole (3i)

Obtained as a white solid in 74% yield (45.4 mg). R_f (petroleum ether/CH_2Cl_2 = 5:1) = 0.55. Mp: 129.9 – 130.4 ℃. ^{1}H NMR (400 MHz, $CDCl_3$) δ 8.21 – 8.09 (m, 2H), 7.67 – 7.46 (m, 5H), 7.15 (t, *J* = 8.5 Hz, 2H). ^{19}F NMR (376 MHz, $CDCl_3$) δ -60.5 (s, 3F), -110.4 – -110.5 (m, 1F). ^{13}C NMR (101 MHz, $CDCl_3$) δ 164.2 (d, *J* = 250.8 Hz), 161.4 (s), 145.1 (q, *J* = 40.6 Hz), 136.7 (s), 130.5 (s), 129.6 (s), 128.8 (d, *J* = 8.6 Hz), 125.7 (d, *J* = 3.2 Hz), 125.6 (s), 118.1 (q, *J* = 271.8 Hz), 116.0 (d, *J* = 22.0 Hz). IR (ATR): ν 1611, 1519, 1456, 1434, 1214, 1184, 1130, 1005, 758, 690 cm^{-1}. HRMS (ESI) m/z: calcd. for $C_{15}H_{10}F_4N_3$ $[M + H]^+$: 308.0805; found: 308.0802.

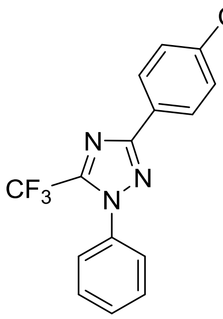

3-(4-chlorophenyl)-1-phenyl-5-(trifluoromethyl)-1*H*-1,2,4-triazole (3j)

Obtained as a white solid in 56% yield (36.3 mg). R_f (petroleum ether/CH_2Cl_2 = 5:1) = 0.50. Mp: 115.2 – 116.5 ℃. ^1H NMR (400 MHz, $CDCl_3$) δ 8.11 (d, J = 8.4 Hz, 2H), 7.63 – 7.47 (m, 5H), 7.43 (d, J = 8.4 Hz, 2H). ^{19}F NMR (376 MHz, $CDCl_3$) δ -60.5 (s, 3F). ^{13}C NMR (101 MHz, $CDCl_3$) δ 161.3 (s), 145.2 (q, J = 40.6 Hz), 136.6 (s), 136.3 (s), 130.6 (s), 129.6 (s), 129.2 (s), 128.1 (s), 128.0 (s), 125.5 (s), 118.1 (q, J = 271.8 Hz). IR (ATR): ν 3309, 2922, 1584, 1498, 1454, 1303, 1138, 769 cm^{-1}. HRMS (ESI) m/z: calcd. for $C_{15}H_{10}ClF_3N_3$ $[M + H]^+$: 324.0510; found: 324.0509.

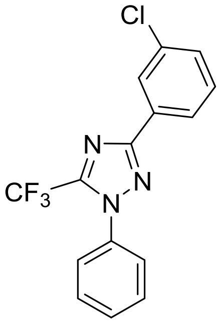

3-(3-chlorophenyl)-1-phenyl-5-(trifluoromethyl)-1*H*-1,2,4-triazole (3k)

Obtained as a yellow solid in 59% yield (38.2 mg). R_f (petroleum ether/CH_2Cl_2 = 5:1) = 0.33. Mp: 108.8 – 109.9 ℃. ^1H NMR (400 MHz, $CDCl_3$) δ 8.19 (s, 1H), 8.06 (d, J = 7.0 Hz, 1H), 7.62 – 7.48 (m, 5H), 7.47 – 7.34 (m, 2H). ^{19}F NMR (376 MHz, $CDCl_3$) δ -60.5 (s, 3F). ^{13}C NMR (101 MHz, $CDCl_3$) δ 161.0 (s), 145.2 (q, J = 40.6 Hz), 136.6 (s), 135.0 (s), 131.2 (s), 130.6 (s), 130.3 (s), 130.2 (s), 129.6 (s), 126.9 (s), 125.5 (s), 124.9 (s), 118.1 (q, J = 271.9 Hz). IR (ATR): ν 1591, 1521, 1437, 1302, 1182, 1120, 1000, 744, 680 cm^{-1}. HRMS (ESI) m/z: calcd. for $C_{15}H_{10}ClF_3N_3$ $[M + H]^+$: 324.0510; found: 324.0509.

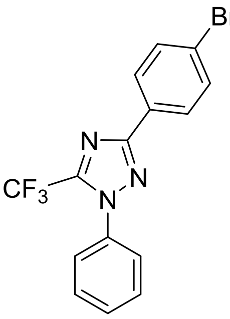

3-(4-bromophenyl)-1-phenyl-5-(trifluoromethyl)-1*H*-1,2,4-triazole (3l)

Obtained as an orange solid in 45% yield (32.9 mg). R_f (CH_2Cl_2/petroleum ether = 1:10) = 0.85. Mp: 103.3 – 104.2 ℃. ^1H NMR (400 MHz, $CDCl_3$) δ 8.08 (d, J = 8.2 Hz, 2H), 7.63 (d, J = 8.3 Hz, 2H), 7.61 – 7.50 (m, 5H). ^{19}F NMR (376 MHz, $CDCl_3$) δ -60.5 (s, 3F). ^{13}C NMR (101 MHz, $CDCl_3$) δ 161.2 (s), 145.1 (q, J = 40.3 Hz), 136.5 (s), 132.0 (s), 130.5 (s), 129.5 (s), 128.3 (s), 128.2 (s), 125.4 (s), 124.6 (s), 117.9 (q, J = 280.7 Hz). IR (ATR): ν 1608, 1485, 1445, 1294, 1218, 1187, 1123, 997, 739, 677 cm^{-1}. HRMS (ESI) m/z: calcd. for $C_{15}H_{10}BrF_3N_3$ $[M + H]^+$: 368.0005; found: 368.0007.

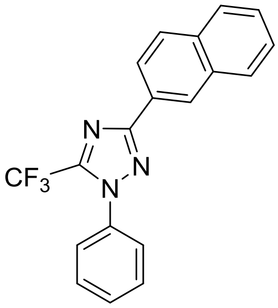

3-(naphthalen-2-yl)-1-phenyl-5-(trifluoromethyl)-1*H*-1,2,4-triazole (3m)

Obtained as a white solid in 75% yield (50.9 mg). R_f (petroleum ether/CH_2Cl_2 = 5:1) = 0.37. Mp: 103.1 – 104.5 ℃. ^1H NMR (400 MHz, $CDCl_3$) δ 8.72 (s, 1H), 8.37 – 8.18 (m, 1H), 7.99 – 7.89 (m, 2H), 7.89 – 7.82 (m, 1H), 7.64 – 7.45 (m, 7H). ^{19}F NMR (376 MHz, $CDCl_3$) δ -60.4 (s, 3F). ^{13}C NMR (101 MHz, $CDCl_3$) δ 162.2 (s), 145.2 (q, J = 40.5 Hz), 136.7 (s), 134.3 (s), 133.3 (s), 130.5 (s), 129.6 (s), 128.8 (s), 128.7 (s), 127.9 (s), 127.1 (s), 126.8 (s), 126.7 (s), 126.6 (s), 125.6 (s), 123.9 (s), 118.2 (q, J = 271.8 Hz). IR (ATR): ν 2923, 1594, 1505, 1462, 1320, 1292, 1269, 1187, 1129, 765, 689 cm^{-1}. HRMS (ESI) m/z: calcd. for $C_{19}H_{13}F_3N_3$ $[M + H]^+$: 340.1056;

found: 340.1055.

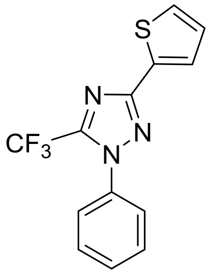

1-phenyl-3-(thiophen-2-yl)-5-(trifluoromethyl)-1*H*-1,2,4-triazole (3n)

Obtained as a white solid in 55% yield (32.5 mg). R_f (petroleum ether/CH_2Cl_2 = 5:1) = 0.40. Mp: 102.4 – 103.8 °C. ^{1}H NMR (400 MHz, $CDCl_3$) δ 7.81 (s, 1H), 7.54 (s, 5H), 7.41 (d, *J* = 3.8 Hz, 1H), 7.13 (s, 1H). ^{19}F NMR (376 MHz, $CDCl_3$) δ -60.5 (s, 3F). ^{13}C NMR (101 MHz, $CDCl_3$) δ 158.4 (s), 144.9 (q, *J* = 40.6 Hz), 136.4 (s), 131.9 (s), 130.5 (s), 129.6 (s), 128.0 (s), 127.8 (s), 125.6 (s), 118.0 (q, *J* = 272.0 Hz). IR (ATR): ν 3105, 3091, 2928, 2850, 1563, 1524, 1493, 1471, 1300, 1210, 1100, 840, 770, 685 cm^{-1}. HRMS (ESI) m/z: calcd. for $C_{13}H_9F_3N_3S$ $[M + H]^+$: 296.0464; found: 296.0467.

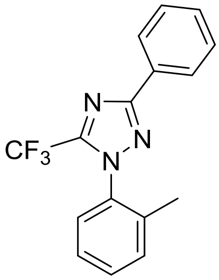

3-phenyl-1-(*o*-tolyl)-5-(trifluoromethyl)-1*H*-1,2,4-triazole (3o)

Obtained as colorless oil in 56% yield (33.9 mg). R_f (petroleum ether/CH_2Cl_2 = 5:1) = 0.55. ^{1}H NMR (400 MHz, $CDCl_3$) δ 8.27 – 8.11 (m, 2H), 7.55 – 7.45 (m, 4H), 7.42 (d, *J* = 7.8 Hz, 1H), 7.40 – 7.33 (m, 2H), 2.17 (s, 3H). ^{19}F NMR (376 MHz, $CDCl_3$) δ -61.9 (s, 3F). ^{13}C NMR (101 MHz, $CDCl_3$) δ 162.3 (s), 145.9 (q, *J* = 40.3 Hz), 135.8 (s), 135.6 (s), 131.4 (s), 131.1 (s), 130.3 (s), 129.6 (s), 128.9 (s), 127.4 (s), 126.9 (s), 126.8 (s), 118.1 (q, *J* = 271.8 Hz), 17.1 (s). IR (ATR): ν 2934, 1499, 1468, 1305, 1182, 1137, 994, 761, 711, 688 cm^{-1}. HRMS (ESI) m/z: calcd. for $C_{16}H_{13}F_3N_3$ $[M + H]^+$:

304.1056; found: 304.1054.

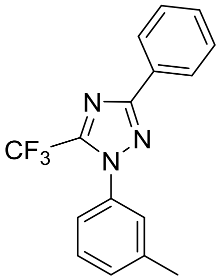

3-phenyl-1-(*m*-tolyl)-5-(trifluoromethyl)-1*H*-1,2,4-triazole (3p)

Obtained as a white solid in 65% yield (39.4 mg). R_f (petroleum ether/CH_2Cl_2 = 5:1) = 0.29. Mp: 66.1 – 67.5 °C. ^1H NMR (400 MHz, $CDCl_3$) δ 8.24 – 8.10 (m, 2H), 7.53 – 7.28 (m, 7H), 2.44 (s, 3H). ^{19}F NMR (376 MHz, $CDCl_3$) δ -60.5 (s, 3F). ^{13}C NMR (101 MHz, $CDCl_3$) δ 162.1 (s), 145.0 (q, *J* = 40.4 Hz), 139.9 (s), 136.6 (s), 131.2 (s), 130.2 (s), 129.5 (s), 129.3 (s), 128.8 (s), 126.8 (s), 126.1 (s), 122.6 (s), 118.2 (q, *J* = 271.7 Hz), 21.3 (s). IR (ATR): ν 2921, 1602, 1524, 1487, 1305, 1190, 1128, 1025, 795, 688 cm^{-1}. HRMS (ESI) m/z: calcd. for $C_{16}H_{13}F_3N_3$ $[M + H]^+$: 304.1056; found: 304.1055.

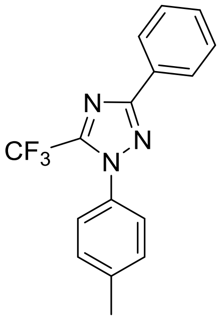

3-phenyl-1-(*p*-tolyl)-5-(trifluoromethyl)-1*H*-1,2,4-triazole (3q)

Obtained as a white solid in 59% yield (35.7 mg). R_f (petroleum ether/CH_2Cl_2 = 5:1) = 0.53. Mp: 77.6 – 78.9 °C. ^1H NMR (400 MHz, $CDCl_3$) δ 8.24 – 8.12 (m, 2H), 7.53 – 7.44 (m, 3H), 7.41 (d, *J* = 8.1 Hz, 2H), 7.34 (d, *J* = 8.0 Hz, 2H), 2.45 (s, 3H). ^{19}F NMR (376 MHz, $CDCl_3$) δ -60.6 (s, 3F). ^{13}C NMR (101 MHz, $CDCl_3$) δ 162.0 (s), 145.1 (q, *J* = 40.4 Hz), 140.8 (s), 134.3 (s), 130.2 (s), 130.1 (s), 129.6 (s), 128.8 (s), 126.8 (s), 125.4 (s), 118.2 (q, *J* = 271.7 Hz), 21.4 (s). IR (ATR): ν 2931, 1515, 1468,

1305, 1176, 1123, 1002, 817, 739, 711 cm^{-1}. HRMS (ESI) m/z: calcd. for $C_{16}H_{13}F_3N_3$ $[M + H]^+$: 304.1056; found: 304.1055.

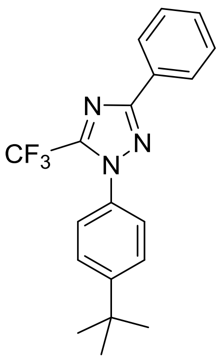

1-(4-(*tert*-butyl)phenyl)-3-phenyl-5-(trifluoromethyl)-1*H*-1,2,4-triazole (3r)

Obtained as a white solid in 60% yield (41.9 mg). R_f (petroleum ether/CH_2Cl_2 = 5:1) = 0.12. Mp: 116.0 – 117.2 ℃. ^{1}H NMR (400 MHz, $CDCl_3$) δ 8.23 – 8.11 (m, 2H), 7.55 (d, J = 7.9 Hz, 2H), 7.52 – 7.40 (m, 5H), 1.38 (s, 9H). ^{19}F NMR (376 MHz, $CDCl_3$) δ -60.5 (s, 3F). ^{13}C NMR (101 MHz, $CDCl_3$) δ 162.0 (s), 153.9 (s), 145.0 (q, J = 40.4 Hz), 134.1 (s), 130.2 (s), 129.6 (s), 128.8 (s), 126.8 (s), 126.5 (s), 125.1 (s), 118.2 (q, J = 271.7 Hz), 35.1 (s), 31.4 (s). IR (ATR): ν 2962, 1515, 1468, 1442, 1308, 1190, 1137, 1067, 1005, 826 cm^{-1}. HRMS (ESI) m/z: calcd. for $C_{19}H_{19}F_3N_3$ $[M + H]^+$: 346.1526; found: 346.1524.

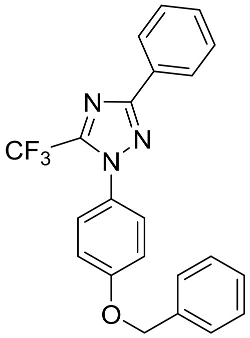

1-(4-(benzyloxy)phenyl)-3-phenyl-5-(trifluoromethyl)-1*H*-1,2,4-triazole (3s)

Obtained as a white solid in 49% yield (38.1 mg). R_f (petroleum ether/CH_2Cl_2 = 1:1) = 0.63. Mp: 122.5 – 123.9 ℃. ^{1}H NMR (400 MHz, $CDCl_3$) δ 8.22 – 8.10 (m, 2H), 7.53 – 7.29 (m, 10H), 7.08 (d, J = 8.7 Hz, 2H), 5.11 (s, 2H). ^{19}F NMR (376 MHz,

$CDCl_3$) δ -60.6 (s, 3F). ^{13}C NMR (101 MHz, $CDCl_3$) δ 161.9 (s), 160.1 (s), 145.1 (q, *J* = 40.2 Hz), 136.2 (s), 130.2 (s), 129.7 (s), 129.5 (s), 128.8 (s), 128.4 (s), 127.6 (s), 127.0 (s), 126.8 (s), 118.2 (q, *J* = 271.7 Hz), 115.5 (s), 70.5 (s). IR (ATR): ν 3301, 2917, 1510, 1440, 1300, 1190, 1016, 680 cm^{-1}. HRMS (ESI) m/z: calcd. for $C_{22}H_{17}F_3N_3O$ [M + H]$^+$: 396.1318; found: 396.1320.

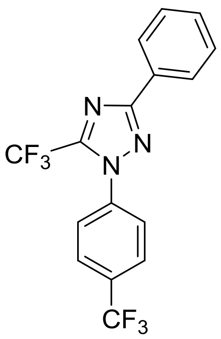

3-phenyl-5-(trifluoromethyl)-1-(4-(trifluoromethyl)phenyl)-1*H*-1,2,4-triazole (3t)

Obtained as a white solid in 46% yield (32.9 mg). R_f (petroleum ether/CH_2Cl_2 = 5:1) = 0.36. Mp: 67.1 – 68.7 ℃. ^1H NMR (400 MHz, $CDCl_3$) δ 8.25 – 8.15 (m, 2H), 7.87 (d, *J* = 8.4 Hz, 2H), 7.74 (d, *J* = 8.4 Hz, 2H), 7.56 – 7.45 (m, 3H). ^{19}F NMR (376 MHz, $CDCl_3$) δ -60.3 (s, 3F), -62.8 (s, 3F). ^{13}C NMR (101 MHz, $CDCl_3$) δ 162.7 (s), 145.1 (q, *J* = 40.9 Hz), 139.5 (s), 132.5 (q, *J* = 33.3 Hz), 130.6 (s), 129.1 (s), 129.0 (s), 127.0 (q, *J* = 3.5 Hz), 126.9 (s), 125.9 (s), 123.5 (q, *J* = 272.4 Hz), 118.1 (q, *J* = 271.8 Hz). IR (ATR): ν 2917, 1616, 1524, 1462, 1305, 1137, 1106, 1064, 840, 677 cm^{-1}. HRMS (ESI) m/z: calcd. for $C_{16}H_{10}F_6N_3$ [M + H]$^+$: 358.0773; found: 358.0772.

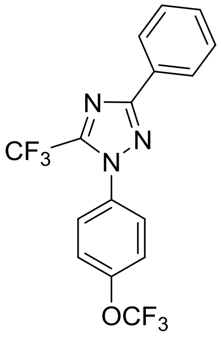

3-phenyl-1-(4-(trifluoromethoxy)phenyl)-5-(trifluoromethyl)-1*H*-1,2,4-triazole (3u)

Obtained as a white solid in 63% yield (46.9 mg). R_f (petroleum ether/CH_2Cl_2 = 5:1) = 0.53. Mp: 71.3 – 72.9 ℃. ^{1}H NMR (400 MHz, $CDCl_3$) δ 8.25 – 8.14 (m, 2H), 7.63 (d, J = 8.8 Hz, 2H), 7.56 – 7.47 (m, 3H), 7.43 (d, J = 8.5 Hz, 2H). ^{19}F NMR (376 MHz, $CDCl_3$) δ -57.9 (s, 3F), -60.5 (s, 3F). ^{13}C NMR (101 MHz, $CDCl_3$) δ 162.5 (s), 150.4 (q, J = 1.7 Hz), 145.2 (q, J = 40.7 Hz), 135.0 (s), 130.5 (s), 129.2 (s), 128.9 (s), 127.3 (s), 126.8 (s), 121.9 (s), 120.5 (q, J = 253.5 Hz), 118.1 (q, J = 271.8 Hz). IR (ATR): ν 1496, 1249, 1165, 1120, 1000, 851, 683 cm^{-1}. HRMS (ESI) m/z: calcd. for $C_{16}H_{10}F_6N_3O$ [M + H]$^+$: 374.0723; found: 374.0723.

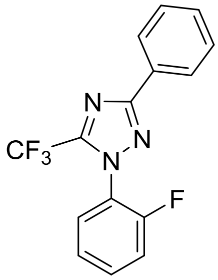

1-(2-fluorophenyl)-3-phenyl-5-(trifluoromethyl)-1*H*-1,2,4-triazole (3v)

Obtained as a reddish brown solid in 48% yield (31.3 mg). R_f (petroleum ether/CH_2Cl_2 = 5:1) = 0.55. Mp: 65.0 – 66.0 ℃. ^{1}H NMR (400 MHz, $CDCl_3$) δ 8.26 – 8.15 (m, 2H), 7.66 – 7.58 (m, 1H), 7.58 – 7.52 (m, 1H), 7.52 – 7.45 (m, 3H), 7.42 – 7.31 (m, 2H). ^{19}F NMR (376 MHz, $CDCl_3$) δ -62.3 (d, J = 4.2 Hz, 3F), -121.1 – -121.4 (m, 1F). ^{13}C NMR (101 MHz, $CDCl_3$) δ 162.9 (s), 157.1 (d, J = 255.1 Hz), 146.5 (q, J = 40.7 Hz), 132.8 (d, J = 7.8 Hz), 130.4 (s), 129.3 (s), 128.9 (s), 128.8 (s), 126.9 (s), 124.9 (d, J = 4.1 Hz), 118.0 (q, J = 272.1 Hz), 117.1 (s), 116.9 (s). IR (ATR): ν 2928, 1597, 1532, 1496, 1442, 1185, 1131, 1075, 944, 817, 764 cm^{-1}. HRMS (ESI) m/z: calcd. for $C_{15}H_{10}F_4N_3$ [M + H]$^+$: 308.0805; found: 308.0804.

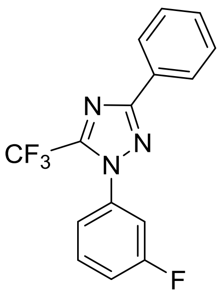

1-(3-fluorophenyl)-3-phenyl-5-(trifluoromethyl)-1*H*-1,2,4-triazole (3w)

Obtained as a white solid in 63% yield (38.7 mg). R_f (petroleum ether/CH_2Cl_2 = 5:1) = 0.45. Mp: 51.2 – 52.7 ℃. ^{1}H NMR (400 MHz, $CDCl_3$) δ 8.27 – 8.12 (m, 2H), 7.63 – 7.45 (m, 4H), 7.45 – 7.25 (m, 3H). ^{19}F NMR (376 MHz, $CDCl_3$) δ -60.5 (s, 3F), -109.8 – -109.8 (m, 1F). ^{13}C NMR (101 MHz, $CDCl_3$) δ 162.7 (d, *J* = 251.0 Hz), 162.4 (s), 145.1 (q, *J* = 40.7 Hz), 137.8 (d, *J* = 10.0 Hz), 131.0 (d, *J* = 8.8 Hz), 130.5 (s), 129.2 (s), 128.9 (s), 126.9 (s), 121.3 (d, *J* = 1.7 Hz), 118.1 (q, *J* = 271.9 Hz), 117.6 (d, *J* = 21.0 Hz), 113.4 (d, *J* = 25.0 Hz). IR (ATR): ν 2906, 1597, 1493, 1440, 1314, 1187, 1126, 1011, 871 cm^{-1}. HRMS (ESI) m/z: calcd. for $C_{15}H_{10}F_4N_3$ $[M + H]^+$: 308.0805; found: 308.0804.

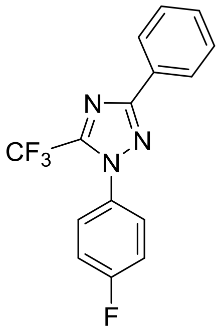

1-(4-fluorophenyl)-3-phenyl-5-(trifluoromethyl)-1*H*-1,2,4-triazole (3x)

Obtained as a white solid in 61% yield (37.5 mg). R_f (petroleum ether/CH_2Cl_2 = 5:1) = 0.35. Mp: 71.1 – 72.3 ℃. ^{1}H NMR (400 MHz, $CDCl_3$) δ 8.22 – 8.01 (m, 2H), 7.62 – 7.38 (m, 5H), 7.23 (t, *J* = 8.3 Hz, 2H). ^{19}F NMR (376 MHz, $CDCl_3$) δ -60.6 (s, 3F), -109.4 – -109.4 (m, 1F). ^{13}C NMR (101 MHz, $CDCl_3$) δ 163.4 (d, *J* = 252.4 Hz), 162.3 (s), 145.2 (q, *J* = 40.6 Hz), 132.7 (d, *J* = 3.3 Hz), 130.4 (s), 129.3 (s), 128.9 (s), 127.7 (d, *J* = 9.1 Hz), 126.8 (s), 118.2 (q, *J* = 270.7 Hz), 116.7 (d, *J* = 23.3 Hz). IR (ATR): ν 3069, 2928, 1507, 1305, 1190, 1117, 988, 831, 742, 714, 677 cm^{-1}. HRMS

(ESI) m/z: calcd. for $C_{15}H_{10}F_4N_3$ [M + H]$^+$: 308.0805; found: 308.0804.

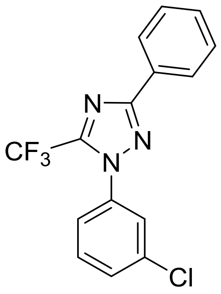

1-(3-chlorophenyl)-3-phenyl-5-(trifluoromethyl)-1*H*-1,2,4-triazole (3y)

Obtained as white solid in 65% yield (41.9 mg). R_f (petroleum ether/CH_2Cl_2 = 5:1) = 0.23. Mp: 88.5 – 89.3 ℃. ^{1}H NMR (400 MHz, $CDCl_3$) δ 8.26 – 8.13 (m, 2H), 7.62 (s, 1H), 7.60 – 7.43 (m, 6H). ^{19}F NMR (376 MHz, $CDCl_3$) δ -60.4 (s, 3F). ^{13}C NMR (101 MHz, $CDCl_3$) δ 162.4 (s), 145.1 (q, *J* = 40.7 Hz), 137.6 (s), 135.4 (s), 130.7 (s), 130.6 (s), 130.5 (s), 129.2 (s), 128.9 (s), 126.9 (s), 126.0 (s), 123.6 (s), 118.1 (q, *J* = 271.9 Hz). IR (ATR): ν 1585, 1485, 1179, 1128, 1011, 868 cm^{-1}. HRMS (ESI) m/z: calcd. for $C_{15}H_{10}ClF_3N_3$ [M + H]$^+$: 324.0510; found: 324.0511.

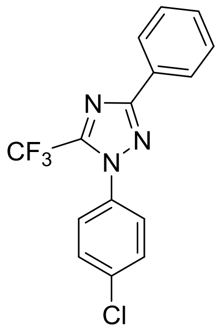

1-(4-chlorophenyl)-3-phenyl-5-(trifluoromethyl)-1*H*-1,2,4-triazole (3z)

Obtained as a white solid in 59% yield (38.2 mg). R_f (petroleum ether/CH_2Cl_2 = 5:1) = 0.35. Mp: 84.8 – 85.9 ℃. ^{1}H NMR (400 MHz, $CDCl_3$) δ 8.21 – 8.10 (m, 2H), 7.56 – 7.38 (m, 7H). ^{19}F NMR (376 MHz, $CDCl_3$) δ -60.4 (s, 3F). ^{13}C NMR (101 MHz, $CDCl_3$) δ 162.4 (s), 145.1 (q, *J* = 40.6 Hz), 136.5 (s), 135.2 (s), 130.4 (s), 129.8 (s), 129.2 (s), 128.9 (s), 126.8 (s, 2C), 118.1 (q, *J* = 271.9 Hz). IR (ATR): ν 1535, 1493, 1440, 1302, 1218, 1176, 1002, 831, 739, 714, 683 cm^{-1}. HRMS (ESI) m/z: calcd. for $C_{15}H_{10}ClF_3N_3$ [M + H]$^+$: 324.0510; found: 324.0509.

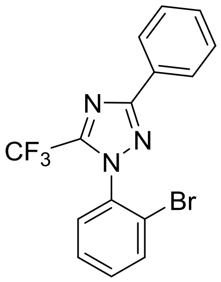

1-(2-bromophenyl)-3-phenyl-5-(trifluoromethyl)-1*H*-1,2,4-triazole (3aa)

Obtained as a reddish brown solid in 40% yield (40.0 mg). R_f (petroleum ether/CH_2Cl_2 = 5:1) = 0.68. Mp: 83.1 – 84.2 ℃. ^{1}H NMR (400 MHz, $CDCl_3$) δ 8.27 – 8.15 (m, 2H), 7.81 (d, *J* = 7.7 Hz, 1H), 7.63 – 7.39 (m, 6H). ^{19}F NMR (376 MHz, $CDCl_3$) δ -62.0 (s, 3F). ^{13}C NMR (101 MHz, $CDCl_3$) δ 162.6 (s), 146.1 (q, *J* = 40.8 Hz), 136.0 (s), 133.9 (s), 132.5 (s), 130.4 (s), 129.3 (s), 129.2 (s), 128.9 (s), 128.4 (s), 126.9 (s), 122.1 (s), 118.0 (q, *J* = 277.5 Hz). IR (ATR): ν 2923, 1515, 1490, 1428, 1305, 1193, 1137, 1050, 988, 711 cm^{-1}. HRMS (ESI) m/z: calcd. for $C_{15}H_{10}BrF_3N_3$ $[M + H]^+$: 368.0005; found: 368.0007.

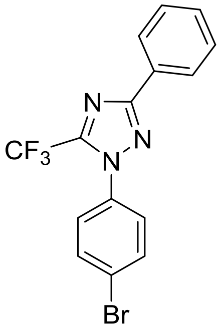

1-(4-bromophenyl)-3-phenyl-5-(trifluoromethyl)-1*H*-1,2,4-triazole (3ab)

Obtained as a white solid in 58% yield (42.6 mg). R_f (petroleum ether/CH_2Cl_2 = 5:1) = 0.50. Mp: 86.4 – 87.5 ℃. ^{1}H NMR (400 MHz, $CDCl_3$) δ 8.22 – 8.08 (m, 2H), 7.68 (d, *J* = 8.4 Hz, 2H), 7.53 – 7.44 (m, 3H), 7.42 (d, *J* = 8.4 Hz, 2H). ^{19}F NMR (376 MHz, $CDCl_3$) δ -60.4 (s, 3F). ^{13}C NMR (101 MHz, $CDCl_3$) δ 162.4 (s), 145.0 (q, *J* = 40.7 Hz), 135.7 (s), 132.8 (s), 130.4 (s), 129.2 (s), 128.9 (s), 127.0 (s), 126.8 (s), 124.6 (s), 118.3 (q, *J* = 260.3 Hz). IR (ATR): ν 1518, 1485, 1305, 1215, 1179, 1117, 1067, 991, 820, 683 cm^{-1}. HRMS (ESI) m/z: calcd. for $C_{15}H_{10}BrF_3N_3$ $[M + H]^+$:

368.0005; found: 368.0006.

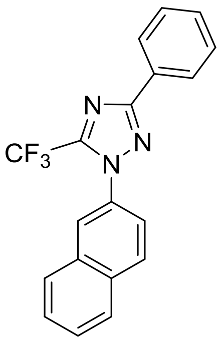

1-(naphthalen-2-yl)-3-phenyl-5-(trifluoromethyl)-1*H*-1,2,4-triazole (3ac)

Obtained as a white solid in 67% yield (45.4 mg). R_f (petroleum ether/CH_2Cl_2 = 5:1) = 0.25. Mp: 98.0 – 99.6 ℃. ^{1}H NMR (400 MHz, $CDCl_3$) δ 8.33 – 8.21 (m, 2H), 8.13 – 8.02 (m, 2H), 8.02 – 7.92 (m, 2H), 7.73 – 7.60 (m, 3H), 7.59 – 7.46 (m, 3H). ^{19}F NMR (376 MHz, $CDCl_3$) δ -60.4 (s, 3F). ^{13}C NMR (101 MHz, $CDCl_3$) δ 162.3 (s), 145.3 (q, J = 40.4 Hz), 134.0 (s), 133.7 (s), 132.9 (s), 130.3 (s), 129.8 (s), 129.6 (s), 128.9 (s), 128.6 (s), 128.1 (s), 127.9 (s), 127.6 (s), 126.9 (s), 124.7 (s), 122.8 (s), 118.3 (q, J = 271.8 Hz). IR (ATR): ν 1597, 1513, 1479, 1437, 1193, 1128, 1072, 1028, 851 cm^{-1}. HRMS (ESI) m/z: calcd. for $C_{19}H_{13}F_3N_3$ [M + H]$^+$: 340.1056; found: 340.1055.

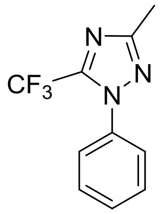

3-methyl-1-phenyl-5-(trifluoromethyl)-1*H*-1,2,4-triazole (5a)

Obtained as an yellow oil in 22% yield (9.9 mg). R_f (petroleum ether) = 0.90. ^{1}H NMR (400 MHz, $CDCl_3$) δ 7.75 – 7.20 (m, 5H), 2.50 (s, 3H). ^{19}F NMR (376 MHz, $CDCl_3$) δ -60.6 (s, 3F). ^{13}C NMR (101 MHz, $CDCl_3$) δ 161.0 (s), 144.3 (q, J = 40.3 Hz), 136.5 (s), 130.1 (s), 129.4 (s), 125.3 (s), 118.0 (q, J = 271.5 Hz), 13.6 (s). IR (ATR): ν 1734, 1513, 1300, 1182, 1128, 1095, 1002, 685 cm^{-1}. HRMS (ESI) m/z:

calcd. for $C_{10}H_9F_3N_3$ [M + H]$^+$: 228.0743; found: 228.0742.

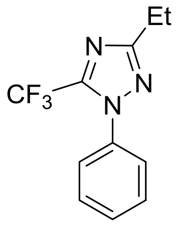

3-ethyl-1-phenyl-5-(trifluoromethyl)-1*H*-1,2,4-triazole (5b)

Obtained as a reddish-brown oil in 23% yield (17.6 mg). R_f (petroleum ether) = 0.92. ^{1}H NMR (400 MHz, $CDCl_3$) δ 7.58 – 7.52 (m, 3H), 7.51 – 7.44 (m, 2H), 2.88 (q, J = 7.6 Hz, 2H), 1.41 (t, J = 7.6 Hz, 3H). ^{19}F NMR (376 MHz, $CDCl_3$) δ -60.5 (s, 3F). ^{13}C NMR (101 MHz, $CDCl_3$) δ 165.7 (s), 144.1 (q, J = 40.3 Hz), 136.6 (s), 130.1 (s), 129.4 (s), 125.4 (s), 118.1 (q, J = 271.6 Hz), 21.6 (s), 12.3 (s). IR (ATR): ν 3341, 2898, 1413, 1135, 1089, 1018, 990, 851 cm^{-1}. HRMS (ESI) m/z: calcd. for $C_{11}H_{11}F_3N_3$ [M + H]$^+$: 242.0900; found: 242.0909.

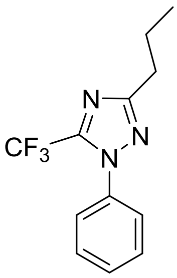

1-phenyl-3-propyl-5-(trifluoromethyl)-1*H*-1,2,4-triazole (5c)

Obtained as a reddish-brown liquid in 40% yield (20.4 mg). R_f (petroleum ether) = 0.78. ^{1}H NMR (400 MHz, $CDCl_3$) δ 7.56 – 7.49 (m, 3H), 7.52 – 7.44 (m, 2H), 2.81 (t, J = 7.5 Hz, 2H), 1.98 – 1.77 (m, 2H), 1.03 (t, J = 7.4 Hz, 3H). ^{19}F NMR (376 MHz, $CDCl_3$) δ -60.5 (s, 3F). ^{13}C NMR (101 MHz, $CDCl_3$) δ 164.7 (s), 144.2 (q, J = 40.3 Hz), 136.6 (s), 130.1 (s), 129.4 (s), 125.3, 118.1 (q, J = 271.6 Hz), 30.0 (s), 21.5 (s), 13.8 (s). IR (ATR): ν 3321, 2887, 1507, 1413, 1300, 1143, 1027, 993, 868, 681 cm^{-1}. HRMS (ESI) m/z: calcd. for $C_{12}H_{13}F_3N_3$ [M + H]$^+$: 256.1056; found: 256.1055.

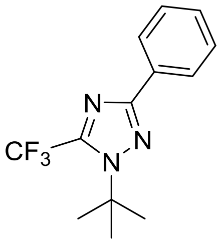

1-(*tert*-butyl)-3-phenyl-5-(trifluoromethyl)-1*H*-1,2,4-triazole (5e)

Obtained as a pale yellow oil in 26% yield (135.0 mg) on a 1.0 mmol scale. R_f (petroleum ether/CH_2Cl_2 =10:1) = 0.25. ^1H NMR (400 MHz, $CDCl_3$) δ 8.14 (d, *J* = 6.5 Hz, 2H), 7.52 – 7.38 (m, 3H), 1.77 (s, 9H). ^{13}C NMR (101 MHz, $CDCl_3$) δ 158.9 (s), 143.9 (q, *J* = 40.1 Hz), 130.0 (s), 129.6 (s), 128.6 (s), 126.4 (s), 118.6 (q, *J* = 270.6 Hz), 63.7 (s), 29.9 (q, *J* = 2.1 Hz). ^{19}F NMR (376 MHz, $CDCl_3$) δ -58.0 (s, 3F). IR (ATR): ν 3330, 3097, 1651, 1509, 1373, 1264, 1191, 812, 692 cm^{-1}. HRMS (EI) m/z: calcd. for $C_{13}H_{14}F_3N_3$ $[M]^+$: 269.1134; found: 269.1137.

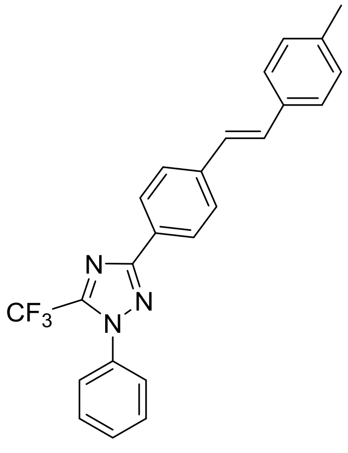

3-(4-(4-methylstyryl)phenyl)-1-phenyl-5-(trifluoromethyl)-1*H*-1,2,4-triazole (6)

R_f (petroleum ether/ethyl acetate = 10:1) = 0.56. Mp: 114.6 – 115.8 ℃. ^1H NMR (400 MHz, $CDCl_3$) δ 8.19 (d, *J* = 8.2 Hz, 2H), 7.63 (d, *J* = 8.2 Hz, 2H), 7.59 (s, 5H), 7.46 (d, *J* = 7.8 Hz, 2H), 7.26 – 7.12 (m, 4H), 2.39 (s, 3H). ^{19}F NMR (376 MHz, $CDCl_3$) δ -60.5 (s, 3F). ^{13}C NMR (101 MHz, $CDCl_3$) δ 161.9 (s), 144.9 (q, *J* = 40.5 Hz), 139.4 (s), 137.9 (s), 136.6 (s), 134.3 (s), 130.4 (s), 129.8 (s), 129.5 (s), 127.0 (s, 2C), 126.7 (s), 126.6 (s), 125.5 (s), 118.1 (q, *J* = 271.9 Hz), 21.3 (s). IR (ATR): ν 2909, 1709, 1605, 1457, 1179, 1128, 1000, 966, 837, 747, 685 cm^{-1}. HRMS (ESI) m/z: calcd. for $C_{24}H_{19}F_3N_3$ $[M + H]^+$: 406.1526; found: 406.1528.

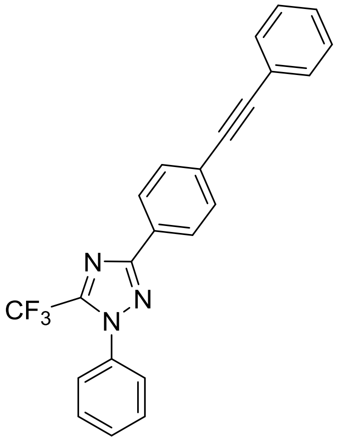

1-phenyl-3-(4-(phenylethynyl)phenyl)-5-(trifluoromethyl)-1*H*-1,2,4-triazole (7)

R_f (petroleum ether/ethyl acetate = 10:1) = 0.50. Mp: 132.1 – 133.8 ℃. ^{1}H NMR (400 MHz, $CDCl_3$) δ 8.21 (d, *J* = 8.3 Hz, 2H), 7.67 (d, *J* = 8.3 Hz, 2H), 7.63 – 7.49 (m, 7H), 7.48 – 7.33 (d, *J* = 1.9 Hz, 3H). ^{19}F NMR (376 MHz, $CDCl_3$) δ -60.5 (s, 3F). ^{13}C NMR (101 MHz, $CDCl_3$) δ 161.5 (s), 145.1 (q, *J* = 40.6 Hz), 136.6 (s), 132.0 (s), 131.7 (s), 130.4 (s), 129.5 (s), 129.0 (s), 128.5 (s), 128.4 (s), 126.6 (s), 125.5 (s), 125.1 (s), 123.0 (s), 118.0 (q, *J* = 271.8 Hz), 91.2 (s), 89.1 (s). IR (ATR): ν 1597, 1493, 1300, 1213, 1128, 997, 834, 750, 674 cm^{-1}. HRMS (ESI) m/z: calcd. for $C_{23}H_{15}F_3N_3$ $[M + H]^+$: 390.1213; found: 390.1214.

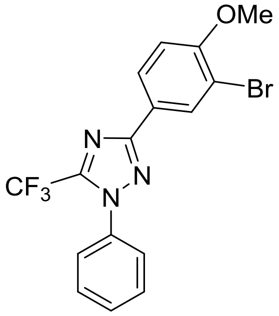

3-(3-bromo-4-methoxyphenyl)-1-phenyl-5-(trifluoromethyl)-1*H*-1,2,4-triazole (8)

R_f (petroleum ether/ethyl acetate = 10:1) = 0.36. Mp: 128.5 – 129.6 ℃. ^{1}H NMR (400 MHz, $CDCl_3$) δ 8.41 (d, *J* = 1.8 Hz, 1H), 8.18 – 8.06 (m, 1H), 7.68 – 7.49 (m, 5H), 7.00 (d, *J* = 8.6 Hz, 1H), 3.98 (s, 3H). ^{19}F NMR (376 MHz, $CDCl_3$) δ -60.5 (s, 3F). ^{13}C NMR (101 MHz, $CDCl_3$) δ 160.7 (s), 157.4 (s), 144.9 (q, *J* = 40.5 Hz), 136.5 (s), 131.7 (s), 130.4 (s), 129.5 (s), 127.1 (s), 125.4 (s), 123.3 (s), 118.0 (q, *J* = 263.8 Hz),

112.1 (s), 111.7 (s), 56.4 (s). IR (ATR): ν 2914, 1605, 1493, 1468, 1423, 1285, 1182, 1120, 997, 742, 677 cm^{-1}. HRMS (ESI) m/z: calcd. for $C_{16}H_{12}BrF_3N_3O$ $[M + H]^+$: 398.0110; found: 398.0115.

Crystal structure analyses

The crystal samples of **3s** were prepared by slow volatilization in a DCM/ petroleum ether (1:1) solvent mixture. The suitable crystals of **3s** (CCDC 2183507) were mounted on quartz fibers and X-ray data collected on a Bruker AXS APEX diffractometer, equipped with a CCD detector at -50 ºC, using MoKα radiation (λ 0.71073 Å). The data was corrected for Lorentz and polarisation effect with the SMART suite of programs and for absorption effects with SADABS.[3] Structure solution and refinement were carried out with the SHELXTL suite of programs. The structure was solved by direct methods to locate the heavy atoms, followed by difference maps for the light non-hydrogen atoms.

Table S1. Crystal data and structure refinement for compounds

Compounds	**3s** (CCDC 2183507)
Empirical formula	$C_{22}H_{16}F_3N_3O$
Formula weight	395.38
Temperature/K	296 K
Wavelength/Å	0.71073
Crystal system	monoclinic
a/Å	8.5293(9)
b/Å	5.3922(5)
c/Å	41.161(5)
α/°	90.00
β/°	93.907(3)
γ/°	90.00
Volume/$Å^3$	1888.7(3)
Z	4
Density (calc.)/cm^3	1.390
Absorption coefficient /mm^{-1}	0.108
F(000)	816.0
Crystal size/mm	0.2×0.2×0.05
Theta range for data collection / °	2.53~24.99
Reflections collected	3304
Independent reflections	1741
Data/restraints/parameters	3304/0/262
Goodness-of-fit on F^2	1.009
Final R indexes [I>=2σ (I)]	0.0538
Final R indexes [all data]	0.1294
Largest diff. peak and hole / e $Å^{-3}$	0.10/-0.20

ORTEP diagrams

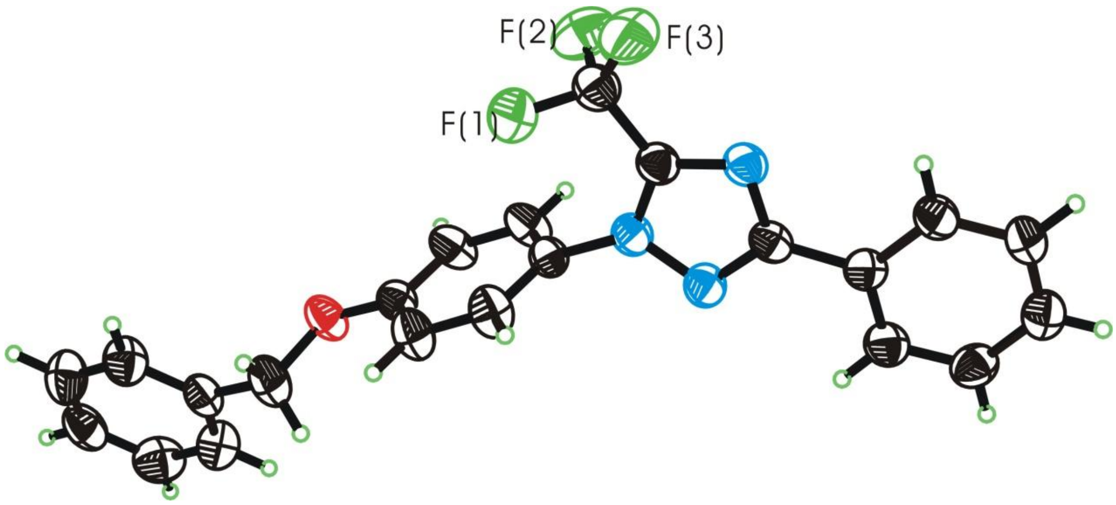

Figure S1. ORTEP diagram of 3s with thermal ellipsoids at the 40% probability level

References

[1] B. Lin, Y. Yao, Y. Huang, Z. Weng, *Org. Lett.* **2022**, *24*, 2055-2058.

[2] a) C. Tirapegui, W. Acevedo-Fuentes, P. Dahech, C. Torrent, P. Barrias, M. Rojas-Poblete, C. Mascayano, *Bioorg. Med. Chem. Lett.* **2017**, *27,* 1649-1653; b) I. Yavari, O. Khaledian, *Chem. Commun.* **2020**, *56*, 9150-9153.

[3] SHELXTL version 5.03; Bruker Analytical X-ray Systems, Madison, WI, 1997.

Copies of ^{1}H NMR, ^{19}F NMR and ^{13}C NMR spectra

^{1}H NMR spectra of S-2s (400 MHz, DMSO-d_6)

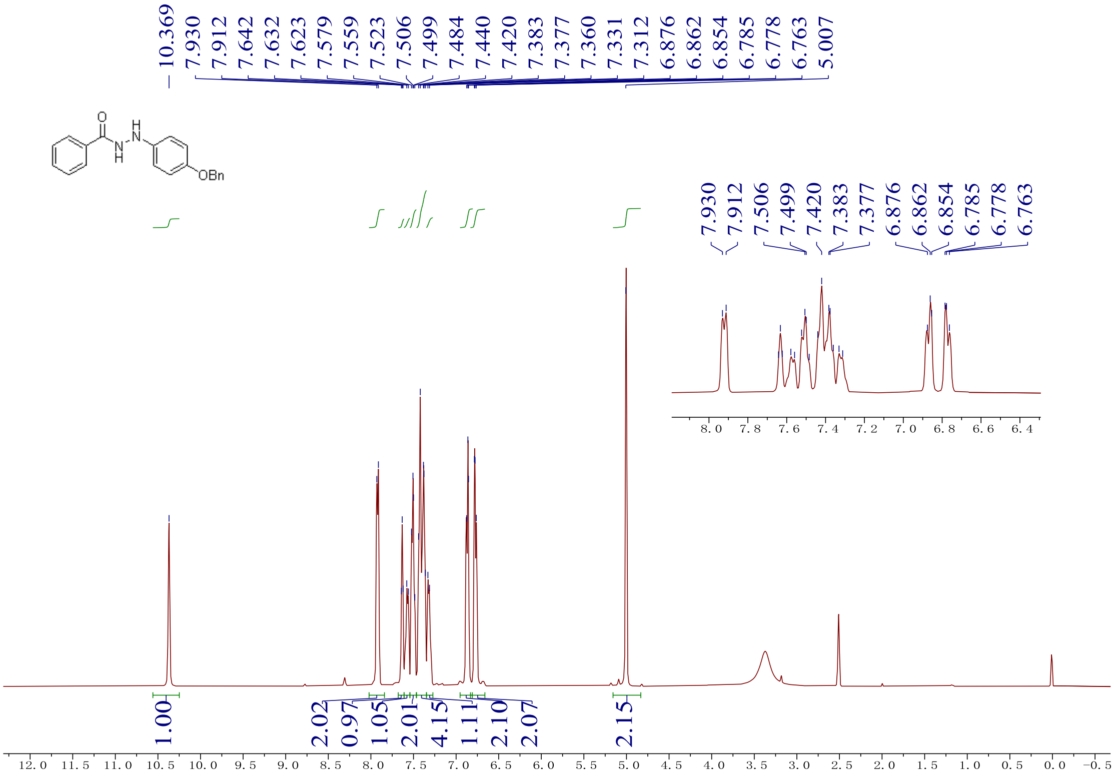

^{13}C{^{1}H} NMR spectra of S-2s (101 MHz, DMSO-d_6)

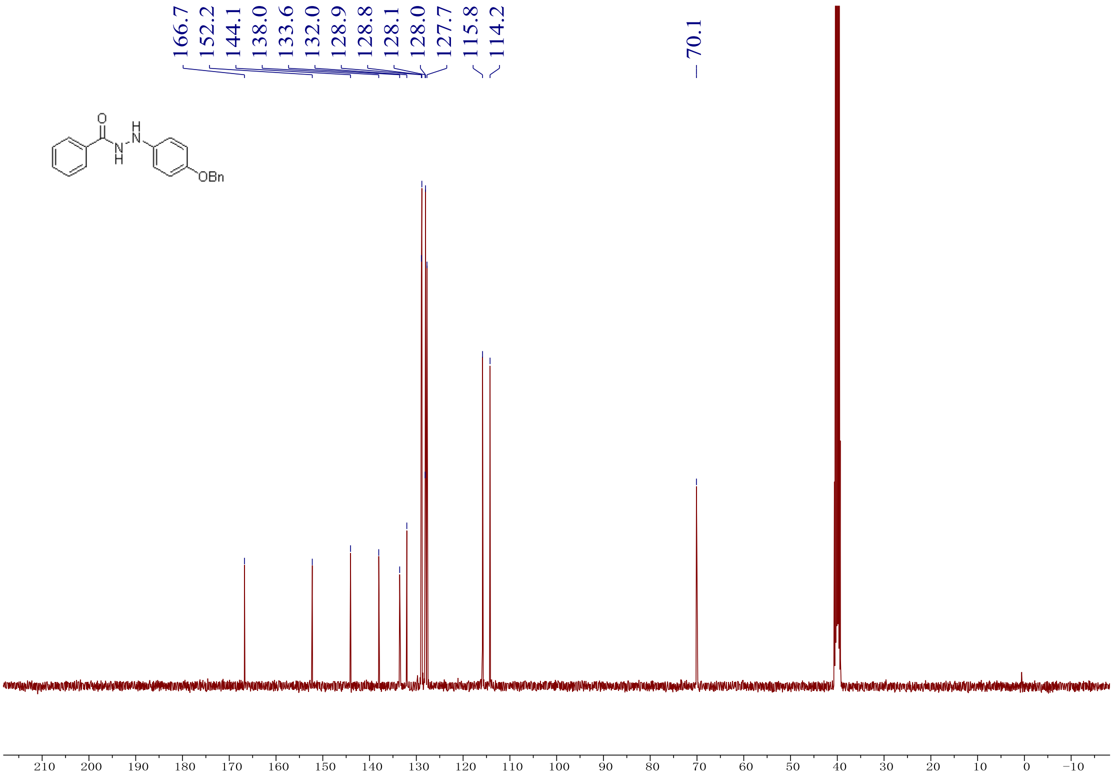

^{1}H NMR spectra of S-2aa (400 MHz, DMSO-d_6)

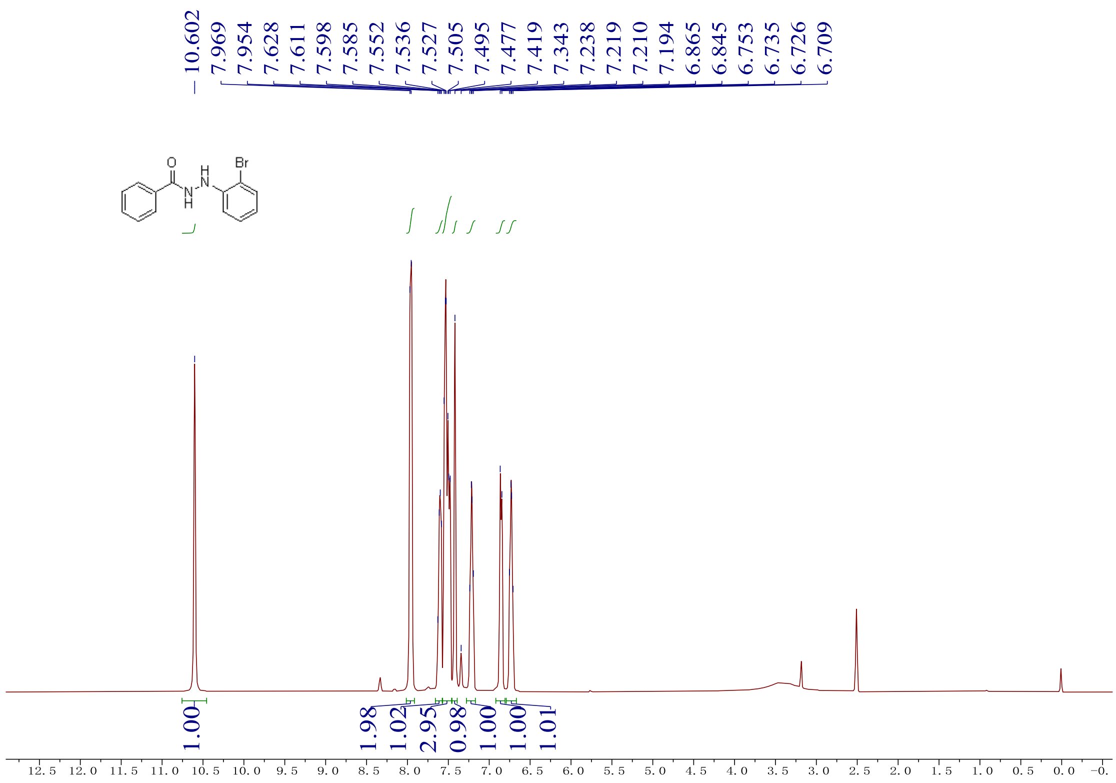

$^{13}C\{^{1}H\}$ NMR spectra of S-2aa (101 MHz, DMSO-d_6)

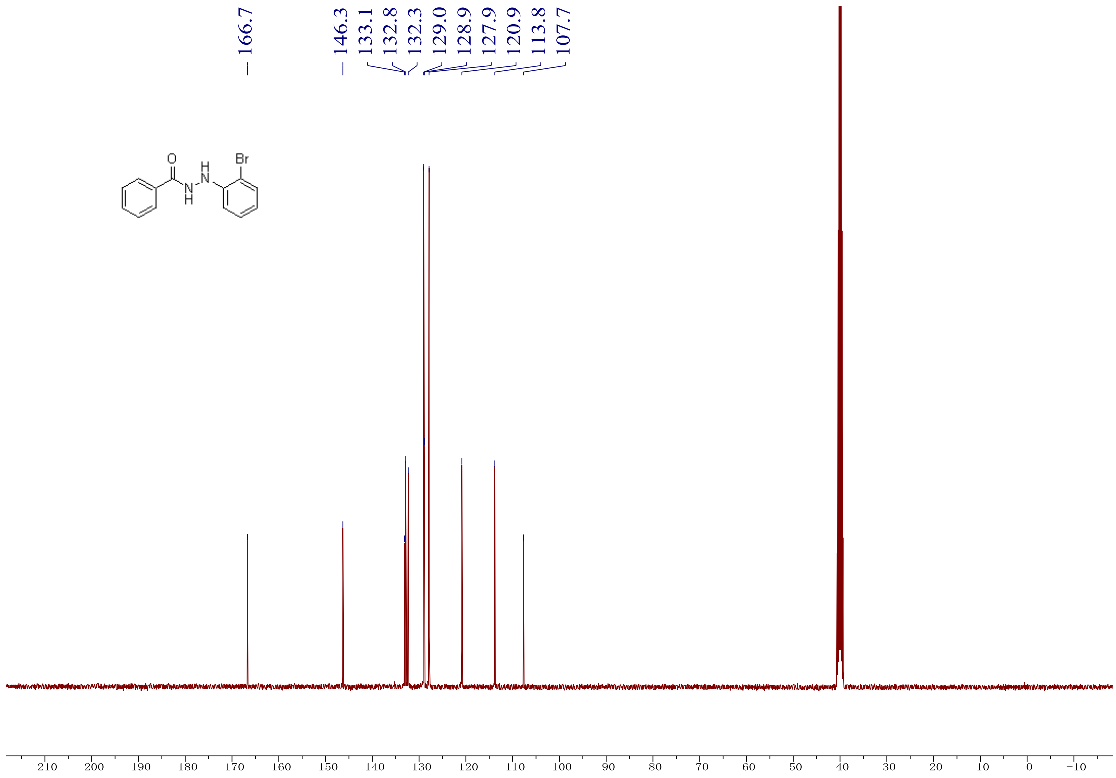

^{1}H NMR spectra of 2r (400 MHz, $CDCl_3$)

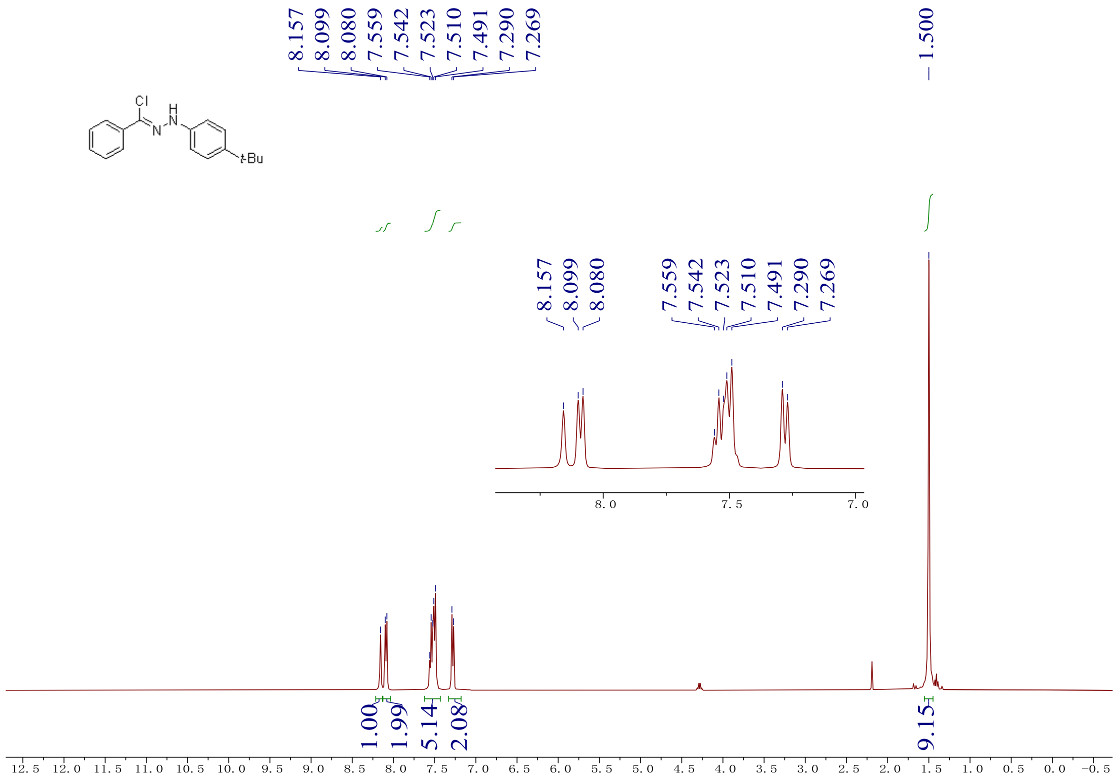

^{13}C{^{1}H} NMR spectra of 2r (101 MHz, $CDCl_3$)

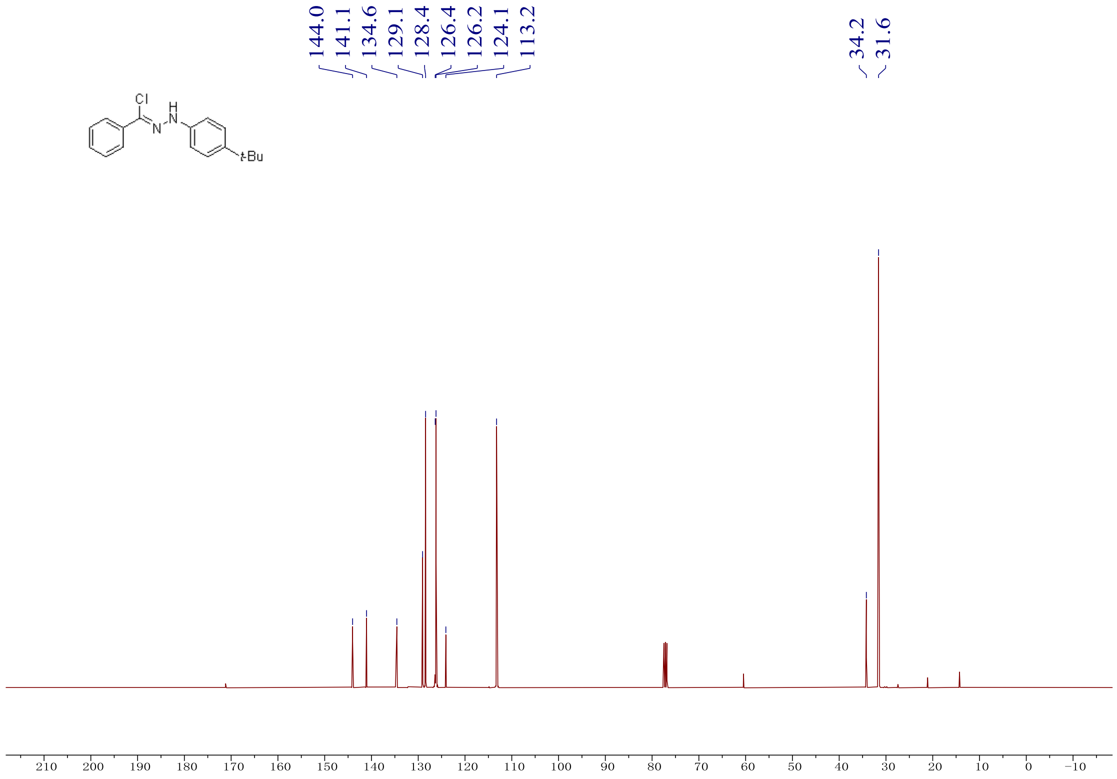

^{1}H NMR spectra of 2s (400 MHz, CDCl$_3$)

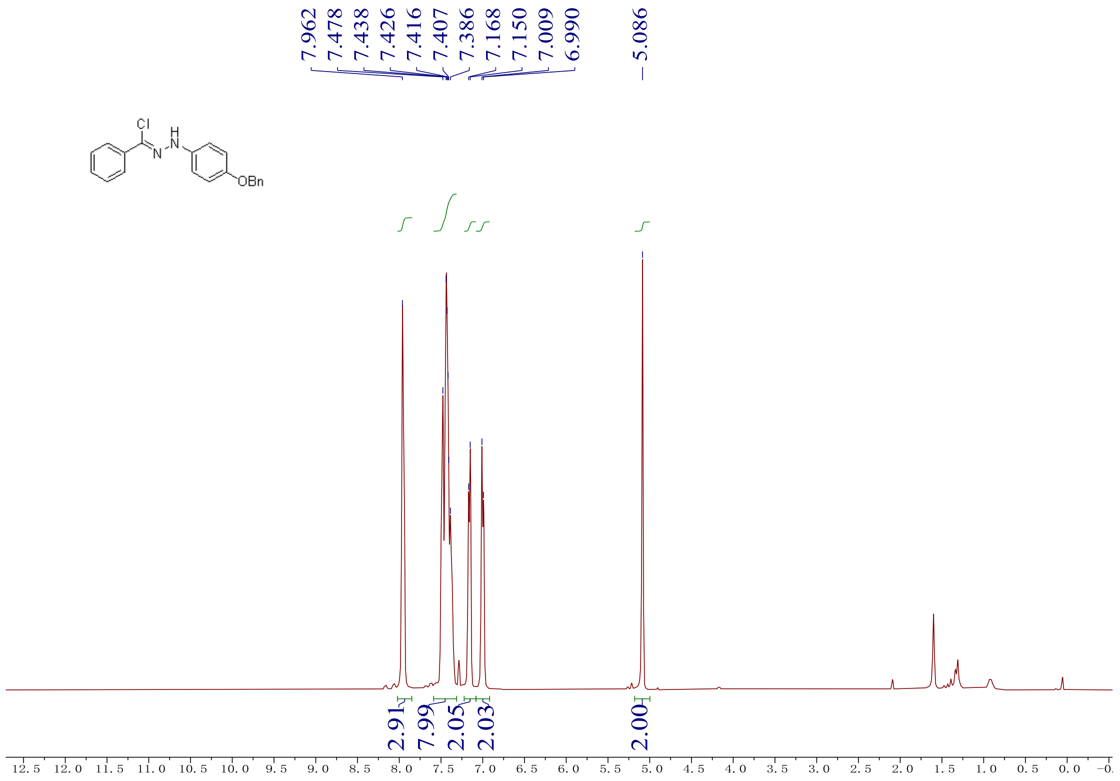

^{13}C{^{1}H} NMR spectra of 2s (101 MHz, CDCl$_3$)

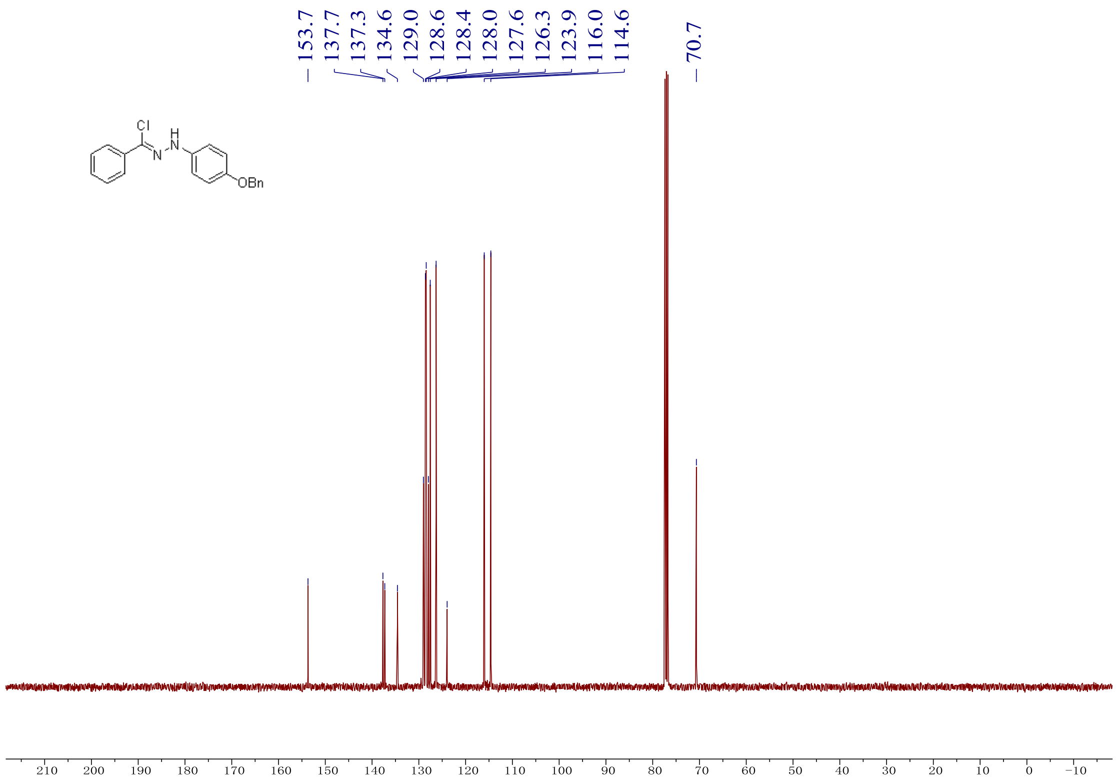

^{1}H NMR spectra of 2u (400 MHz, $CDCl_3$)

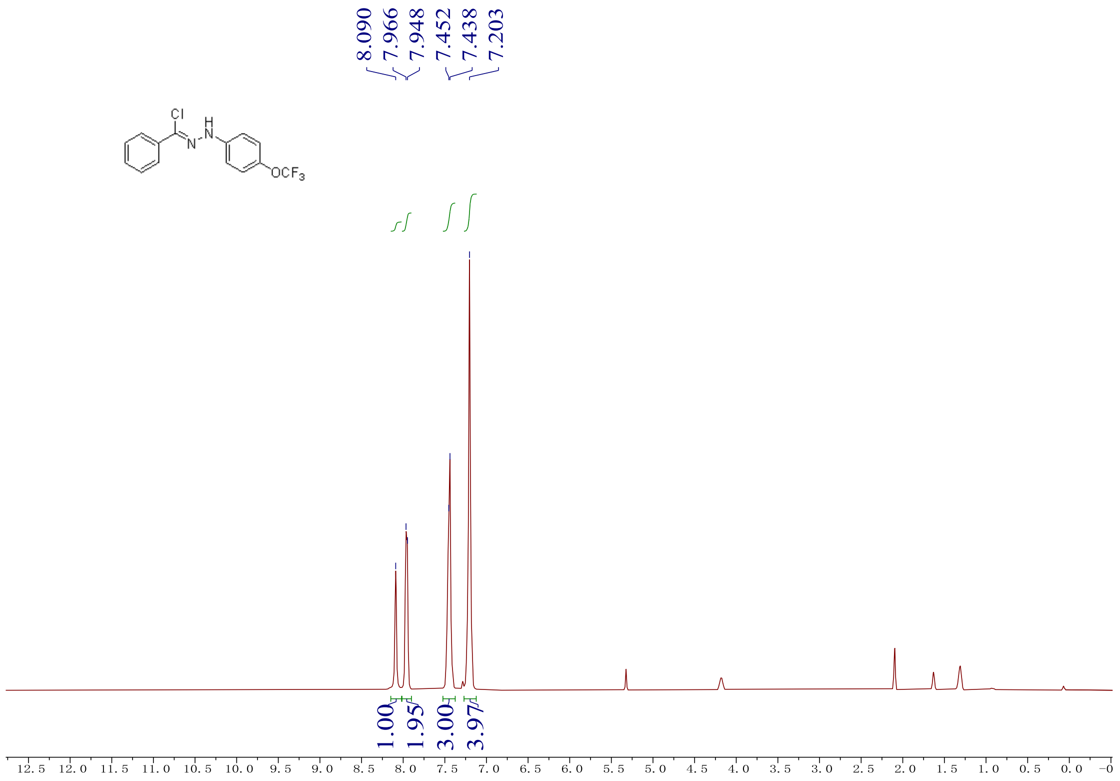

^{13}C{^{1}H} NMR spectra of 2u (101 MHz, $CDCl_3$)

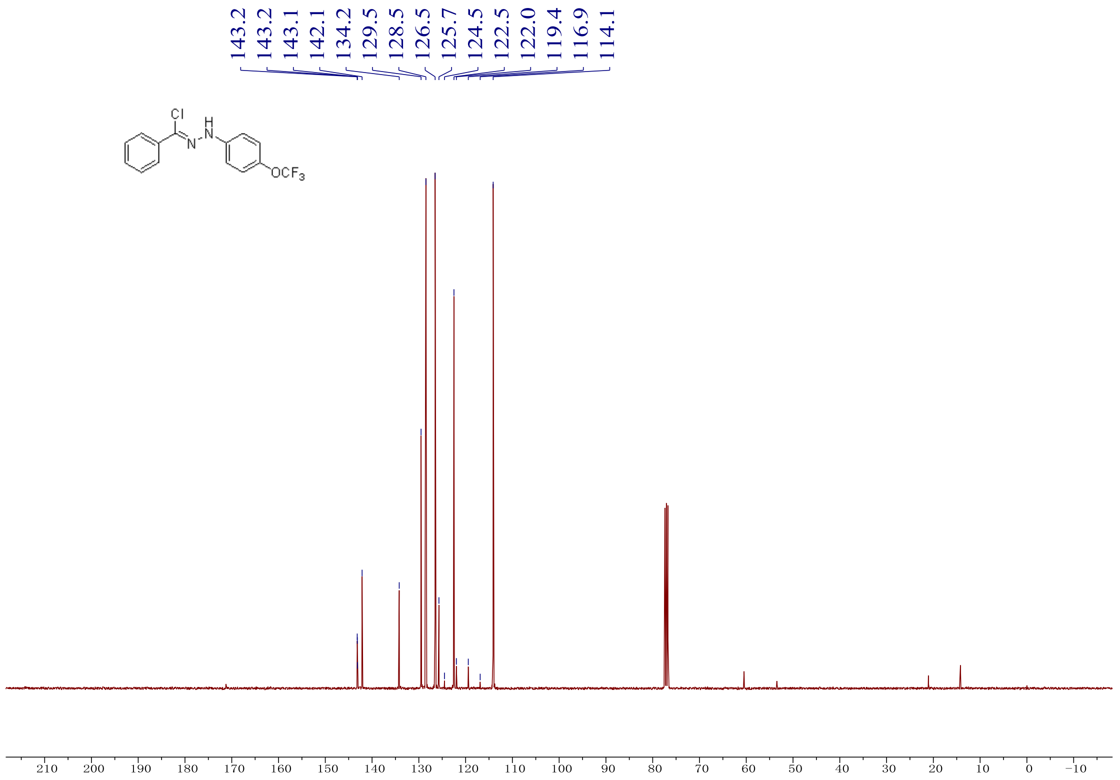

^{1}H NMR spectra of 2aa (400 MHz, $CDCl_3$)

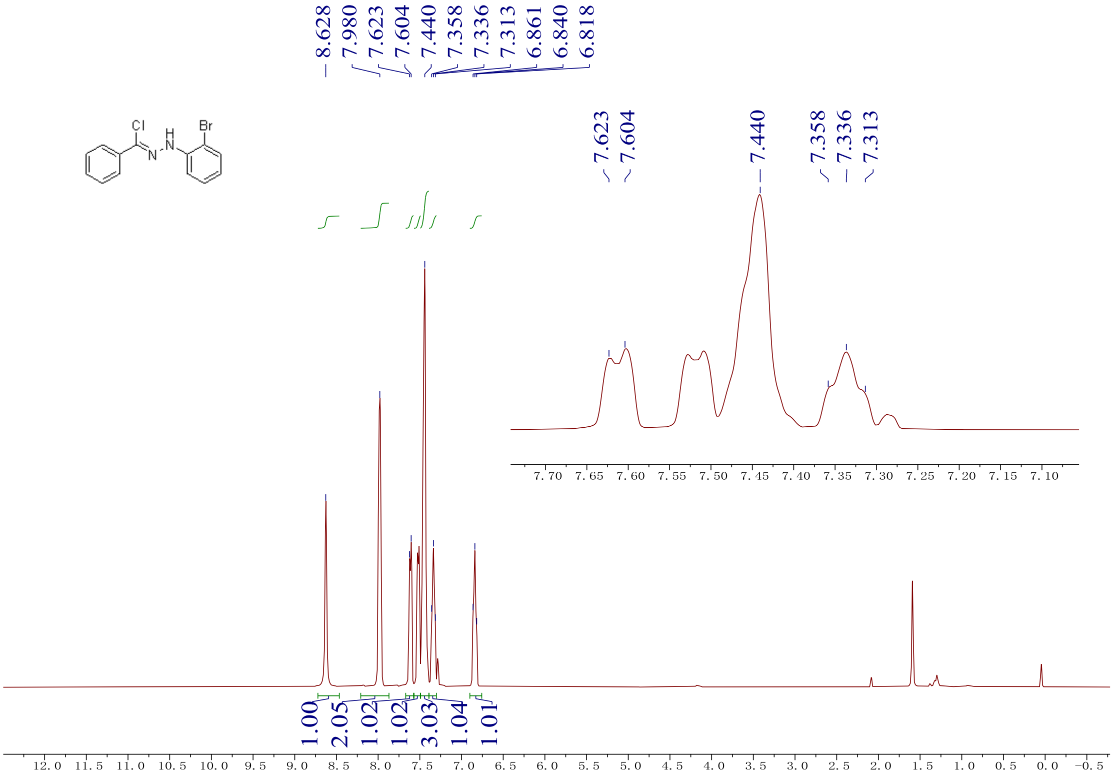

$^{13}C\{^{1}H\}$ NMR spectra of 2aa (101 MHz, $CDCl_3$)

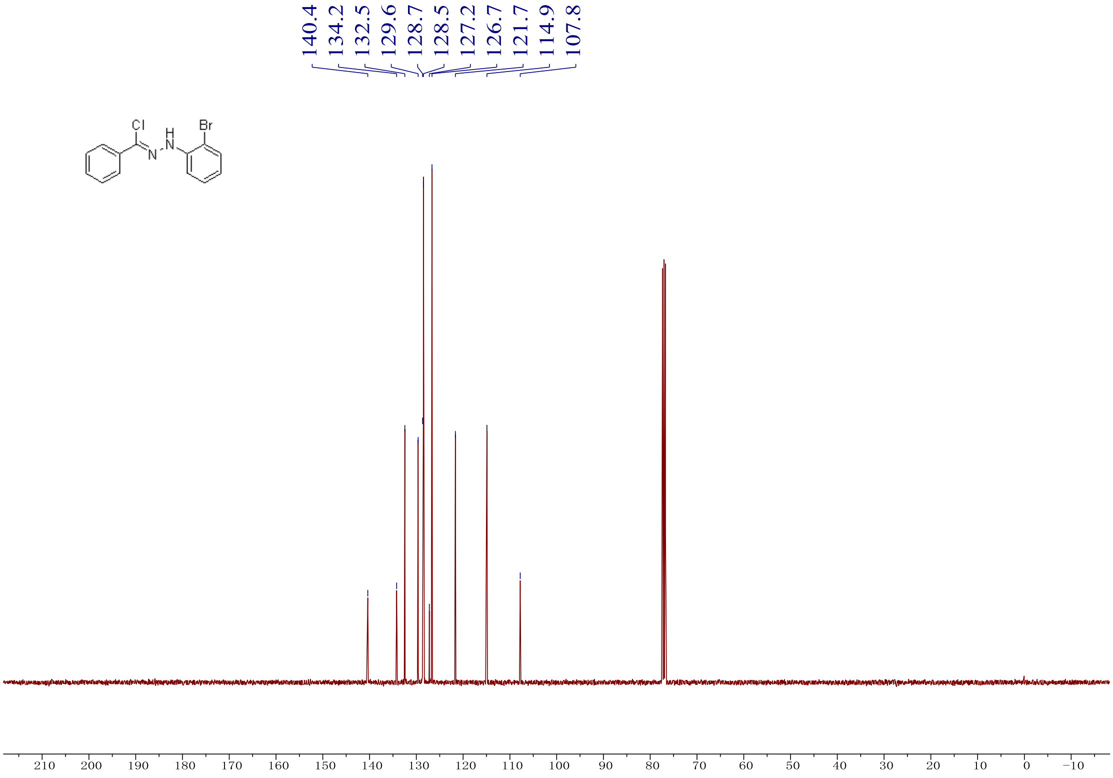

^{1}H NMR spectra of 2ac (400 MHz, $CDCl_3$)

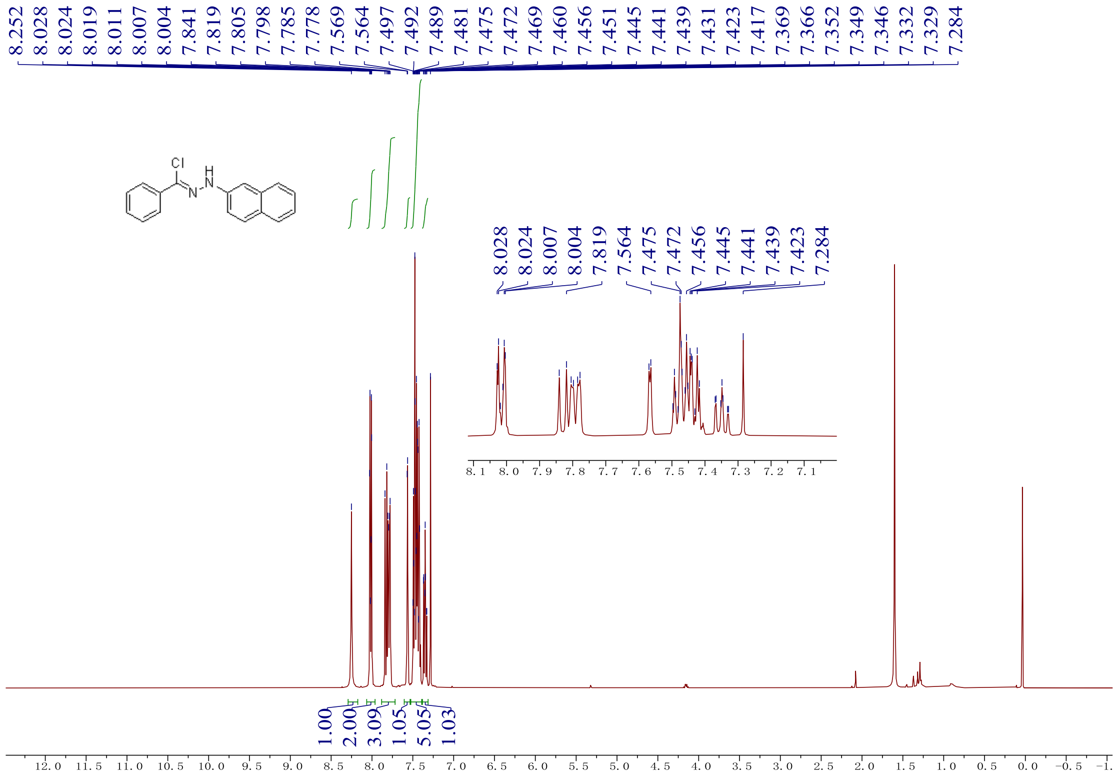

^{13}C{^{1}H} NMR spectra of 2ac (101 MHz, $CDCl_3$)

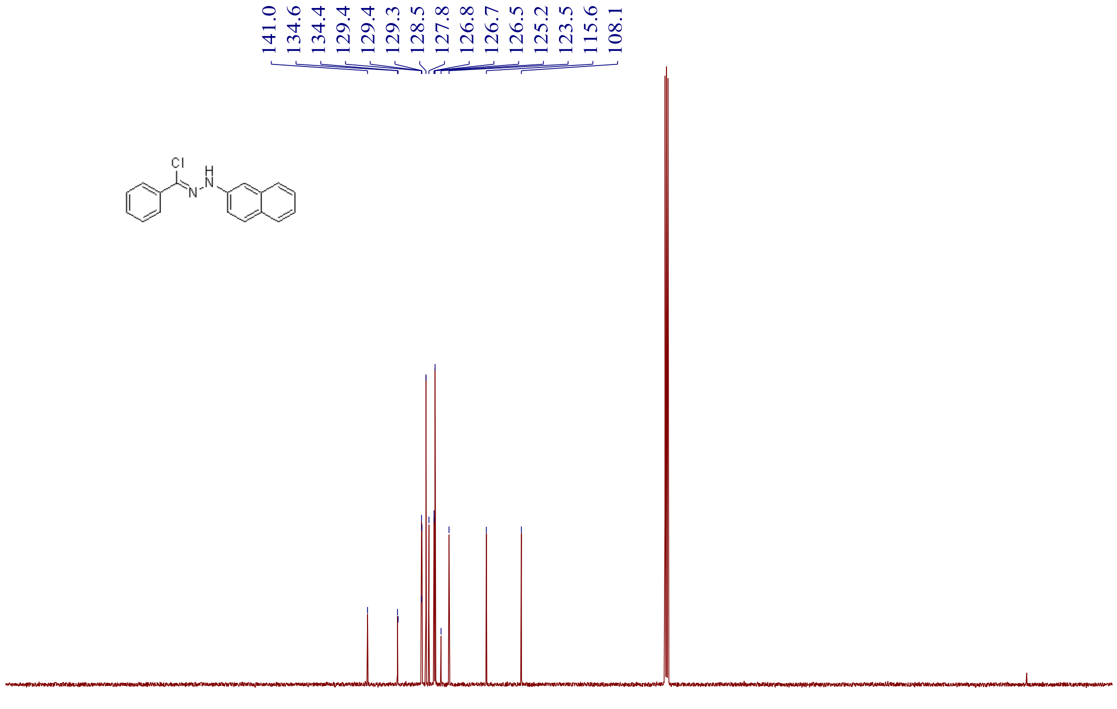

^{1}H NMR spectra of 3a (400 MHz, $CDCl_3$)

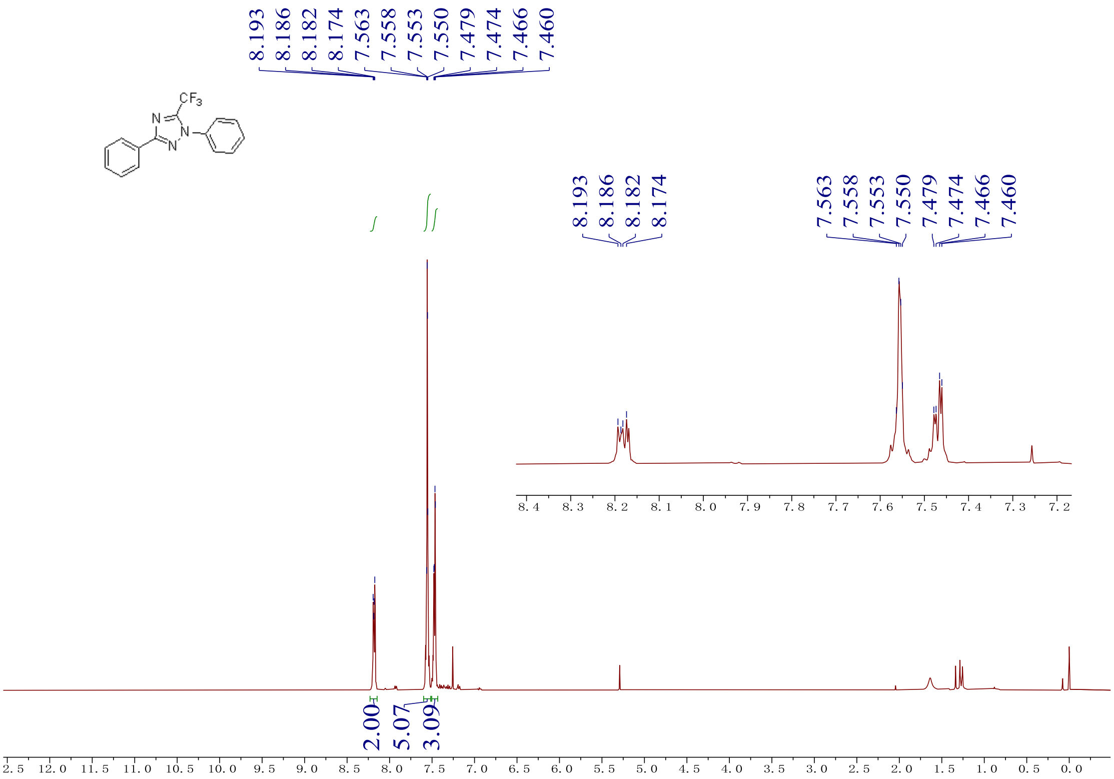

^{13}C{^{1}H} NMR spectra of 3a (101 MHz, $CDCl_3$)

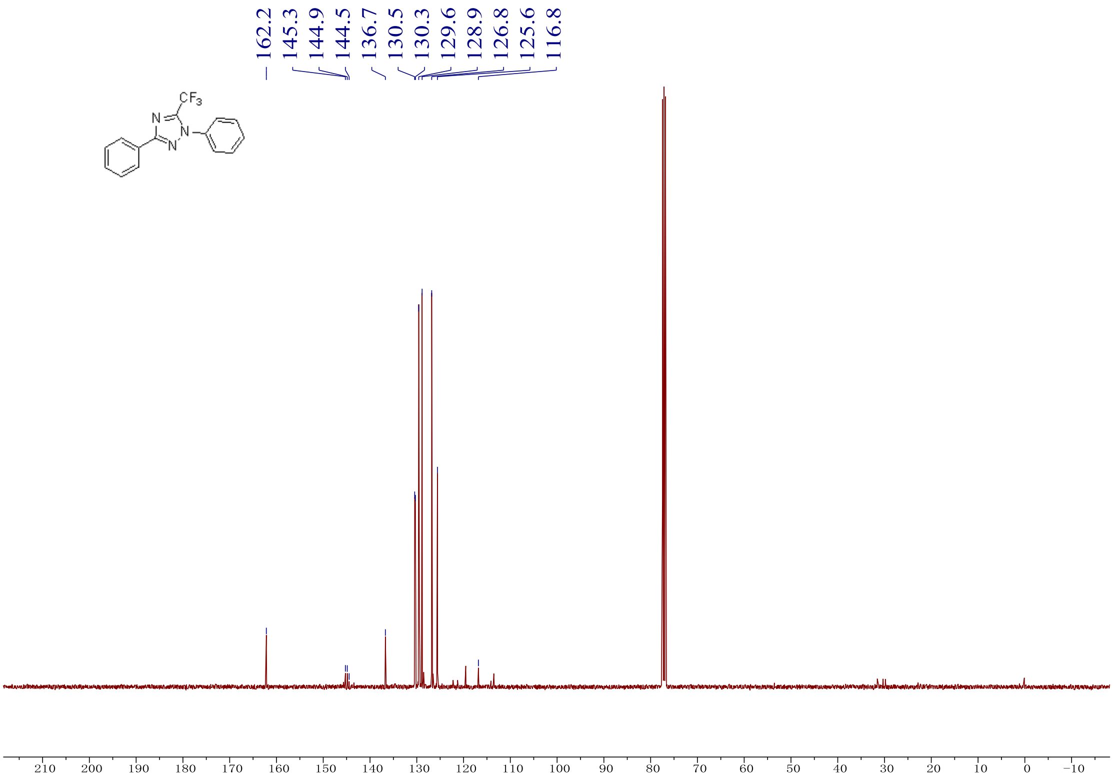

^{19}F NMR spectra of 3a (376 MHz, $CDCl_3$)

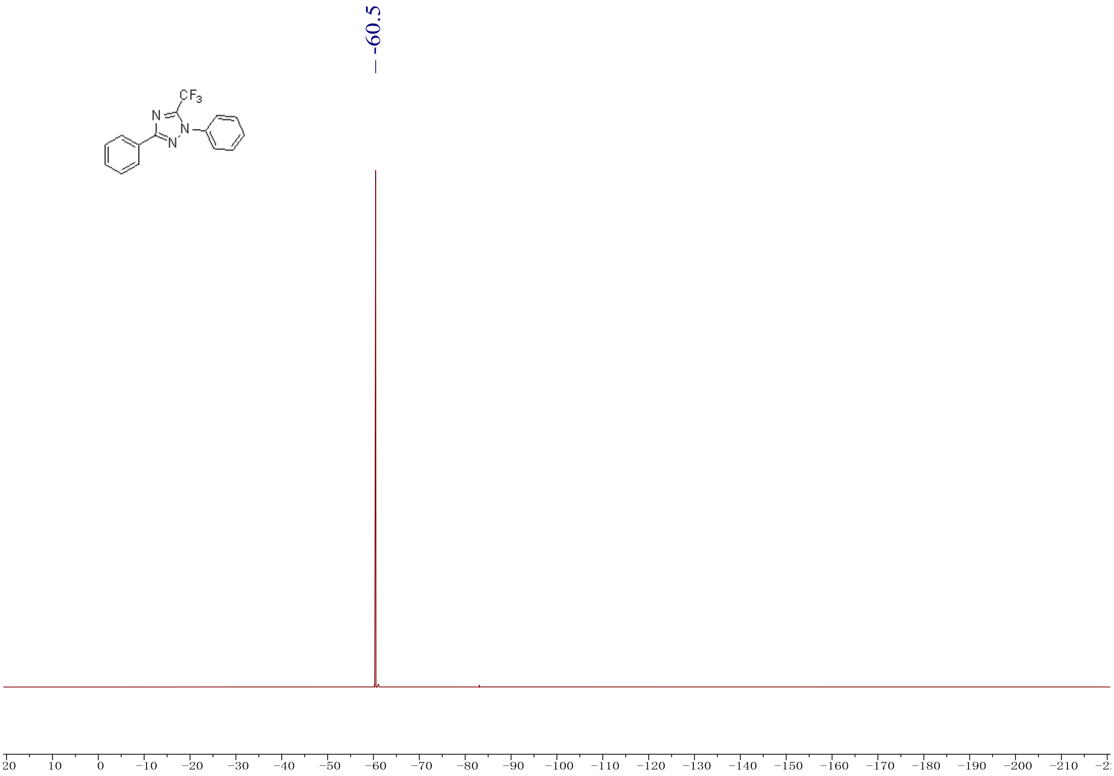

^{1}H NMR spectra of 3b (400 MHz, $CDCl_3$)

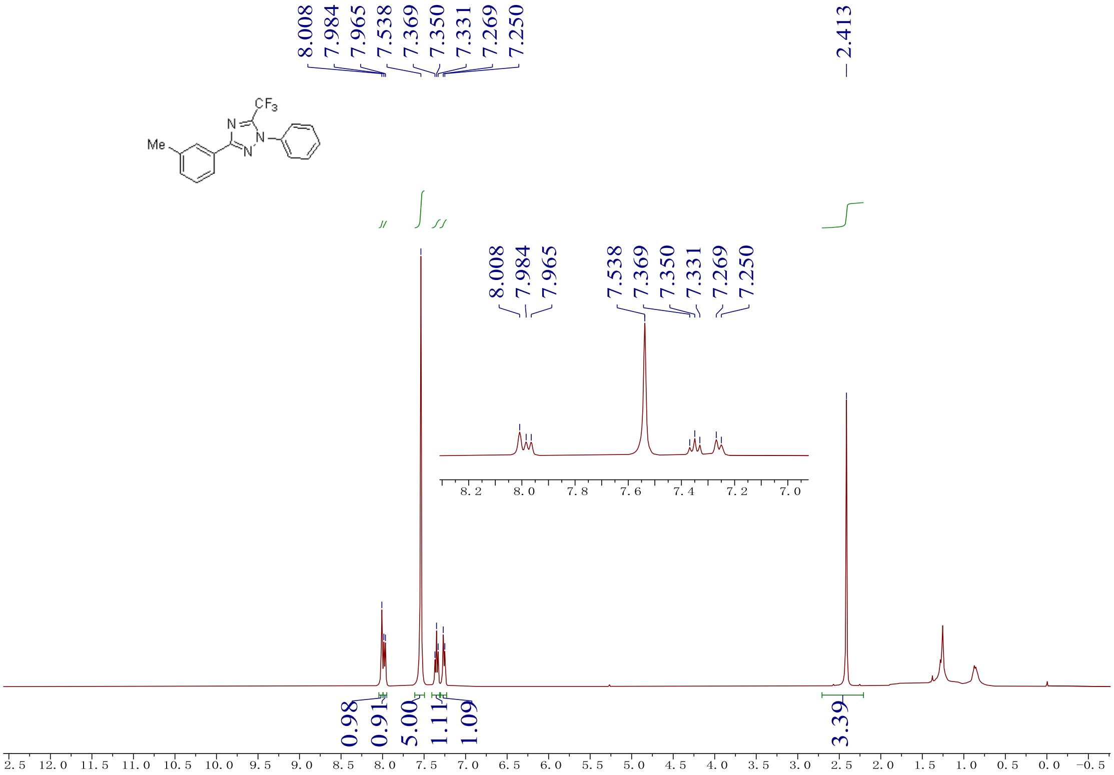

$^{13}C\{^{1}H\}$ NMR spectra of 3b (101 MHz, $CDCl_3$)

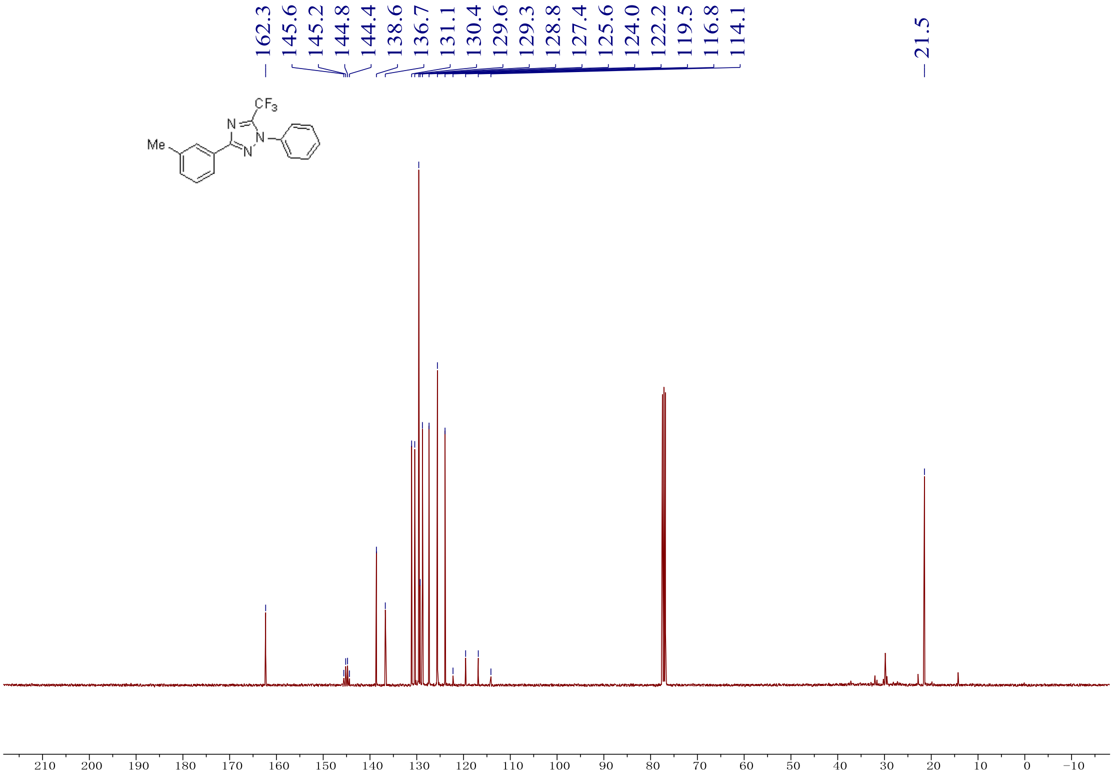

^{19}F NMR spectra of 3b (376 MHz, $CDCl_3$)

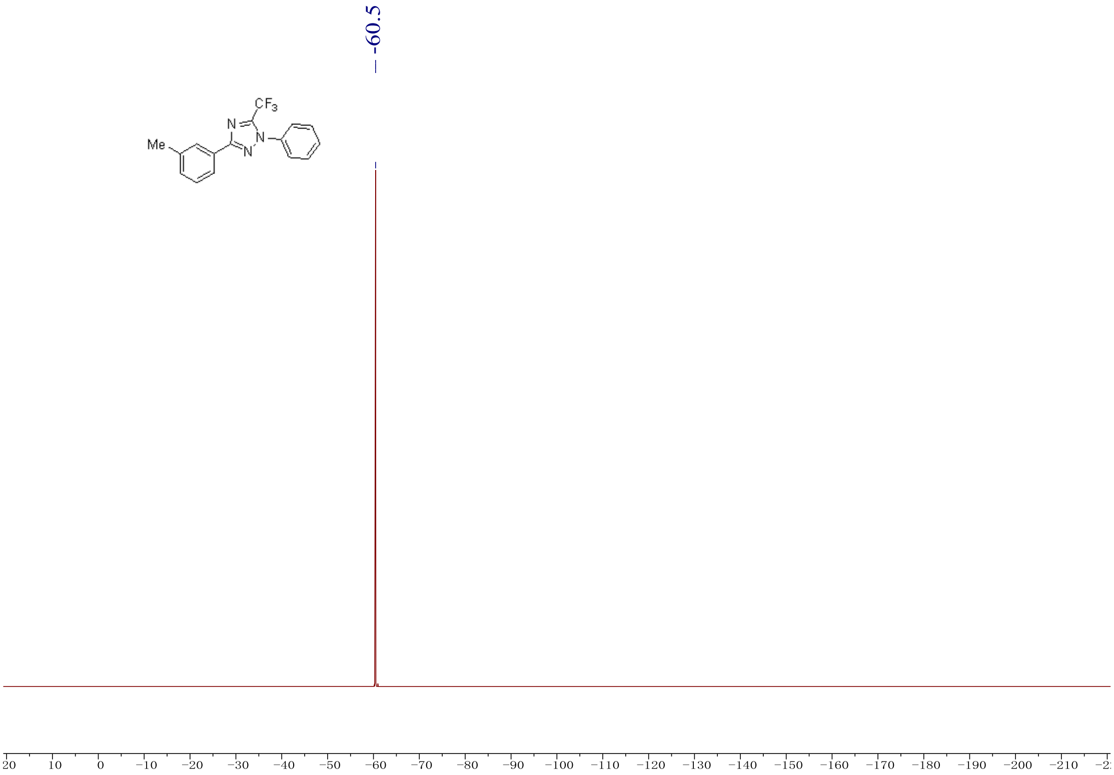

^{1}H NMR spectra of 3c (400 MHz, $CDCl_3$)

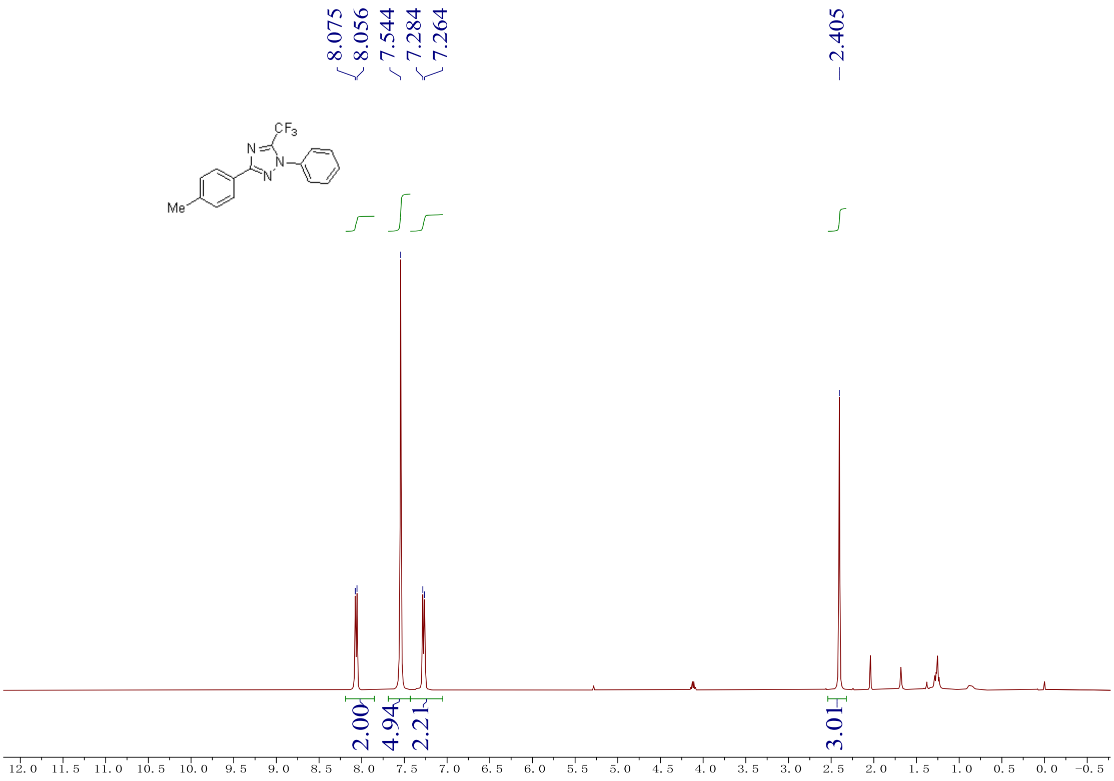

$^{13}C\{^{1}H\}$ NMR spectra of 3c (101 MHz, $CDCl_3$)

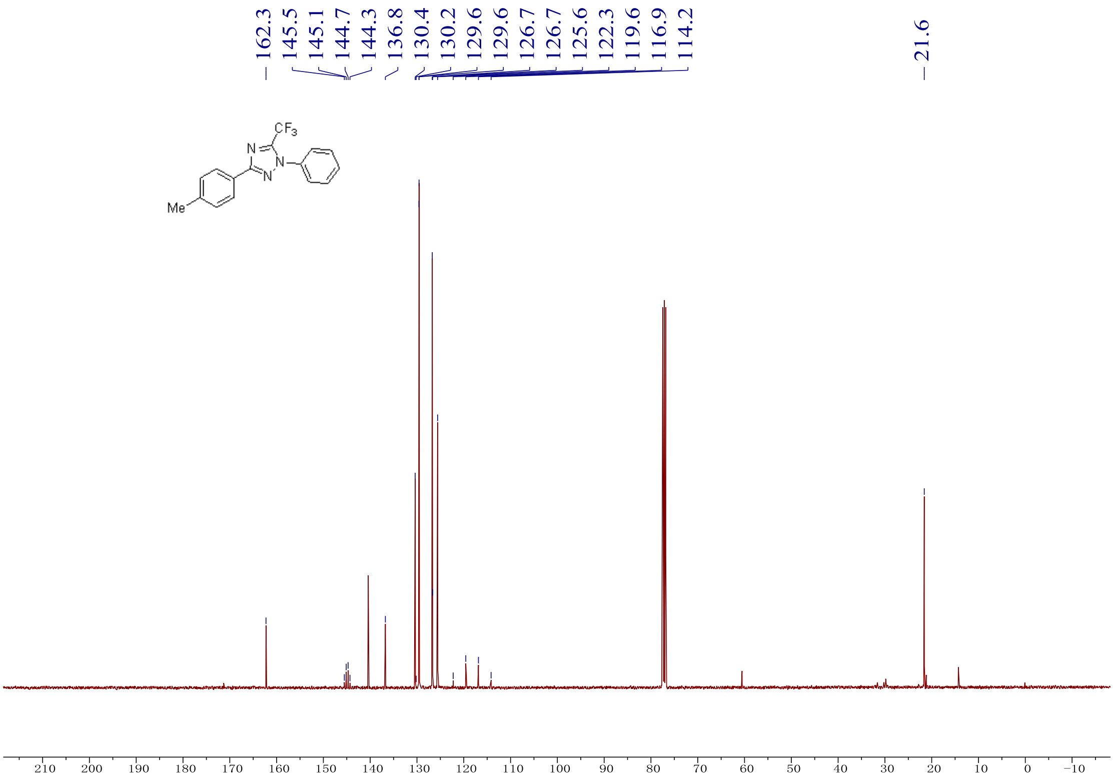

^{19}F NMR spectra of 3c (376 MHz, $CDCl_3$)

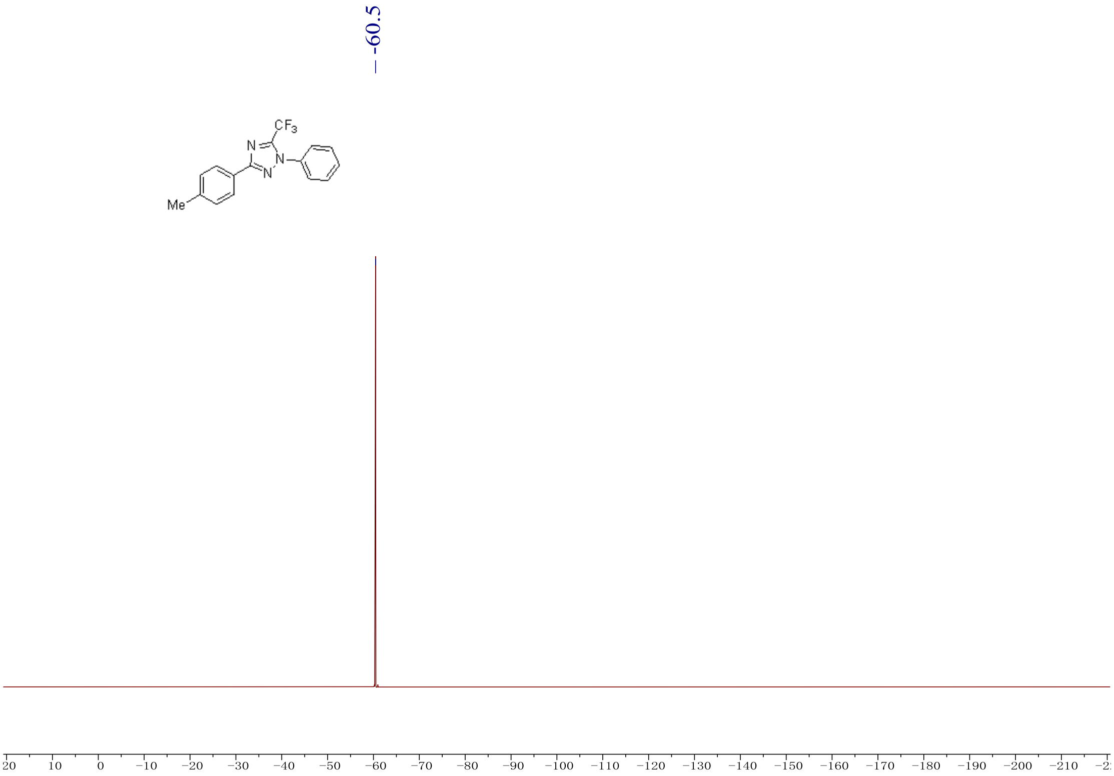

^{1}H NMR spectra of 3d (400 MHz, $CDCl_3$)

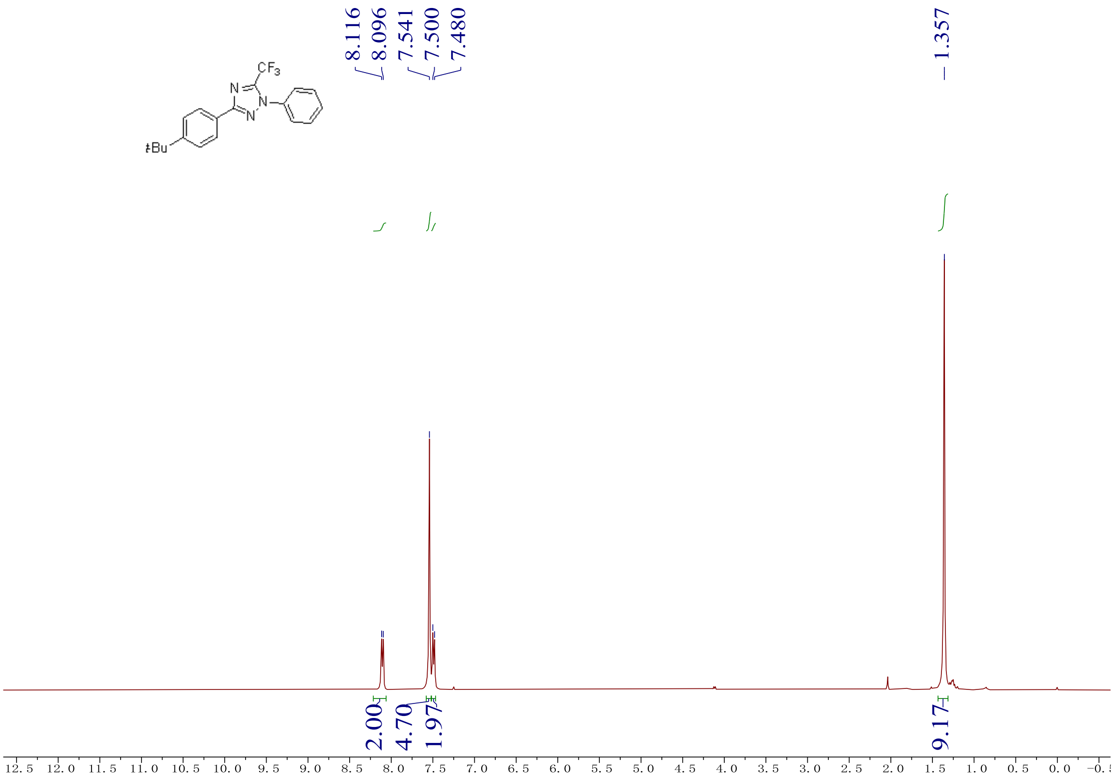

$^{13}C\{^{1}H\}$ NMR spectra of 3d (101 MHz, $CDCl_3$)

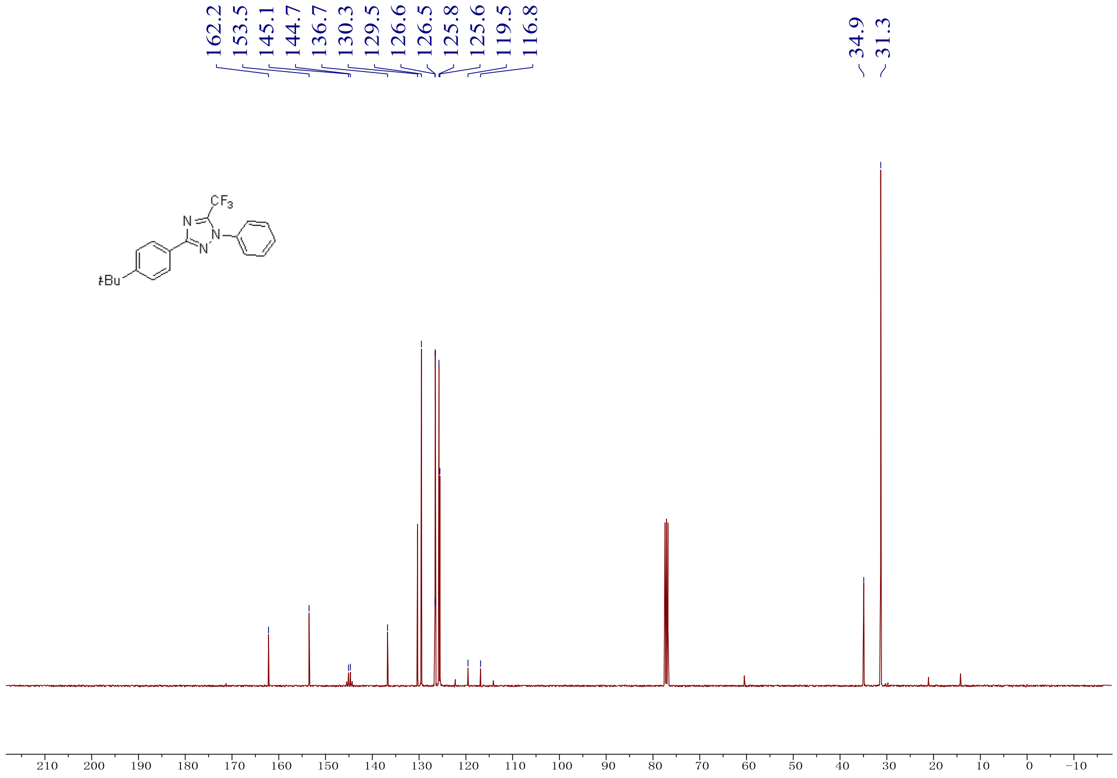

^{19}F NMR spectra of 3d (376 MHz, $CDCl_3$)

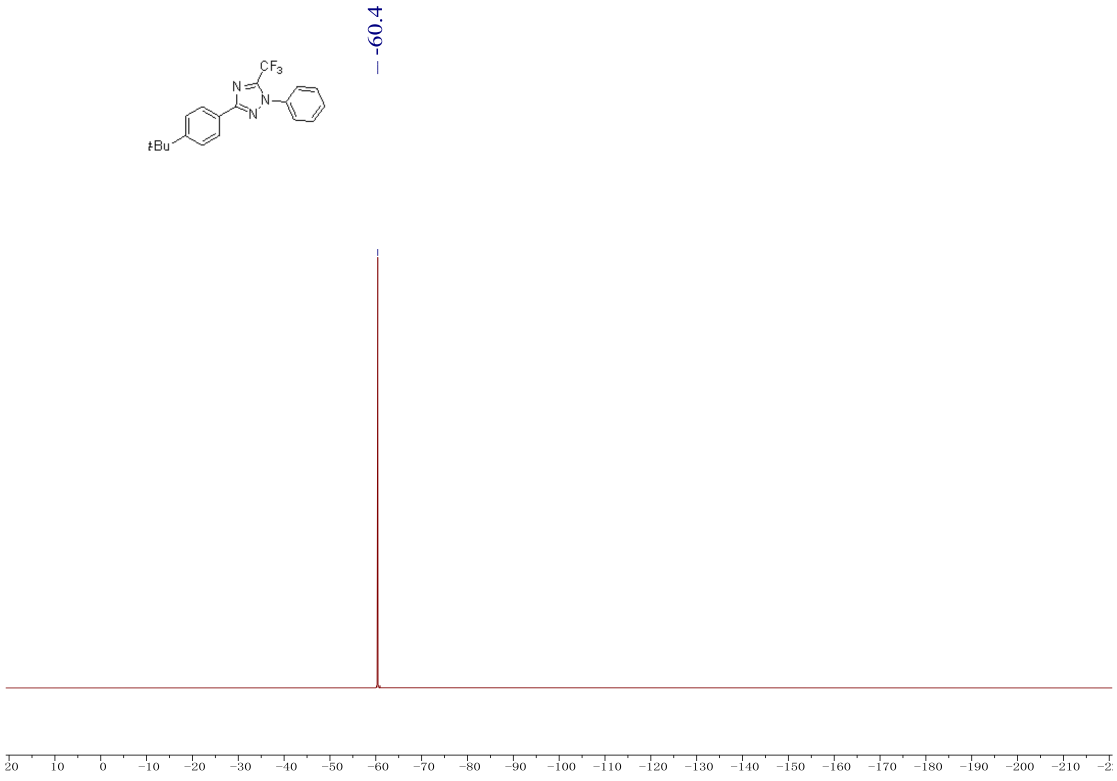

^{1}H NMR spectra of 3e (400 MHz, $CDCl_3$)

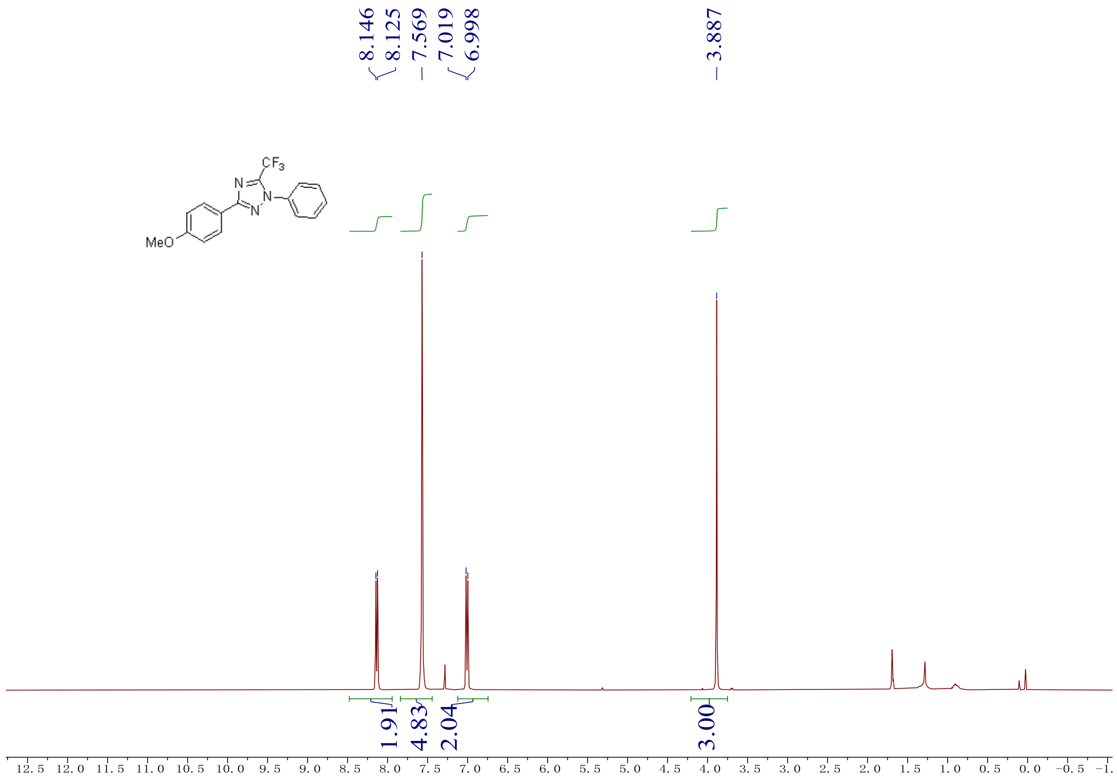

$^{13}C\{^{1}H\}$ NMR spectra of 3e (101 MHz, $CDCl_3$)

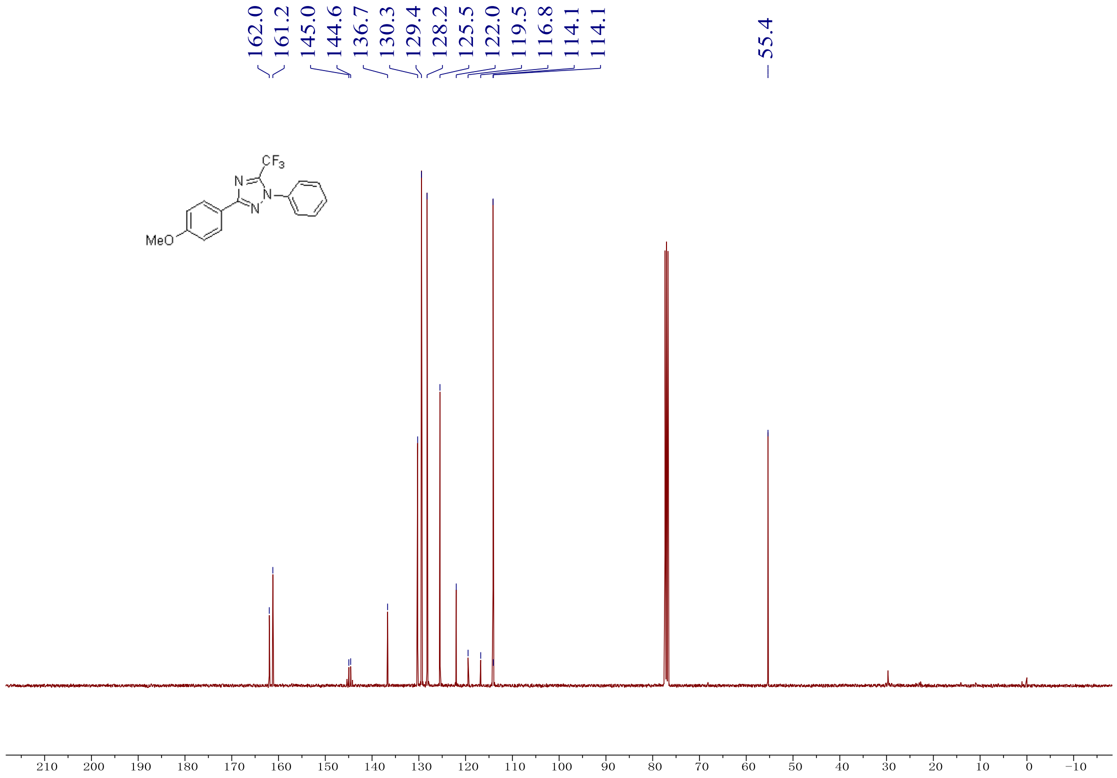

^{19}F NMR spectra of 3e (376 MHz, $CDCl_3$)

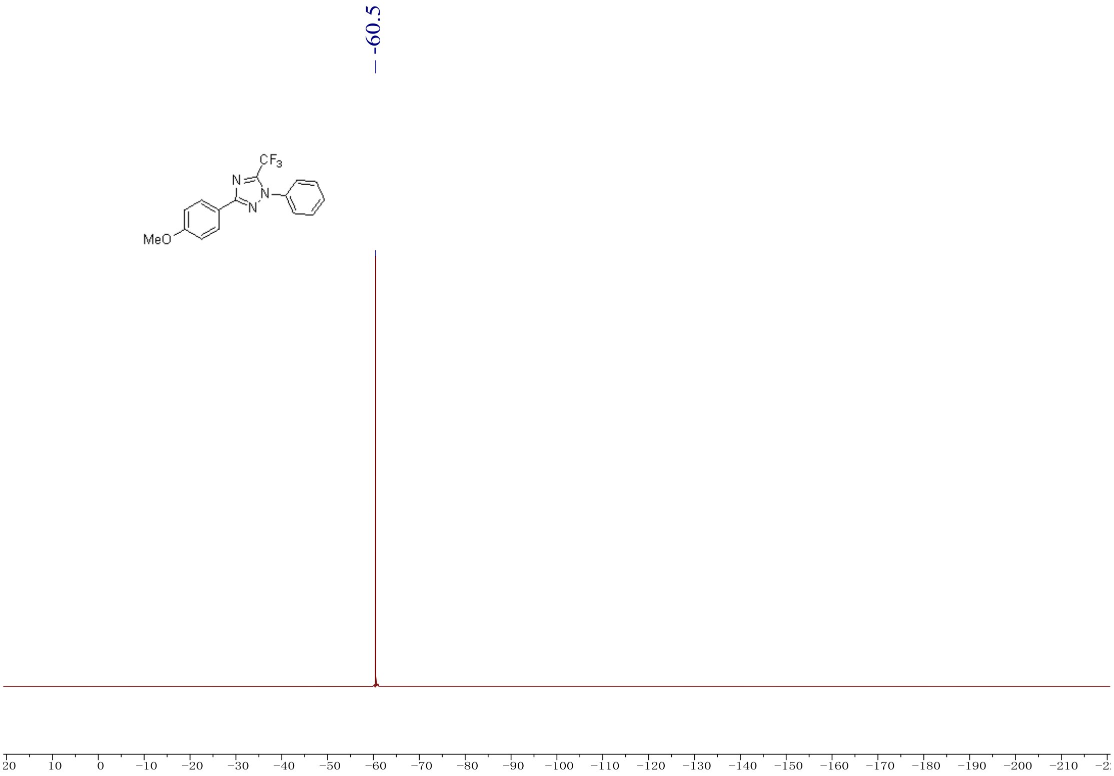

^{1}H NMR spectra of 3f (400 MHz, $CDCl_3$)

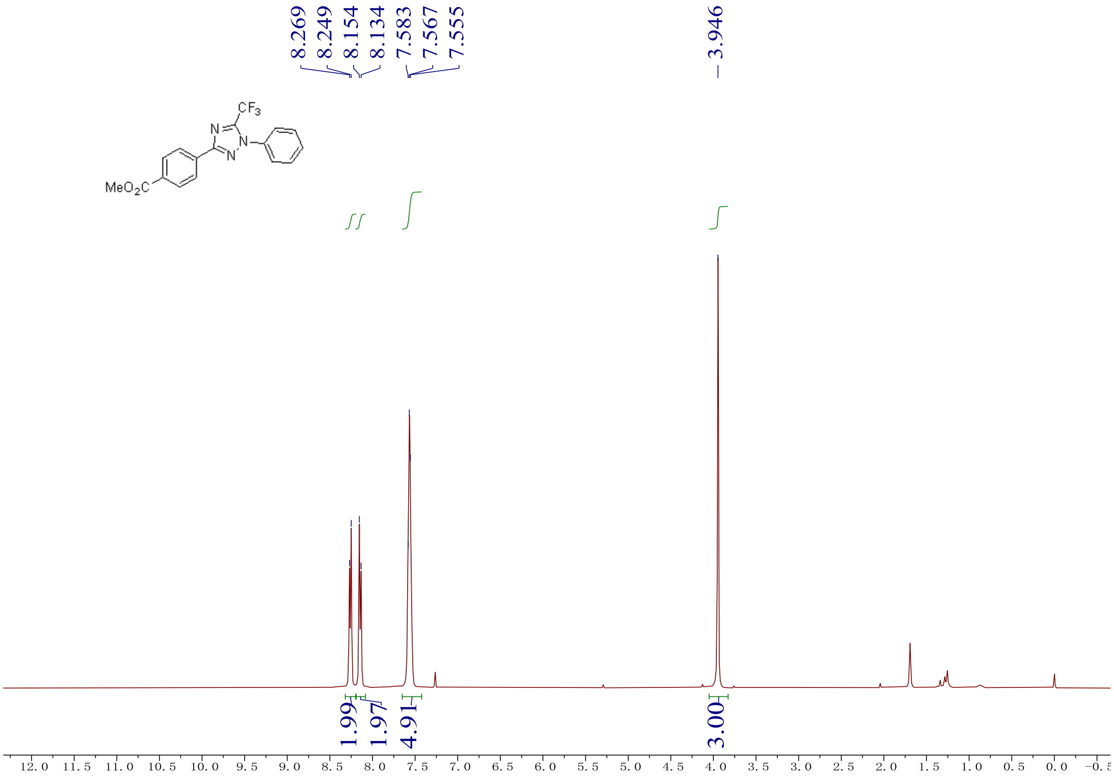

$^{13}C\{^{1}H\}$ NMR spectra of 3f (101 MHz, CDCl$_3$)

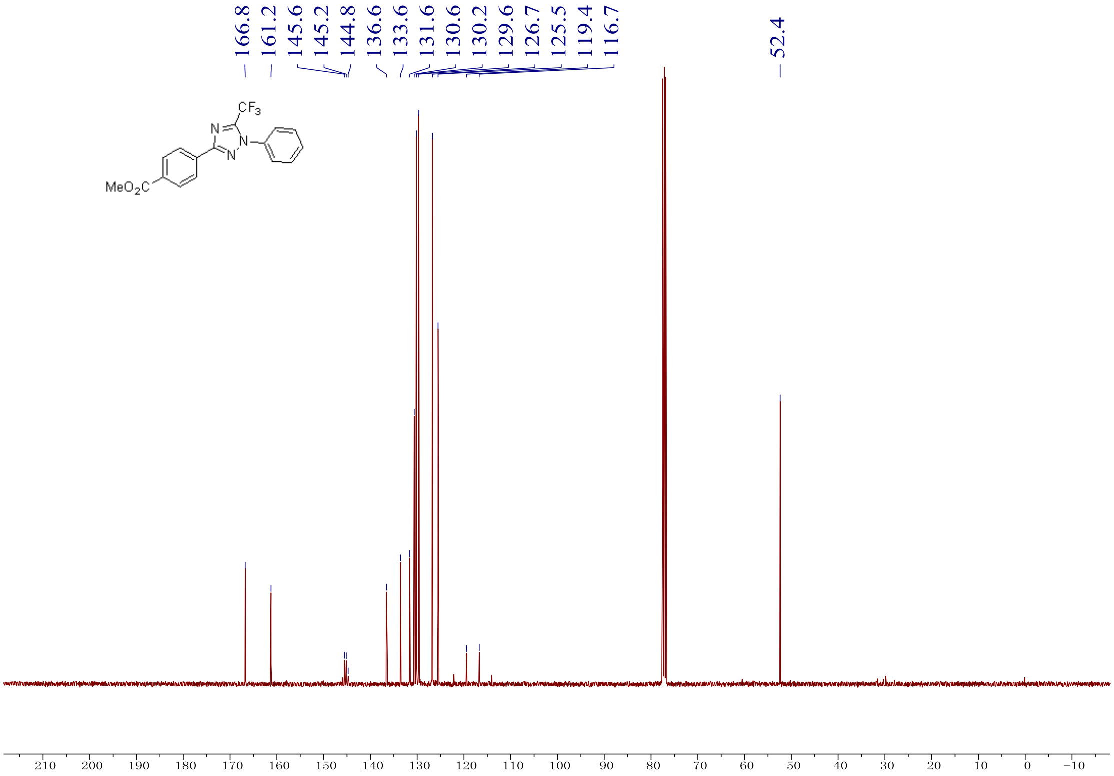

^{19}F NMR spectra of 3f (376 MHz, CDCl$_3$)

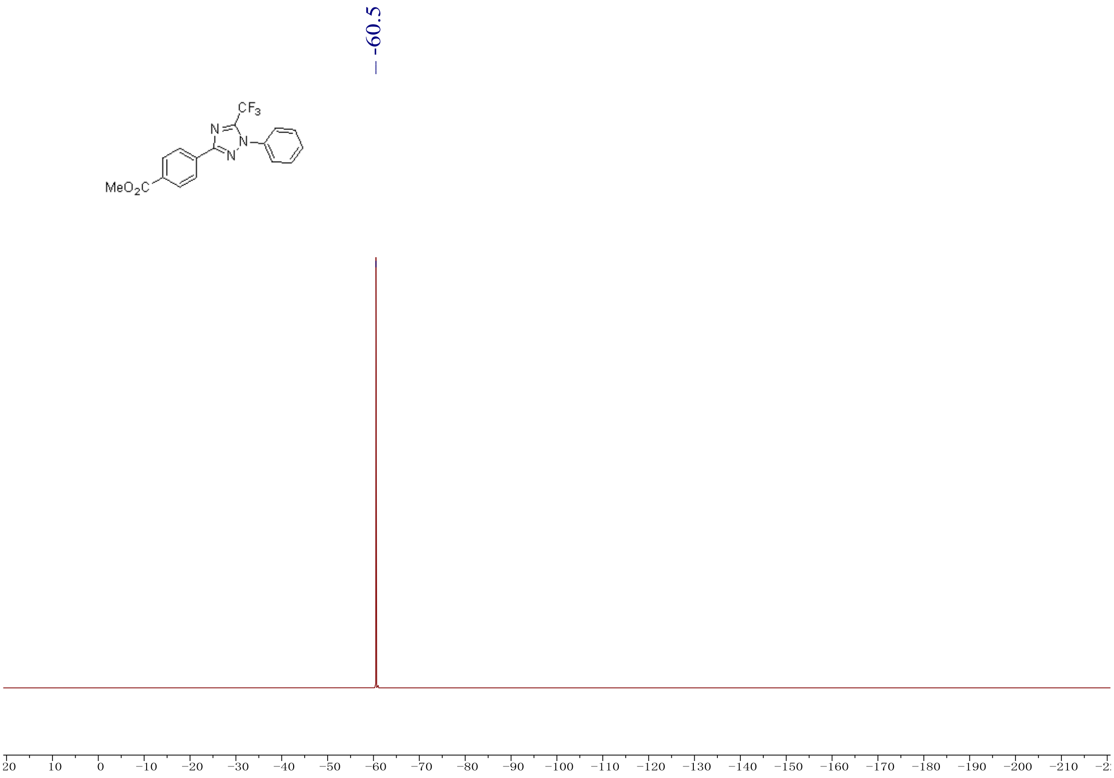

^{1}H NMR spectra of 3g (400 MHz, $CDCl_3$)

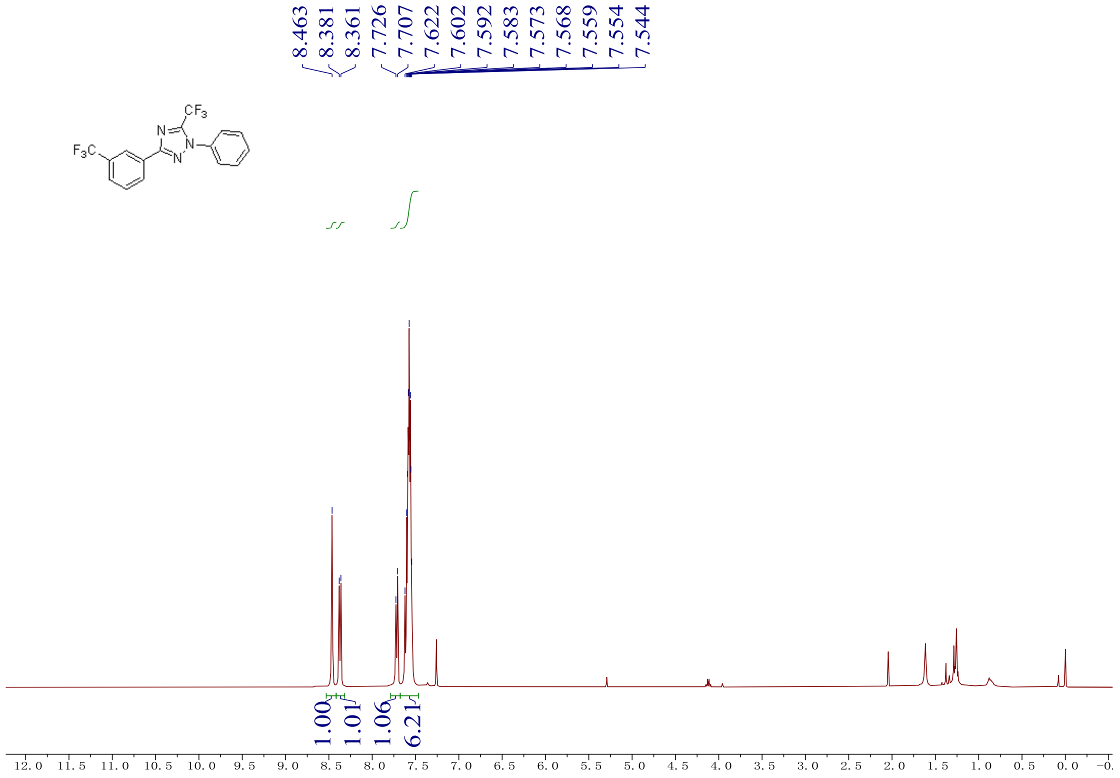

^{13}C{^{1}H} NMR spectra of 3g (101 MHz, $CDCl_3$)

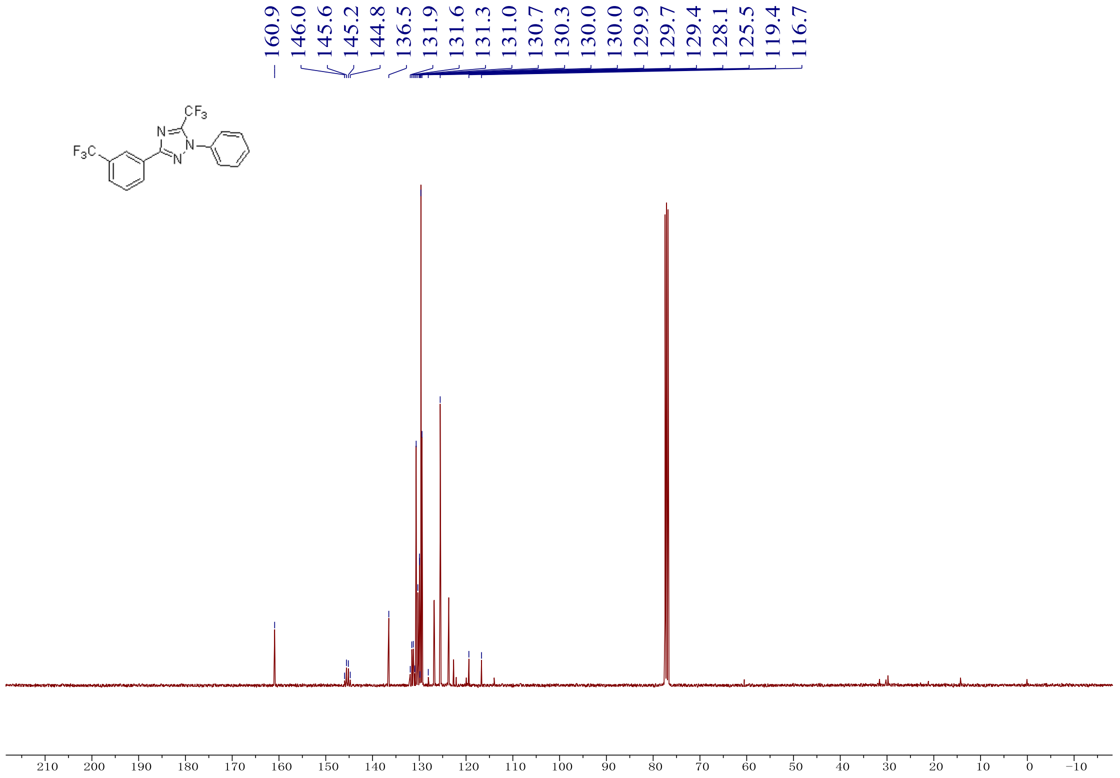

^{19}F NMR spectra of 3g (376 MHz, $CDCl_3$)

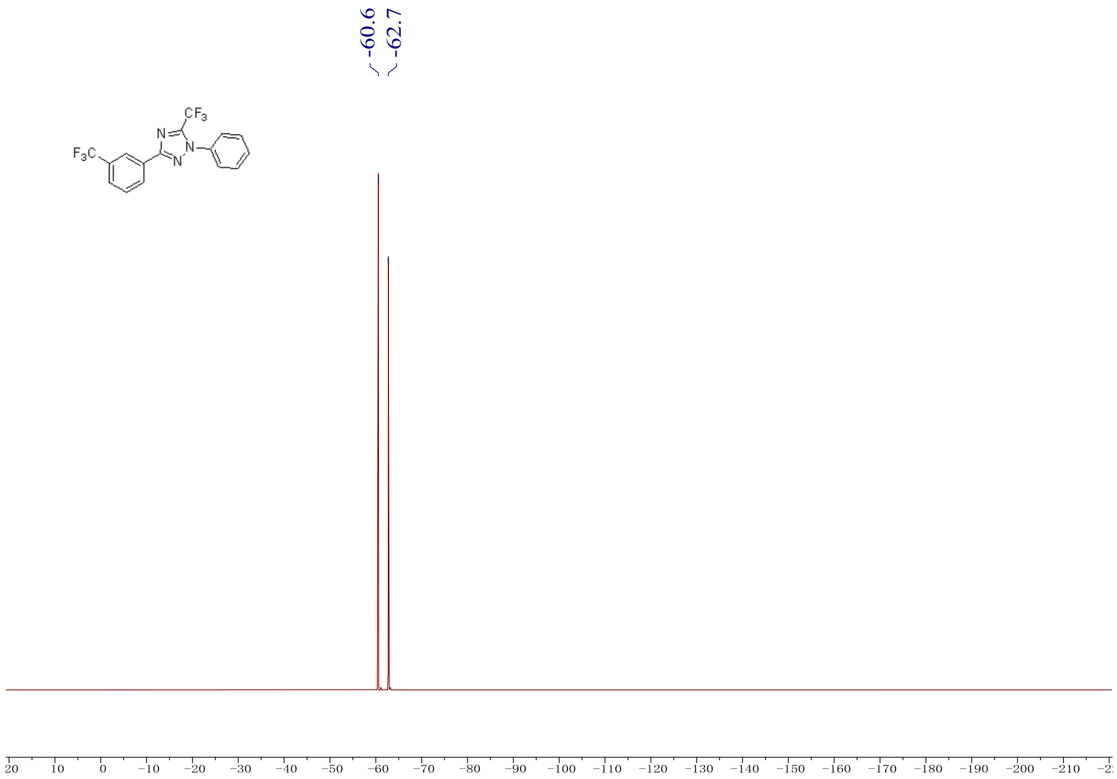

^{1}H NMR spectra of 3h (400 MHz, $CDCl_3$)

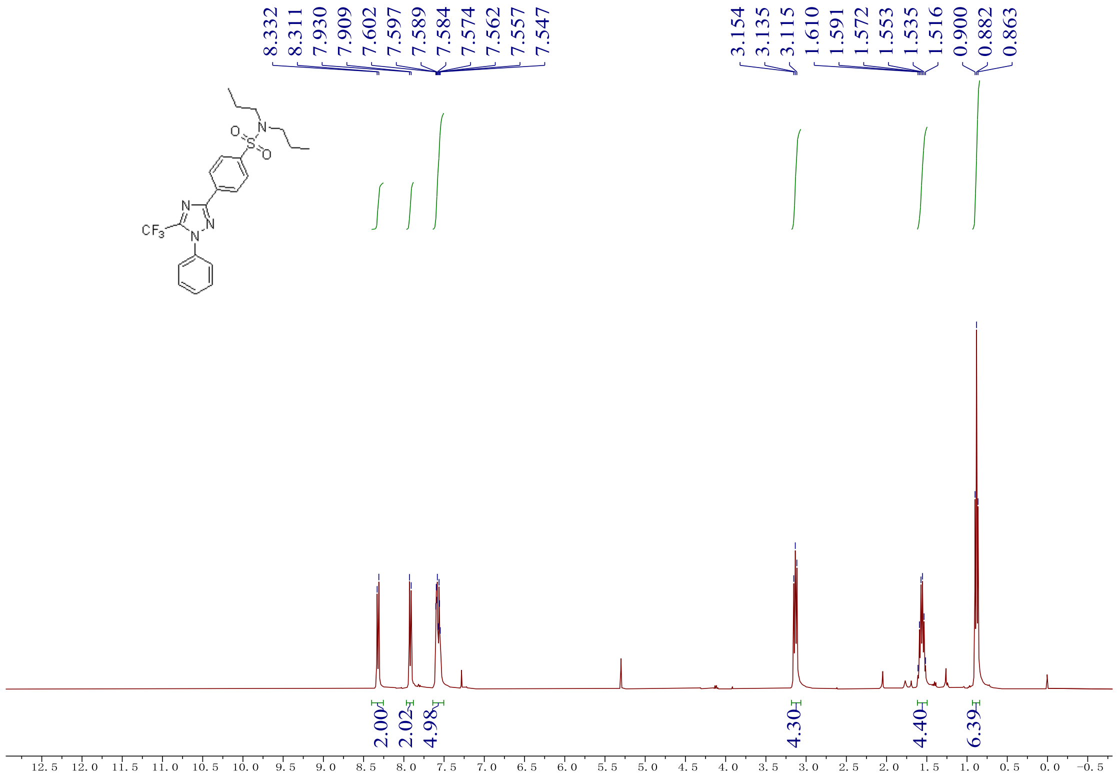

$^{13}C\{^{1}H\}$ NMR spectra of 3h (101 MHz, $CDCl_3$)

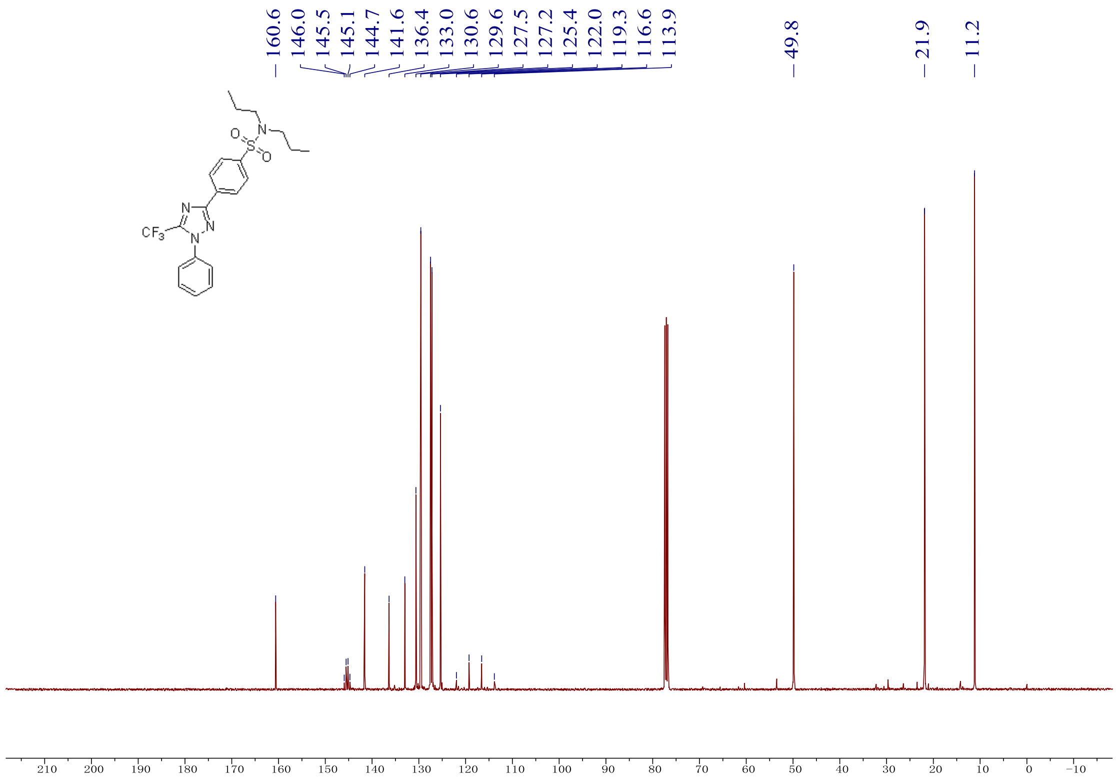

^{19}F NMR spectra of 3h (376 MHz, $CDCl_3$)

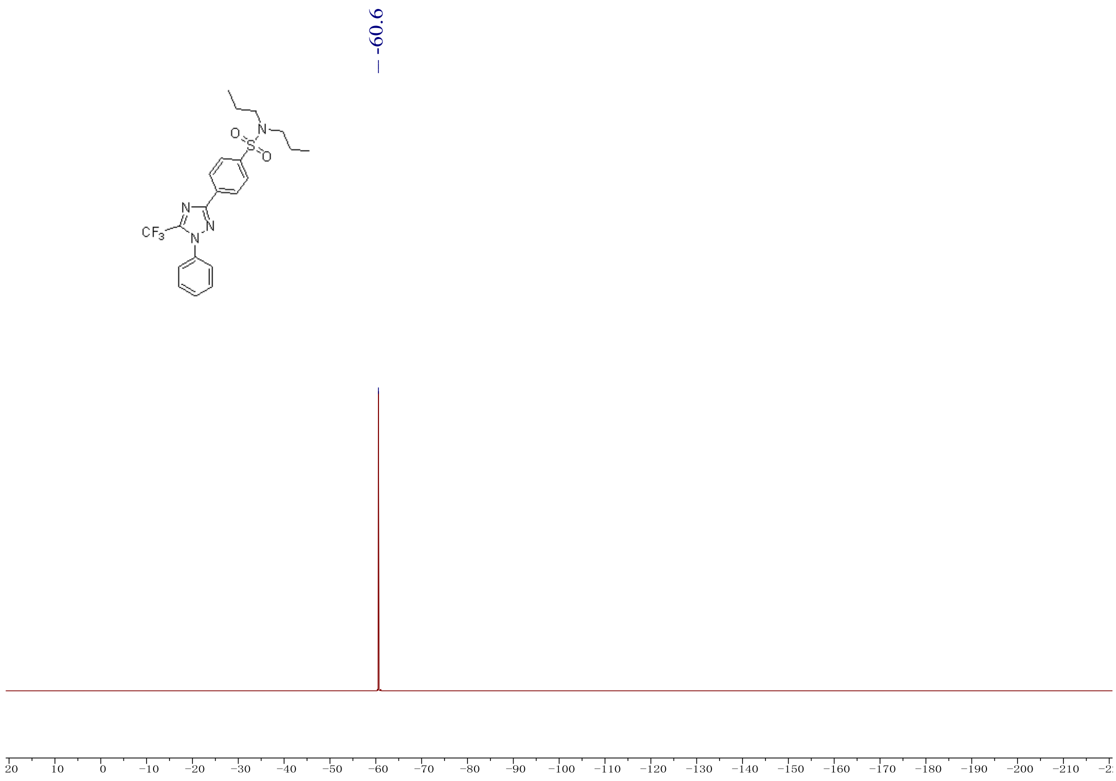

^{1}H NMR spectra of 3i (400 MHz, $CDCl_3$)

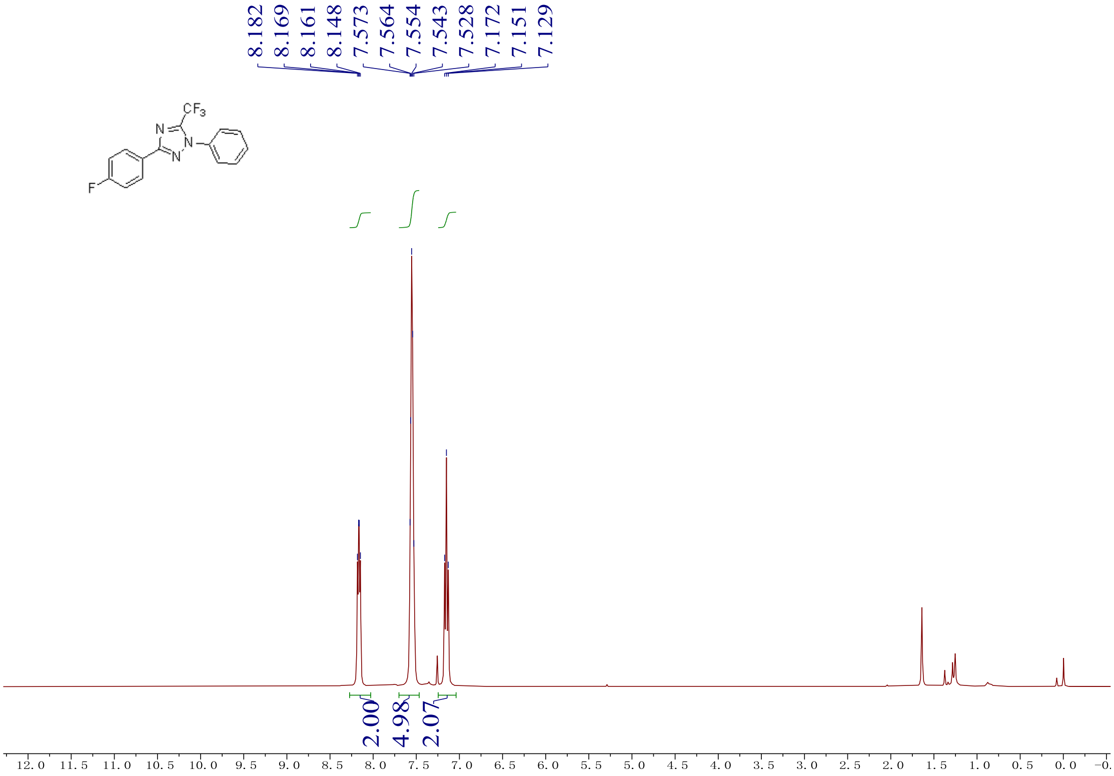

^{13}C{^{1}H} NMR spectra of 3i (101 MHz, $CDCl_3$)

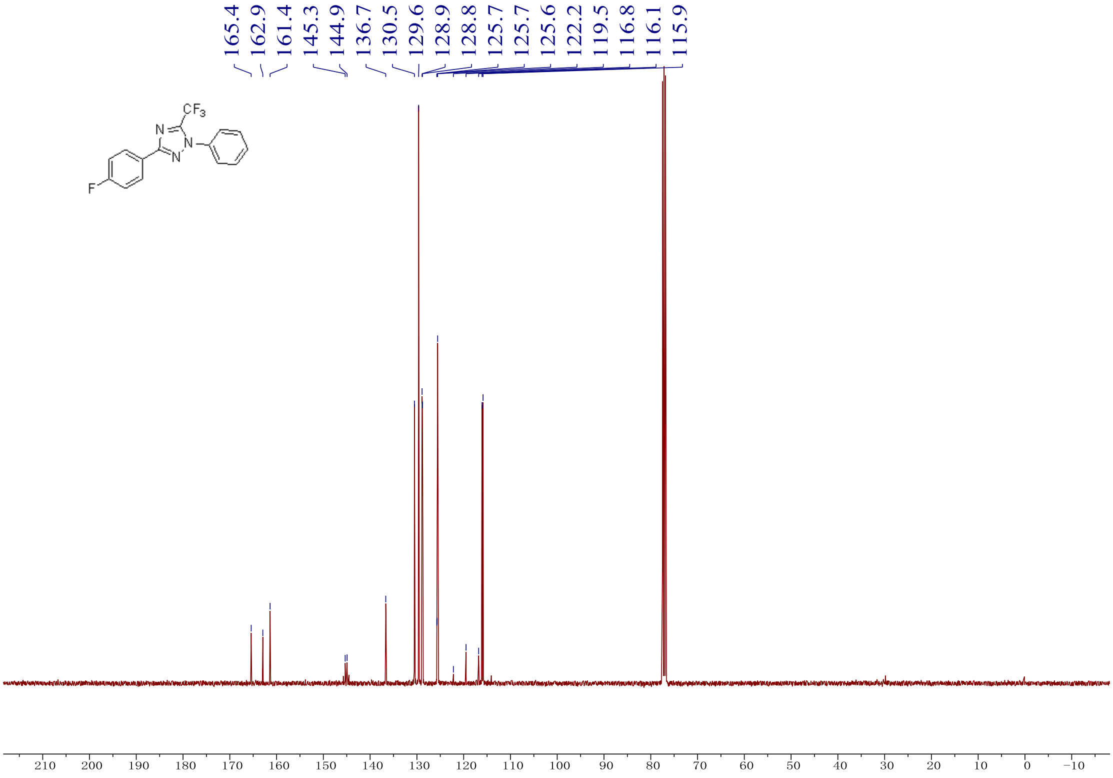

^{19}F NMR spectra of 3i (376 MHz, $CDCl_3$)

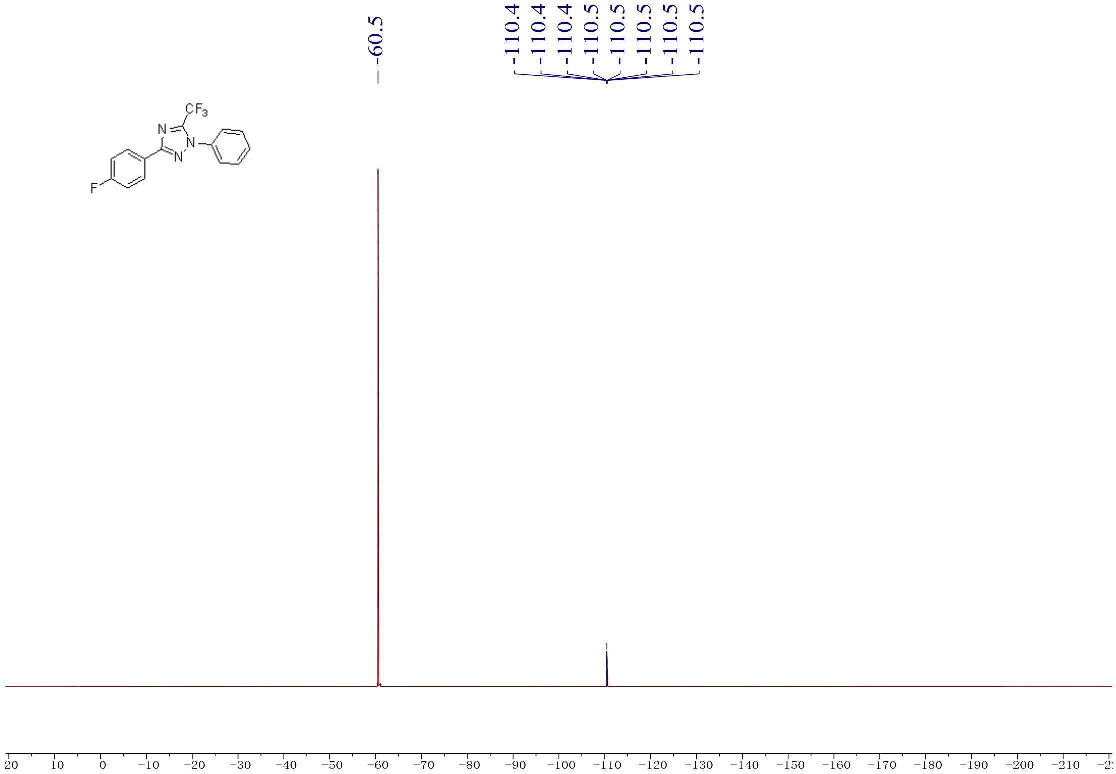

^{1}H NMR spectra of 3j (400 MHz, $CDCl_3$)

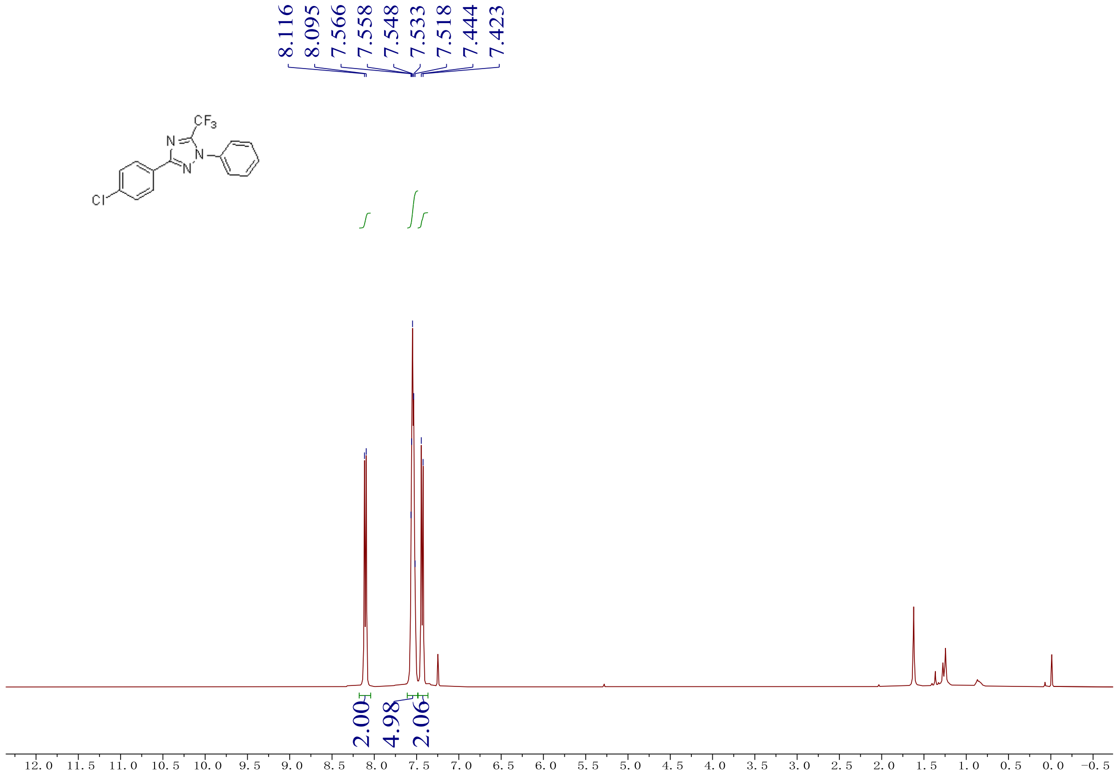

$^{13}C\{^1H\}$ NMR spectra of 3j (101 MHz, $CDCl_3$)

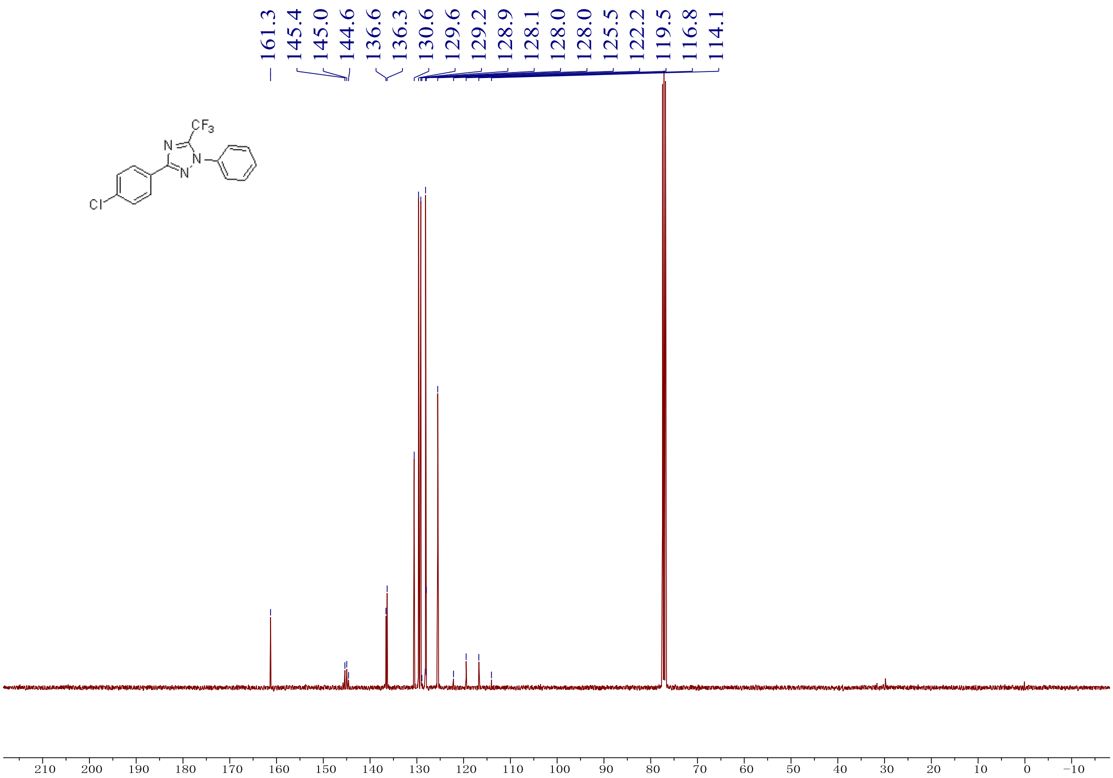

^{19}F NMR spectra of 3j (376 MHz, $CDCl_3$)

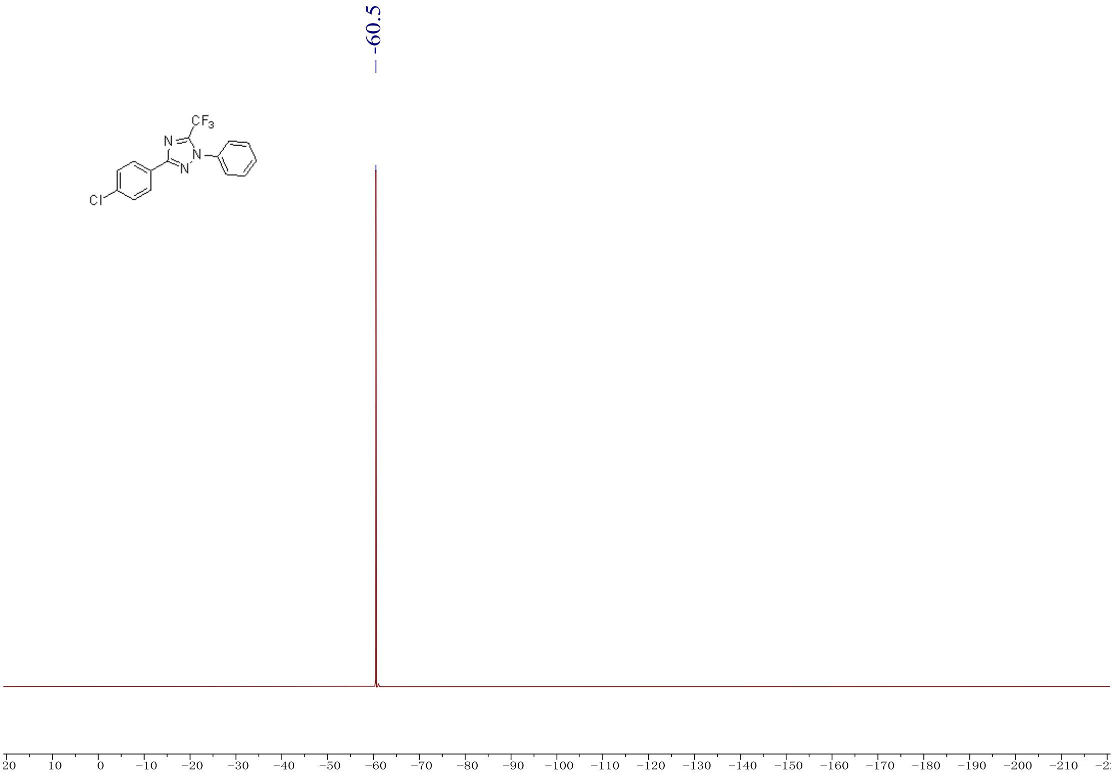

^{1}H NMR spectra of 3k (400 MHz, $CDCl_3$)

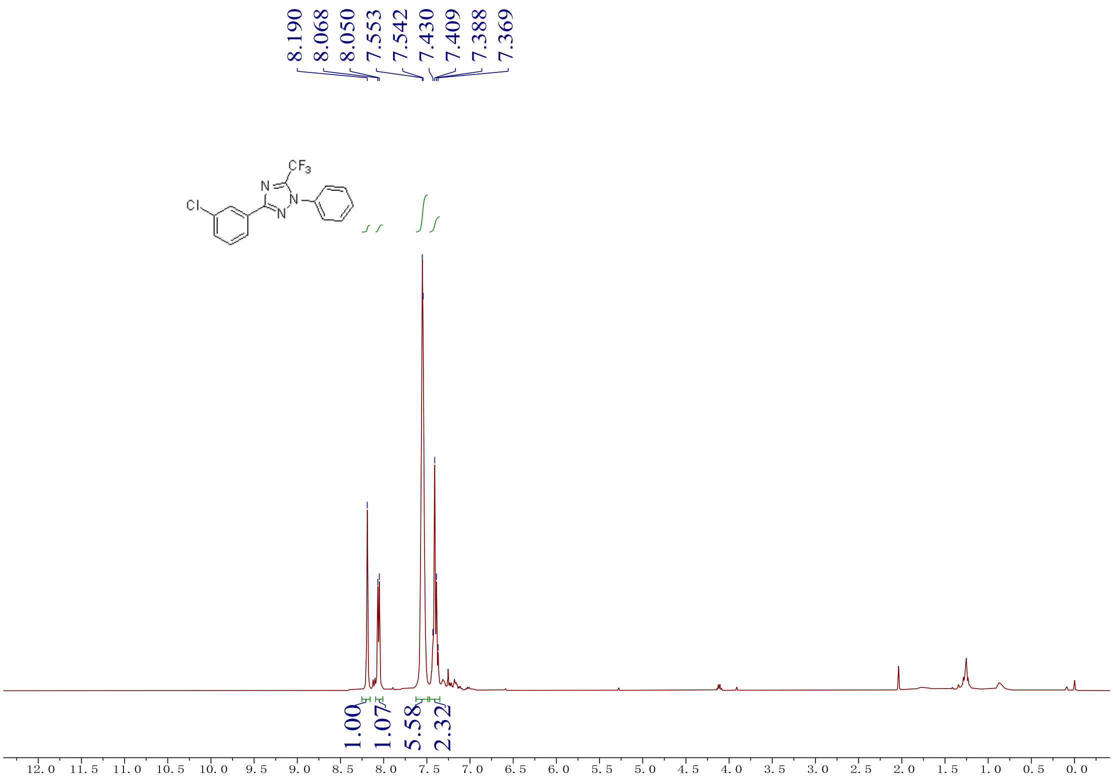

^{13}C{^{1}H} NMR spectra of 3k (101 MHz, $CDCl_3$)

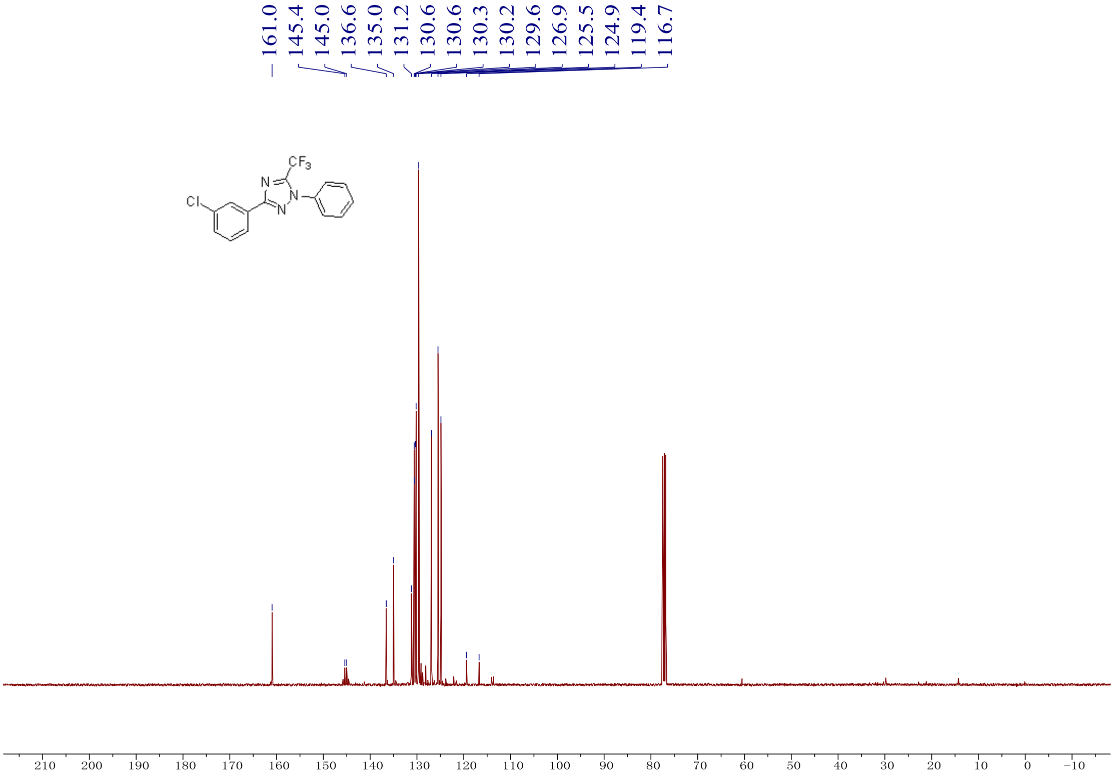

^{19}F NMR spectra of 3k (376 MHz, $CDCl_3$)

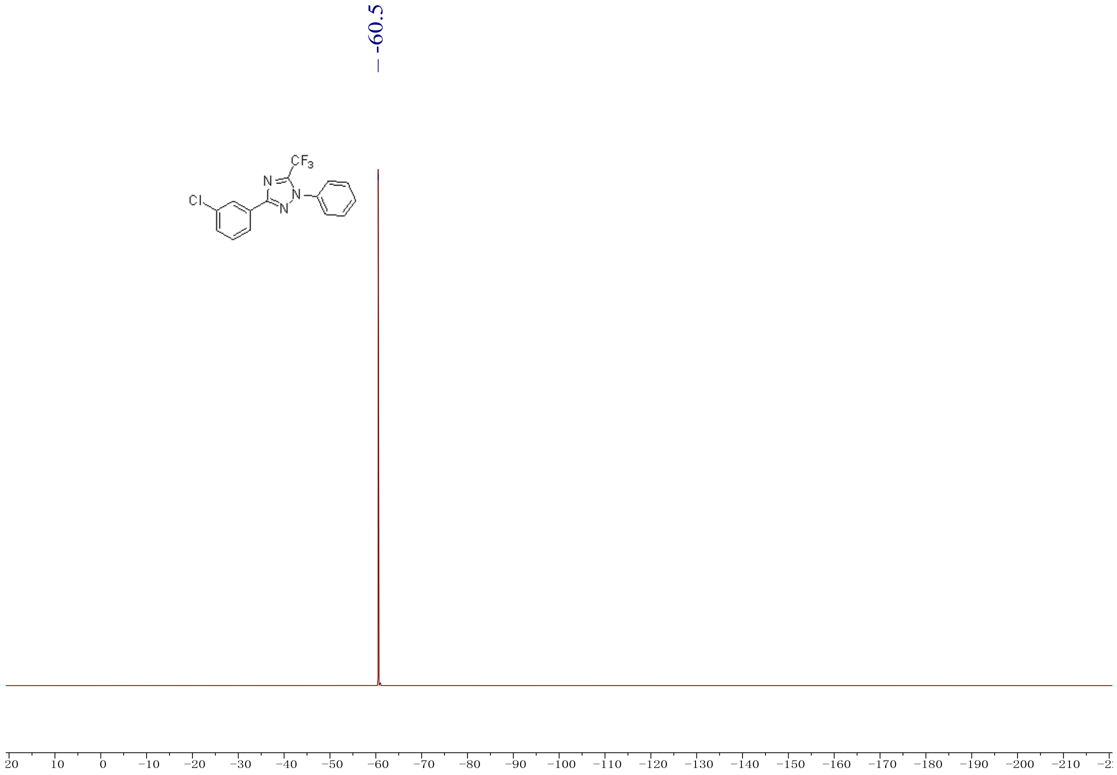

^{1}H NMR spectra of 3l (400 MHz, $CDCl_3$)

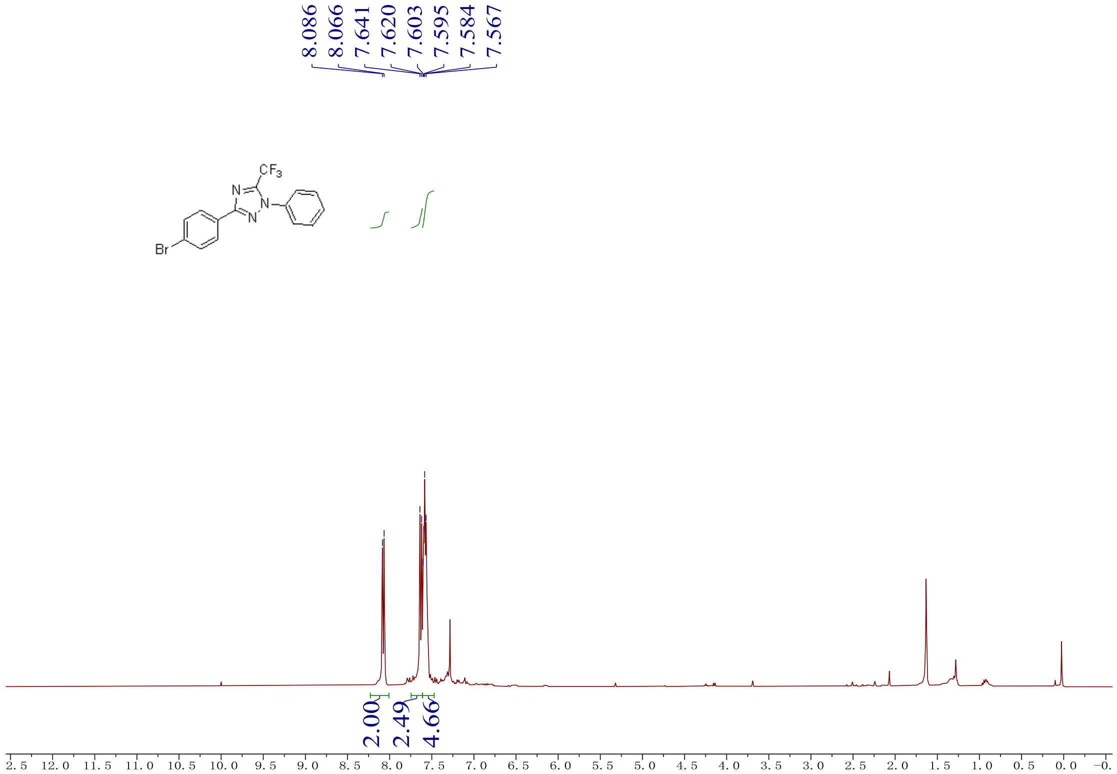

^{13}C{^{1}H} NMR spectra of 3l (101 MHz, CDCl$_3$)

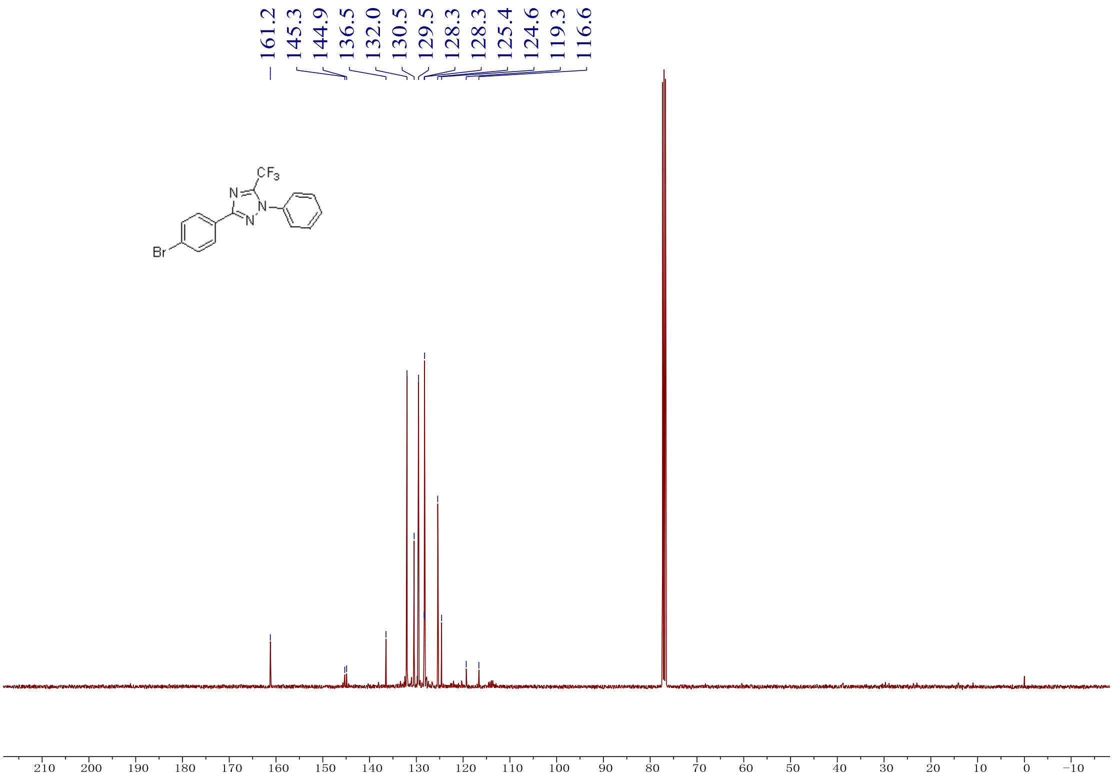

^{19}F NMR spectra of 3l (376 MHz, CDCl$_3$)

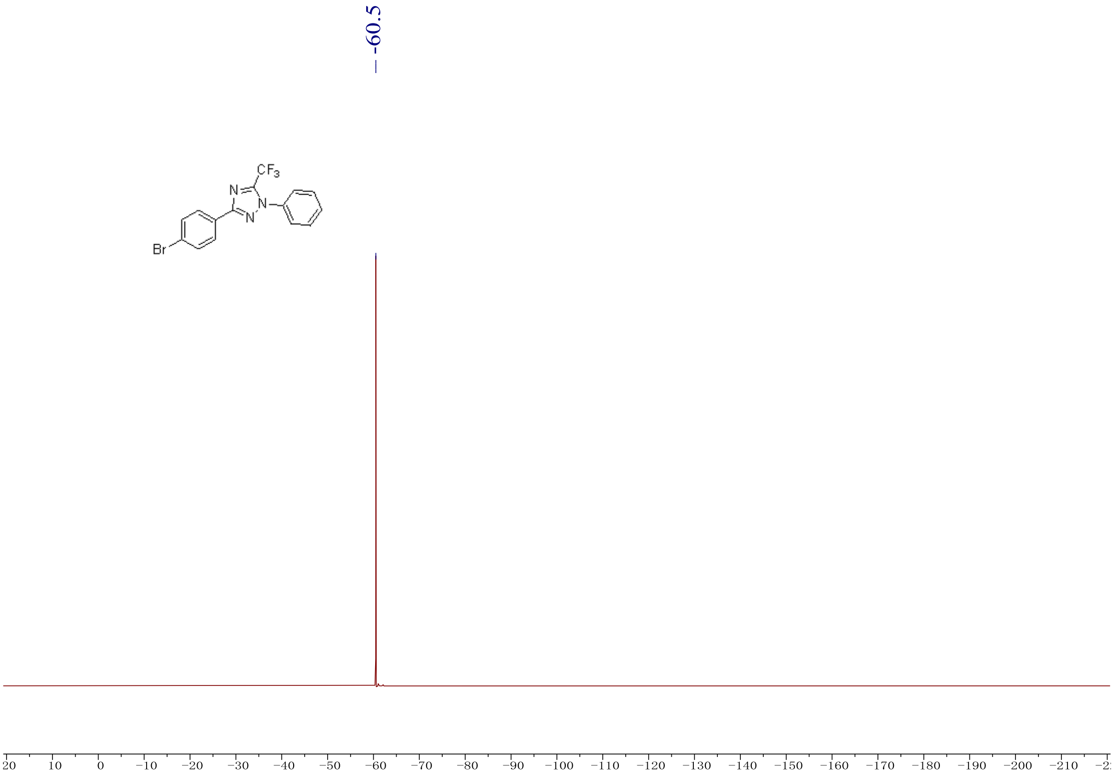

^{1}H NMR spectra of 3m (400 MHz, $CDCl_3$)

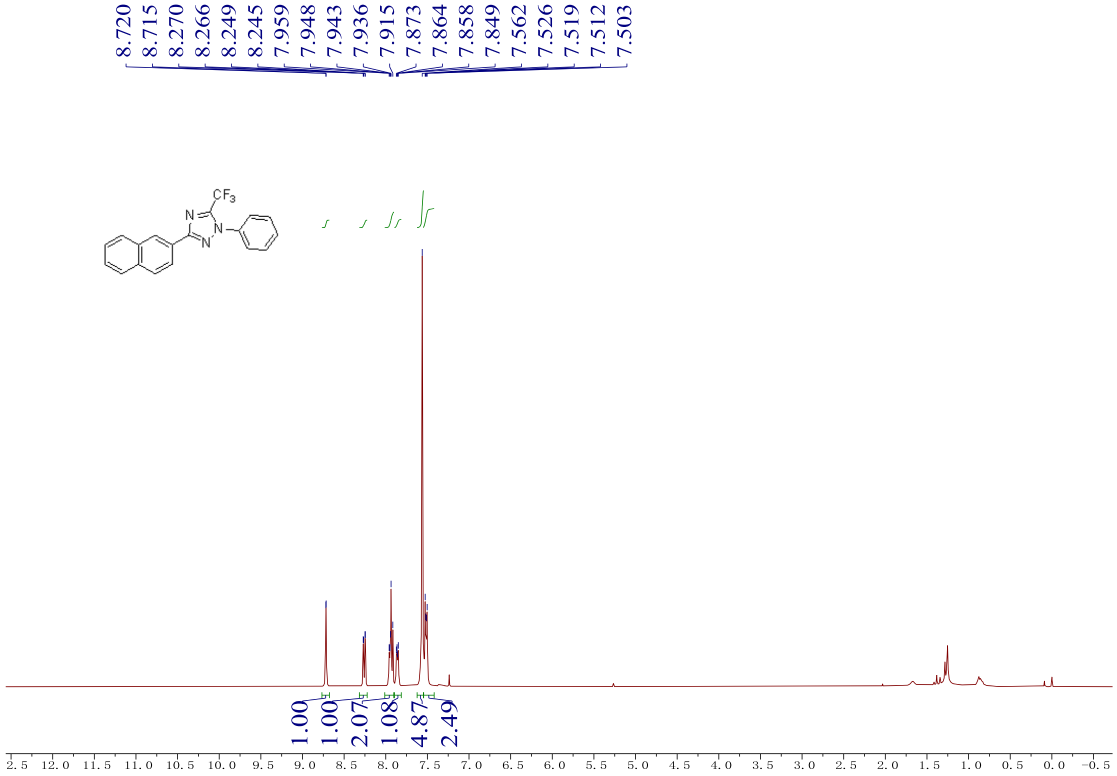

$^{13}C\{^{1}H\}$ NMR spectra of 3m(101 MHz, $CDCl_3$)

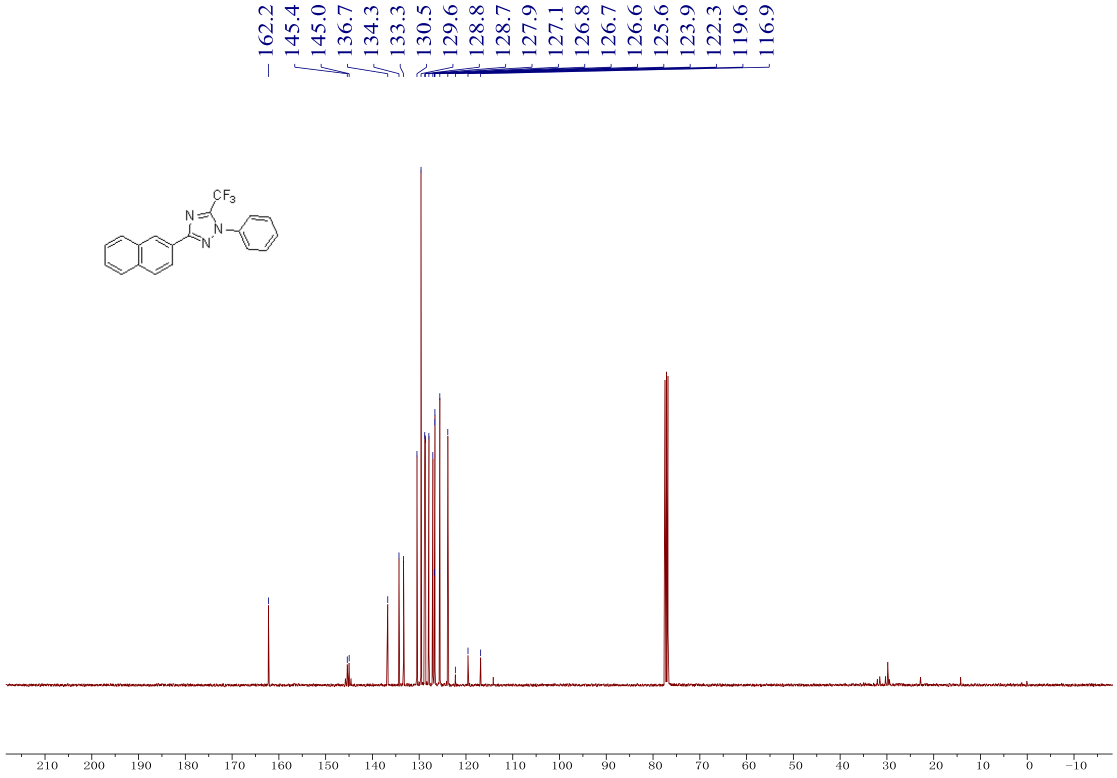

^{19}F NMR spectra of 3m (376 MHz, $CDCl_3$)

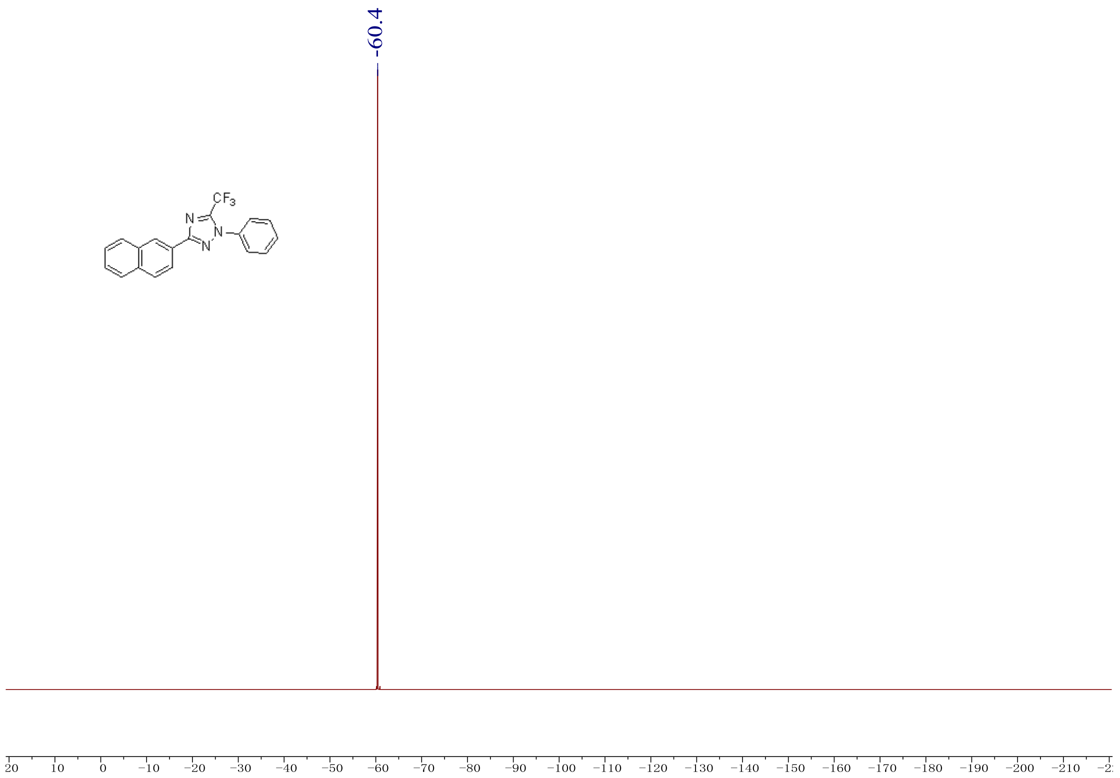

^{1}H NMR spectra of 3n (400 MHz, $CDCl_3$)

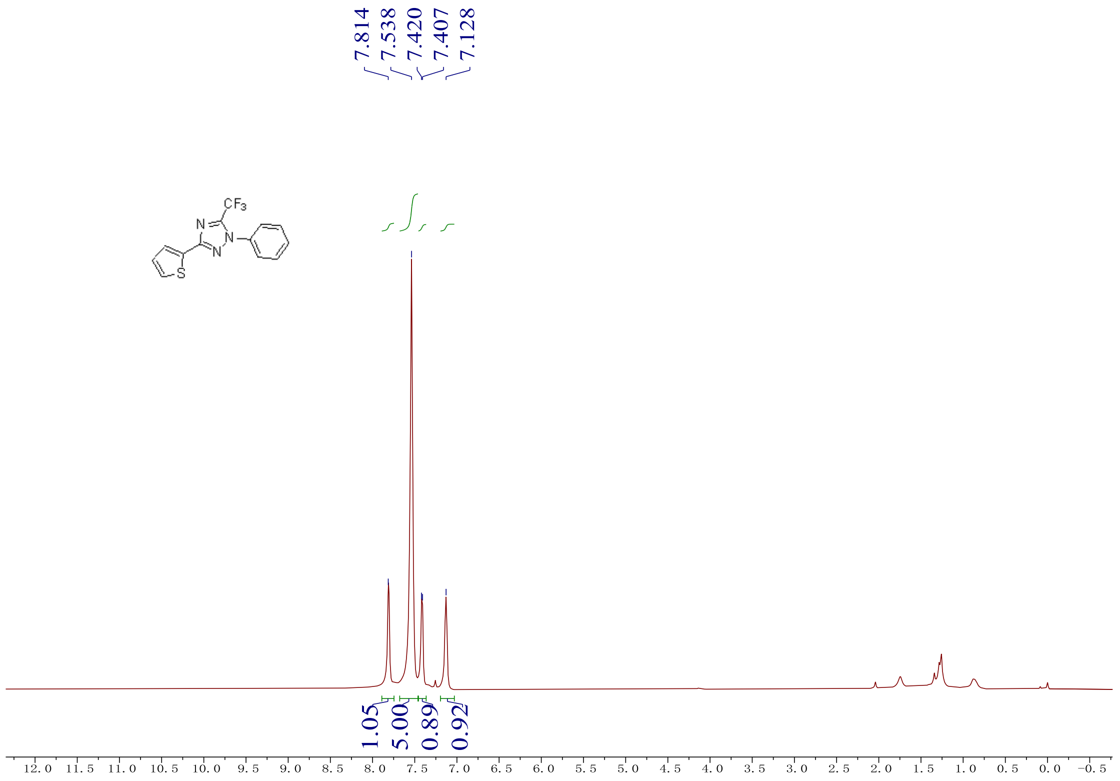

$^{13}C\{^{1}H\}$ NMR spectra of 3n (101 MHz, $CDCl_3$)

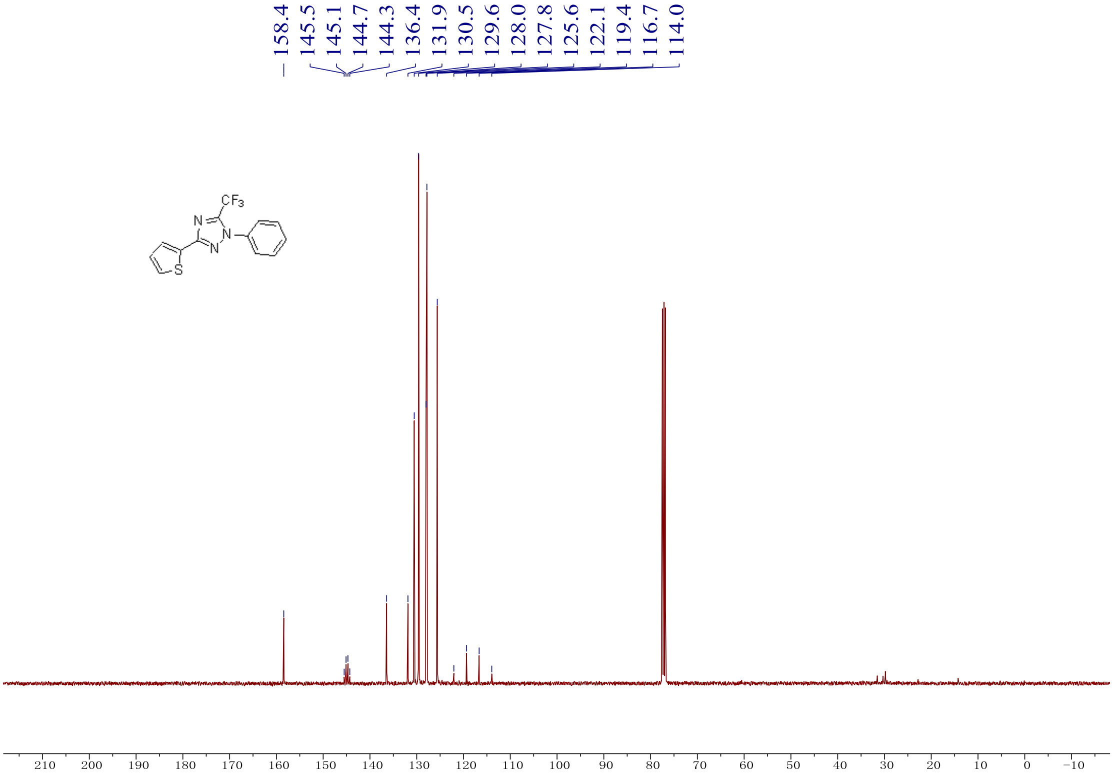

^{19}F NMR spectra of 3n (376 MHz, $CDCl_3$)

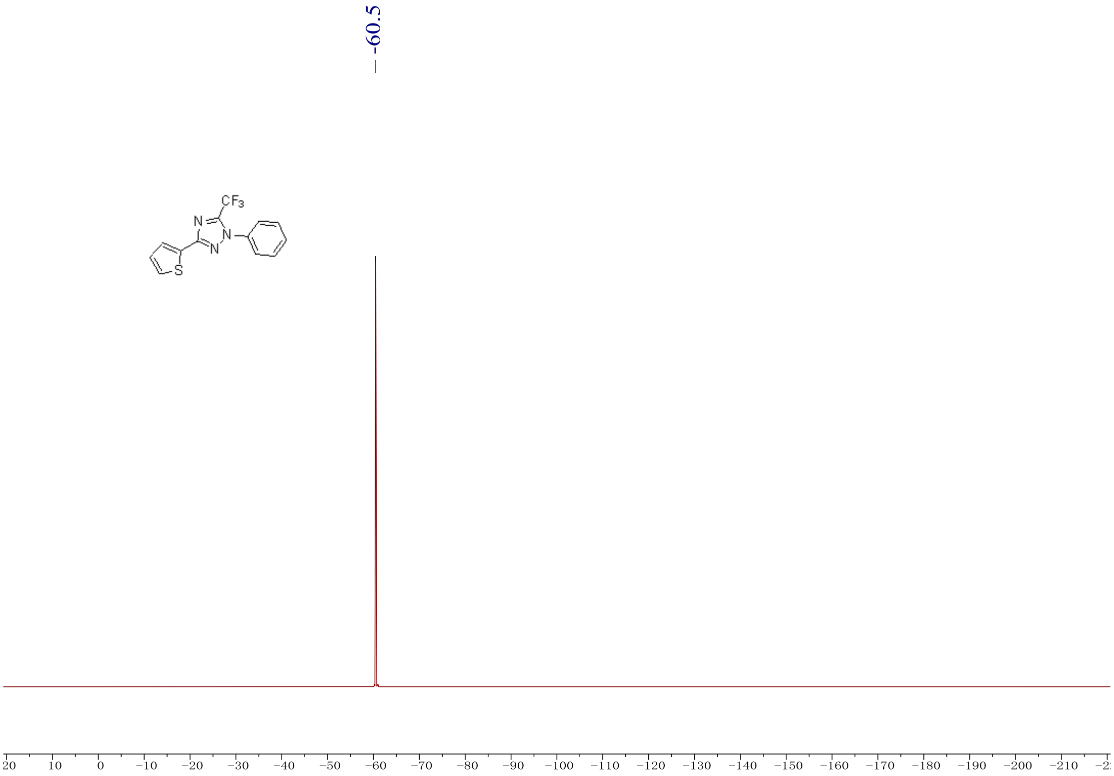

^{1}H NMR spectra of 3o (400 MHz, $CDCl_3$)

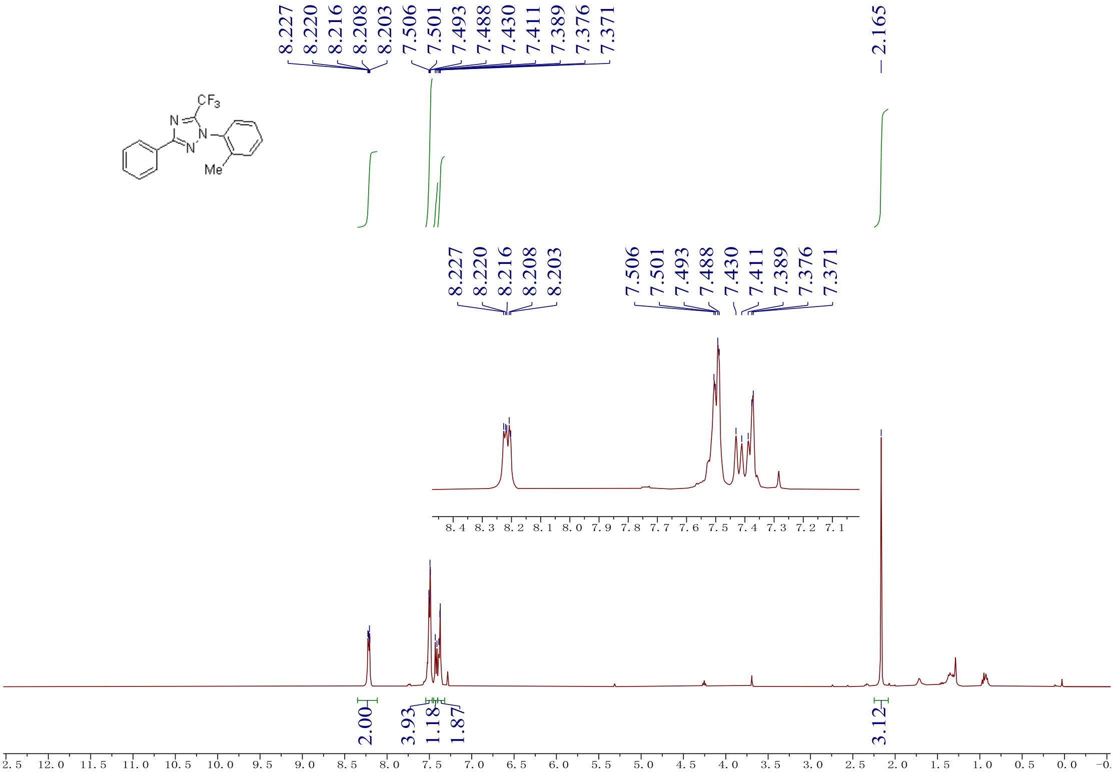

$^{13}C\{^{1}H\}$ NMR spectra of 3o (101 MHz, $CDCl_3$)

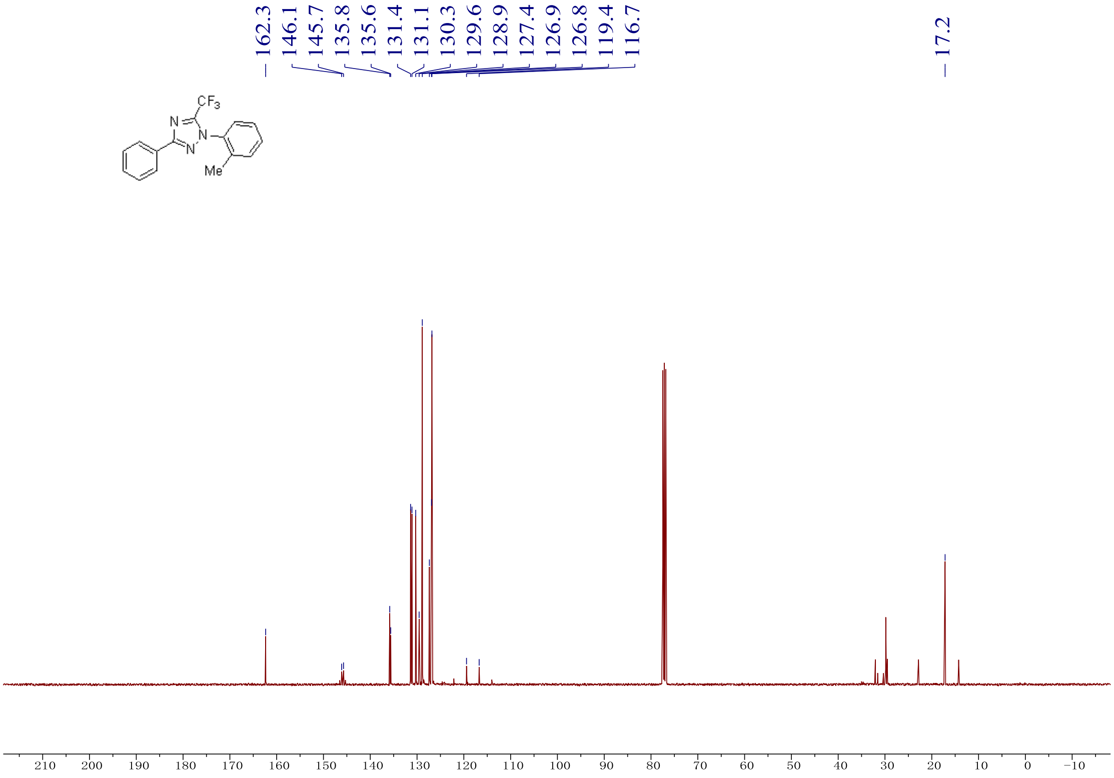

^{19}F NMR spectra of 3o (376 MHz, $CDCl_3$)

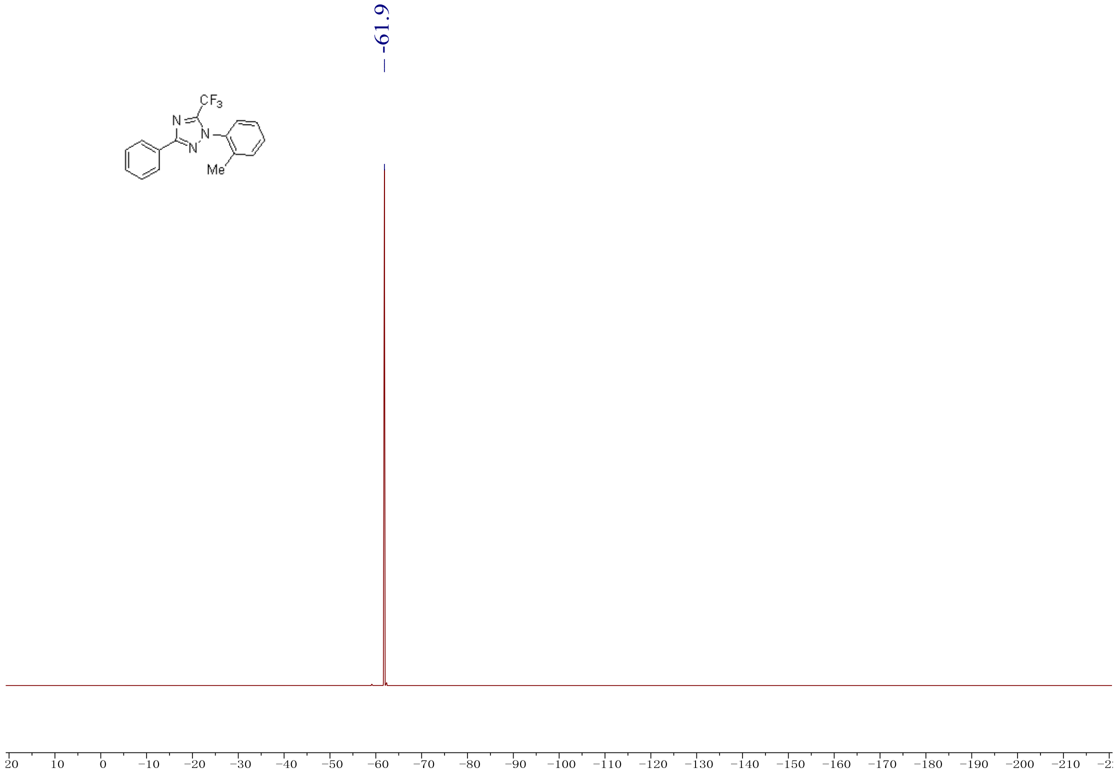

^{1}H NMR spectra of 3p (400 MHz, $CDCl_3$)

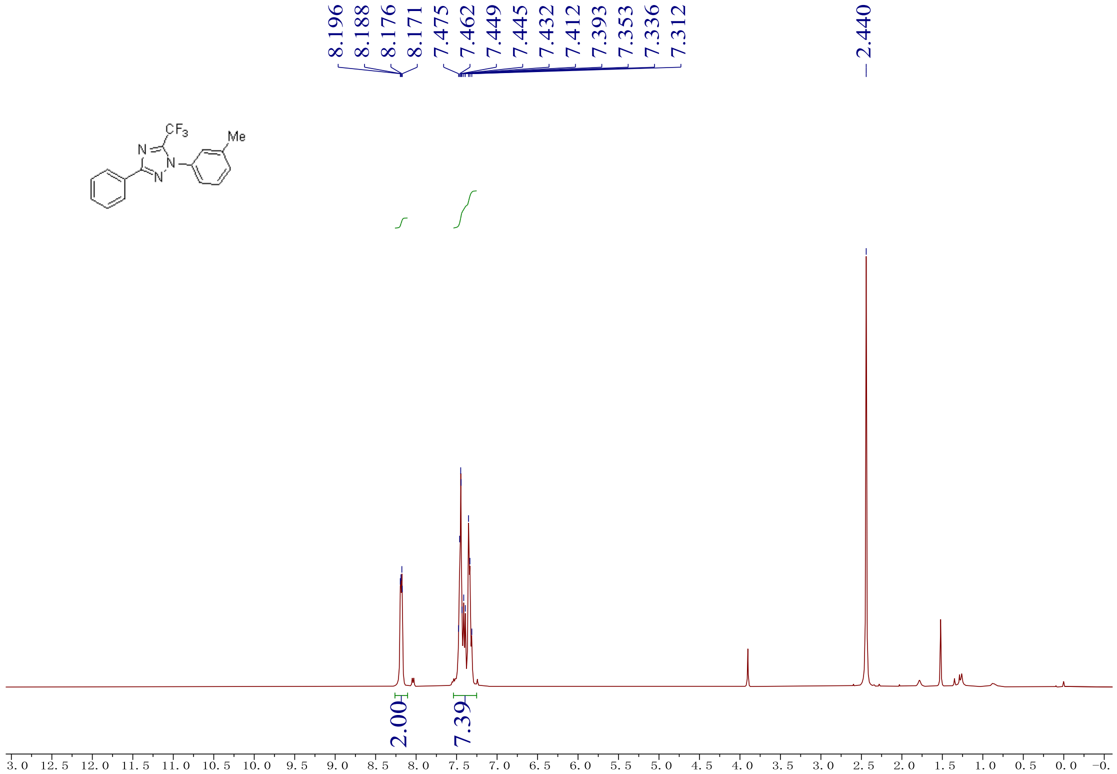

$^{13}C\{^{1}H\}$ NMR spectra of 3p (101 MHz, $CDCl_3$)

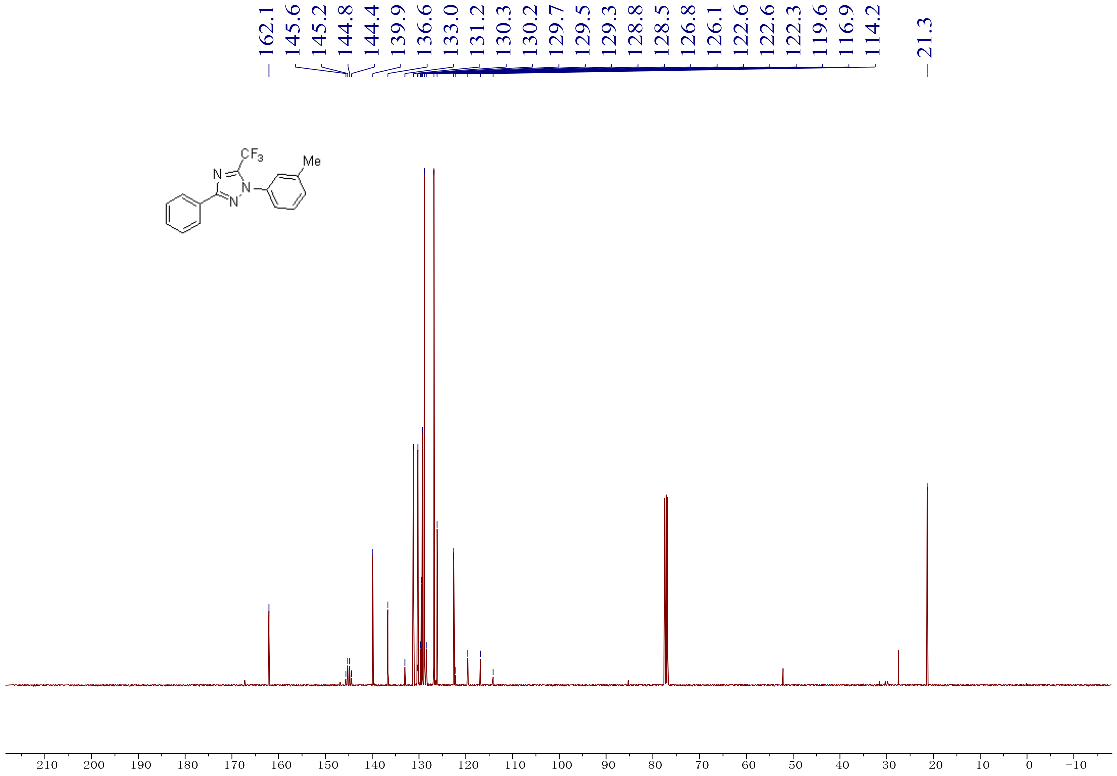

^{19}F NMR spectra of 3p (376 MHz, $CDCl_3$)

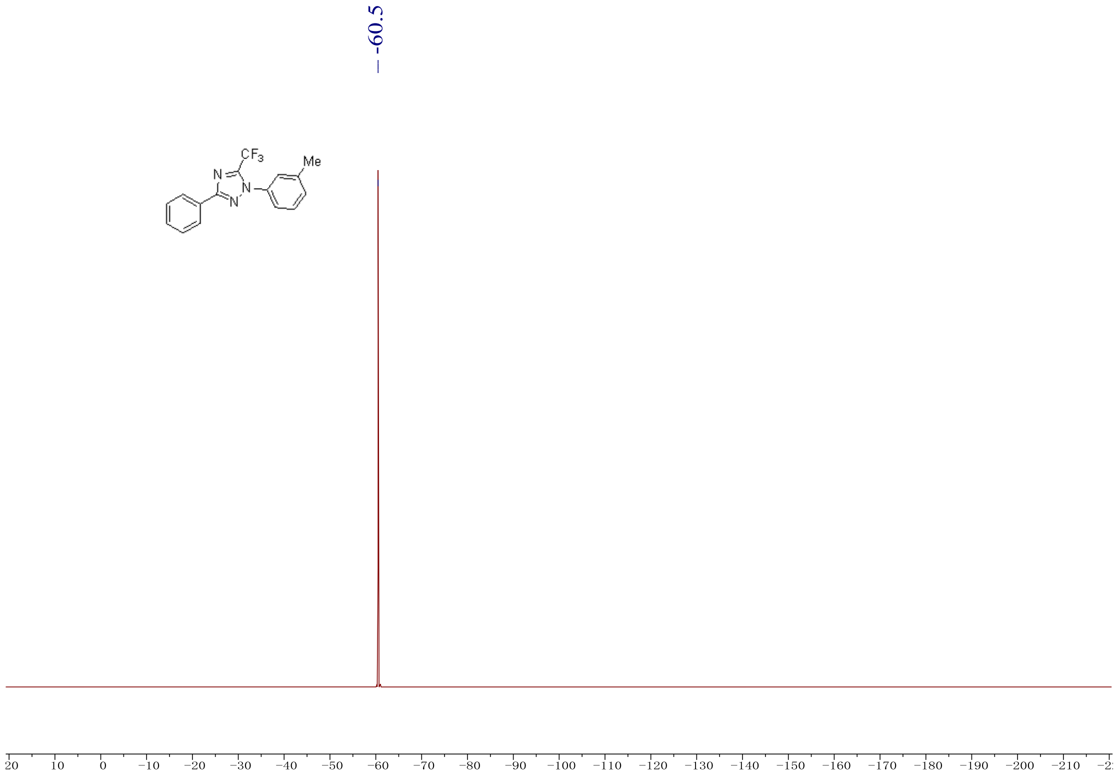

^{1}H NMR spectra of 3q (400 MHz, $CDCl_3$)

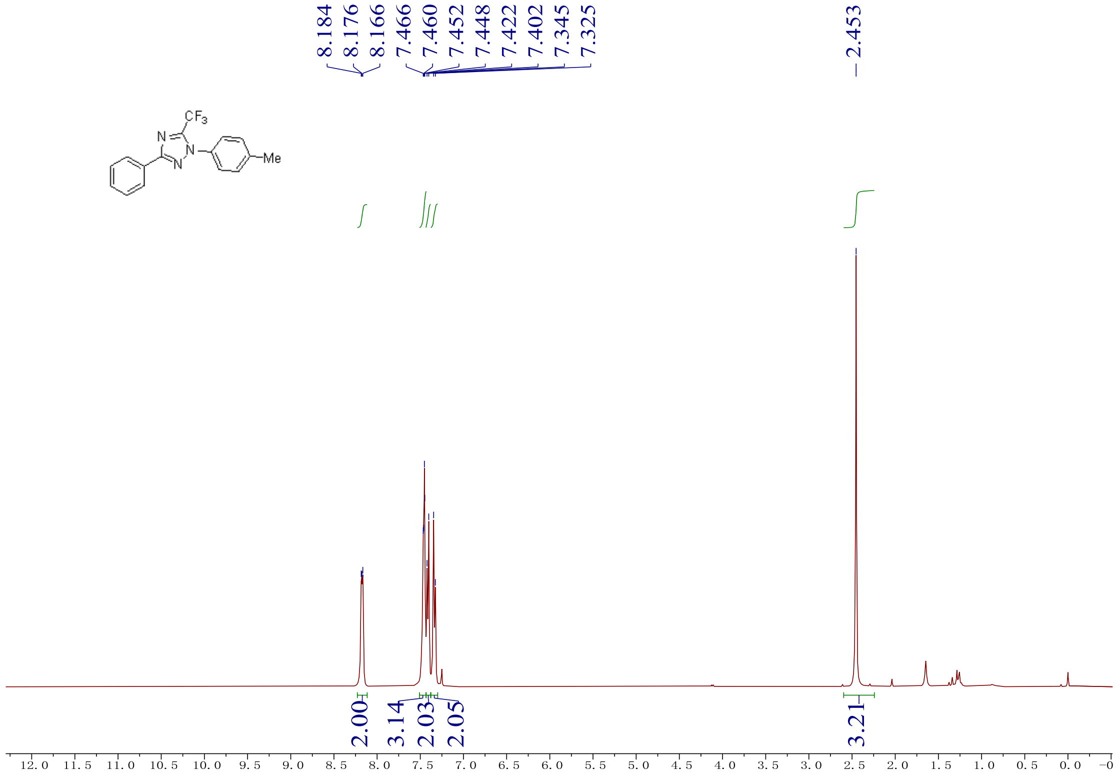

^{13}C{^{1}H} NMR spectra of 3q (101 MHz, $CDCl_3$)

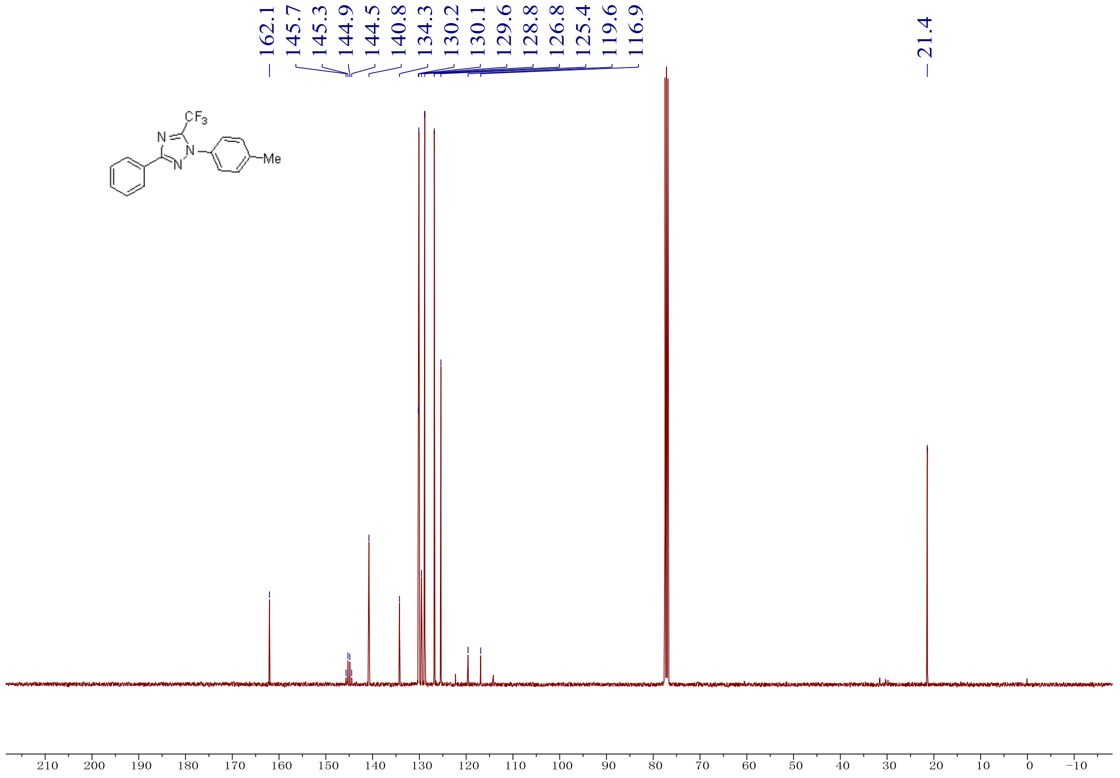

^{19}F NMR spectra of 3q (376 MHz, $CDCl_3$)

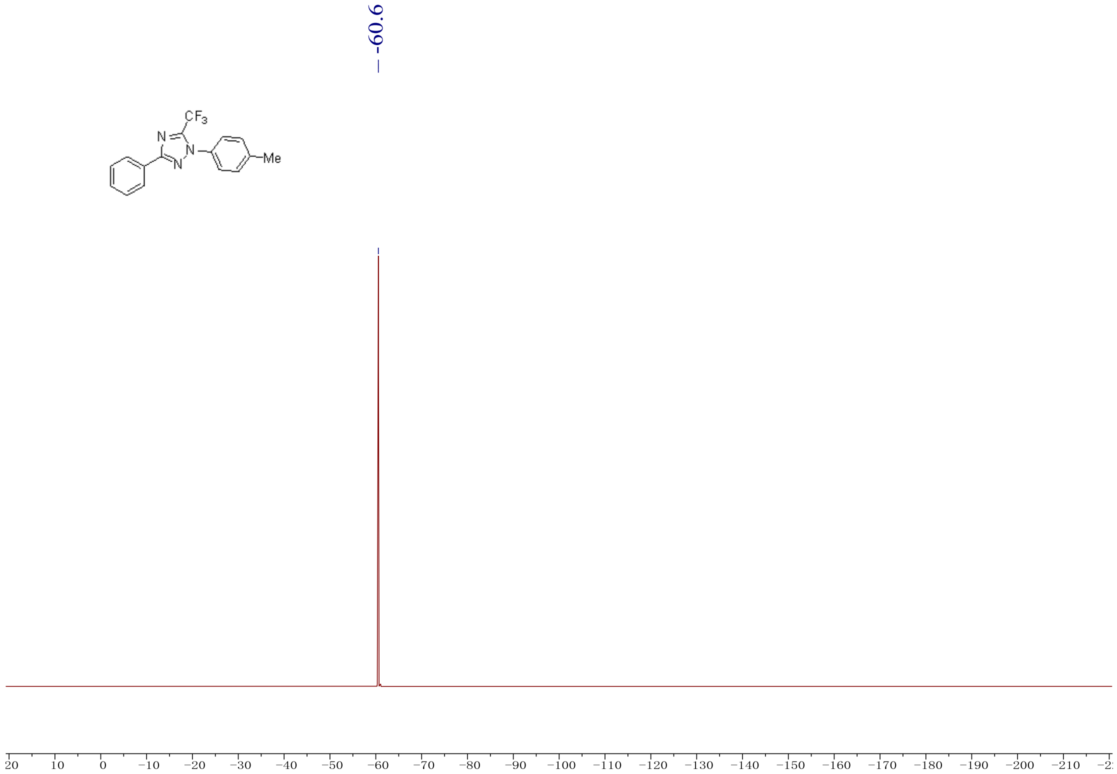

^{1}H NMR spectra of 3r (400 MHz, $CDCl_3$)

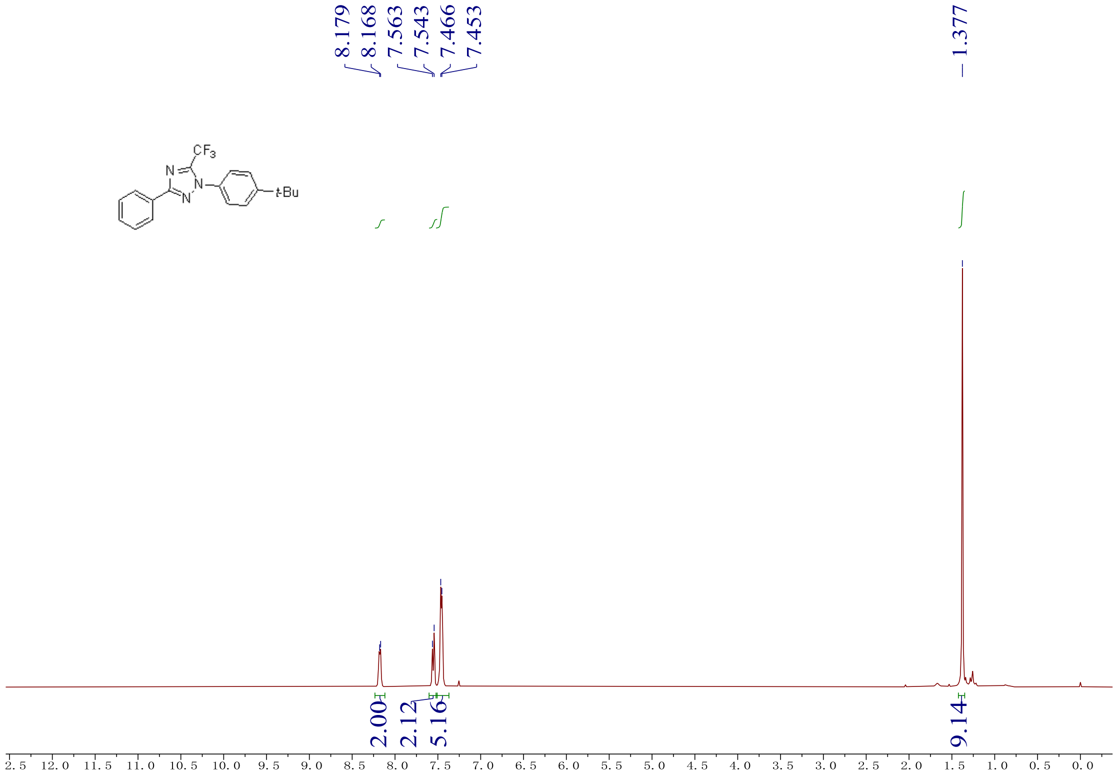

$^{13}C\{^{1}H\}$ NMR spectra of 3r (101 MHz, $CDCl_3$)

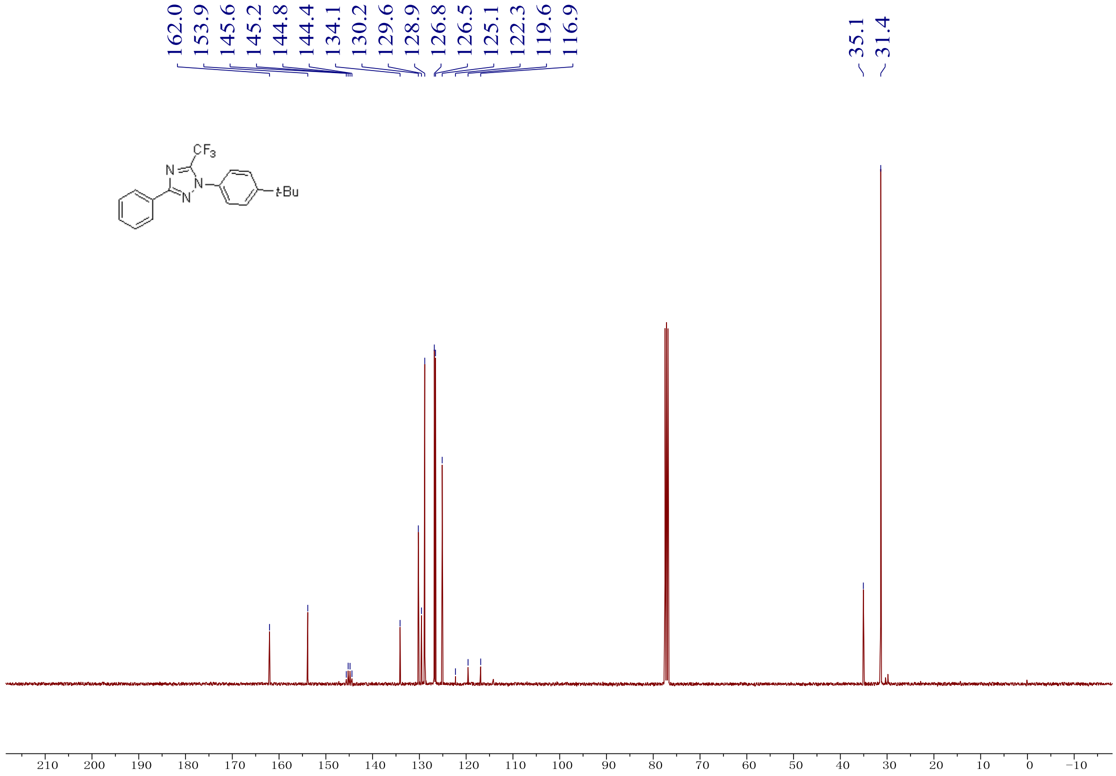

^{19}F NMR spectra of 3r (376 MHz, $CDCl_3$)

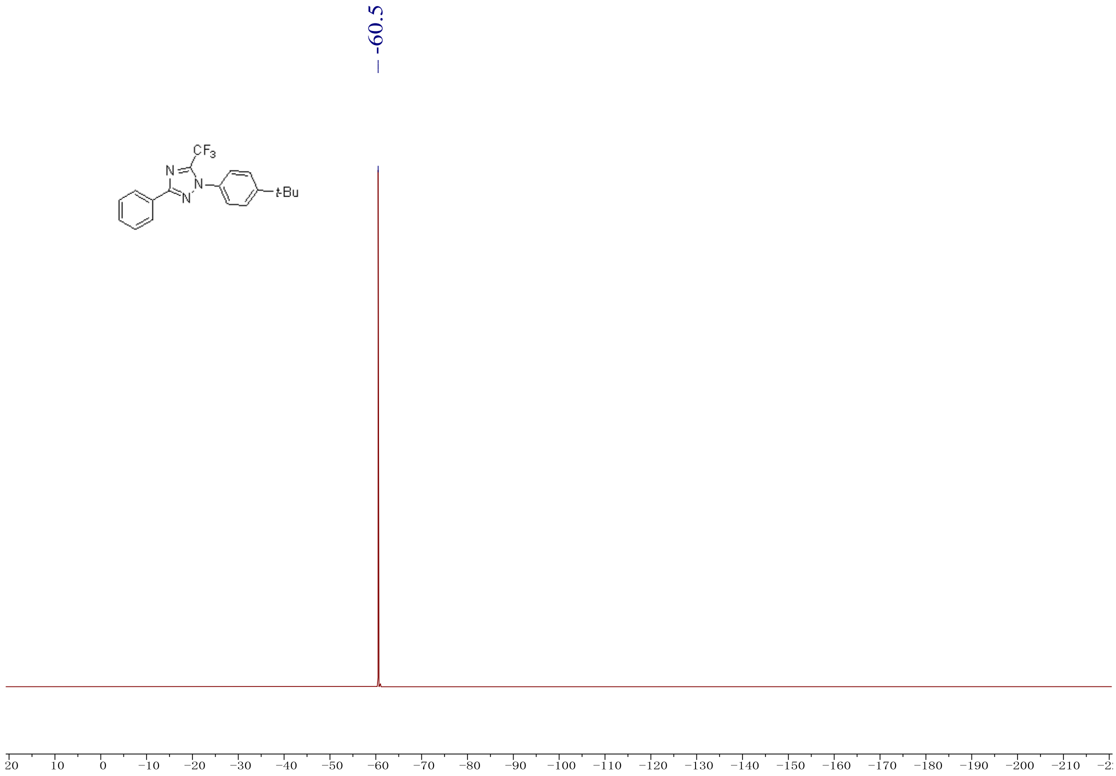

^{1}H NMR spectra of 3s (400 MHz, $CDCl_3$)

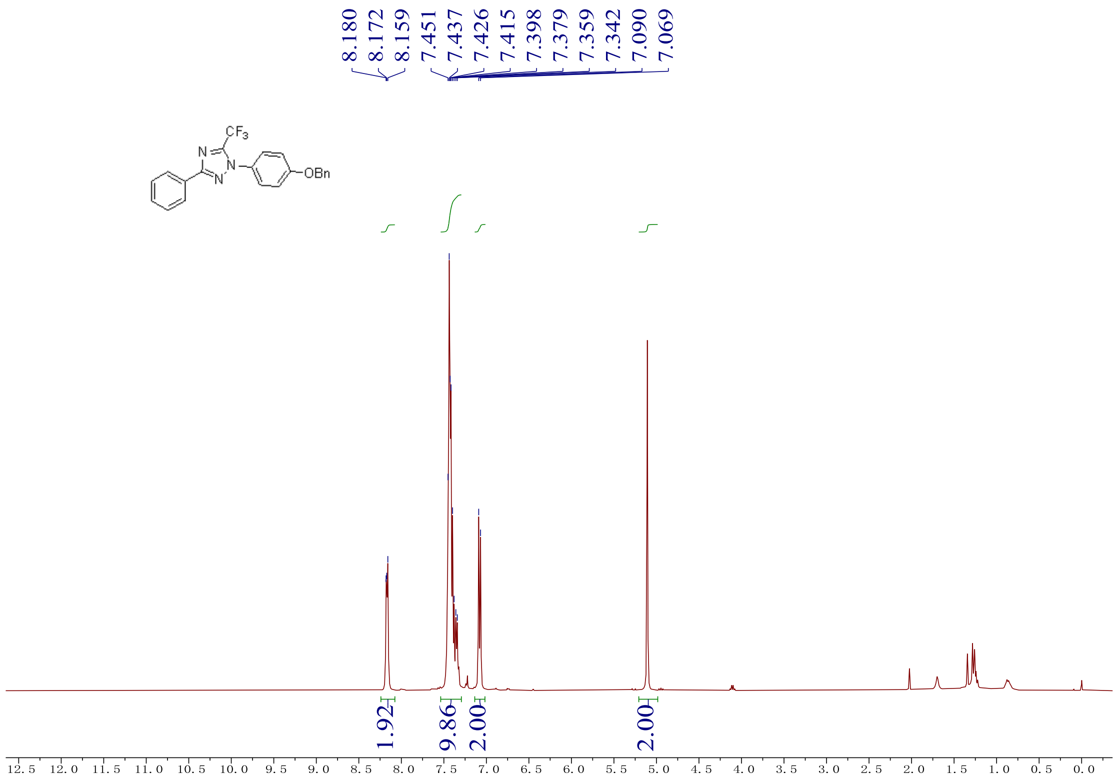

$^{13}C\{^{1}H\}$ NMR spectra of 3s (101 MHz, $CDCl_3$)

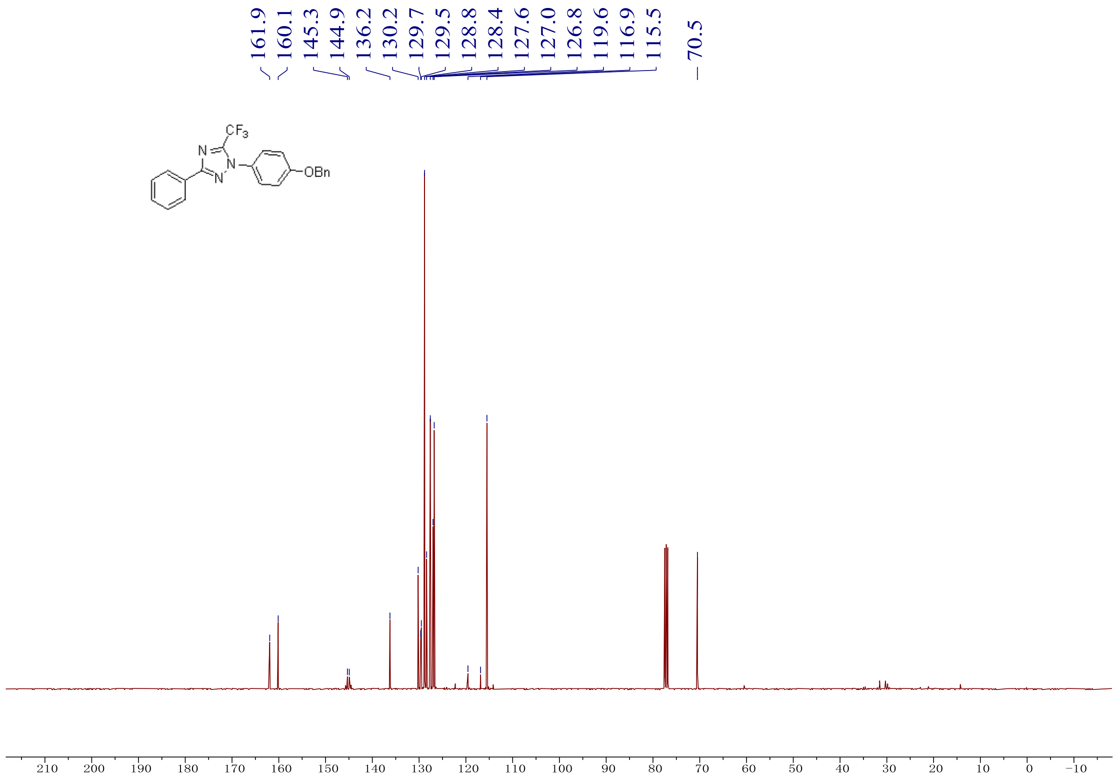

^{19}F NMR spectra of 3s (376 MHz, $CDCl_3$)

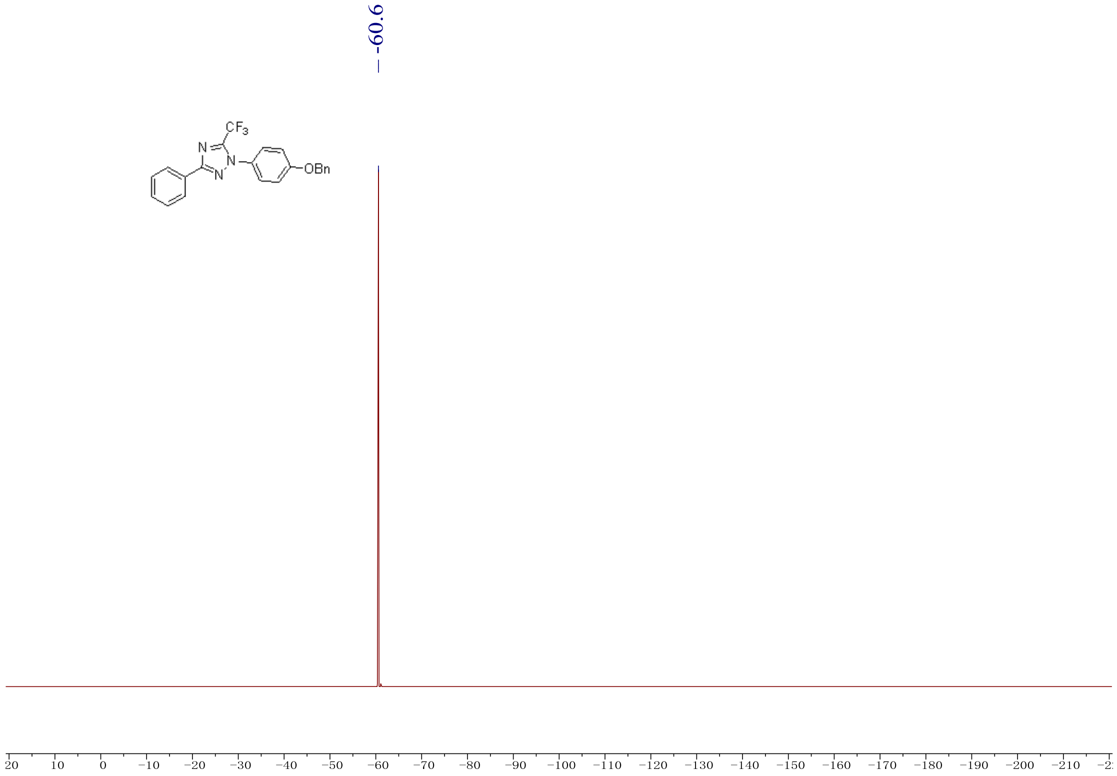

^{1}H NMR spectra of 3t (400 MHz, $CDCl_3$)

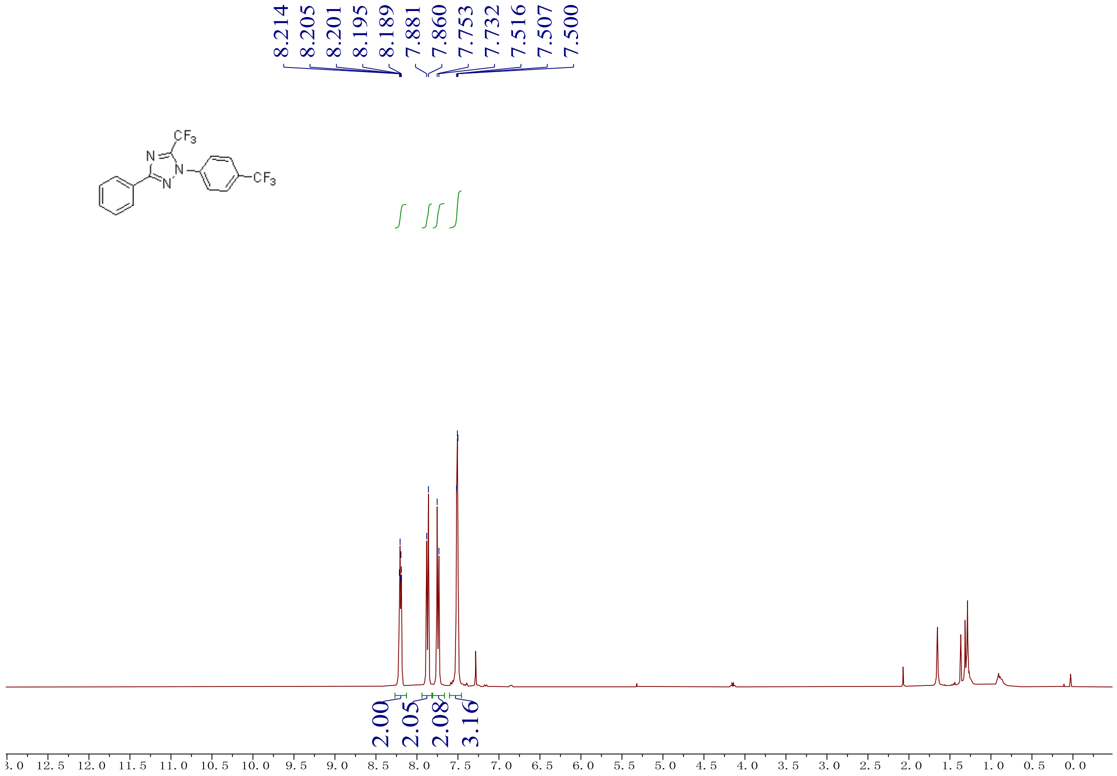

$^{13}C\{^{1}H\}$ NMR spectra of 3t (101 MHz, $CDCl_3$)

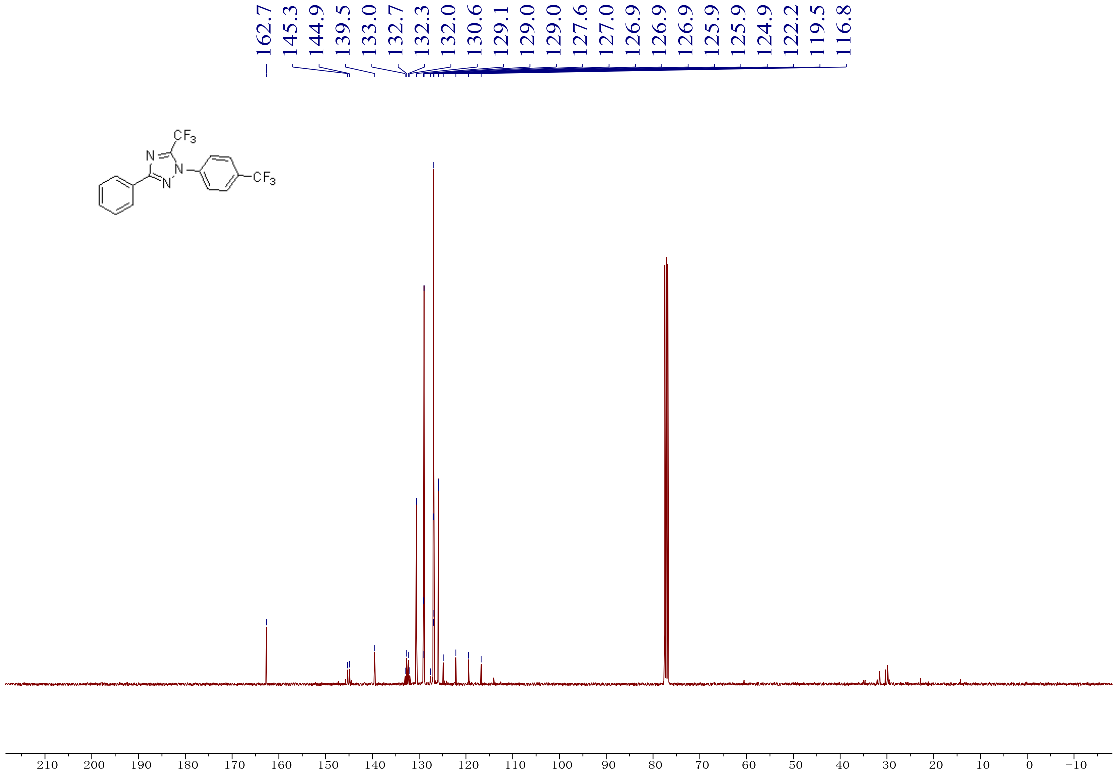

^{19}F NMR spectra of 3t (376 MHz, $CDCl_3$)

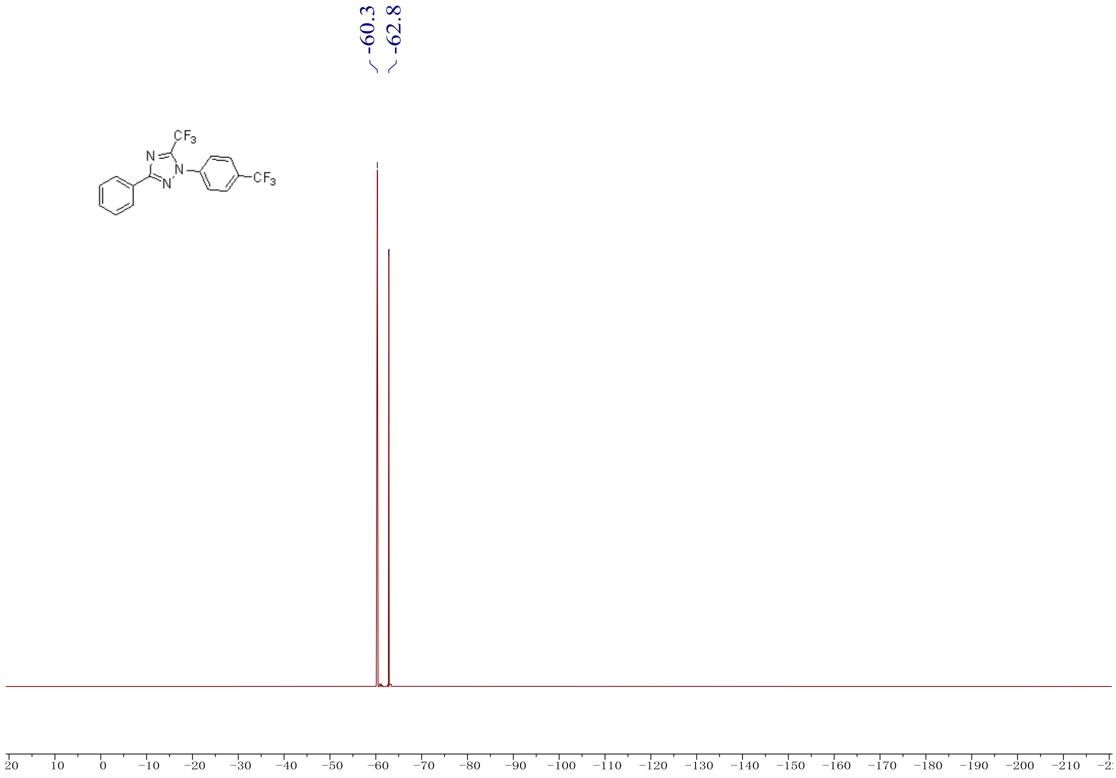

^{1}H NMR spectra of 3u (400 MHz, $CDCl_3$)

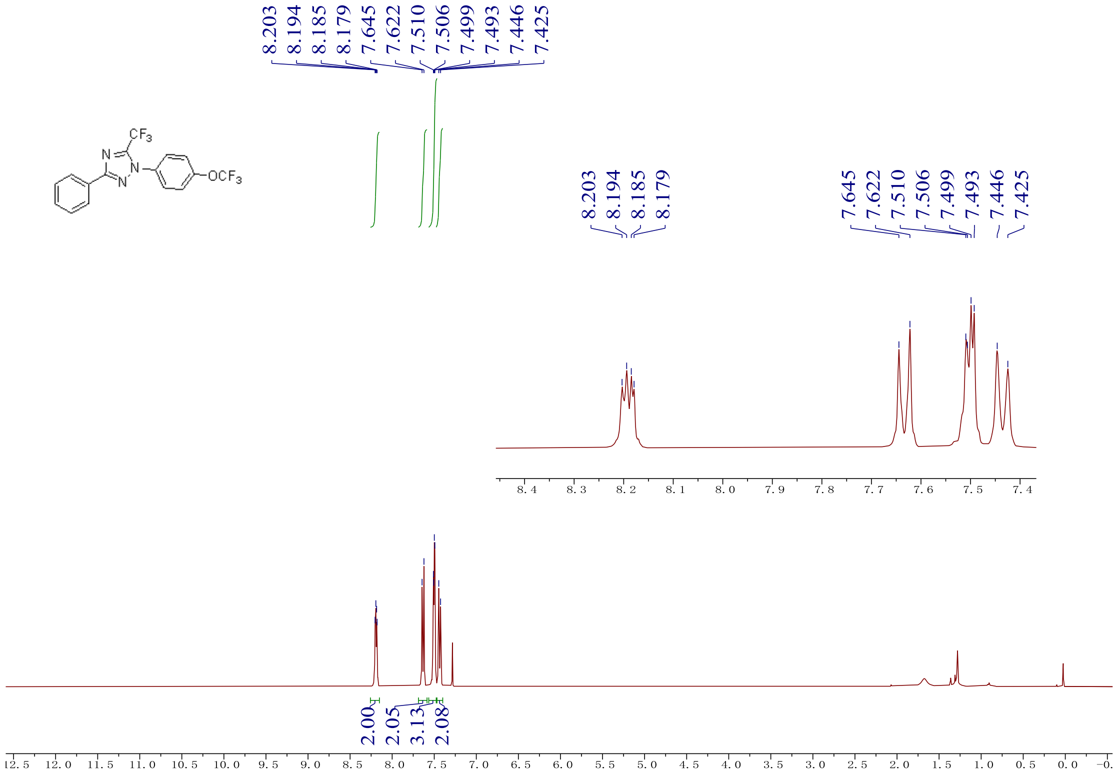

^{13}C{^{1}H} NMR spectra of 3u (101 MHz, $CDCl_3$)

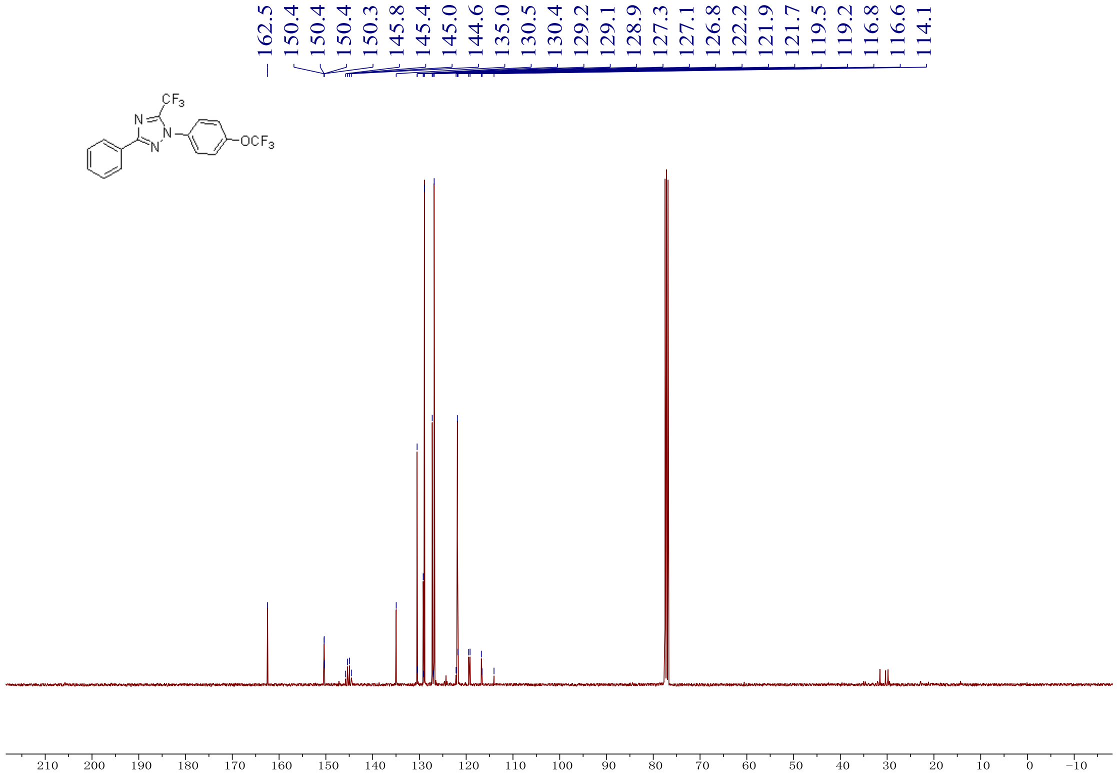

^{19}F NMR spectra of 3u (376 MHz, $CDCl_3$)

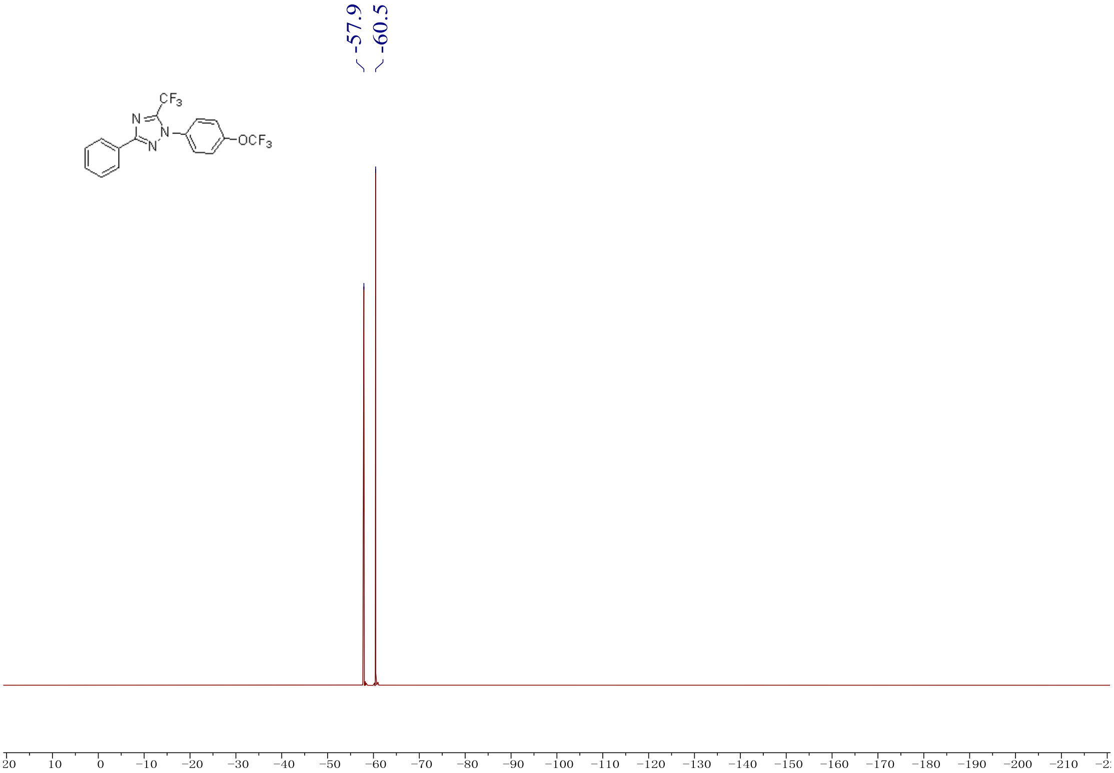

^{1}H NMR spectra of 3v (400 MHz, $CDCl_3$)

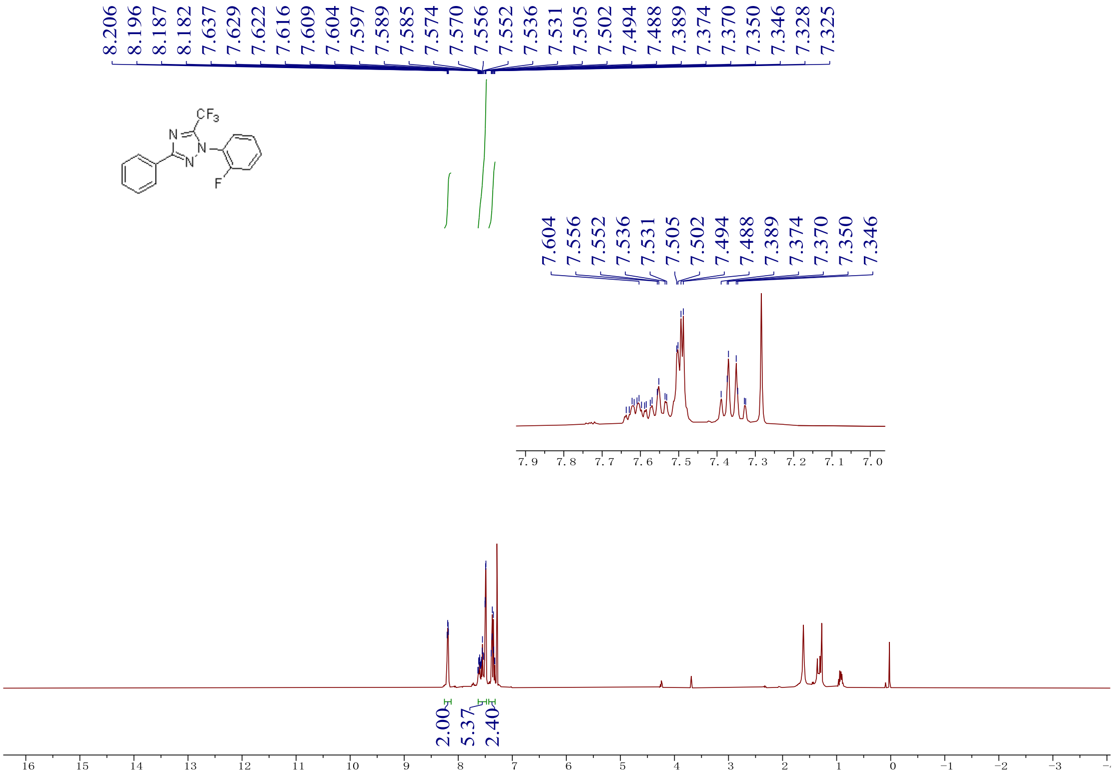

$^{13}C\{^{1}H\}$ NMR spectra of 3v (101 MHz, $CDCl_3$)

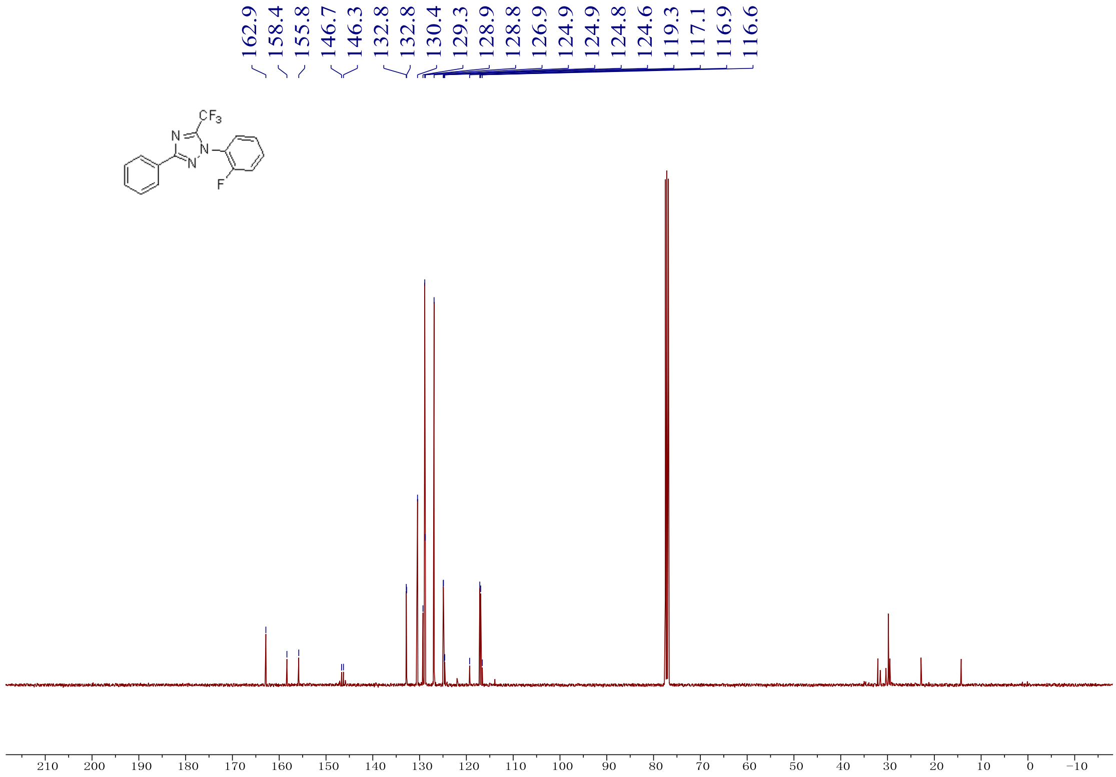

^{19}F NMR spectra of 3v (376 MHz, $CDCl_3$)

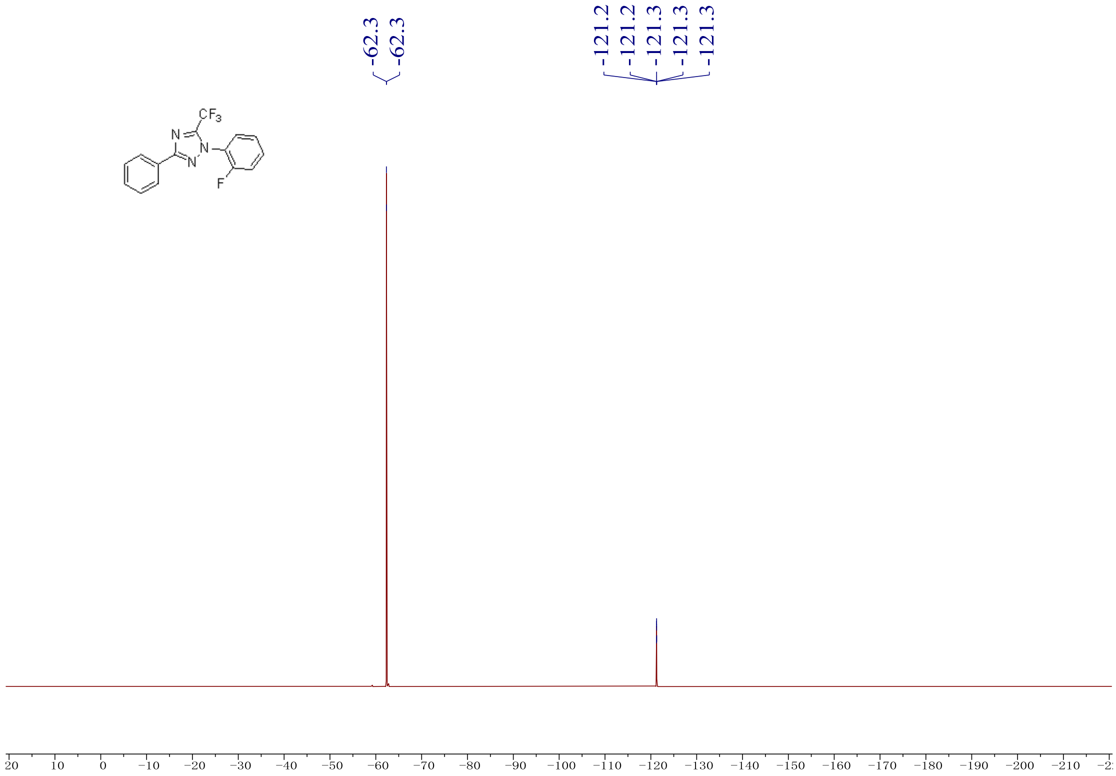

^{1}H NMR spectra of 3w (400 MHz, $CDCl_3$)

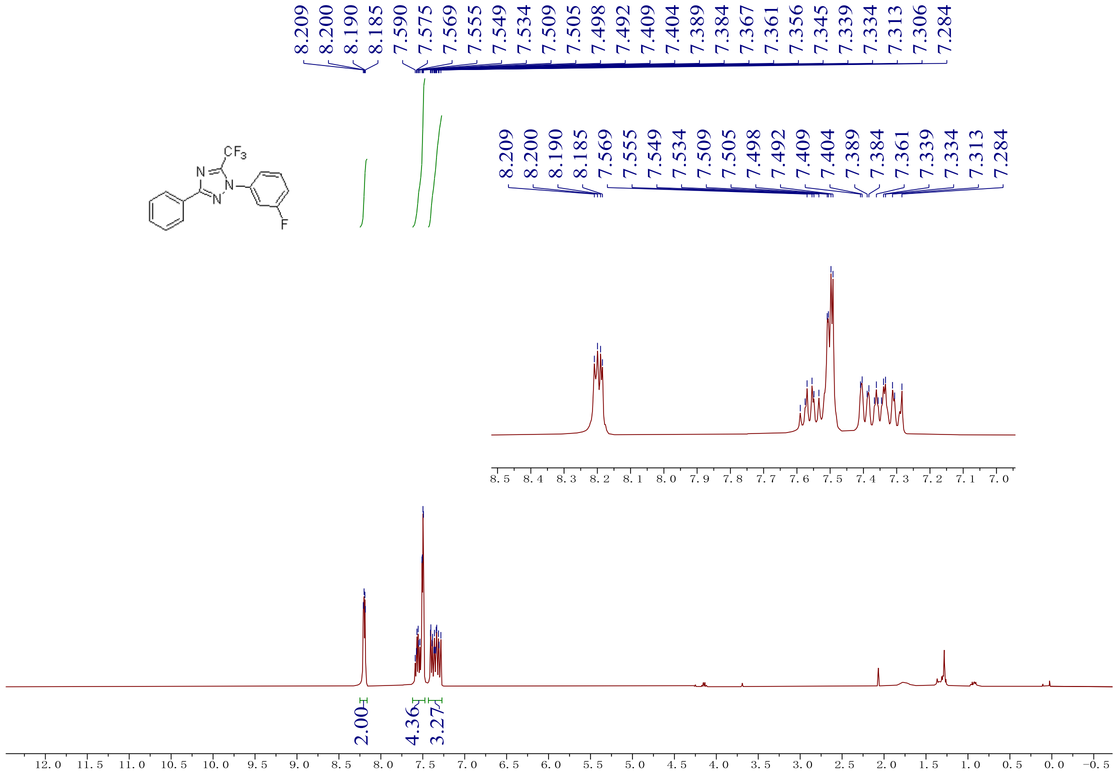

$^{13}C\{^{1}H\}$ NMR spectra of 3w (101 MHz, $CDCl_3$)

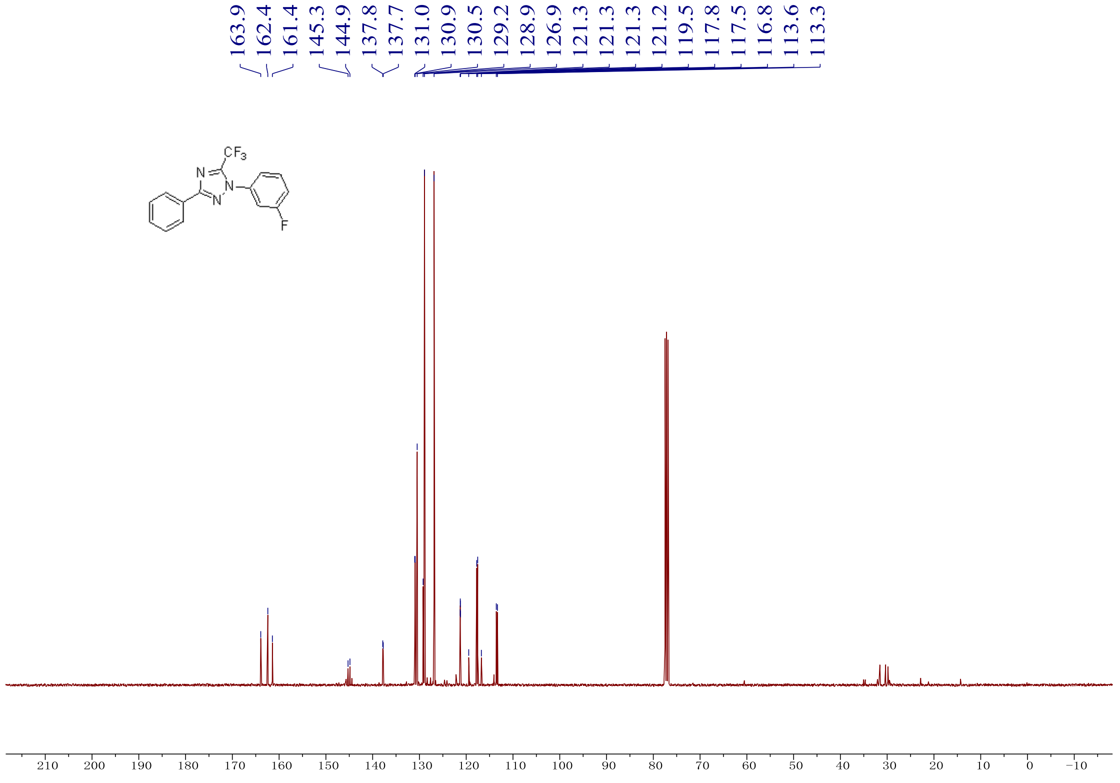

^{19}F NMR spectra of 3w (376 MHz, $CDCl_3$)

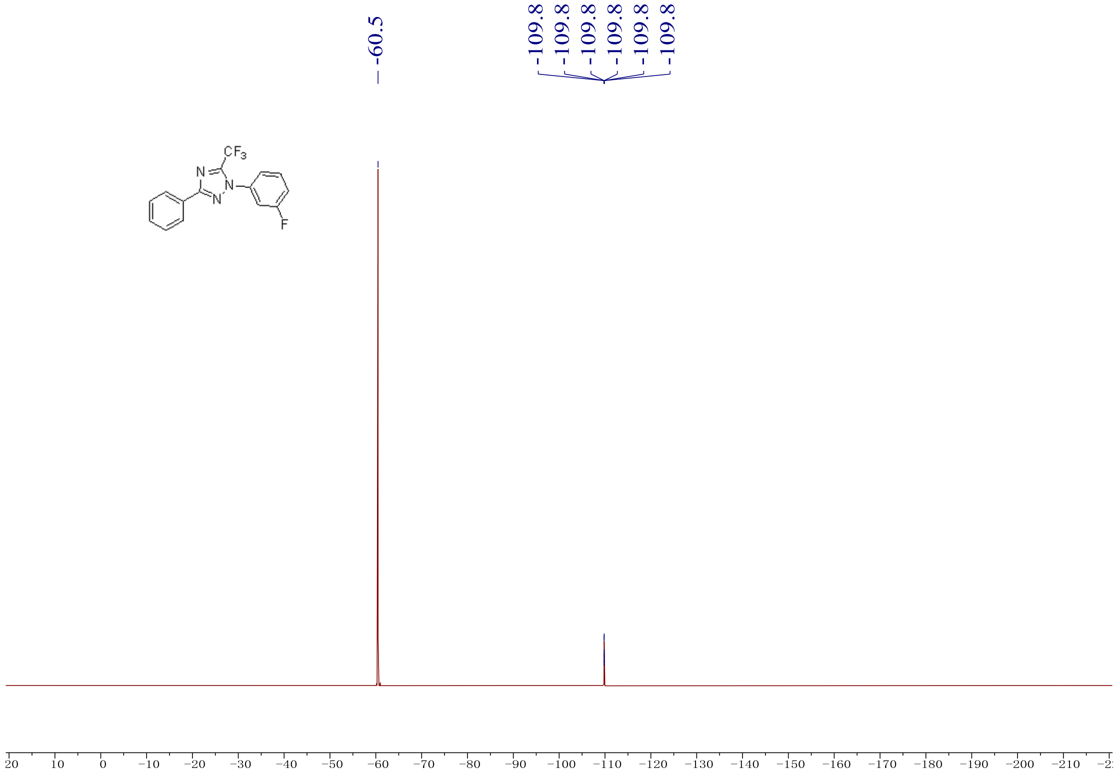

^{1}H NMR spectra of 3x (400 MHz, $CDCl_3$)

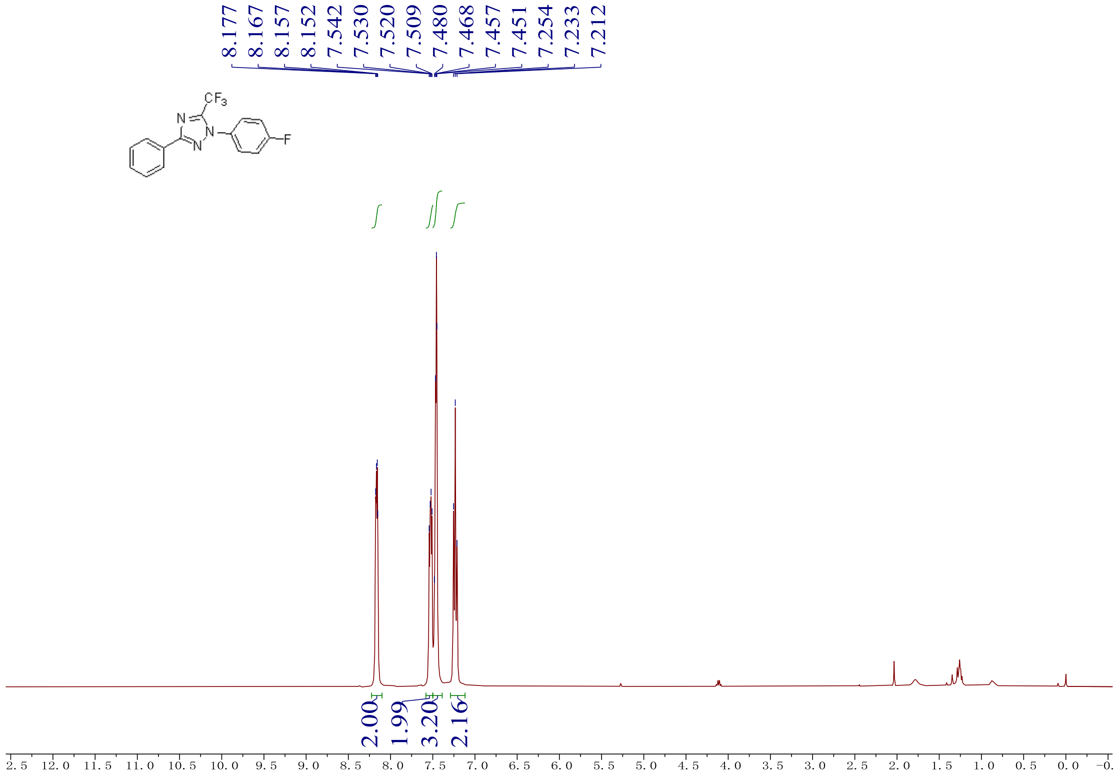

$^{13}C\{^{1}H\}$ NMR spectra of 3x (101 MHz, $CDCl_3$))

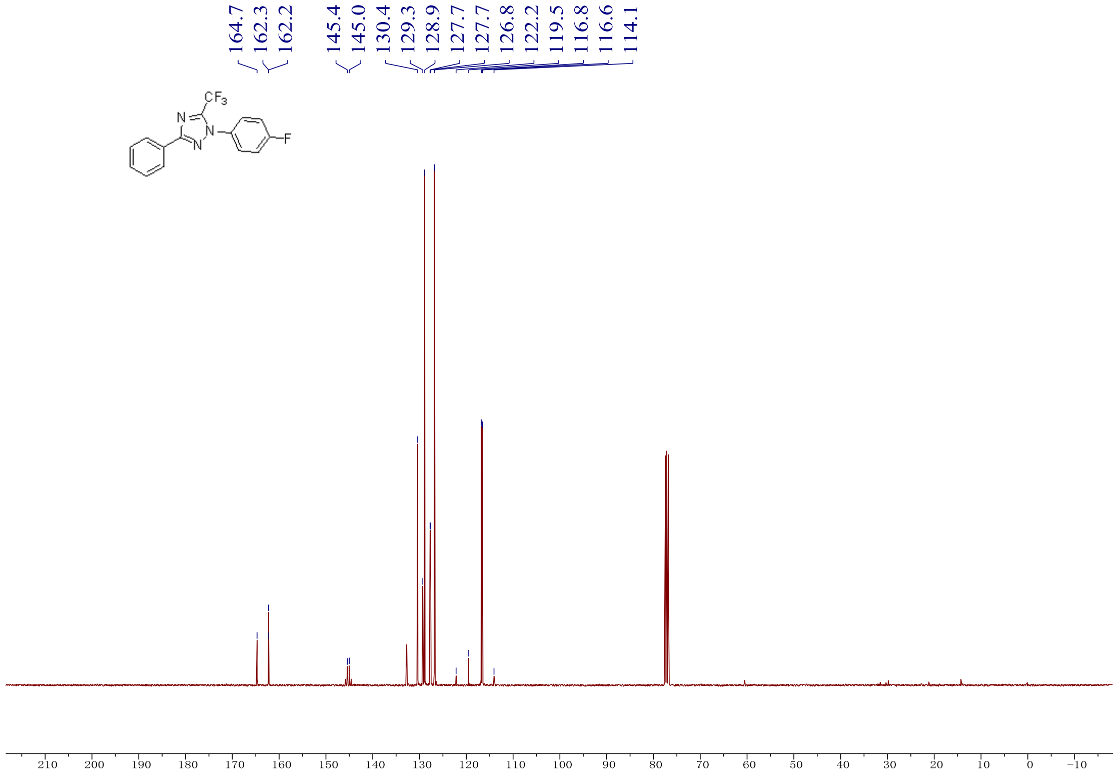

^{19}F NMR spectra of 3x (376 MHz, $CDCl_3$)

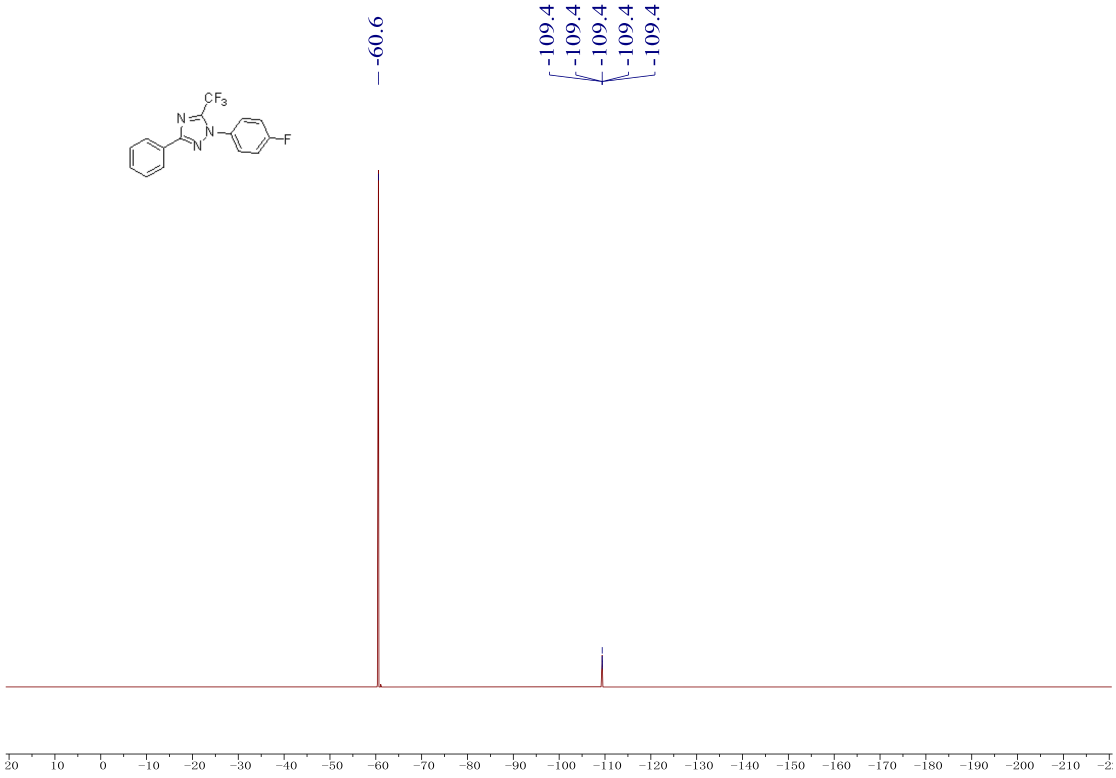

^{1}H NMR spectra of 3y (400 MHz, $CDCl_3$)

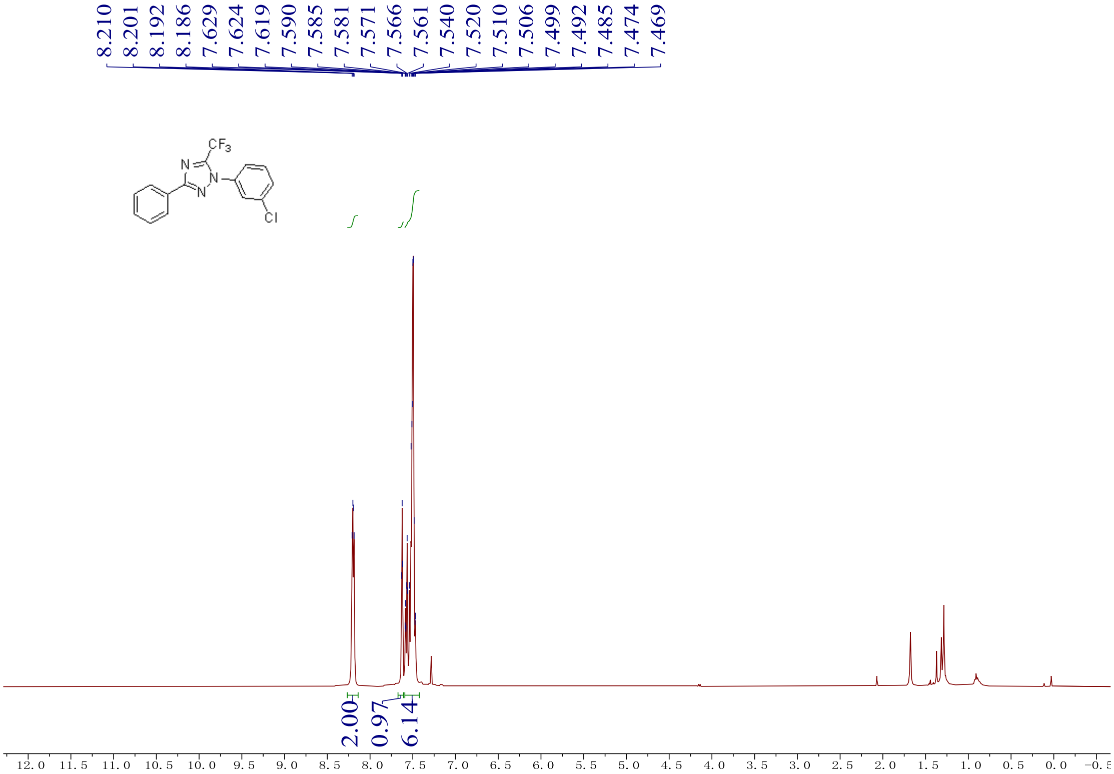

^{13}C{^{1}H} NMR spectra of 3y (101 MHz, $CDCl_3$)

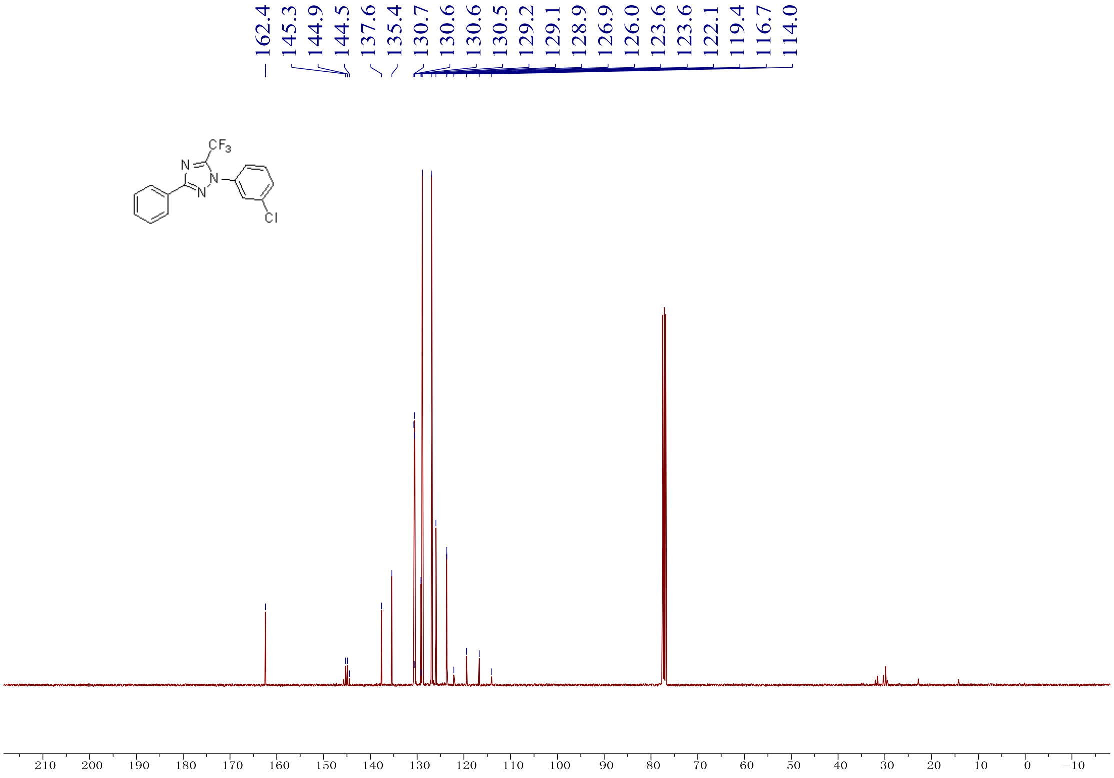

^{19}F NMR spectra of 3y (376 MHz, $CDCl_3$)

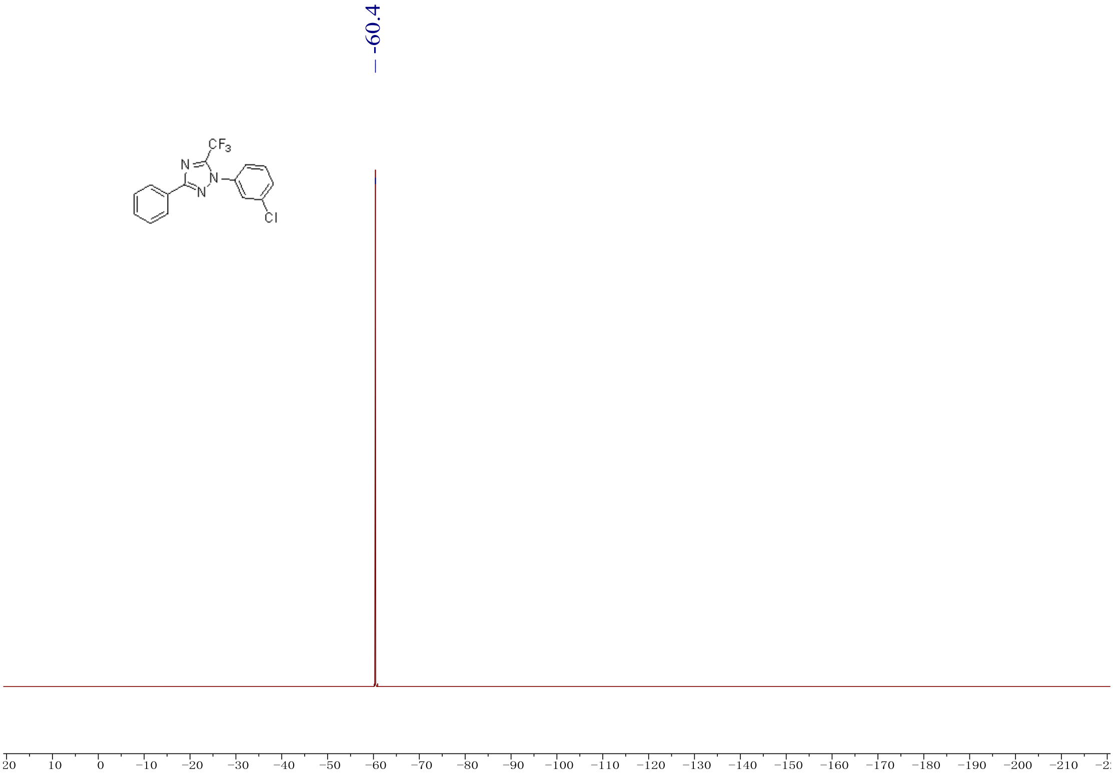

^{1}H NMR spectra of 3z (400 MHz, $CDCl_3$)

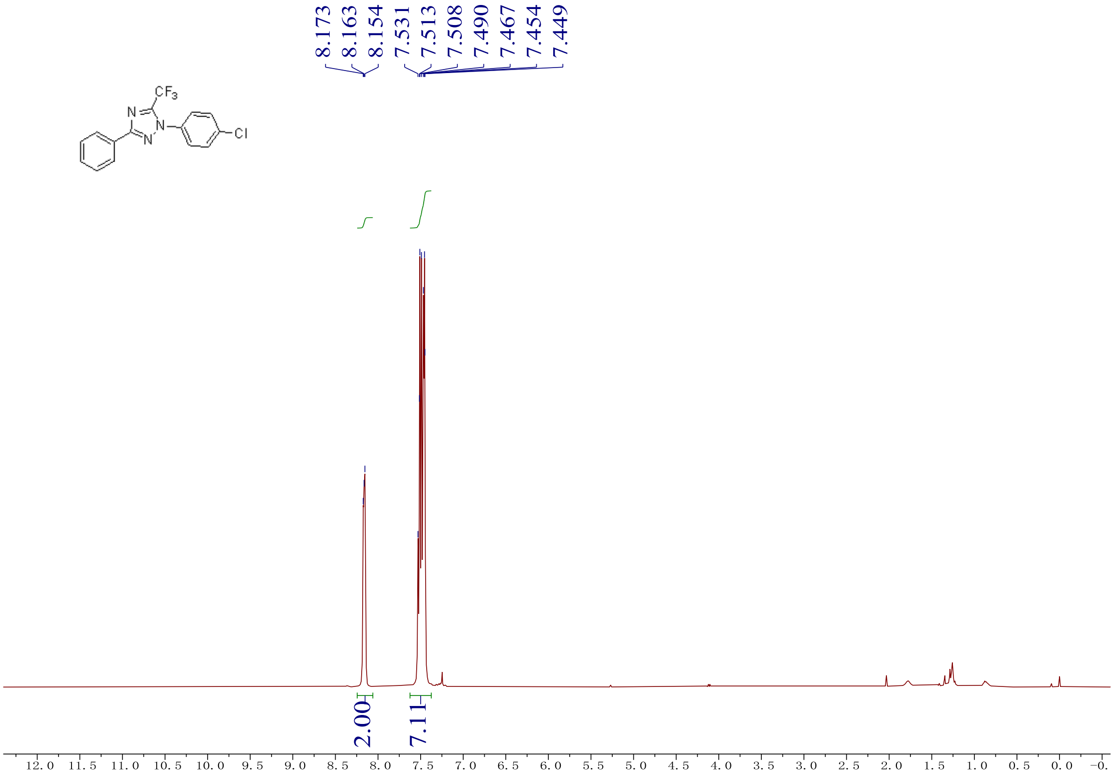

$^{13}C\{^{1}H\}$ NMR spectra of 3z (101 MHz, $CDCl_3$)

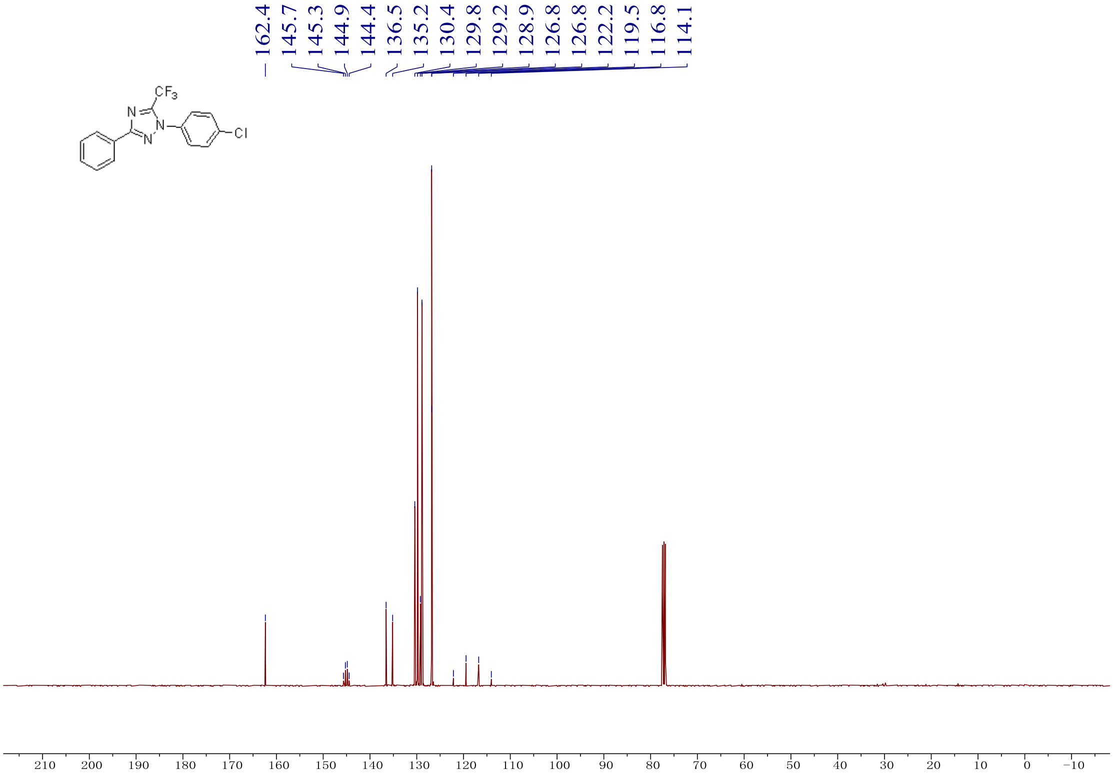

^{19}F NMR spectra of 3z (376 MHz, $CDCl_3$)

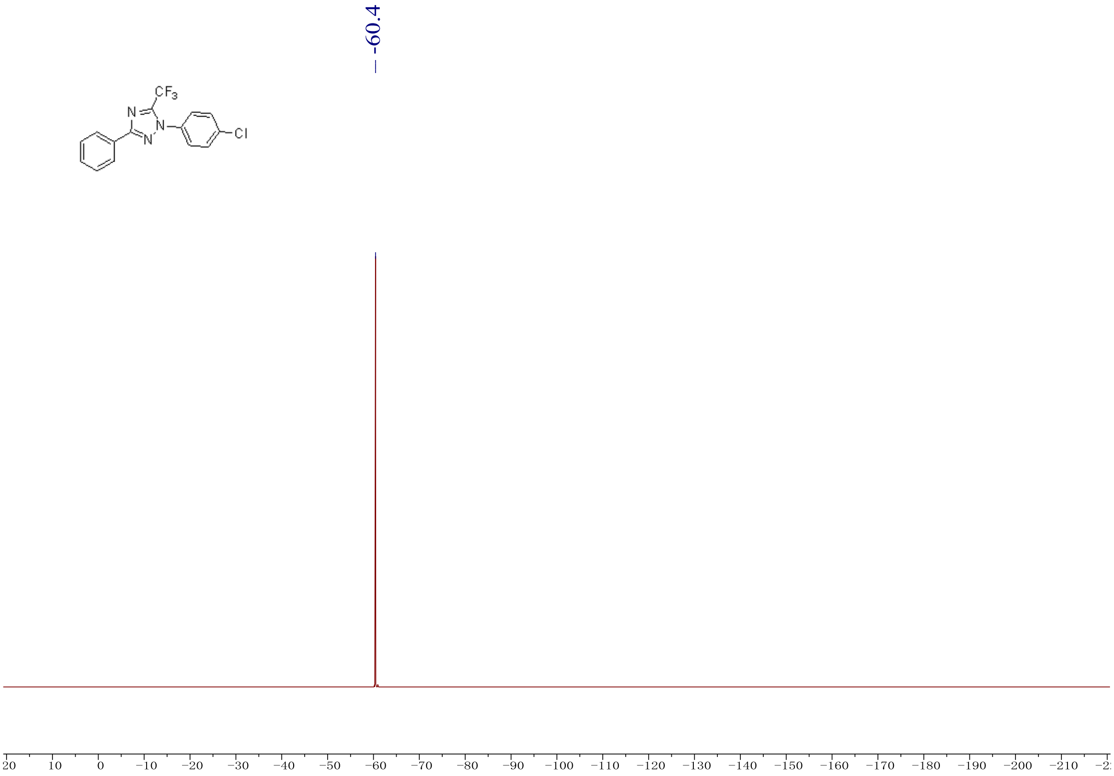

^{1}H NMR spectra of 3aa (400 MHz, $CDCl_3$)

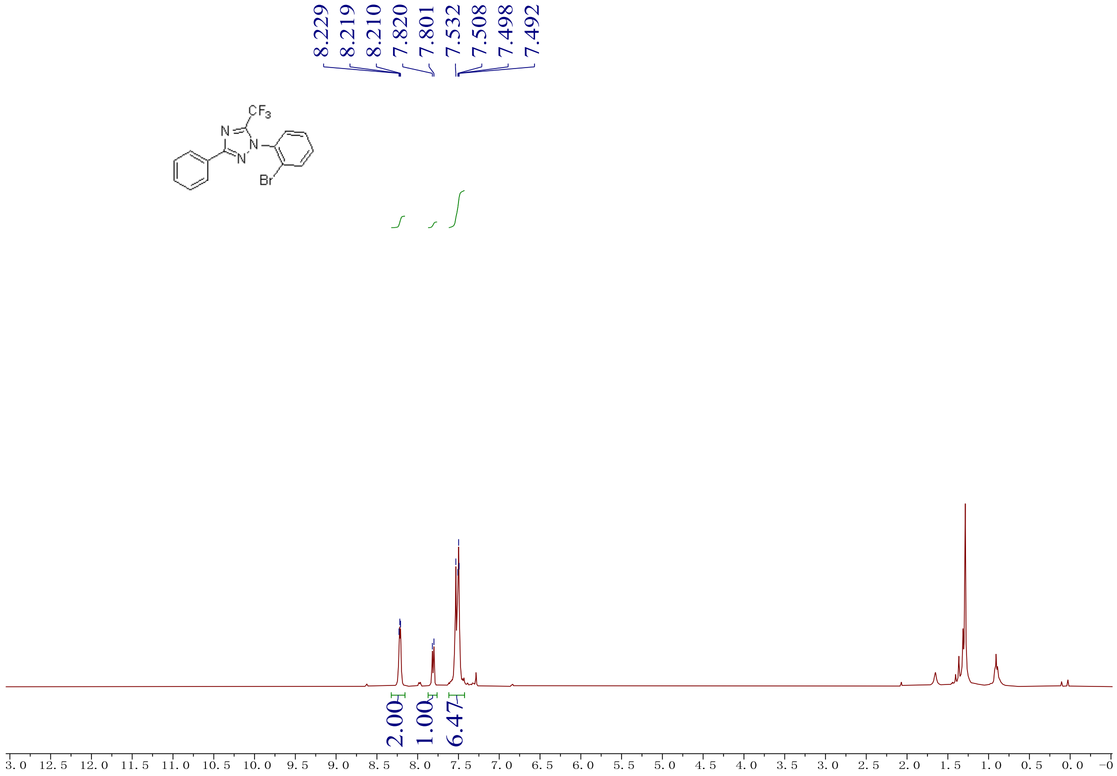

$^{13}C\{^{1}H\}$ NMR spectra of 3aa (101 MHz, $CDCl_3$)

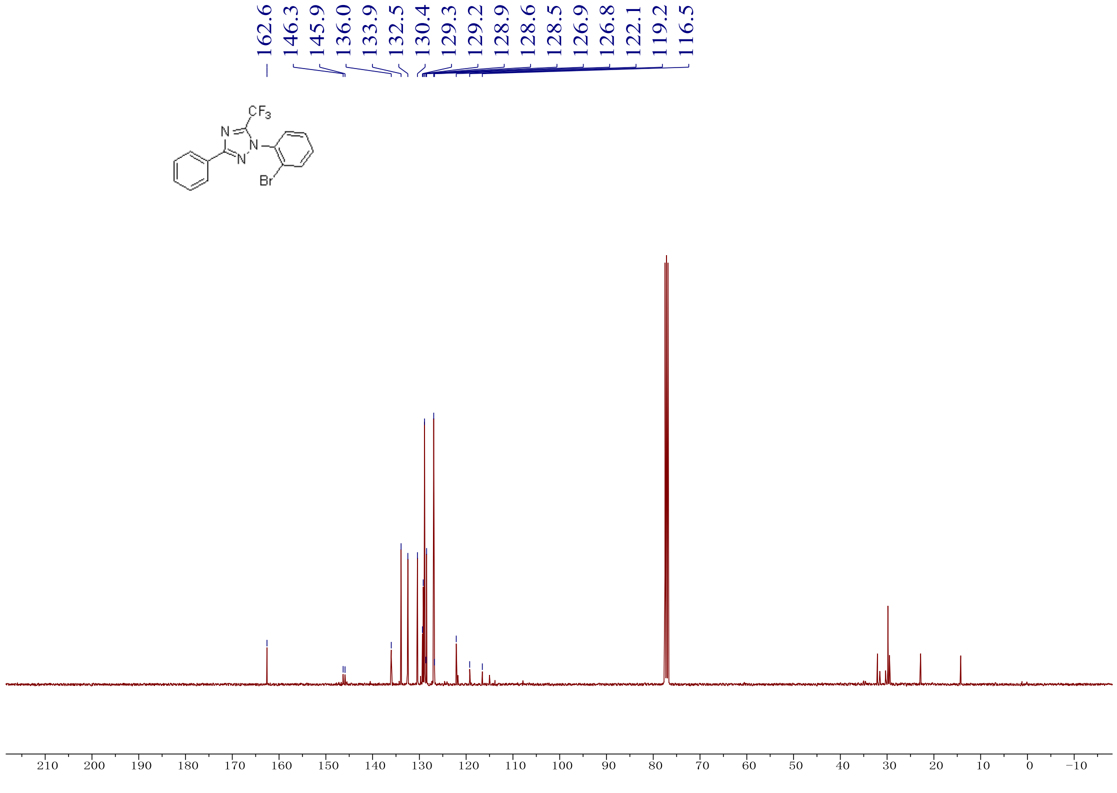

^{19}F NMR spectra of 3aa (376 MHz, $CDCl_3$)

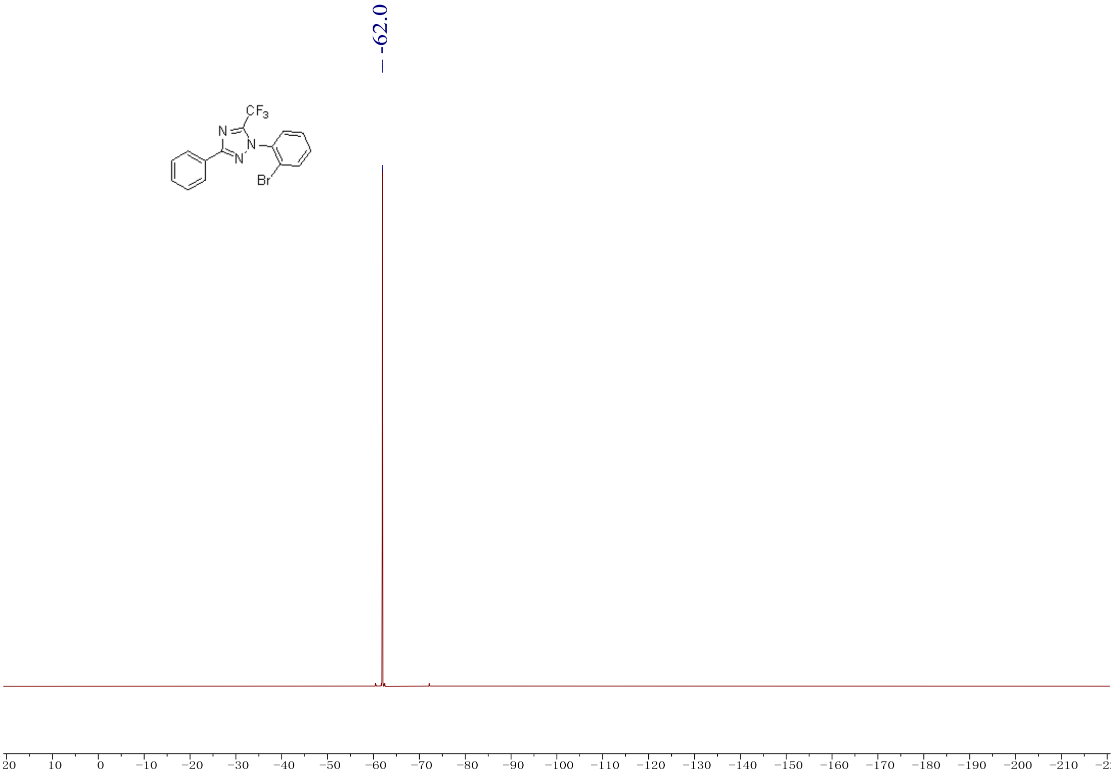

^{1}H NMR spectra of 3ab (400 MHz, $CDCl_3$)

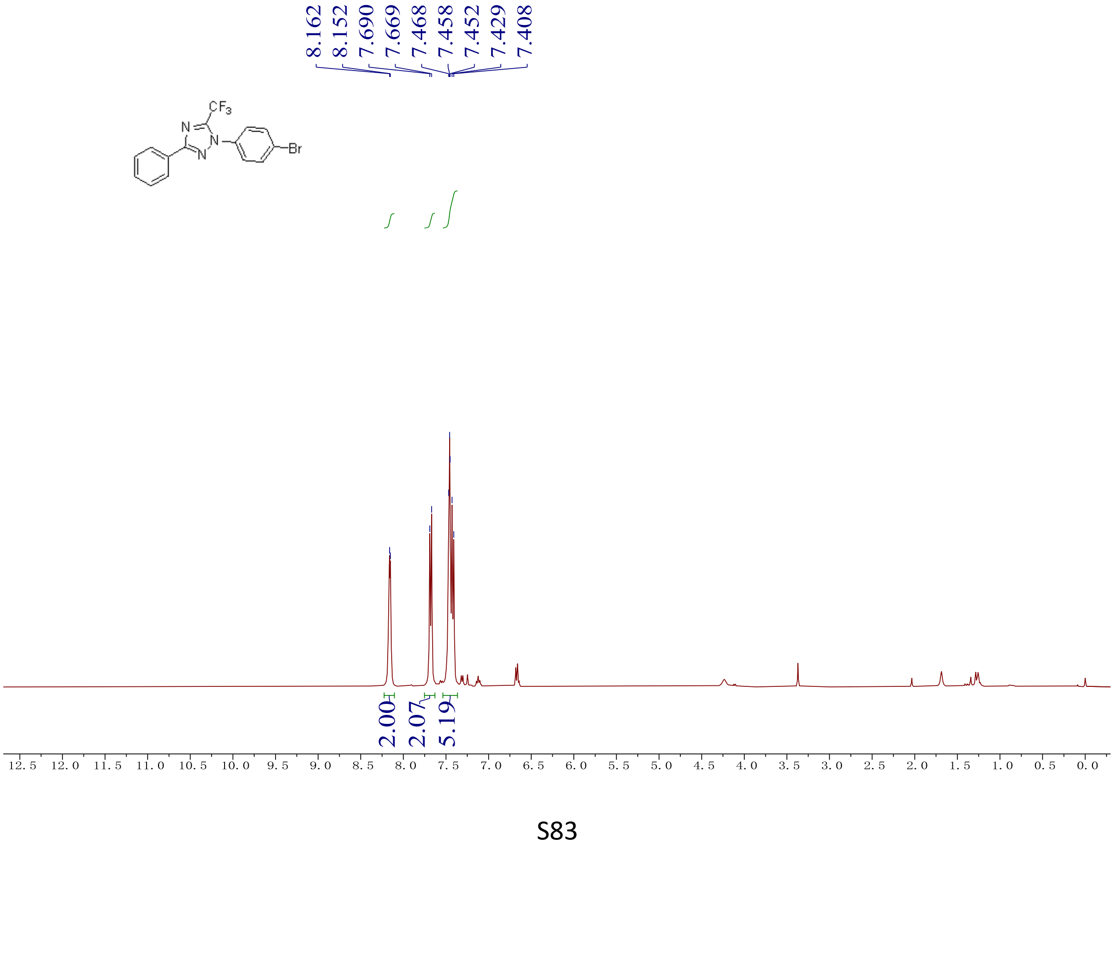

^{13}C{^{1}H} NMR spectra of 3ab (101 MHz, $CDCl_3$)

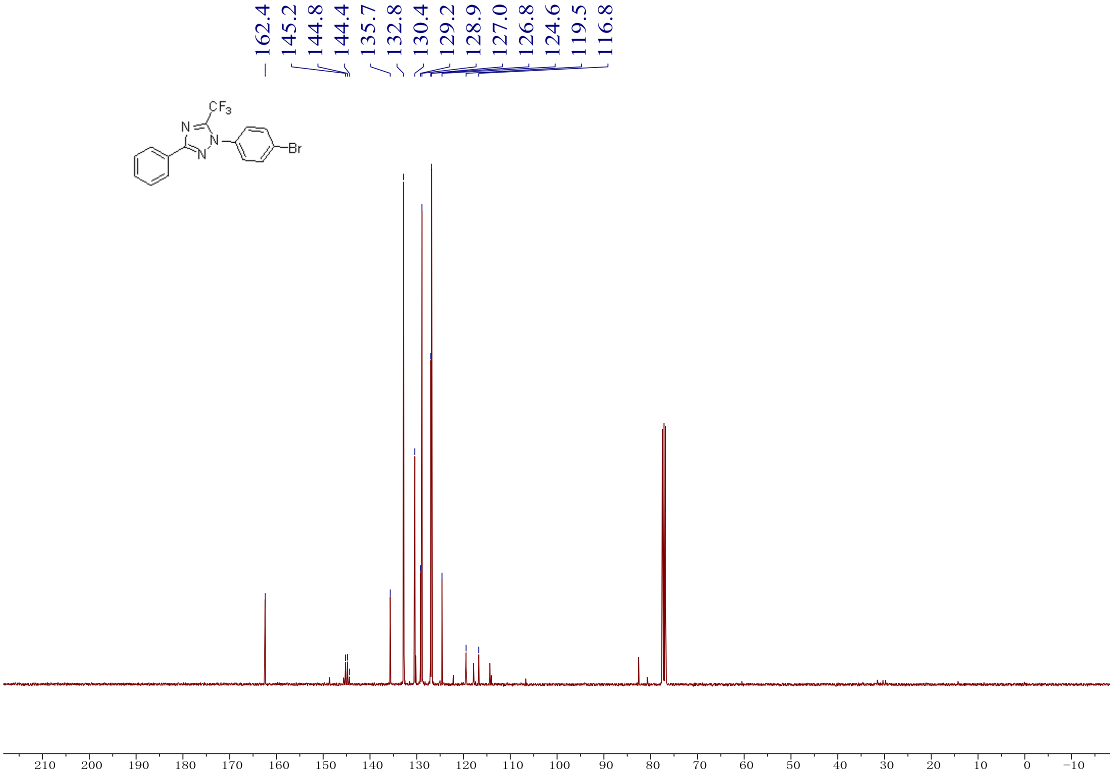

^{19}F NMR spectra of 3ab (376 MHz, $CDCl_3$)

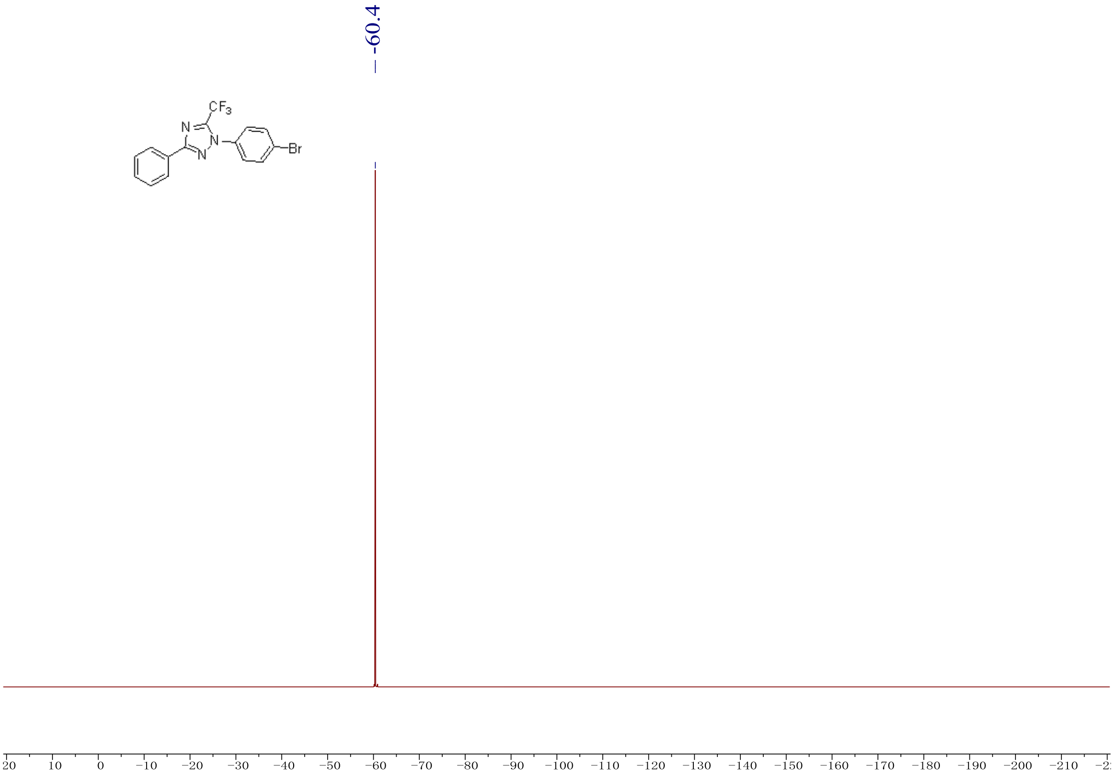

^{1}H NMR spectra of 3ac (400 MHz, $CDCl_3$)

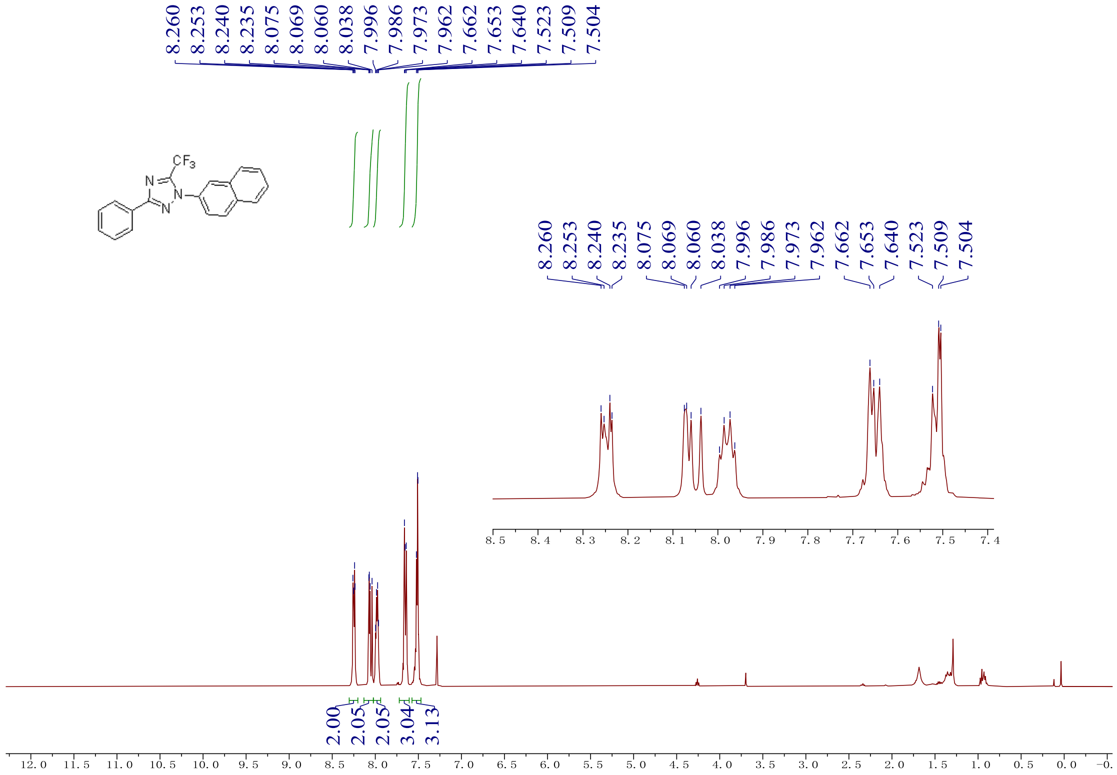

^{13}C{^{1}H} NMR spectra of 3ac (101 MHz, $CDCl_3$)

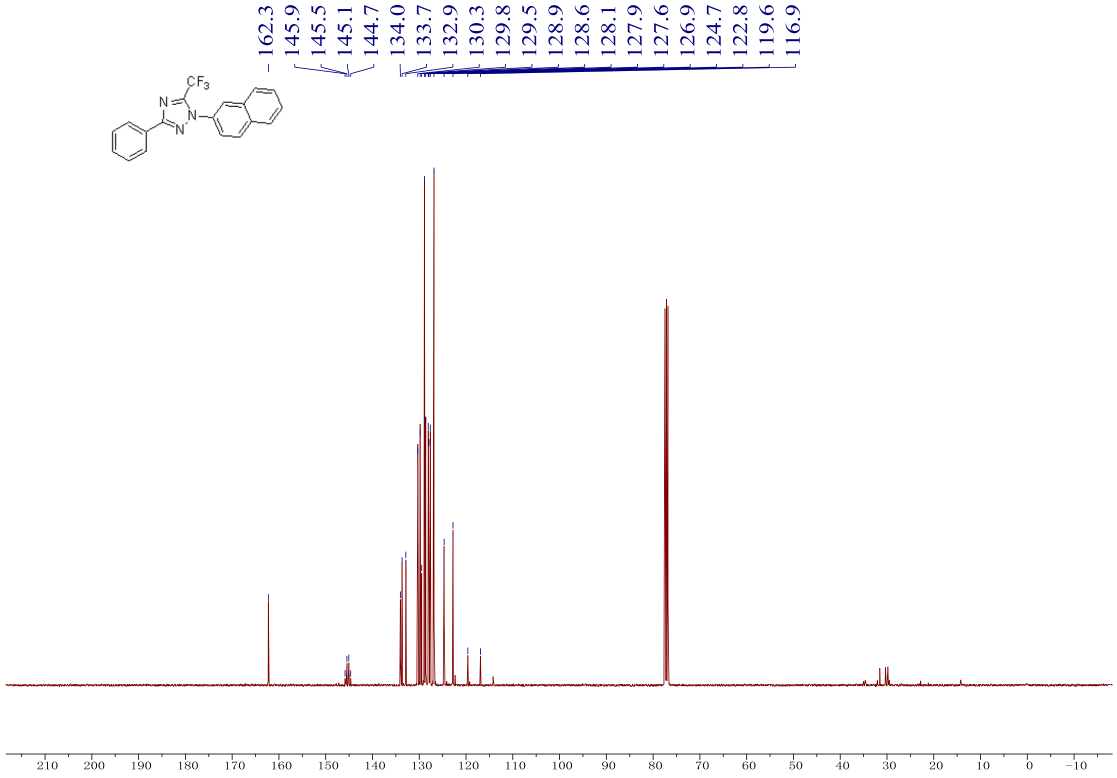

^{19}F NMR spectra of 3ac (376 MHz, $CDCl_3$)

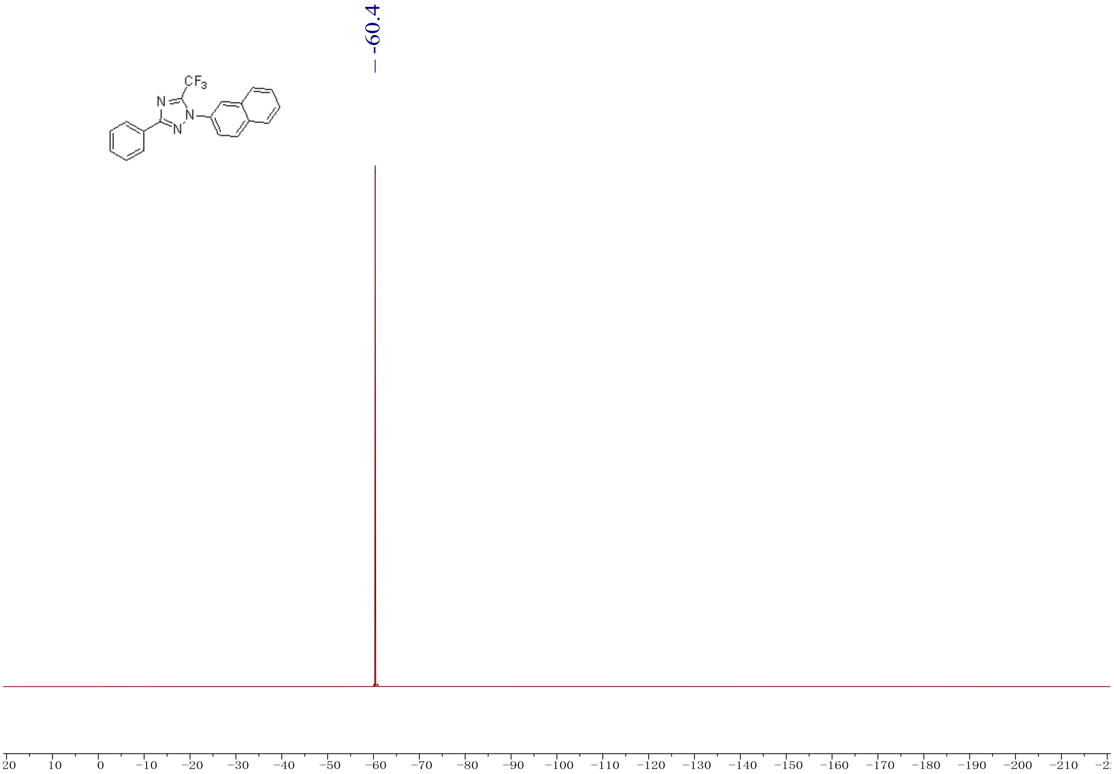

^{1}H NMR spectra of 5a (400 MHz, $CDCl_3$)

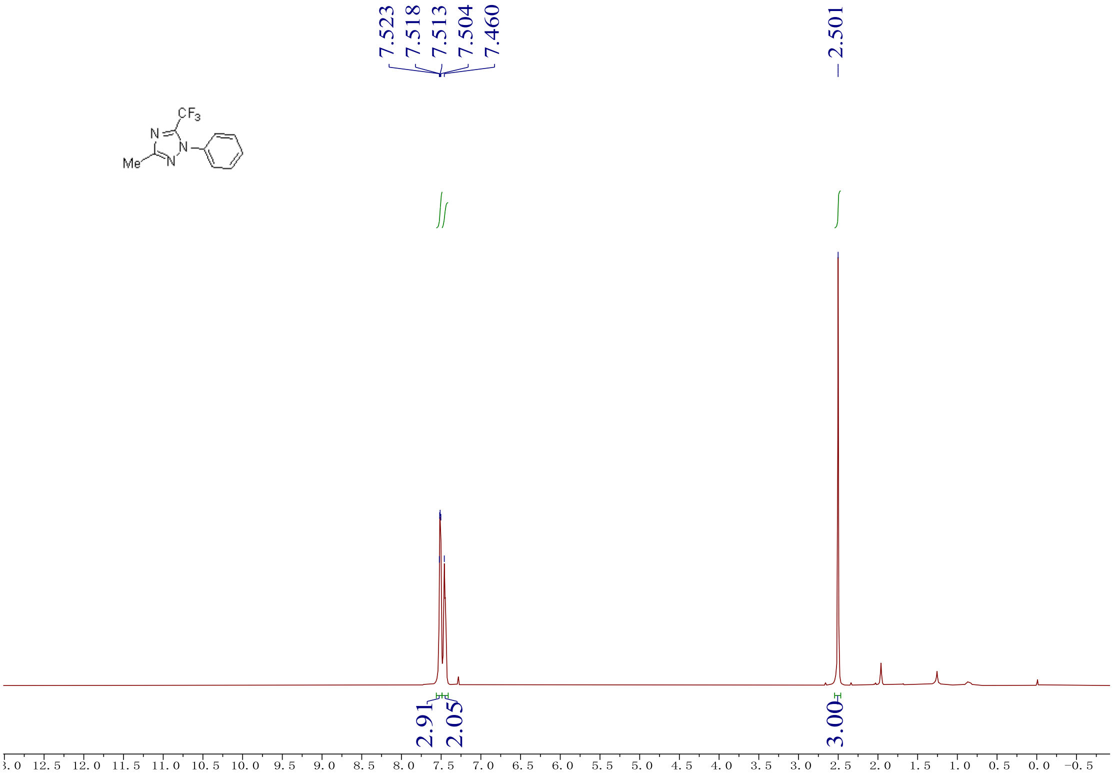

$^{13}C\{^{1}H\}$ NMR spectra of 5a (101 MHz, $CDCl_3$)

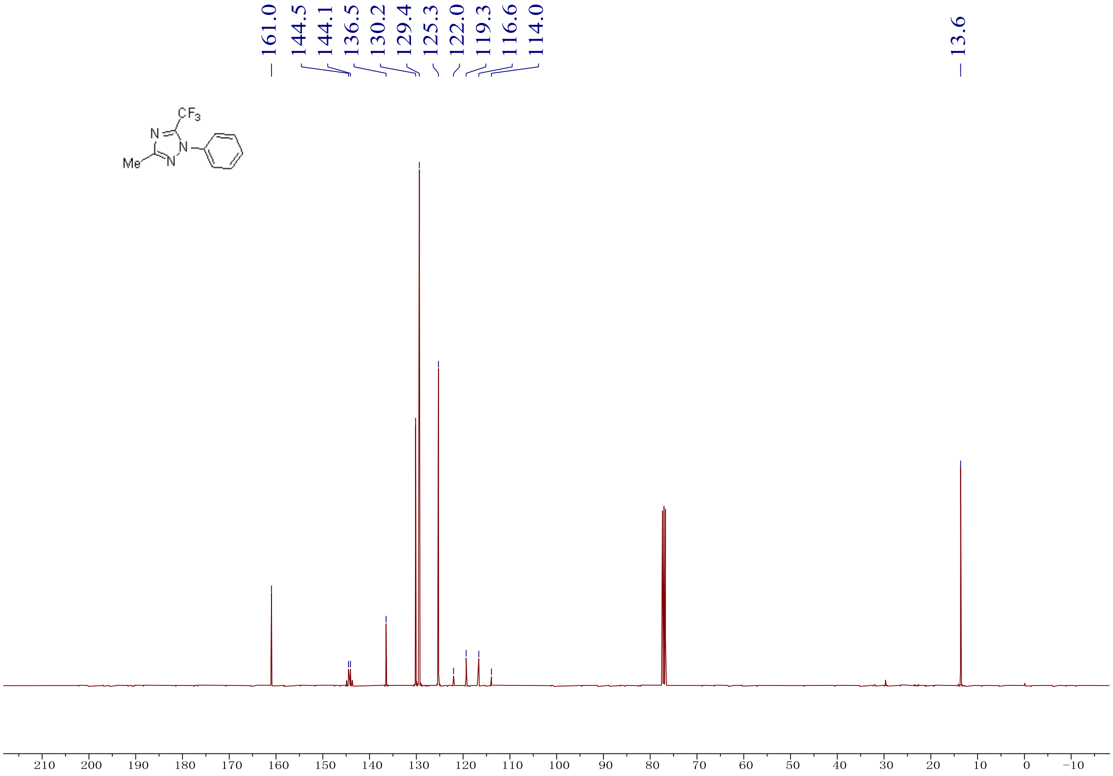

^{19}F NMR spectra of 5a (376 MHz, $CDCl_3$)

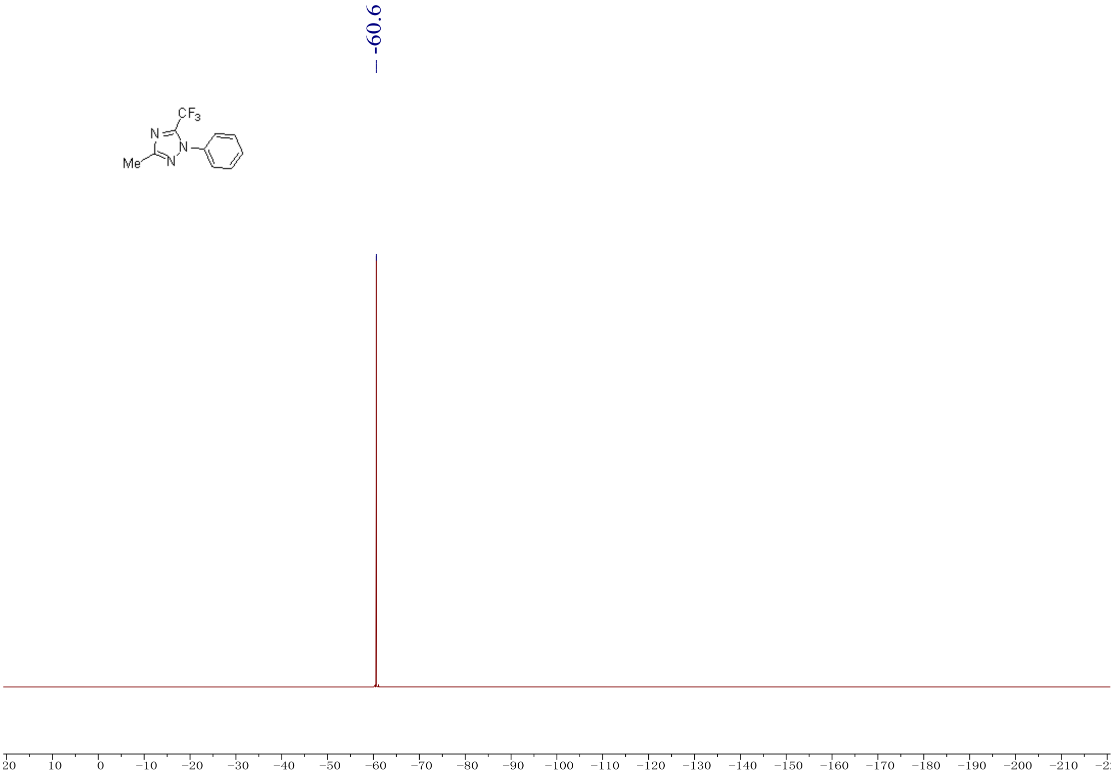

^{1}H NMR spectra of 5b (400 MHz, $CDCl_3$)

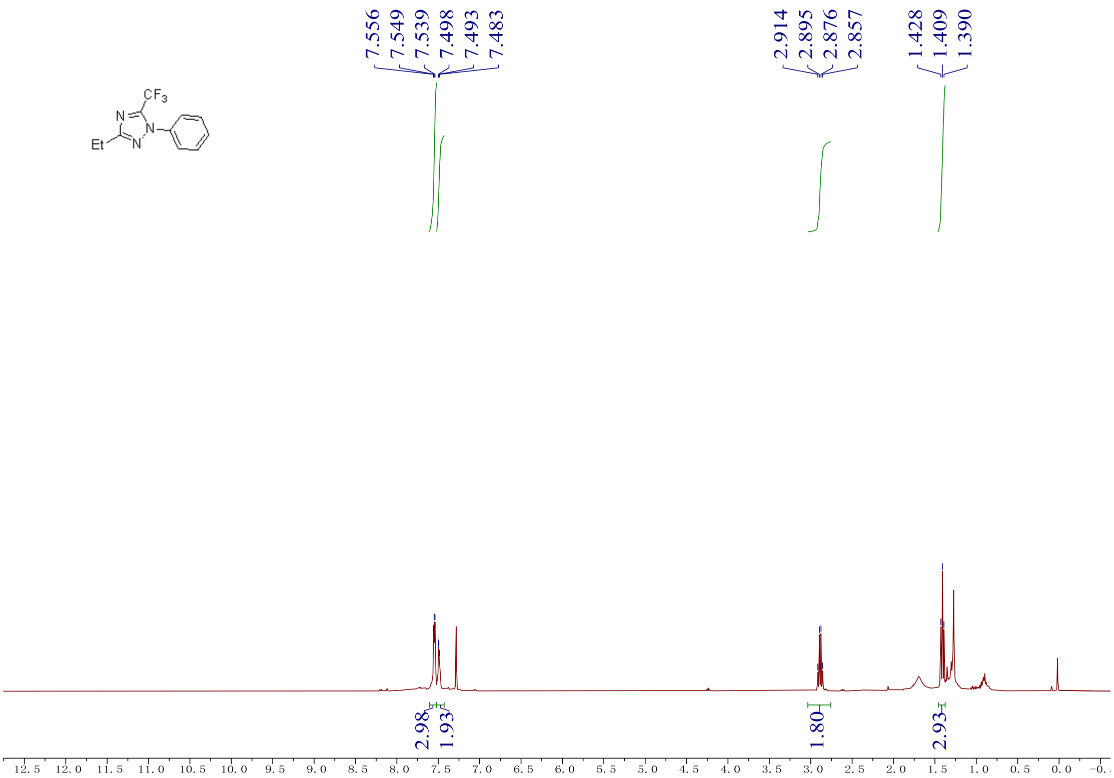

$^{13}C\{^{1}H\}$ NMR spectra of 5b (101 MHz, $CDCl_3$)

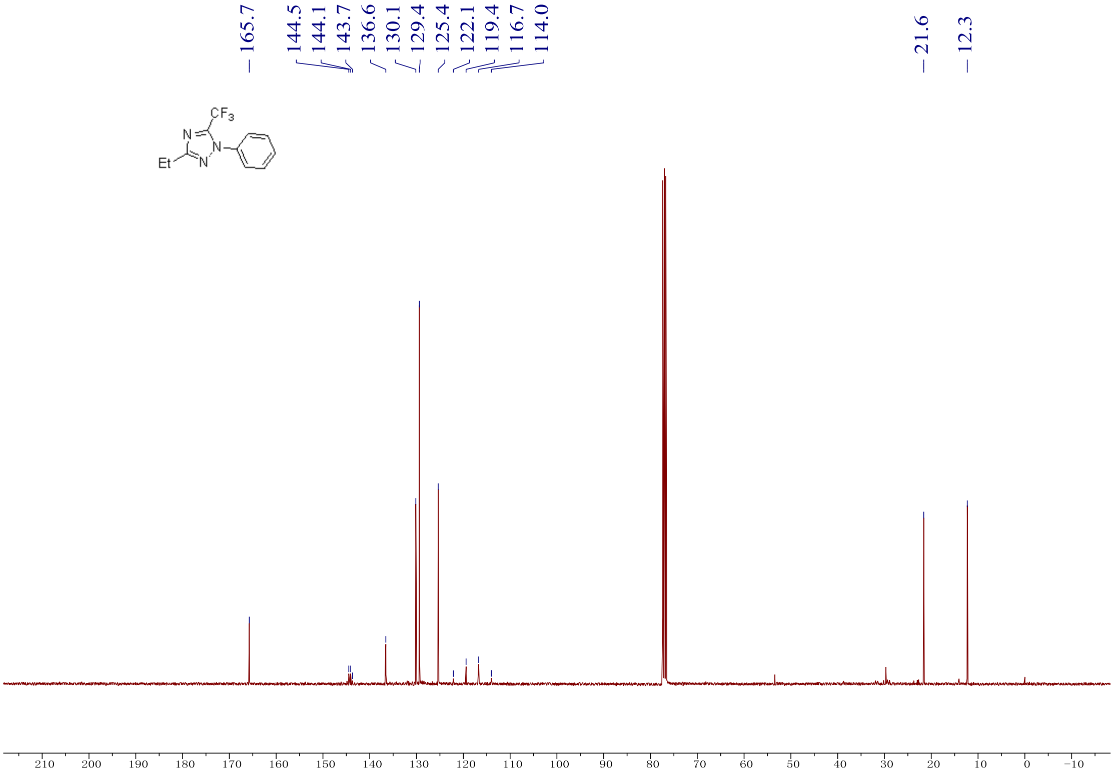

^{19}F NMR spectra of 5b (376 MHz, $CDCl_3$)

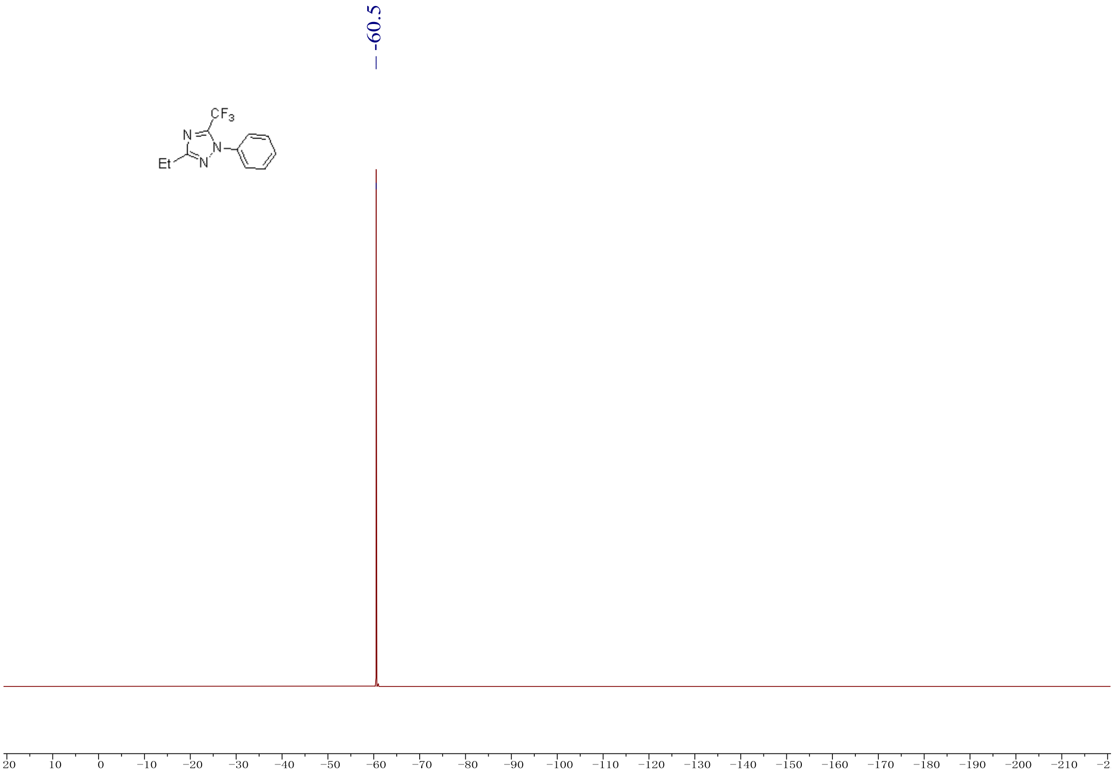

^{1}H NMR spectra of 5c (400 MHz, $CDCl_3$)

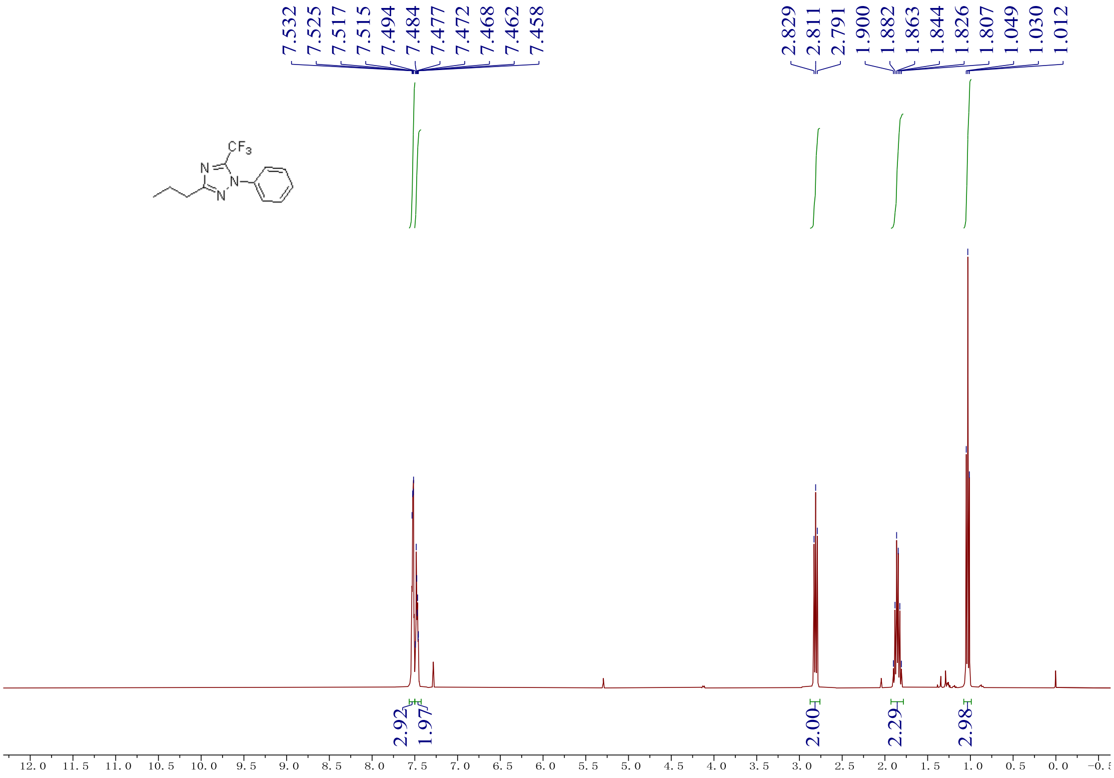

$^{13}C\{^{1}H\}$ NMR spectra of 5c (101 MHz, $CDCl_3$)

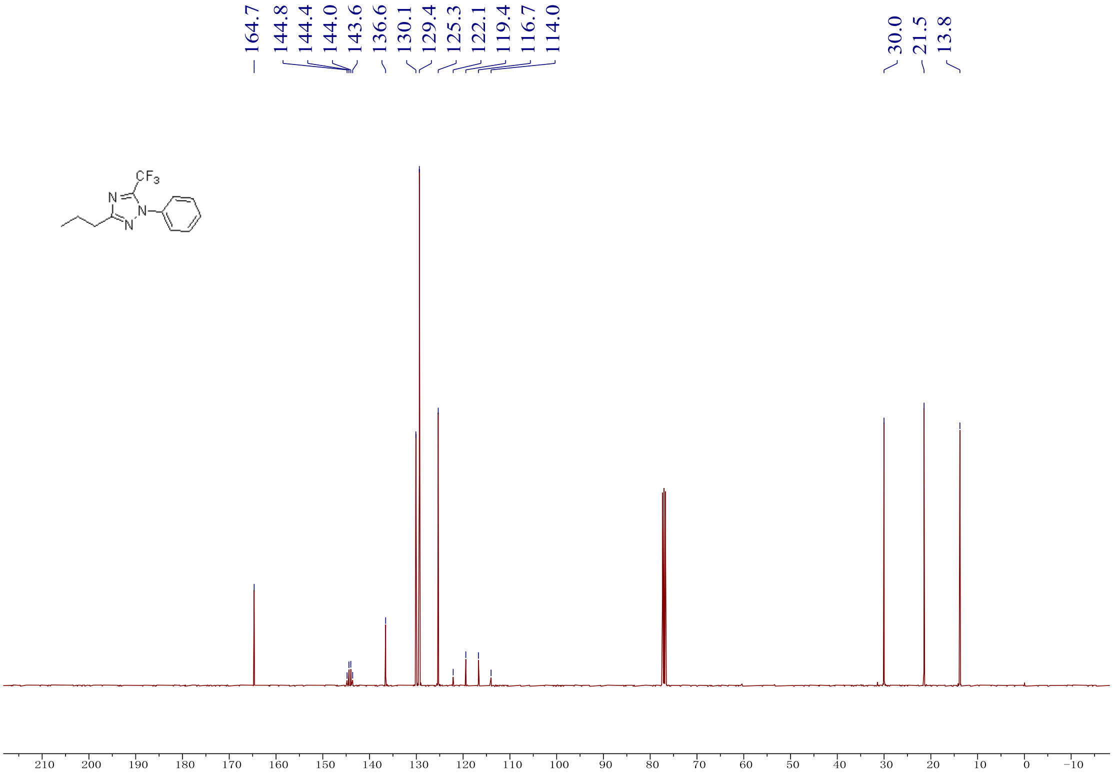

^{19}F NMR spectra of 5c (376 MHz, $CDCl_3$)

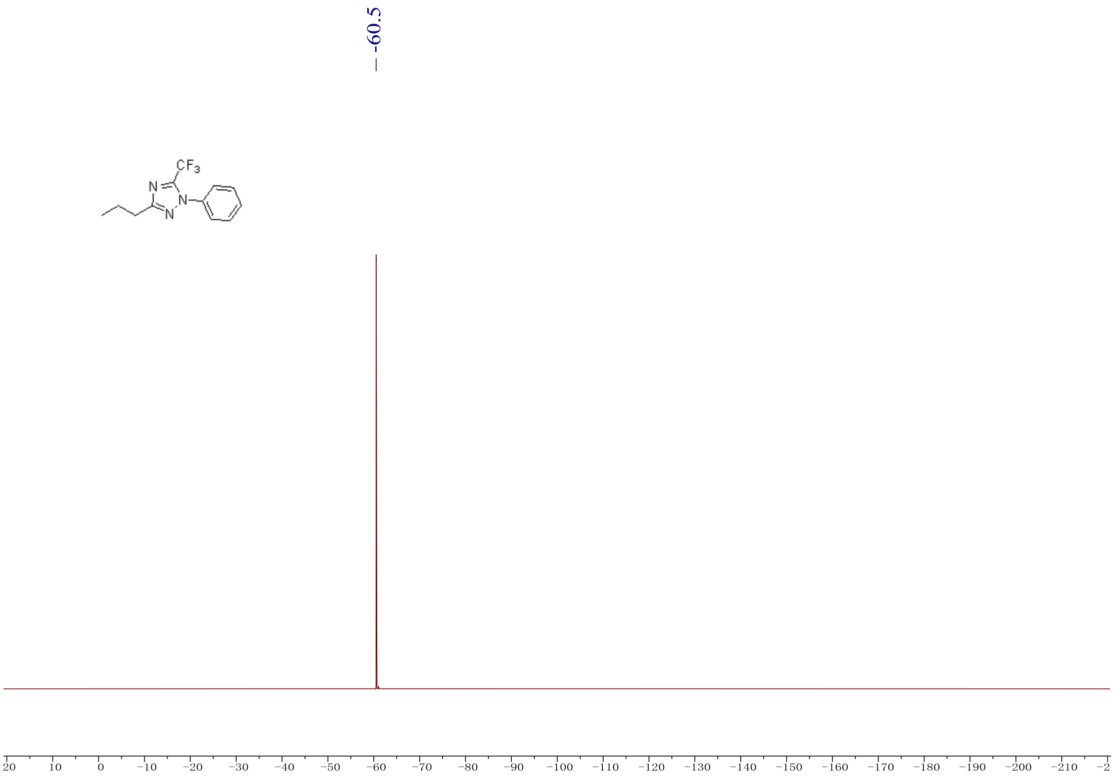

^{1}H NMR spectra of 5e (400 MHz, $CDCl_3$)

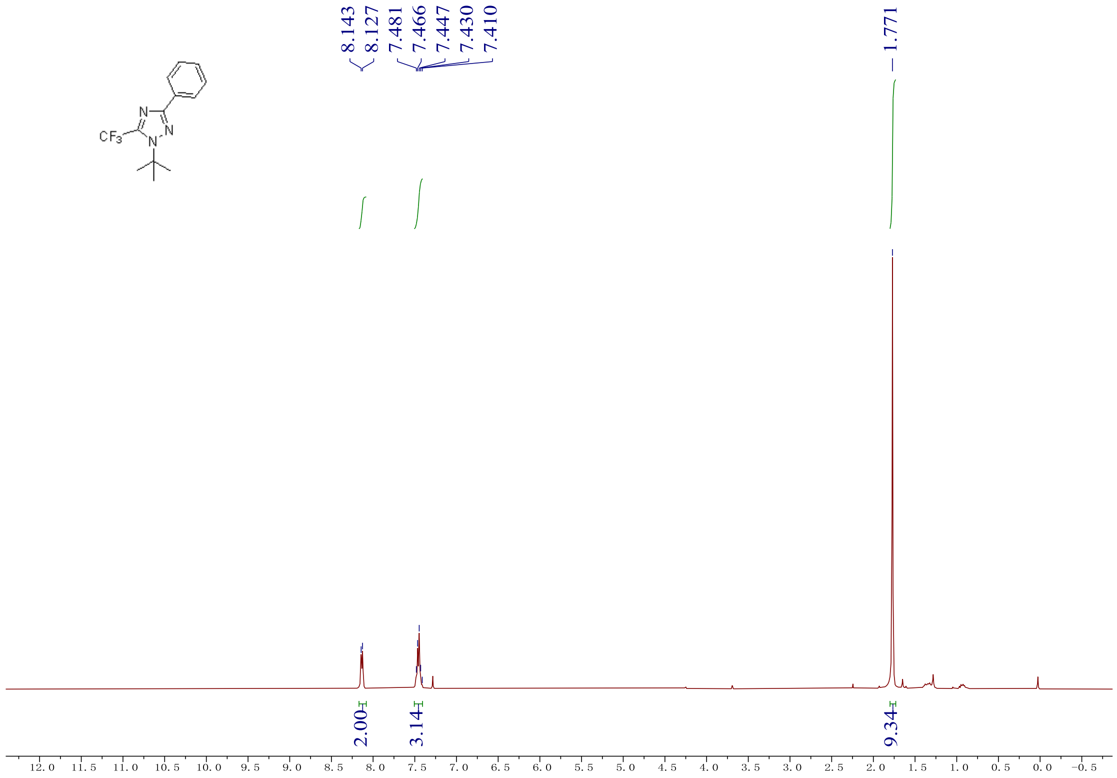

$^{13}C\{^{1}H\}$ NMR spectra of 5e (101 MHz, $CDCl_3$)

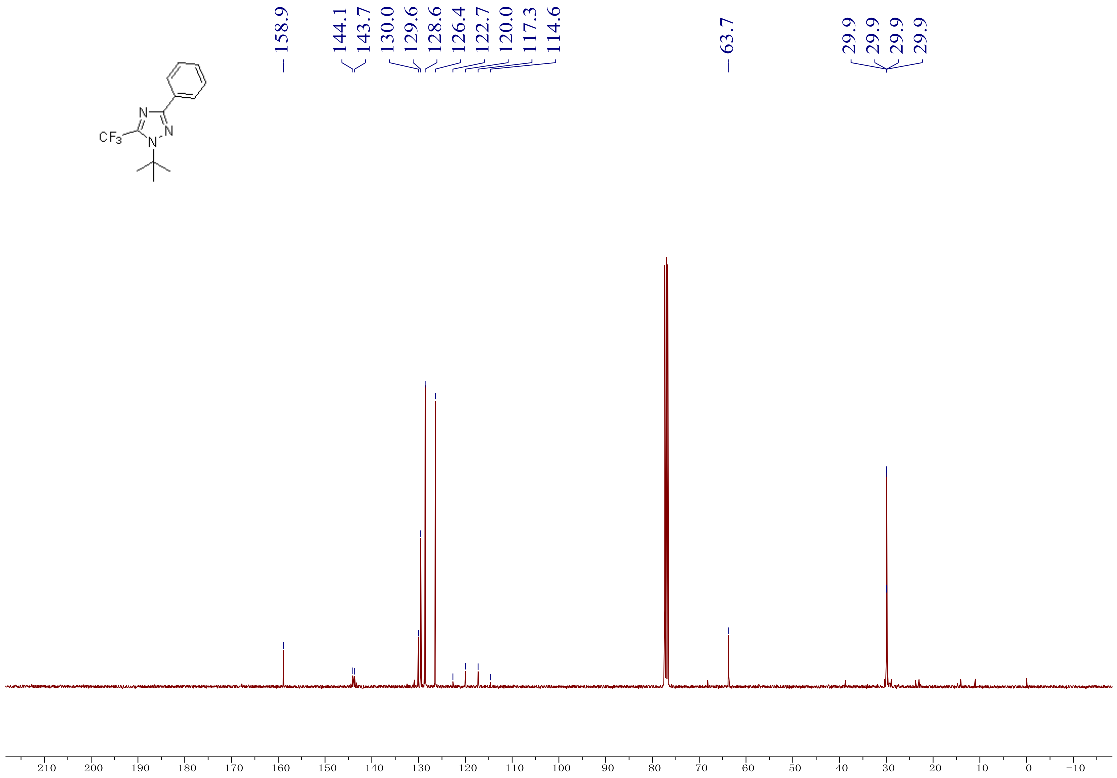

^{19}F NMR spectra of 5e (376 MHz, $CDCl_3$)

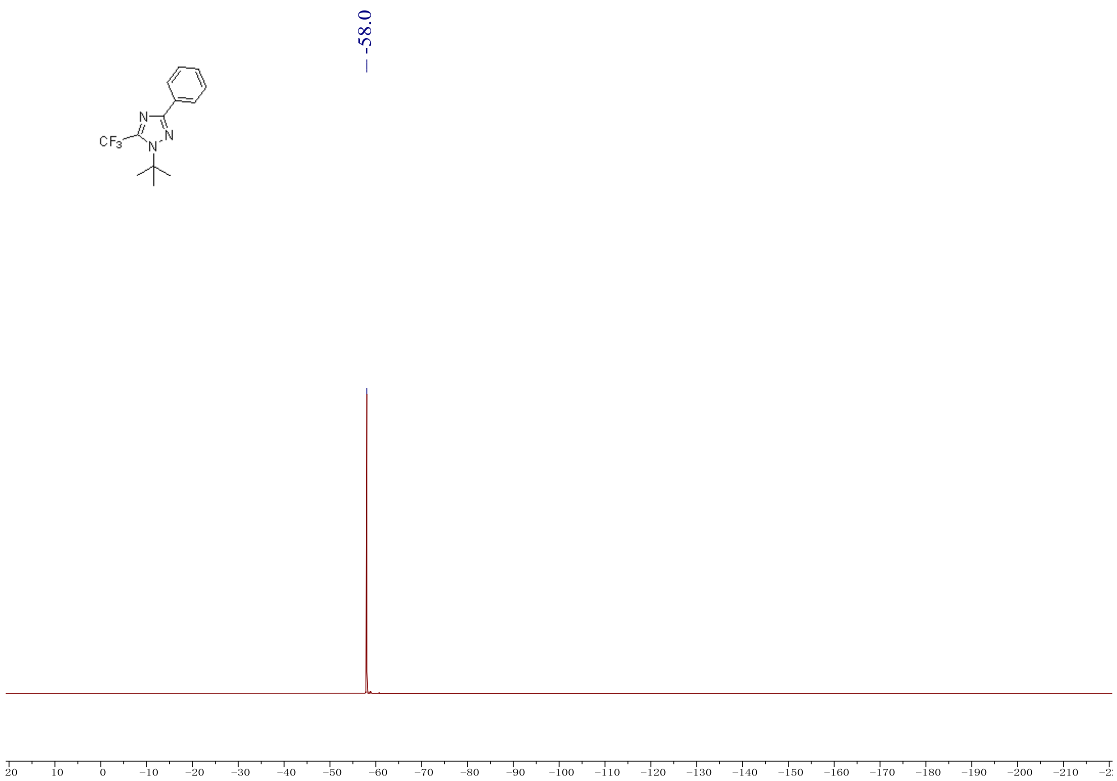

^{1}H NMR spectra of 6 (400 MHz, $CDCl_3$)

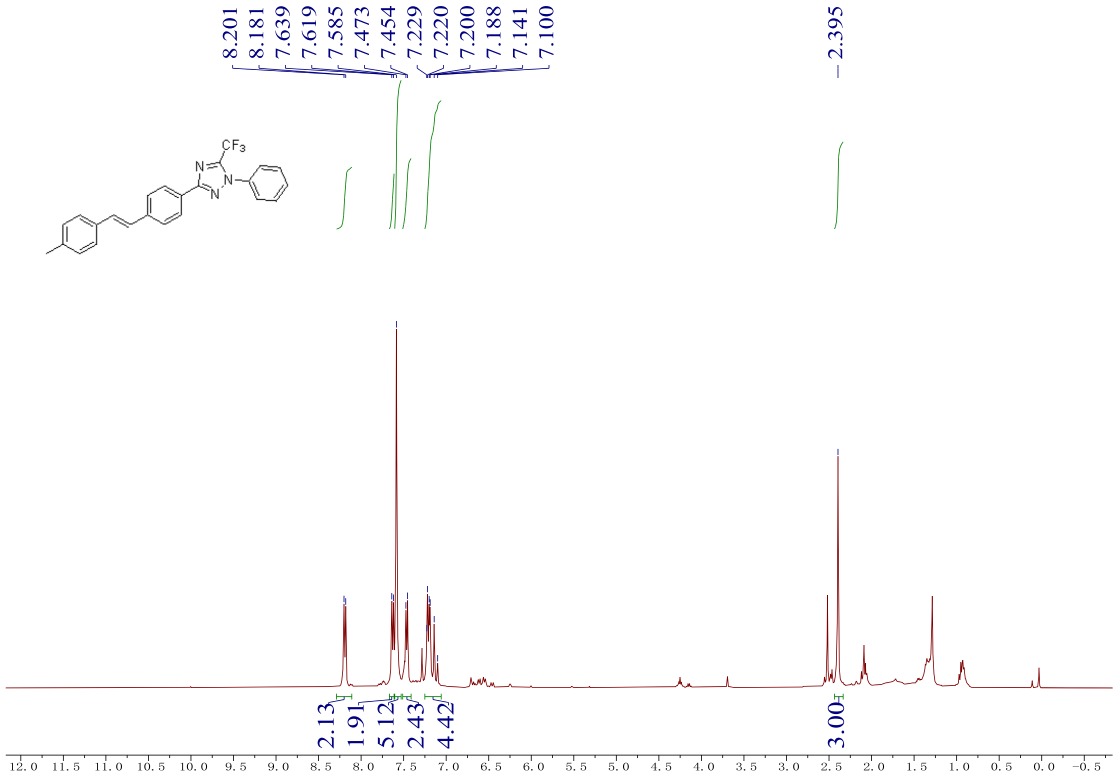

$^{13}C\{^1H\}$ NMR spectra of 6 (101 MHz, $CDCl_3$)

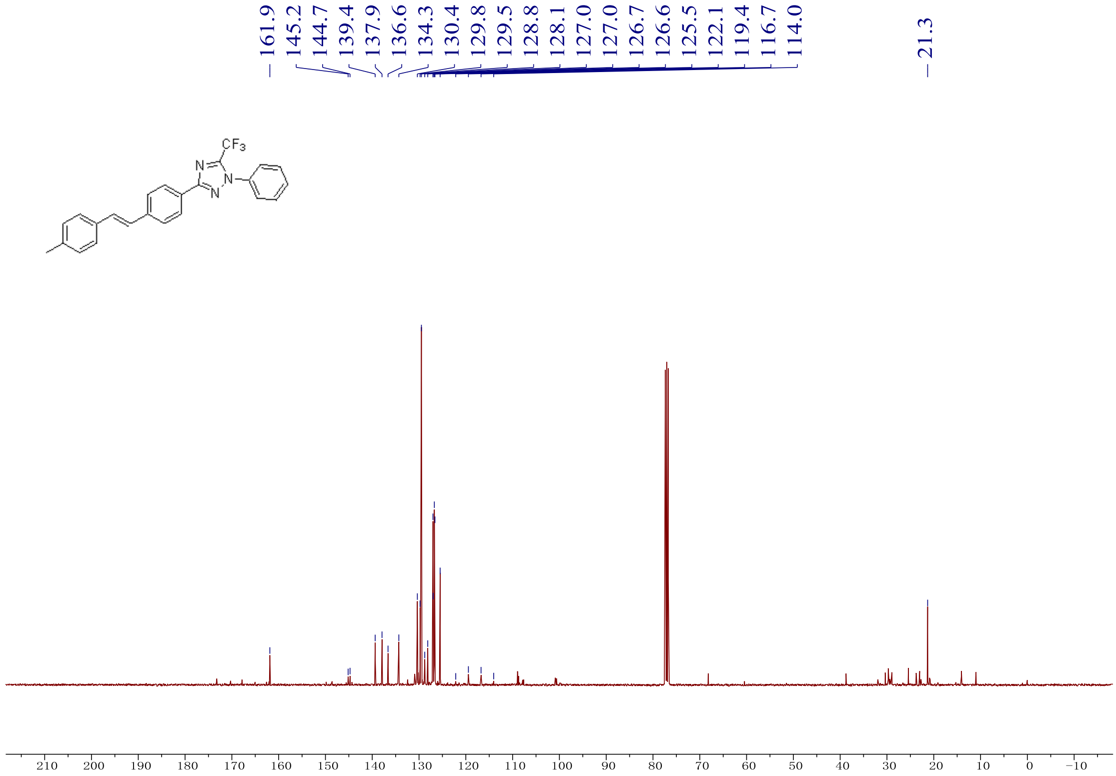

^{19}F NMR spectra of 6 (376 MHz, $CDCl_3$)

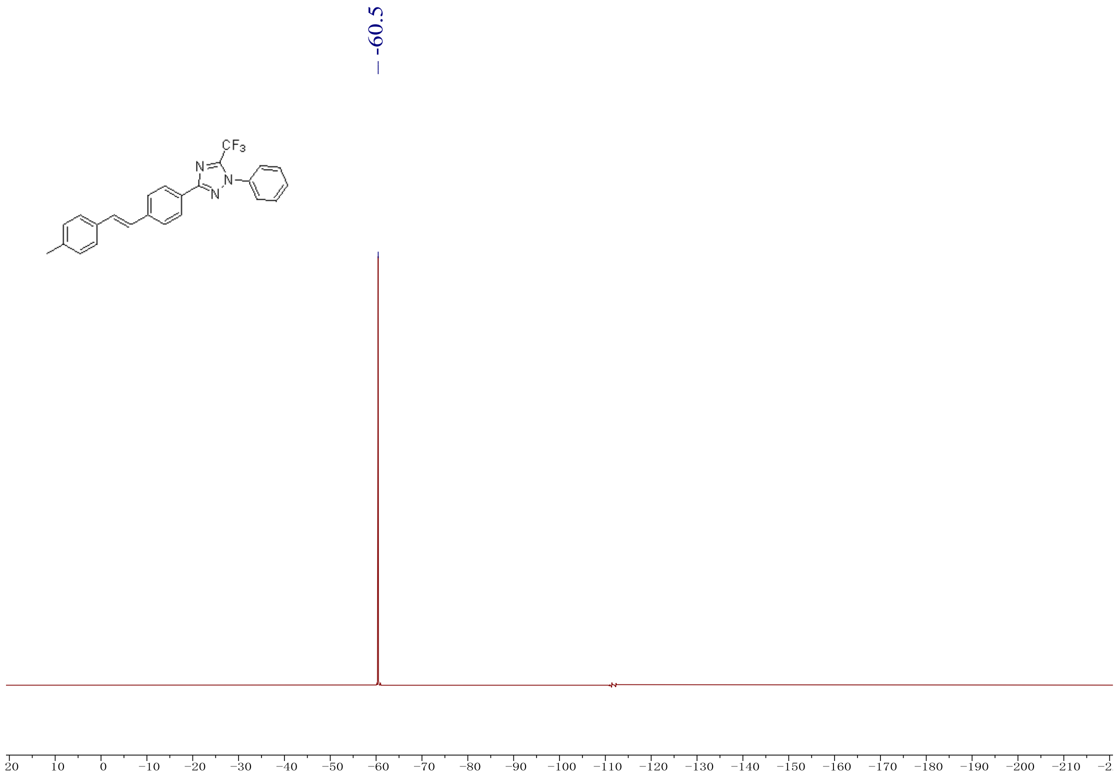

^{1}H NMR spectra of 7 (400 MHz, $CDCl_3$)

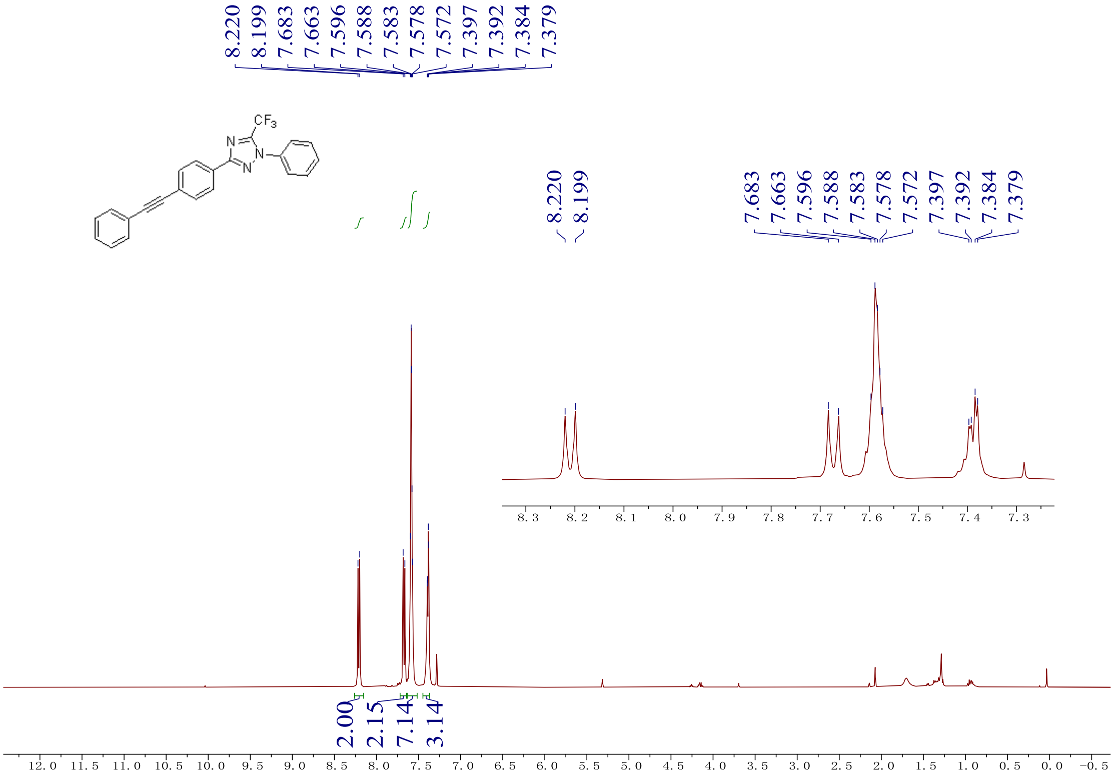

$^{13}C\{^1H\}$ NMR spectra of 7 (101 MHz, $CDCl_3$)

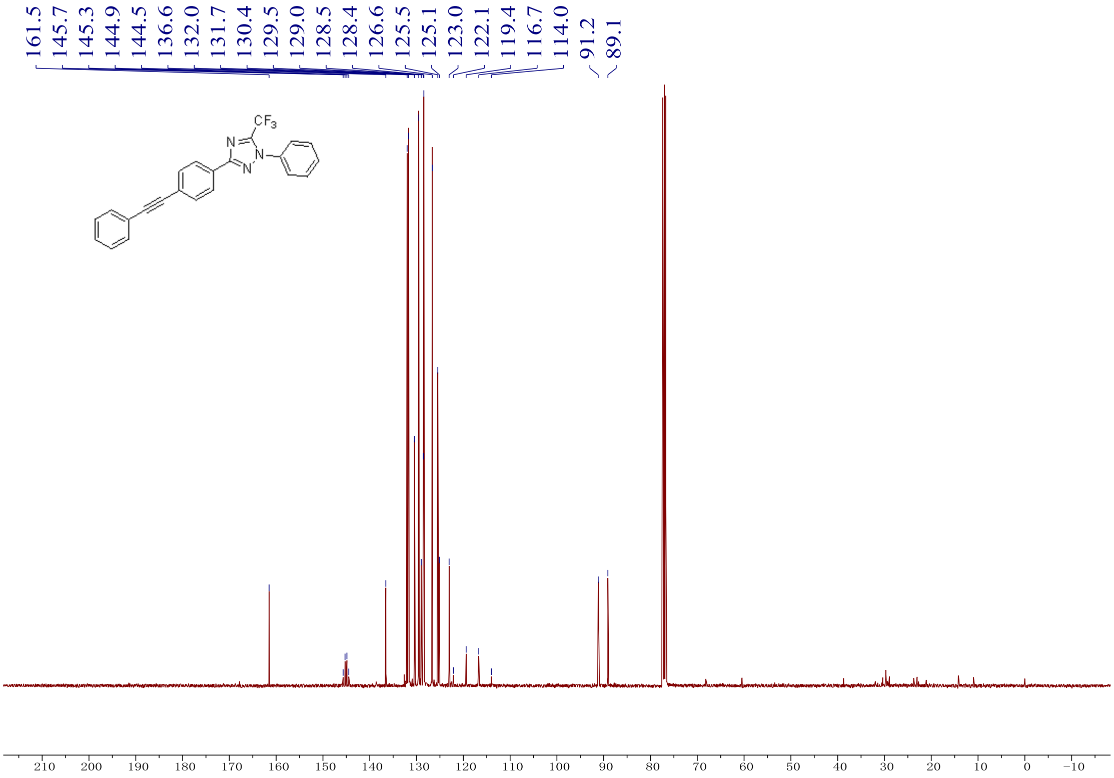

^{19}F NMR spectra of 7 (376 MHz, $CDCl_3$)

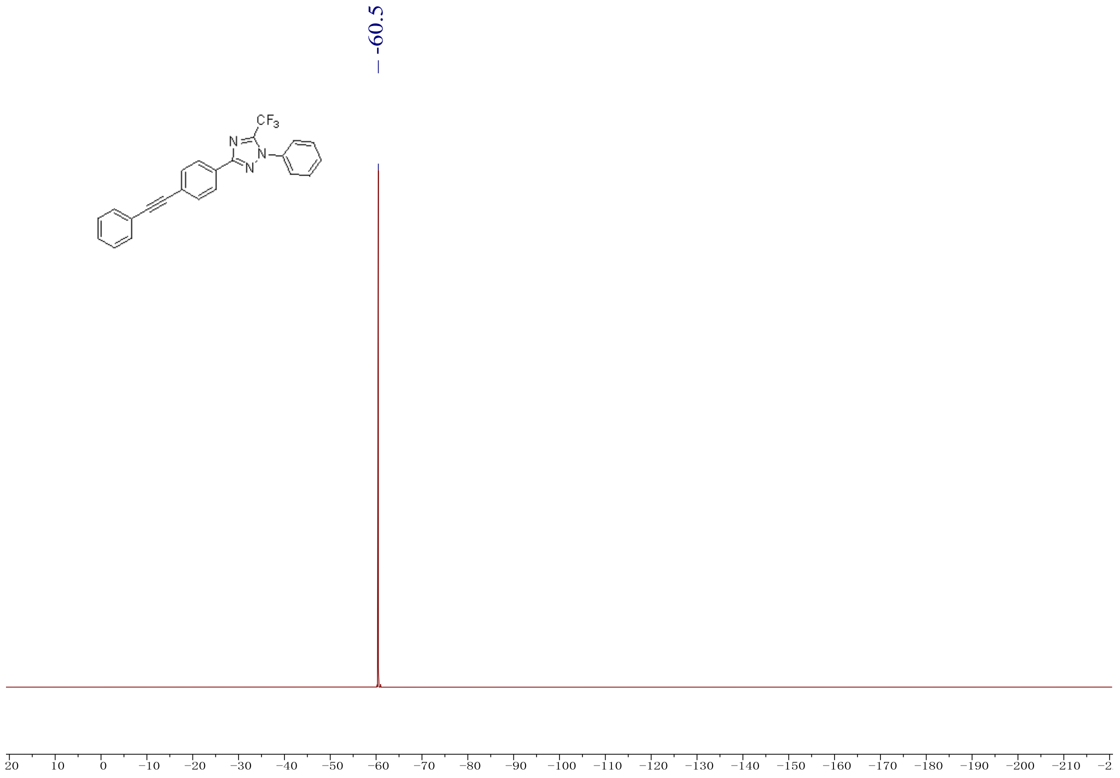

^{1}H NMR spectra of 8 (400 MHz, $CDCl_3$)

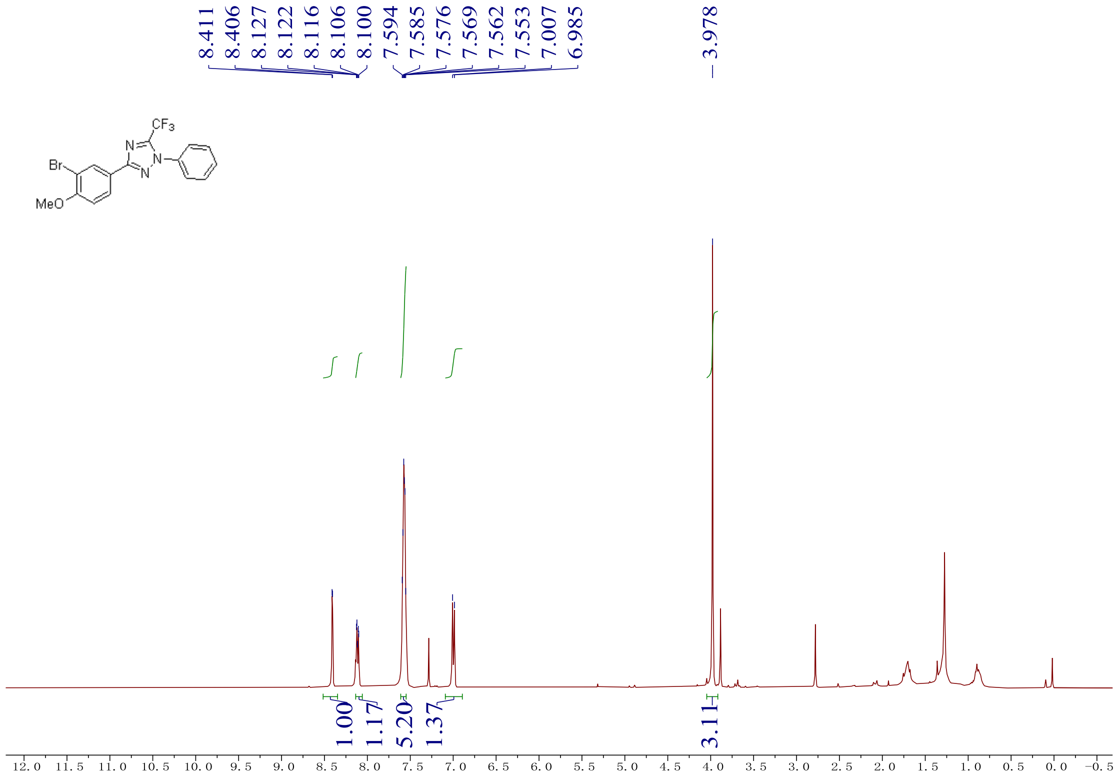

$^{13}C\{^1H\}$ NMR spectra of 8 (101 MHz, $CDCl_3$)

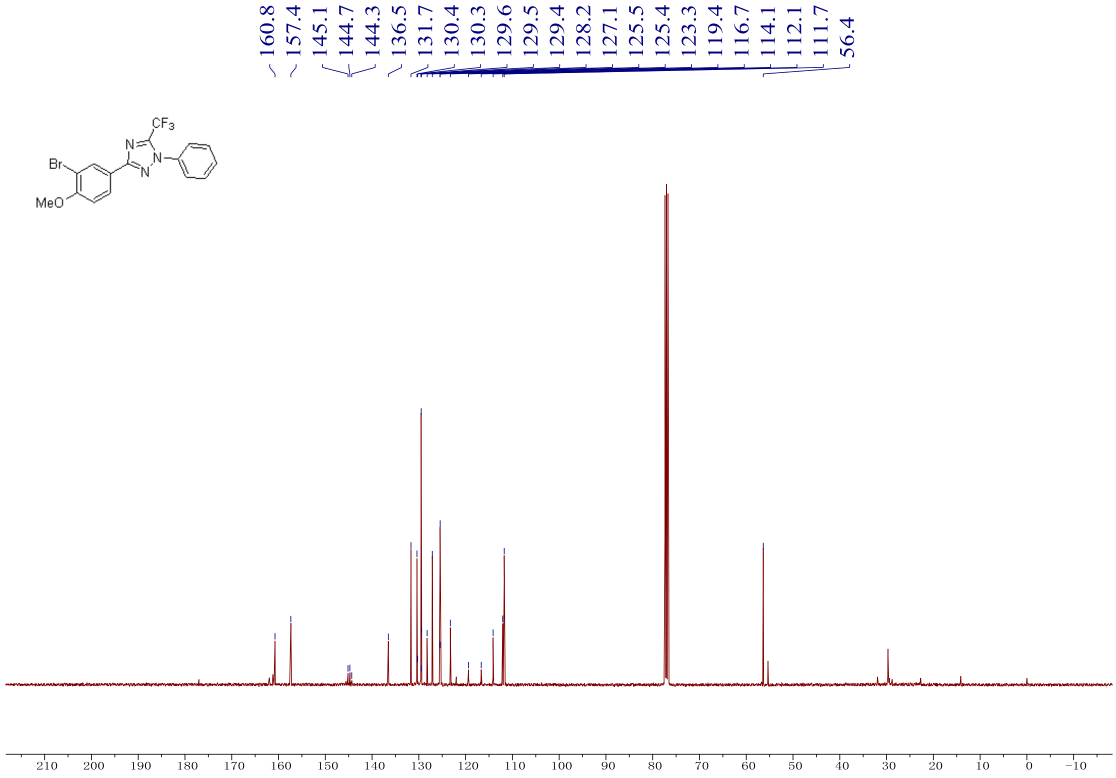

^{19}F NMR spectra of 8 (376 MHz, $CDCl_3$)

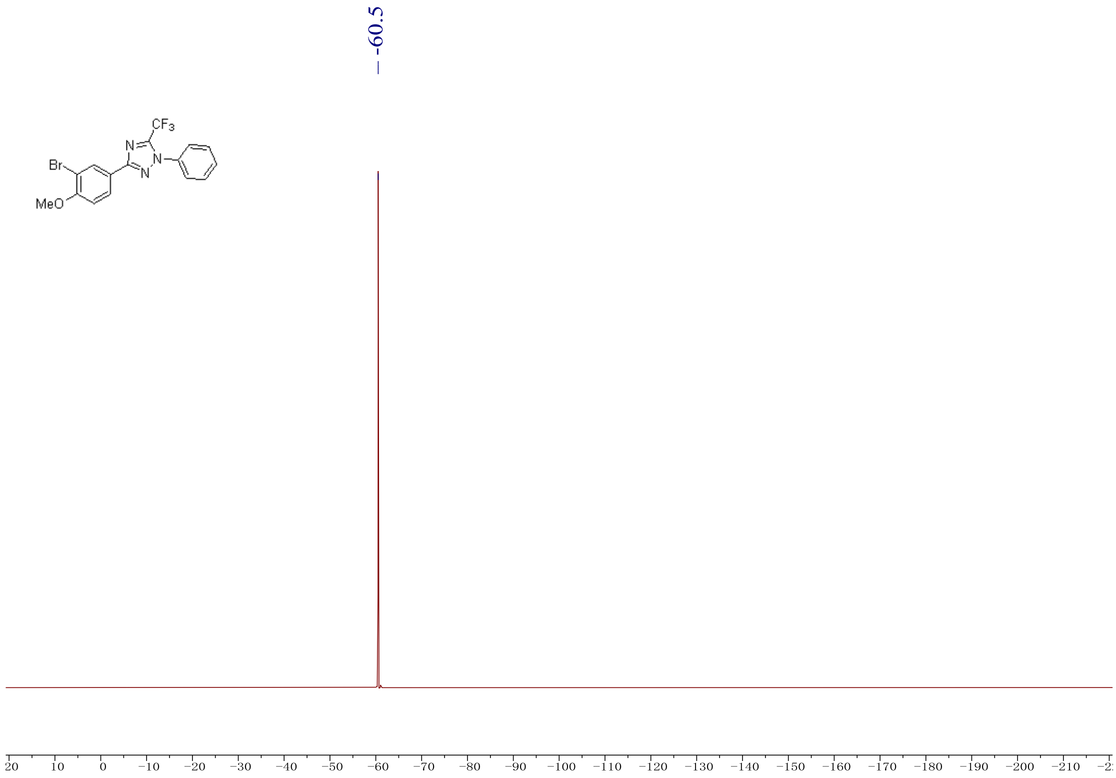

Supplement: Supplementary file 1 [file molecules-27-06568-s001.zip › molecules-1935243-supplementary.pdf]
